# Network Pharmacology and Molecular Docking-Based Approach Revealing the Potential Anticancer Compounds and Molecular Mechanisms of *Paris polyphylla* Against Colorectal Cancer

**DOI:** 10.3390/ijms27093874

**Published:** 2026-04-27

**Authors:** Chakkrit Khanaree, Ratchanon Inpan, Weerakit Taychaworaditsakul, Nahathai Dukeaw

**Affiliations:** 1 School of Traditional and Alternative Medicine, Chiang Rai Rajabhat University, Chiang Rai 57100, Thailand; chakkrit.kh@gmail.com; 2 Clinical Research Center for Food and Herbal Product Trials and Development (CR-FAH), Faculty of Medicine, Chiang Mai University, Chiang Mai 50200, Thailand; ratchanon.inpan@gmail.com; 3 Department of Pharmacology, Faculty of Medicine, Chiang Mai University, Chiang Mai 50200, Thailand; 4 Office of Research Administration, Chiang Mai University, Chiang Mai 50200, Thailand; 5 Department of Biochemistry, Faculty of Medicine, Chiang Mai University, Chiang Mai 50200, Thailand; weerakit.tay@cmu.ac.th; 6 School of Health Science, Mae Fah Luang University, Chiang Rai 57100, Thailand; 7 Biomedical Technology Research Group for Vulnerable Populations, Mae Fah Luang University, Chiang Rai 57100, Thailand

**Keywords:** *Paris polyphylla*, colorectal cancer, network pharmacology, molecular docking

## Abstract

Colorectal cancer (CRC) remains a major cause of cancer-related morbidity and mortality worldwide, highlighting the need for safer and more effective therapeutic agents. This study investigated the potential anticancer compounds and mechanisms of *Paris polyphylla* against CRC using an integrated approach combining network pharmacology, molecular docking, and in vitro validation. Bioactive compounds were screened from multiple databases, and their putative targets were intersected with CRC-related genes. Protein–protein interaction and enrichment analyses were performed to identify key targets and pathways, followed by the docking of selected compounds with major hub proteins. The cytotoxic and molecular effects of *P. polyphylla* rhizome extract (PPRE) were then evaluated in SW480 and HCT116 cells. A total of 74 compounds were identified, of which 12 were retained for target prediction, yielding 180 overlapping genes between *P. polyphylla* targets and CRC-associated genes. Network analysis highlighted STAT3, EGFR, SRC, IL-6, and AKT1 as key hub targets, with enrichment in cancer-related, EGFR resistance, and PI3K–Akt pathways. Docking showed favorable binding affinities, particularly between prosapogenin A and AKT1. Experimentally, PPRE reduced CRC cell viability and downregulated STAT3, EGFR, SRC, IL-6, and AKT1 expression. These findings suggest that *P. polyphylla* exerts anticancer effects through the coordinated modulation of multiple oncogenic pathways in CRC.

## 1. Introduction

Colorectal cancer (CRC) is one of the most common malignancies worldwide and represents a major global health burden due to its high incidence and mortality. Globally, CRC ranks as the third most frequently diagnosed cancer, accounting for approximately 10% of all cancer cases, and it is the second leading cause of cancer-related deaths [1]. The incidence and mortality of CRC continue to increase, particularly in middle- and low-income countries. By 2030, global incidence is projected to rise by approximately 60%, reaching about 2.2 million new cases and 1.1 million deaths annually [2]. Current treatments primarily involve surgery combined with chemotherapy and radiotherapy; however, these therapies often lack specificity and may damage rapidly dividing normal cells, leading to severe adverse effects. Disease recurrence and metastasis remain major challenges, and more than half of CRC patients are diagnosed at advanced stages, with a five-year survival rate of about 14% [3,4]. Despite advances in diagnostic technologies and the development of targeted therapies and immunotherapy, the prognosis for many CRC patients remains poor due to tumor relapse and therapeutic resistance [5]. Therefore, the development of safer and more effective therapeutic agents is urgently needed to improve clinical outcomes in CRC.

Natural products derived from medicinal plants have gained considerable attention as potential sources of anticancer agents. Experimental and clinical evidence indicates that plant-derived compounds exhibit diverse pharmacological activities and therapeutic benefits in various cancers, including improving quality of life, prolonging survival, reducing treatment-related side effects, and enhancing the efficacy of conventional therapies [6,7,8,9,10]. Owing to their multi-target pharmacological actions and relatively favorable safety profiles, medicinal plants represent valuable resources for the discovery of novel anticancer drugs.

*Paris polyphylla* (Melanthiaceae) is an important medicinal herb widely used in traditional medicine to treat various diseases, including fever, inflammation, and cancer [11,12]. Pharmacological studies have demonstrated that *P. polyphylla* exhibits diverse biological activities, including antibacterial, anti-inflammatory, antioxidant, cardioprotective, immunomodulatory, and anticancer effects [13]. Increasing attention has recently focused on its anticancer potential in CRC [14]. For example, the ethanolic extract of *P. polyphylla* inhibits proliferation of DLD-1 colorectal cancer cells by inducing autophagy and enhancing the efficacy of doxorubicin, with pennogenin 3-O-β-chacotrioside and polyphyllin VI identified as key active compounds [15]. In addition, *P. polyphylla* extract induces apoptosis in HCT116 colorectal cancer cells through increased reactive oxygen species production and activation of caspase-3 signaling, effects mainly attributed to steroidal saponins. The extract has also been reported to enhance the therapeutic efficacy of chemotherapeutic agents such as fluorouracil and cisplatin [16]. Phytochemical studies have identified a wide range of bioactive constituents in *P. polyphylla*, including steroidal saponins, C21 steroidal compounds, flavonoids, phytosterols, polysaccharides, phytoecdysteroids, and amino acids. Among these, steroidal saponins, known as Paris saponins, are the major pharmacologically active components. Several Paris saponins, including polyphyllin I [17,18], polyphyllin II [19], polyphyllin VI [15,20], polyphyllin VII [21,22,23] and polyphyllin IX [24], have shown potent anticancer activity against CRC through multiple mechanisms. However, the complex pharmacological mechanisms underlying the multi-component and multi-target actions of *P. polyphylla* against CRC remain insufficiently understood.

Network pharmacology has emerged as a powerful systems-based approach for elucidating the mechanisms of complex herbal medicines by integrating systems biology, bioinformatics, and pharmacology to analyze interactions among bioactive compounds, molecular targets, and signaling pathways [25]. Molecular docking is also an important tool in structure-based drug discovery, enabling prediction of binding interactions between bioactive molecules and target proteins at the atomic level and providing insights into ligand–receptor binding modes and affinities [26].

Therefore, the present study employed an integrated strategy combining network pharmacology, molecular docking, and in vitro experimental validation to investigate the potential anticancer compounds and mechanisms of *P. polyphylla* against CRC. The findings may provide new insights into the molecular mechanisms underlying the anticancer effects of *P. polyphylla* and support its potential development as a therapeutic candidate for CRC.

## 2. Results

### 2.1. Identification of Bioactive Compounds from Paris polyphylla

Comprehensive database mining identified 74 bioactive compounds from *P. polyphylla*, including 47 from TCMBank, 2 from TCM-ID, 5 from KNApSAcK, and 20 from Dr. Duke’s Phytochemical and Ethnobotanical Databases. After removing duplicates, 54 non-redundant compounds were retained. Of these, 15 compounds yielded reliable ADME-related predictions using SwissADME, while the remaining compounds could not be evaluated due to incomplete structural information or computational limitations.

The predicted ADME properties and drug-likeness profiles of the 15 compounds are summarized in Table 1. Eight compounds including 20-hydroxyecdysone, diosgenin, diosmetin, flavone, pennogenin, pregnane-3,20-diol, protocatechuic acid methyl ester, and spirostanol were predicted to have high GI absorption, whereas seven showed low GI absorption, corresponding to SwissADME bioavailability scores of 0.55 and 0.17, respectively. Consistently, all high-absorption compounds satisfied Lipinski’s rule of five (0–1 violations), while low-absorption compounds exhibited two or more violations. Molsoft drug-likeness scores ranged from −0.74 to 1.37, with 20-hydroxyecdysone showing the highest score.

Overall, several constituents demonstrated pharmacokinetic characteristics compatible with oral bioavailability. Nevertheless, given that major bioactive constituents of *P. polyphylla*, particularly steroidal saponins, have been reported to exhibit potent anticancer activity despite relatively low predicted bioavailability, ADME parameters were used to provide supportive pharmacokinetic context rather than strict exclusion criteria for subsequent network pharmacology and docking analyses.

### 2.2. Identification of Colorectal Cancer-Related Genes and Paris polyphylla Target Genes

A total of 7602 CRC-related genes were retrieved from four databases, including 4283 from NCBI, 2841 from GeneCards, 436 from CTD, and 42 from TTD. After the removal of duplicate entries and verification against UniProtKB to standardize gene annotations, 5237 non-redundant CRC-associated genes were retained (Supplementary Table S1).

Potential molecular targets of *P. polyphylla* compounds were predicted using SwissTargetPrediction (probability > 0.1) and STITCH (confidence score > 0.1). Under these criteria, 12 compounds including diosmetin, spirostanol, 20-hydroxyecdysone, pennogenin, diosgenin, dextrin, pregnane-3,20-diol, flavone, kaempferol 3-gentiobioside-7-rhamnoside, prosapogenin A, diosgenin tetraglycoside, and polyphyllin E (RG) were predicted to interact with potential protein targets. These 12 compounds from *P. polyphylla* were predicted to interact with a total of 271 target genes, which were subsequently used to construct the compound–target network.

### 2.3. Compound–Target Network of Paris polyphylla in Colorectal Cancer

**Figure 1:**
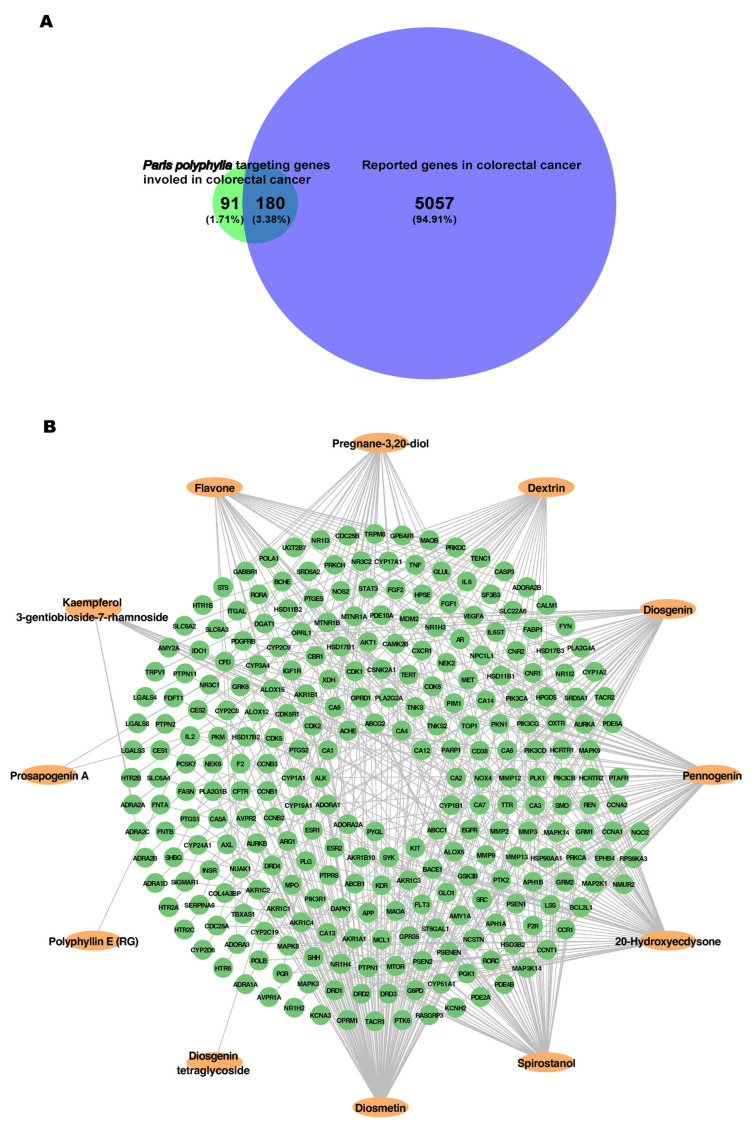
Compound–target network of *Paris polyphylla* in colorectal cancer. (**A**) Venn diagram showing the intersection between predicted *P. polyphylla* target genes (*n* = 271) and colorectal cancer-related genes (*n* = 5237). A total of 180 overlapping genes were identified, while 91 genes were unique to *P. polyphylla* targets and 5057 genes were unique to colorectal cancer-related genes. (**B**) Compound–target interaction network constructed using Cytoscape, showing interactions between *P. polyphylla* bioactive compounds (orange oval nodes) and their associated target genes (green circular nodes). Targets were predicted using SwissTargetPrediction (probability > 0.1) and STITCH (confidence score > 0.1). Edges represent compound–target interactions.

Intersection analysis between the 271 predicted *P. polyphylla* target genes and 5237 curated CRC-related genes identified 180 overlapping genes, whereas 91 genes were unique to *P. polyphylla* targets and 5057 genes were unique to CRC-related genes (Figure 1A). These overlapping genes were considered putative therapeutic targets of *P. polyphylla* against CRC. To further explore compound–target relationships, a compound–target interaction network was constructed using the 12 selected bioactive compounds and their 271 predicted target genes. The resulting network comprised 283 nodes and 449 edges, representing interactions between 12 compounds and 271 associated target genes (Figure 1B).

Topological analysis based on degree centrality revealed differential connectivity among compounds. Diosmetin exhibited the highest degree value, interacting with 107 target genes, followed by spirostanol (64 genes), 20-hydroxyecdysone (60 genes), pennogenin (54 genes), diosgenin (43 genes), dextrin (34 genes), pregnane-3,20-diol (31 genes), flavone (30 genes), and kaempferol 3-gentiobioside-7-rhamnoside (11 genes). Prosapogenin A, diosgenin tetraglycoside, and polyphyllin E (RG) showed limited connectivity, interacting with two, one, and one gene, respectively (Supplementary Table S2).

### 2.4. Protein–Protein Interaction (PPI) Network of Paris polyphylla Targets in Colorectal Cancer

**Figure 2:**
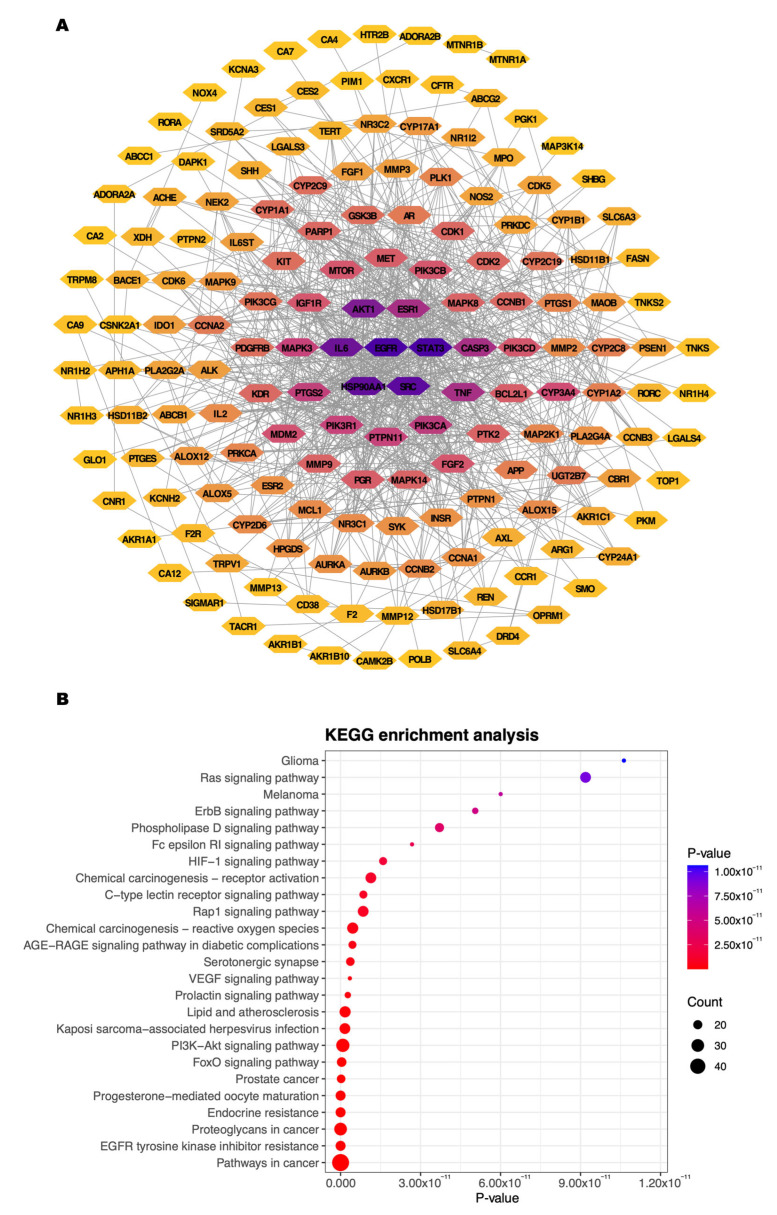
Protein–protein interaction (PPI) network and KEGG pathway enrichment analysis of *Paris polyphylla* target genes in colorectal cancer. (**A**) PPI network constructed using the STRING database and visualized in Cytoscape. Nodes represent proteins and edges indicate known or predicted interactions. Node color intensity corresponds to degree centrality, with darker nodes indicating higher connectivity. (**B**) The top 25 enriched KEGG pathways. The bubble size represents the number of target genes (count), and the color gradient indicates the enrichment *p*-value.

To further investigate the functional interactions among the *P. polyphylla* target genes in CRC, a protein–protein interaction (PPI) network was constructed. Network topological parameters, including degree, betweenness centrality, and closeness centrality, were calculated, and the results are summarized in Supplementary Table S3. The PPI network comprised 166 nodes connected by interaction edges representing known biological associations between proteins (Figure 2A). Topological analysis identified STAT3, EGFR, SRC, HSP90AA1, IL-6, AKT1, CASP3, TNF, ESR1, and PIK3CA as the top 10 hub genes ranked by degree centrality. These highly connected proteins occupied central positions within the network, indicating their potential regulatory roles in CRC-related signaling pathways.

### 2.5. Gene Ontology (GO) and Kyoto Encyclopedia of Genes and Genomes (KEGG) Pathway Enrichment Analysis

GO enrichment analysis revealed significant functional clustering of the target genes across the biological process (BP), cellular component (CC), and molecular function (MF) categories (Table 2). Within the GO BP category, phosphorylation was the most significantly enriched term (45 genes, 24.73%, *p* = 1.39 × 10^−26^), followed by negative regulation of apoptotic process and protein phosphorylation, indicating a strong enrichment of kinase-mediated signaling and apoptosis-related mechanisms. For GO CC, plasma membrane was the predominant enriched term (93 genes, 51.10%, *p* = 4.32 × 10^−12^), indicating that many targets are membrane-associated proteins. In the GO MF category, ATP binding was the most enriched term (52 genes, 28.57%, *p* = 5.80 × 10^−16^), consistent with the enrichment of kinase-related and signaling proteins.

KEGG pathway enrichment analysis further demonstrated significant involvement of cancer-related signaling pathways (Figure 2B). Among the top 25 enriched pathways, pathways in cancer showed the highest gene representation, followed by EGFR tyrosine kinase inhibitor resistance, the PI3K–Akt signaling pathway, the Ras signaling pathway, and the ErbB signaling pathway. These findings suggest that the identified targets are primarily associated with oncogenic signaling cascades relevant to CRC progression.

### 2.6. Compound–Protein–Pathway Network of Paris polyphylla in Colorectal Cancer

**Figure 3:**
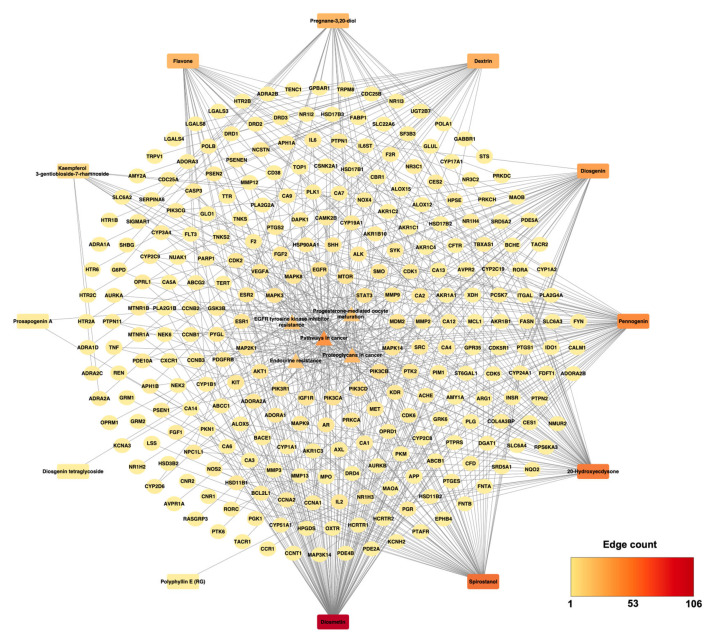
Integrated compound–protein–pathway network of *Paris polyphylla* in colorectal cancer. The network integrates bioactive compounds of *P. polyphylla*, intersecting CRC-associated target genes, and enriched KEGG pathways. Enriched pathways are shown as central triangles, target genes as yellow circular nodes, and bioactive compounds as colored rectangular nodes in the outer layer. Edges represent compound–target and target–pathway associations. Edge color intensity reflects the number of interactions, with red indicating higher connectivity and yellow indicating lower connectivity.

To elucidate the multi-target mechanisms of *P. polyphylla* in CRC, a compound–protein–pathway network was constructed by integrating compound–target interactions, PPI data, and KEGG enrichment results. The integrated network comprised 288 nodes and 581 edges, representing 12 bioactive compounds, intersecting protein targets, and significantly enriched cancer-related pathways (Figure 3; Supplementary Tables S2 and S4).

Among the compounds, diosmetin demonstrated the highest degree centrality, interacting with 107 CRC-associated targets, indicating its prominent role within the network. At the pathway level, Pathways in cancer (hsa05200) showed the greatest connectivity (49 genes), followed by EGFR tyrosine kinase inhibitor resistance (hsa01521), underscoring the enrichment of key oncogenic signaling cascades. Importantly, several high-degree PPI hub genes including STAT3, EGFR, SRC, IL-6, and AKT1 were centrally positioned in the integrated network and participated in both major enriched pathways. Their combined topological prominence and pathway involvement suggest that these proteins function as critical regulatory nodes linking *P. polyphylla* bioactive compounds to CRC-related signaling mechanisms. Accordingly, STAT3, EGFR, SRC, IL-6, and AKT1 were prioritized for subsequent molecular docking to further investigate their mechanistic interactions with active compounds.

### 2.7. Molecular Docking Analysis of Paris polyphylla Compounds Interacting with Colorectal Cancer-Related Proteins

Twelve bioactive compounds, including 20-hydroxyecdysone, dextrin, diosgenin, diosgenin tetraglycoside, diosmetin, flavone, kaempferol 3-gentiobioside-7-rhamnoside, pennogenin, polyphyllin E (RG), pregnane-3,20-diol, prosapogenin A, and spirostanol, were subjected to molecular docking analysis against five key hub proteins identified from network analysis: STAT3 (PDB ID: 6NJS), EGFR (PDB ID: 1M17), SRC (PDB ID: 1O41), IL-6 (PDB ID: 1ALU), and AKT1 (PDB ID: 3O96).

Molecular docking results with the lowest binding energy, interaction residues and bond distances are summarized in Table 3. The lowest binding energy (most negative value) for each compound–protein pair was selected for analysis. All compound–protein interactions exhibited negative binding energies, indicating thermodynamically favorable binding. Among the five targets, AKT1 showed the strongest overall binding affinities with several *P. polyphylla* bioactive compounds, followed by EGFR, STAT3, SRC, and IL-6. Representative docking conformations of the top-ranked ligand–protein complexes are shown in Figure 4A–E, whereas detailed docking interaction diagrams of STAT3, EGFR, SRC, IL-6, and AKT1 with *P. polyphylla* bioactive compounds are provided in Supplementary Figures S1–S5, respectively. Moreover, two-dimensional molecular docking interaction diagrams for all ligand–protein pairs are provided in the Supplementary Figures S6–S54.

**Figure 4:**
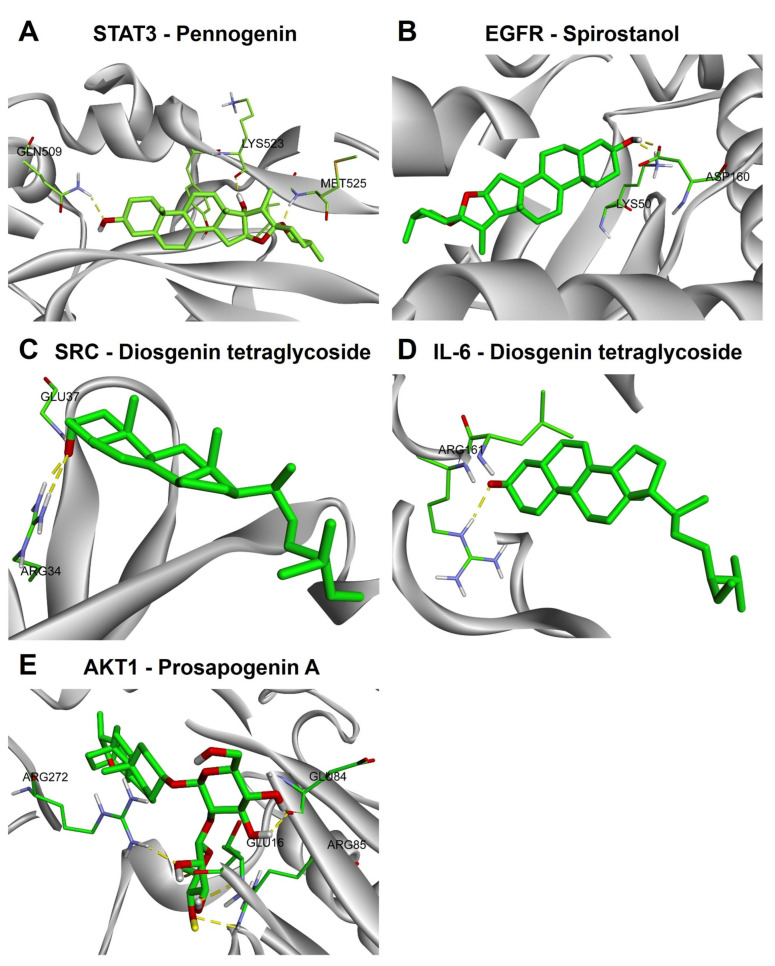
Representative molecular docking conformations of major *Paris polyphylla* bioactive compounds with five key colorectal cancer-related hub proteins: (**A**) STAT3–pennogenin, (**B**) EGFR–spirostanol, (**C**) SRC–diosgenin tetraglycoside, (**D**) IL-6–diosgenin tetraglycoside, and (**E**) AKT1–prosapogenin A. Carbon atoms are shown in green, oxygen in red, nitrogen in purple, sulfur in orange, and hydrogen in white. Yellow dashed lines indicate hydrogen–bond interactions.

For STAT3, pennogenin showed the strongest binding affinity with a docking score of −6.40 kcal/mol, followed by diosgenin tetraglycoside (−6.01 kcal/mol) and diosgenin (−5.98 kcal/mol). The major hydrogen–bond interactions involved MET525, LYS523, GLN509, and GLU503, indicating that these residues may contribute to ligand stabilization within the STAT3 binding pocket (Figure 4A). For EGFR, spirostanol exhibited the most favorable interaction (−7.40 kcal/mol), followed closely by diosgenin (−7.21 kcal/mol) and pennogenin (−6.66 kcal/mol). Key binding residues included ASP160, LYS50, GLU67, and ARG146, supporting stable ligand accommodation within the EGFR active site (Figure 4B).

Moderate binding affinities were observed for SRC and IL-6. Diosgenin tetraglycoside showed the best docking score against SRC (−5.47 kcal/mol), with hydrogen–bond interactions involving GLU37 and ARG34 (Figure 4C). Similarly, diosgenin tetraglycoside exhibited the strongest interaction with IL-6 (−4.97 kcal/mol), primarily through ARG161, suggesting a possible interaction at the cytokine interface region (Figure 4D). Although the docking scores for SRC and IL-6 were weaker than those observed for AKT1 and EGFR, they remained within a favorable binding range and were consistent with their selection as hub targets in the network analysis.

Among all proteins analyzed, AKT1 demonstrated the strongest binding affinity, with prosapogenin A showing the most favorable docking score (−13.25 kcal/mol), followed by spirostanol (−11.27 kcal/mol), diosgenin (−11.10 kcal/mol), and pennogenin (−10.76 kcal/mol). The principal hydrogen–bond interactions involved GLU84, GLU16, ARG272, ARG85, SER204, and THR210, suggesting that these residues are important for ligand recognition within the AKT1 binding pocket (Figure 4E). Collectively, these results indicate that several *P. polyphylla* compounds exhibit favorable in silico interactions with the five selected CRC-related hub proteins, with particularly strong binding observed for AKT1 and EGFR.

### 2.8. Cytotoxicity of Paris polyphylla Rhizome Extract (PPRE) in Colorectal Cancer Cells

**Figure 5:**
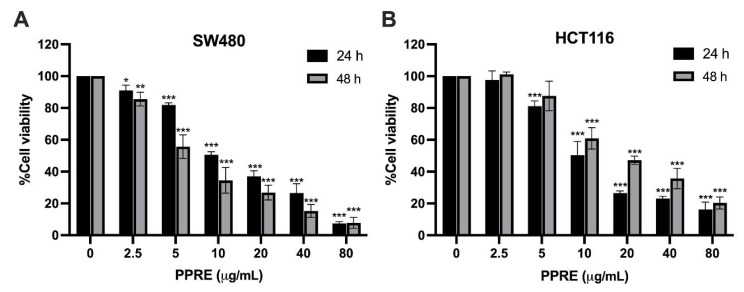
Cytotoxic effects of *Paris polyphylla* rhizome extract (PPRE) on colorectal cancer cells. SW480 (**A**) and HCT116 (**B**) cells were treated with increasing concentrations of PPRE (0–80 µg/mL) for 24 and 48 h. Data are presented as mean ± SD from three independent experiments. Statistical significance compared with the untreated control group was determined using one-way ANOVA followed by Tukey’s multiple comparison test (* *p* < 0.05, ** *p* < 0.01, *** *p* < 0.001).

Extraction of *P. polyphylla* rhizome with 70% ethanol yielded approximately 20% (*w*/*w*). The cytotoxic effects of PPRE were evaluated in SW480 and HCT116 colorectal cancer cells treated with 0–80 µg/mL for 24 and 48 h. As shown in Figure 5, PPRE significantly reduced cell viability in a dose-dependent manner at both time points. In SW480 cells, the IC20 and IC50 values at 24 h were 4.94 ± 0.30 µg/mL and 10.08 ± 1.52 µg/mL, respectively. After 48 h, cytotoxic sensitivity increased, with IC20 and IC50 decreasing to 2.77 ± 0.61 µg/mL and 4.82 ± 0.82 µg/mL. In HCT116 cells, the IC20 and IC50 values at 24 h were 5.25 ± 0.44 µg/mL and 10.28 ± 1.88 µg/mL, respectively. In contrast to SW480, 48 h exposure resulted in slightly higher IC values (IC20 = 6.25 ± 1.38 µg/mL; IC50 = 10.42 ± 4.43 µg/mL), indicating a differential time-dependent response between the two cell lines.

### 2.9. Effects of Paris polyphylla Rhizome Extract (PPRE) on Hub Gene mRNA Expression in Colorectal Cancer Cells

To validate the in silico predictions and molecular docking results, the mRNA expression levels of five hub genes (STAT3, EGFR, SRC, IL-6, and AKT1) were quantified by qRT-PCR following treatment with increasing concentrations of PPRE (0–10 µg/mL) in SW480 and HCT116 cells.

**Figure 6:**
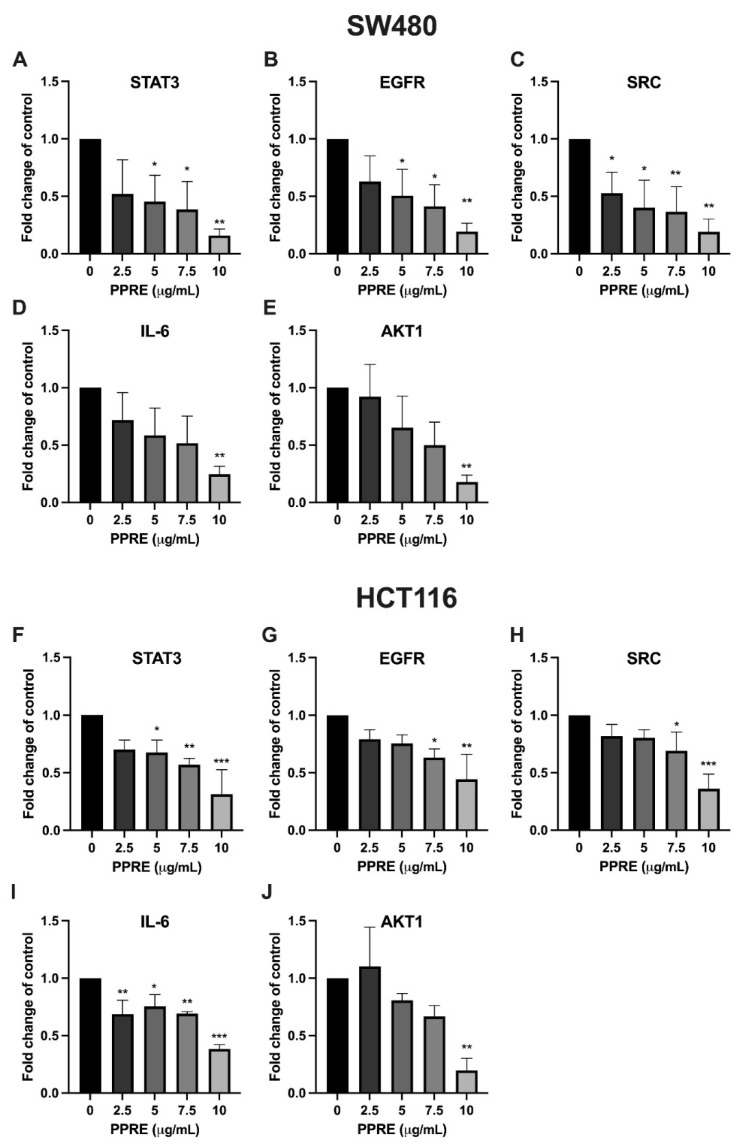
qRT-PCR Validation of STAT3, EGFR, SRC, IL-6, and AKT1 mRNA expression in SW480 (**A**–**E**) and HCT116 (**F**–**J**) cells following treatment with PPRE (0–10 µg/mL). Gene expression levels were normalized to the internal control and expressed as fold change relative to untreated control (0 µg/mL). Data are presented as mean ± SD of three independent experiments. Statistical significance versus the untreated control was determined using one-way ANOVA followed by Tukey’s post hoc test (* *p* < 0.05, ** *p* < 0.01, *** *p* < 0.001).

As shown in Figure 6A–E, PPRE treatment significantly reduced the expression of STAT3, EGFR, SRC, IL-6, and AKT1 in a dose-dependent manner. STAT3 expression was progressively suppressed, with significant reductions observed at 5–10 µg/mL. EGFR and SRC expression levels similarly decreased with increasing PPRE concentrations, showing statistically significant inhibition at ≥5 µg/mL. IL-6 mRNA expression exhibited marked downregulation, particularly at 10 µg/mL. AKT1 expression was also significantly reduced at higher concentrations, consistent with the predicted central role of the PI3K/AKT pathway.

In HCT116 cells (Figure 6F–J), PPRE treatment also reduced the expression of the selected hub genes, although the magnitude of suppression was comparatively less pronounced than in SW480 cells. STAT3 expression decreased significantly at higher concentrations (≥7.5 µg/mL). EGFR and SRC mRNA levels showed moderate but significant reductions. IL-6 expression was significantly suppressed at 10 µg/mL, while AKT1 exhibited a dose-dependent reduction, reaching statistical significance at the highest concentration tested. These findings confirm that PPRE modulates key oncogenic signaling pathways in colorectal cancer cells and support the network pharmacology and docking predictions identifying STAT3, EGFR, SRC, IL-6, and AKT1 as major functional targets.

## 3. Discussion

The present study applied an integrated strategy combining network pharmacology, molecular docking, and in vitro validation to investigate the potential anticancer mechanisms of *P. polyphylla* against CRC. The results revealed a multi-component and multi-target pharmacological profile in which several bioactive compounds were predicted to interact with key oncogenic proteins involved in CRC progression. Network analysis identified 180 overlapping genes between predicted targets of *P. polyphylla* compounds and CRC-related genes, with top 10 hub proteins including STAT3, EGFR, SRC, HSP90AA1, IL-6, AKT1, CASP3, TNF, ESR1, and PIK3CA. Pathway enrichment further highlighted several cancer-associated pathways, particularly pathways in cancer, EGFR tyrosine kinase inhibitor resistance, PI3K–Akt signaling, Ras signaling, and ErbB signaling pathways. These results suggest that *P. polyphylla* may exert anticancer activity through coordinated regulation of multiple oncogenic signaling networks rather than through a single molecular target.

Database-assisted phytochemical compound screening and ADME analysis identified several potentially active compounds in *P. polyphylla*, which can be broadly classified into three major groups: (i) steroidal saponins and steroid derivatives (e.g., diosgenin, pennogenin, and polyphyllin E), (ii) flavonoids (e.g., diosmetin, flavone, and kaempferol derivatives), and (iii) other constituents, including phytoecdysteroids and polysaccharides (e.g., 20-hydroxyecdysone and dextrin). Among these, steroidal saponins represent the predominant pharmacologically active class and have been widely reported to contribute to the anticancer activity of *P. polyphylla*. Although several steroidal saponins exhibited relatively low predicted gastrointestinal absorption, they are recognized as key bioactive constituents of this medicinal plant [27,28]. Steroidal saponins from medicinal plants have been extensively reported to exert anticancer effects through multiple mechanisms, including induction of apoptosis, cell-cycle arrest, and inhibition of oncogenic signaling pathways [29,30,31]. In addition, herbal medicines often exert therapeutic effects through synergistic interactions among multiple compounds. Therefore, strict exclusion of compounds based solely on classical drug-likeness criteria may underestimate the pharmacological relevance of structurally complex phytochemicals [32,33]. Therefore, retaining compounds with biological relevance rather than applying overly restrictive ADME filters may provide a more realistic representation of herbal pharmacology. The observed cytotoxic effects of PPRE are likely attributable to the combined effects of multiple phytochemicals, particularly steroidal saponins reported as major constituents of *P. polyphylla* rhizomes. Nevertheless, future quantitative LC-MS/MS studies are warranted to determine the abundance of individual metabolites and correlate compound levels with biological activity.

The compound–target network analysis suggested that several compounds may function as key regulators within the pharmacological system of *P. polyphylla*. Among these, diosmetin exhibited the highest network connectivity, indicating its potential to interact with multiple protein targets simultaneously. Flavonoids such as diosmetin have previously been reported to exhibit anticancer properties through modulation of oxidative stress, inhibition of tumor cell proliferation, and regulation of signaling pathways including PI3K–Akt and MAPK pathways [34,35]. Similarly, steroidal saponins such as pennogenin and diosgenin derivatives have been shown to induce apoptosis and suppress tumor growth in various cancer models, including colorectal cancer [36,37]. The high network centrality observed for these compounds therefore supports their potential role as important pharmacological contributors to the anticancer activity of *P. polyphylla*.

Protein–protein interaction network analysis identified several hub genes involved in CRC pathogenesis, including STAT3, EGFR, SRC, IL-6, and AKT1. Persistent activation of STAT3 is widely recognized as a key driver of tumor proliferation, inflammation, and immune evasion in colorectal cancer [38]. Similarly, EGFR signaling plays a critical role in CRC progression by regulating cell proliferation, survival, and angiogenesis, and it represents an established therapeutic target in CRC treatment [39,40]. SRC kinase acts as a central signaling mediator linking multiple receptor tyrosine kinases and intracellular pathways involved in tumor invasion and metastasis [41]. In addition, IL-6-mediated inflammatory signaling has been shown to promote tumor progression and STAT3 activation in colorectal cancer [42]. AKT1, a core component of the PI3K–Akt signaling pathway, plays an essential role in regulating cell survival, proliferation, and metabolic reprogramming in cancer cells [43]. The identification of these hub genes suggests that *P. polyphylla* may exert its anticancer activity by simultaneously targeting multiple interconnected signaling pathways involved in tumor development and progression.

Gene ontology and KEGG enrichment analyses further supported this hypothesis by revealing significant enrichment in pathways associated with protein phosphorylation, kinase activity, and membrane-associated signaling processes. Many predicted targets were involved in oncogenic signaling pathways such as PI3K–Akt, Ras, and ErbB signaling pathways. These pathways are known to regulate tumor cell proliferation, survival, migration, and therapeutic resistance in colorectal cancer [44,45,46]. The enrichment of EGFR tyrosine kinase inhibitor resistance pathways further suggests that *P. polyphylla* may influence mechanisms associated with drug resistance, which remains a major challenge in CRC therapy [47].

The integrated compound–protein–pathway network provided a systems-level view of the potential pharmacological interactions of *P. polyphylla*. Within this network, several compounds were linked to multiple hub genes and enriched pathways, illustrating the complex molecular interactions underlying the pharmacological activity of the herbal extract. Such network-based interactions support the concept of polypharmacology, in which therapeutic effects arise from the coordinated modulation of multiple targets and pathways rather than single-target inhibition [48,49]. This systems-level perspective is particularly relevant for herbal medicines, which typically contain numerous bioactive constituents acting synergistically.

Molecular docking analysis further supported the predicted interactions between the selected compounds and key hub proteins. Several compounds exhibited favorable binding affinities with AKT1, EGFR, STAT3, SRC, and IL-6. Among these interactions, prosapogenin A showed the strongest binding affinity with AKT1, suggesting potential modulation of the PI3K–Akt signaling pathway. Natural compounds targeting the PI3K–Akt pathway have attracted considerable interest because this pathway plays a central role in cancer cell survival and resistance to therapy [50,51]. Additional compounds such as spirostanol derivatives and diosgenin also demonstrated favorable interactions with EGFR and AKT1 [52,53,54], supporting the hypothesis that multiple phytochemicals from *P. polyphylla* may cooperatively regulate key oncogenic pathways. Nevertheless, molecular docking provides only theoretical predictions of ligand–protein interactions, and further biochemical studies are required to confirm direct target engagement. Future computational studies should incorporate molecular dynamics simulations to evaluate the conformational stability and dynamic interactions of key ligand–protein complexes identified in the present study.

Experimental validation using cytotoxicity assays demonstrated that the ethanolic extract of *P. polyphylla* inhibited the viability of SW480 and HCT116 colorectal cancer cells in a dose-dependent manner. Differences in sensitivity between the two cell lines were observed, which may reflect variations in genetic background, signaling pathway activation, or metabolic characteristics between CRC subtypes [55]. Such heterogeneity is commonly observed in colorectal cancer and may influence therapeutic responses.

Quantitative real-time PCR analysis further demonstrated that treatment with *P. polyphylla* extract significantly reduced the expression of STAT3, EGFR, SRC, IL-6, and AKT1 in both SW480 and HCT116 cells. These findings provide experimental evidence supporting the computational predictions generated by the network pharmacology analysis. The coordinated downregulation of these genes suggests that *P. polyphylla* may suppress multiple interconnected signaling pathways involved in tumor proliferation, inflammation, and survival. Multi-target modulation of oncogenic signaling networks has increasingly been recognized as a promising strategy for cancer therapy, particularly in complex diseases such as colorectal cancer [56].

Several limitations of this study should be acknowledged. First, the target prediction and network analysis relied on publicly available databases, which may introduce prediction bias or incomplete target coverage. Second, molecular docking provides only computational predictions of ligand–protein interactions and does not confirm direct biochemical binding. Third, experimental validation focused on gene expression analysis, and additional studies examining protein expression, phosphorylation status, and downstream signaling pathways would provide more comprehensive mechanistic insights. Fourth, the phytochemical composition of the tested PPRE was not experimentally characterized by LC-MS/MS or related analytical techniques. Therefore, the predicted compounds were derived from published phytochemical databases, and future studies should perform direct metabolite profiling to confirm the presence and abundance of active constituents in the extract. Furthermore, the in vitro experiments were conducted using crude plant extract, and the specific contributions of individual compounds remain to be clarified. In addition, future in vitro studies should include standard chemotherapeutic agents such as 5-fluorouracil or irinotecan as positive controls to enable direct comparison of anticancer potency.

In conclusion, the present study provides a comprehensive systems-level investigation of the anticancer mechanisms of *P. polyphylla* against colorectal cancer. The integrated analysis identified multiple bioactive compounds, key molecular targets, and relevant signaling pathways involved in the anticancer activity of this medicinal plant. The findings suggest that *P. polyphylla* exerts its anticancer effects through the coordinated regulation of multiple oncogenic pathways, particularly those involving STAT3, EGFR, SRC, IL-6, and AKT1. These results provide new insights into the molecular mechanisms underlying the pharmacological activity of *P. polyphylla* and support its potential development as a source of therapeutic agents for colorectal cancer.

## 4. Materials and Methods

An overview of the network pharmacology workflow of *Paris polyphylla* in relation to CRC is presented in Supplementary Figure S55.

### 4.1. Screening of Bioactive Compounds from Paris polyphylla and ADME Evaluation

Bioactive compounds of *P. polyphylla* were retrieved from TCMBank (https://www.tcmbank.cn (accessed on 16 January 2025)), TCM-ID (https://bidd.group/TCMID/ (accessed on 16 January 2025)), KNApSAcK (https://www.knapsackfamily.com/KNApSAcK/ (accessed on 16 January 2025)), and Dr. Duke’s Phytochemical and Ethnobotanical Databases (https://phytochem.nal.usda.gov (accessed on 16 January 2025)). Duplicate entries were removed to obtain a non-redundant compound dataset. Molecular structural information, including canonical Simplified Molecular Input Line Entry System (SMILES) strings and structured data files (SDFs), was obtained from the PubChem (https://pubchem.ncbi.nlm.nih.gov (accessed on 16 January 2025)) and ZINC (https://zinc.docking.org (accessed on 16 January 2025)) databases. Only compounds with valid canonical SMILES were included for subsequent in silico pharmacokinetic evaluation.

ADME-related properties were predicted using SwissADME (http://www.swissadme.ch (accessed on 16 January 2025)), including gastrointestinal (GI) absorption and bioavailability score, which qualitatively estimates the likelihood of oral absorption based on molecular properties. Drug-likeness was evaluated according to Lipinski’s rule of five and the number of rule violations generated by SwissADME. Quantitative drug-likeness scores were further assessed using the Molsoft molecular property tool (https://molsoft.com/mprop/ (accessed on 16 January 2025)). ADME characteristics were used to describe pharmacokinetic properties and were not applied as strict exclusion criteria in compound selection.

### 4.2. Target Prediction of Paris polyphylla Compounds

Putative targets of the screened compounds were predicted using SwissTargetPrediction (http://www.swisstargetprediction.ch (accessed on 16 January 2025)) and STITCH (http://stitch.embl.de (accessed on 16 January 2025)), restricting the species to *Homo sapiens.* Targets with probability values > 0.1 in SwissTargetPrediction and confidence scores > 0.1 in STITCH were retained. All predicted targets were merged, duplicate entries were removed, and gene symbols were standardized using the UniProtKB database (https://www.uniprot.org/ (accessed on 16 January 2025)), with the organism specified as *Homo sapiens*.

### 4.3. Identification of Colorectal Cancer-Related Genes

CRC-associated genes were retrieved from four databases: NCBI Gene (https://www.ncbi.nlm.nih.gov/gene (accessed on 16 January 2025)), GeneCards (https://www.genecards.org (accessed on 16 January 2025)), the Comparative Toxicogenomics Database (CTD; http://ctdbase.org (accessed on 16 January 2025)), and the Therapeutic Target Database (TTD; https://ttd.idrblab.cn/ (accessed on 16 January 2025)). Searches were conducted using the keyword “colorectal cancer”. All retrieved genes were combined into a single dataset, and duplicate entries were removed. Gene names and symbols were standardized and verified using UniProtKB, restricting the organism to *Homo sapiens*, to obtain a non-redundant list of CRC-related genes.

### 4.4. Construction of Compound–Target and Protein–Protein Interaction (PPI) Networks

The overlapping target genes between *P. polyphylla*-predicted targets and CRC-related genes were identified and visualized by a Venn diagram using BioVenn tool (https://www.biovenn.nl (accessed on 18 January 2025)). The intersecting gene set was imported into the STRING database (https://string-db.org/ (accessed on 16 January 2025)) with the species restricted to *Homo sapiens* and a high-confidence interaction score of at least 0.7. Disconnected nodes were excluded from the analysis to enhance network reliability. Network visualization and topological analysis, including calculation of degree, betweenness centrality, and closeness centrality, were performed using Cytoscape version 3.10.3. Hub genes were identified based on degree centrality ranking. An integrated compound–protein–pathway network was subsequently constructed by combining compound–target interactions, PPI results, and KEGG pathway enrichment data.

### 4.5. Gene Ontology and KEGG Pathway Enrichment Analysis

Functional enrichment analysis was conducted using the Database for Annotation, Visualization and Integrated Discovery (DAVID). Gene Ontology (GO) enrichment was performed across biological process, cellular component, and molecular function categories. Kyoto Encyclopedia of Genes and Genomes (KEGG) pathway enrichment was performed using the modified Fisher’s exact test. Pathways and GO terms with *p*-values less than or equal to 0.05 were considered statistically significant. The top 25 KEGG pathways were ranked according to gene count. Visualization of enrichment results was performed using the ggplot2 package in RStudio version 2024.12.0, build 467.

### 4.6. Molecular Docking Analysis

Ligand structures of selected bioactive compounds were downloaded from the PubChem database and prepared by adding hydrogen atoms followed by geometry optimization using Discovery Studio Visualizer v21.1.0.20298. Crystal structures of target proteins associated with CRC were retrieved from the Protein Data Bank (PDB) database (http://www.rcsb.org/ (accessed on 16 January 2025)). Where required, missing loop regions were refined using the MODELLER web server integrated with UCSF Chimera version 1.14, and the optimal model was selected based on the lowest DOPE-HR score.

Prior to docking, water molecules were removed from protein structures, and protonation states at physiological pH (7.4) were assigned using the PDB2PQR server (https://server.poissonboltzmann.org/pdb2pqr (accessed on 31 January 2025)). Molecular docking simulations were performed using AutoDock 4.2 with the Lamarckian Genetic Algorithm. The native co-crystallized ligand of each protein was used to define the docking grid box and active binding region. For all protein targets, the grid box dimensions were set to 60 × 60 × 60 Å with a grid spacing of 0.375 Å. Grid centers were defined as follows: STAT3 (x = 16.100, y = 54.250, z = 4.802), EGFR (x = 22.265, y = 5.740, z = 59.686), SRC (x = 14.735, y = 23.855, z = 26.031), IL-6 (x = −8.172, y = −12.954, z = 2.725), and AKT1 (x = 11.743, y = −8.682, z = 10.222). One hundred docking runs were performed for each ligand–protein complex. Docking conformations and intermolecular interactions were visualized using Discovery Studio Visualizer v21.1.0.20298.

### 4.7. Plant Collection and Extraction

*P. polyphylla* rhizomes were cultivated and harvested at Ban Ton Plong Tai Community Forest, Bunrueang Sub-District, Chiang Khong District, Chiang Rai Province, Thailand. The plant material was authenticated by the Queen Sirikit Botanic Garden Herbarium (QBG), Chiang Mai, Thailand, and a voucher specimen (QBG No. 149137) was deposited. The rhizomes were dried at 50 °C, pulverized, and extracted with 70% ethanol at a ratio of 100 g per liter by shaking overnight at room temperature. The extract was filtered, evaporated under reduced pressure at 50 °C, and lyophilized to obtain crude ethanolic *P. polyphylla* rhizome extract (PPRE). The PPRE was stored at −20 °C until use for further studies.

### 4.8. Cytotoxicity Assay in Human Colorectal Cancer Cell Lines

The in vitro experiments were approved by the Institutional Biosafety Committee of the Faculty of Medicine, Chiang Mai University (No. CMUIBC02024/2568). Human colorectal cancer cell lines SW480 and HCT116 (ATCC, Manassas, VA, USA) were cultured in Dulbecco’s Modified Eagle Medium (DMEM) supplemented with 10% fetal bovine serum, 100 U/mL penicillin, and 100 µg/mL streptomycin. Cells were maintained at 37 °C in a humidified incubator containing 5% CO_2_.

For the cytotoxicity assay, SW480 and HCT116 cells were seeded at a density of 2 × 10^3^ cells per well in 96-well plates and allowed to attach for 24 h before treatment. Cells were then treated with increasing concentrations of PPRE ranging from 0 to 80 µg/mL for 24 and 48 h. Cell viability was assessed using the MTT assay. Briefly, MTT solution was added and incubated for 4 h at 37 °C. The resulting formazan crystals were dissolved in dimethyl sulfoxide (DMSO), and absorbance was measured at 540/630 nm using a Synergy™ HT Multi-Detection Microplate Reader (BioTek Instruments, Winooski, VT, USA). IC20 and IC50 values were calculated from three independent experiments. Non-cytotoxic concentrations not exceeding the IC20 value were selected for subsequent gene expression analyses.

### 4.9. RNA Extraction, cDNA Synthesis, and Quantitative Real-Time PCR Analysis

SW480 and HCT116 cells were seeded at a density of 1 × 10^5^ cells per well in 6-well plates and treated with PPRE at non-cytotoxic concentrations not exceeding IC20 (0–10 µg/mL) for 24 h. Following treatment, total RNA was extracted using TRIzol reagent according to the manufacturer’s instructions. The concentration and purity of RNA were determined prior to reverse transcription. Complementary DNA (cDNA) was synthesized from 1 µg of total RNA using the ReverTra Ace^®^ qPCR RT Kit (TOYOBO Co., Ltd., Osaka, Japan) following the manufacturer’s protocol.

Quantitative real-time PCR (qRT-PCR) was performed using the SensiFAST™ SYBR^®^ Lo-ROX Kit (Bioline, London, UK) on an Applied Biosystems 7500 Real-Time PCR System (Applied Biosystems, Foster City, CA, USA). GAPDH was used as the internal reference gene. The primer sequences used in this study were as follows: STAT3 forward 5′-CTGTGGGAAGAATCACGCCT-3′ and reverse 5′-ACATCCTGAAGGTGCTGCTC-3′; EGFR forward 5′-AGATCAAAGTGCTGGGCTCC-3′ and reverse 5′-TGGCTTTCGGAGATGTTGCT-3′; SRC forward 5′-ATCACCAGACGGGAGTCAGA-3′ and reverse 5′-CAGTAGGCACCTTTCGTGGT-3′; IL-6 forward 5′-CATCCCATAGCCCAGAGCAT-3′ and reverse 5′-TGGGTCAGGGGTGGTTATTG-3′; AKT1 forward 5′-CAGGATGTGGACCAACGTGA-3′ and reverse 5′-AAGGTGCGTTCGATGACAGT-3′; and GAPDH forward 5′-ACCACAGTCCATGCCATCAC-3′ and reverse 5′-TCCACCACCCTGTTGCTGTA-3′. The PCR amplification program consisted of an initial denaturation at 95 °C for 10 min, followed by 40 cycles of denaturation at 95 °C for 15 s and annealing/extension at 60 °C for 60 s. Relative gene expression levels were normalized to GAPDH and calculated using the 2^−∆∆Ct^ method.

### 4.10. Statistical Analysis

All experiments were performed in triplicate, and data are presented as mean ± standard deviation. Statistical analysis for cytotoxicity assay and qRT-PCR analysis was conducted using GraphPad Prism version 6.0. Differences among groups were analyzed using one-way analysis of variance followed by Tukey’s post hoc test. A *p*-value less than 0.05 was considered statistically significant.

## Figures and Tables

**Table 1:** ADME-related properties and drug-likeness of selected bioactive compounds from *Paris polyphylla*.

No.	Compound	GI Absorption	Bioavailability Score (SwissADME)	Drug-Likeness (SwissADME; Lipinski Violations)	Drug-Likeness Score (Molsoft)
1	20-Hydroxyecdysone	High	0.55	Yes; 1 violation	1.37
2	Chonglou saponin II	Low	0.17	No; 3 violations	0.24
3	Dextrin	Low	0.17	No; 3 violations	0.11
4	Diosgenin	High	0.55	Yes; 1 violation	−0.09
5	Diosgenin palmitate	Low	0.17	No; 2 violations	0.44
6	Diosgenin tetraglycoside	Low	0.17	No; 3 violations	0.24
7	Diosmetin	High	0.55	Yes; 0 violation	0.06
8	Flavone	High	0.55	Yes; 0 violation	0.04
9	Kaempferol 3-gentiobioside-7-rhamnoside	Low	0.17	No; 3 violations	0.75
10	Pennogenin	High	0.55	Yes; 0 violation	0.68
11	Polyphyllin E (RG)	Low	0.17	No; 3 violations	0.24
12	Pregnane-3,20-diol	High	0.55	Yes; 1 violation	−0.29
13	Prosapogenin A	Low	0.17	No; 3 violations	0.24
14	Protocatechuic acid, methyl ester	High	0.55	Yes; 0 violation	0.01
15	Spirostanol	High	0.55	Yes; 1 violation	−0.74

Abbreviations: GI, gastrointestinal. ADME properties were predicted using the SwissADME web tool. Bioavailability score represents the probability of oral bioavailability based on molecular properties. Drug-likeness (Lipinski violations) indicates compliance with Lipinski’s rule of five, where “Yes” denotes compounds with ≤1 violation. Drug-likeness scores were calculated using the Molsoft tool, where higher values indicate better drug-like properties.

**Table 2:** Top five enriched Gene Ontology (GO) terms across the biological process (BP), cellular component (CC), and molecular function (MF) categories.

GO Type	Term	Count	%	*p*-Value
GO BP	Phosphorylation	45	24.73	1.39 × 10^−26^
	Negative regulation of apoptotic process	36	19.78	4.83 × 10^−21^
	Protein phosphorylation	29	15.93	8.32 × 10^−18^
	Protein autophosphorylation	20	10.98	1.09 × 10^−16^
	Xenobiotic metabolic process	18	9.89	8.99 × 10^−16^
GO CC	Plasma membrane	93	51.10	4.32 × 10^−12^
	Cytoplasm	88	48.35	3.04 × 10^−9^
	Cytosol	87	47.80	2.89 × 10^−9^
	Receptor complex	18	9.89	1.26 × 10^−11^
	Membrane raft	17	9.34	4.92 × 10^−11^
GO MF	ATP binding	52	28.57	5.80 × 10^−16^
	Protein serine kinase activity	28	15.38	8.41 × 10^−17^
	Protein serine/threonine kinase activity	28	15.38	2.44 × 10^−16^
	Enzyme binding	27	14.84	1.30 × 10^−15^
	Nuclear receptor activity	14	7.69	2.27 × 10^−15^

Abbreviations: BP, biological process; CC, cellular component; MF, molecular function. Count indicates the number of genes enriched in each GO term; % represents the proportion of genes relative to the total number of target genes. *p*-values were calculated using the DAVID functional annotation tool, and terms with *p* ≤ 0.05 were considered statistically significant.

**Table 3:** Molecular docking interactions between bioactive compounds of *Paris polyphylla* and five key colorectal cancer-related hub proteins identified by network analysis.

Protein	Compound	Lowest Binding Energy (kcal/mol)	Conventional H-Bond Interaction Residues	Bond Distance (Å)
STAT3	Pennogenin	−6.4	MET525	1.68
			LYS523	2.00
			GLN509	2.20
	Diosgenin tetraglycoside	−6.01	GLU503	2.06
	Diosgenin	−5.98	GLN509	2.31
			GLU503	2.94
			MET525	5.33
	Prosapogenin A	−5.74	GLN509 (×2)	2.05, 2.12
	Pregnane-3,20-diol	−5.54	LYS523	2.11
			ASN512	2.12
			GLN509	2.27
	Spirostanol	−5.38	GLN509	2.03
			MET525	2.16
	Diosmetin	−4.45	SER501	1.88
			TYR522	2.12
			GLN509 (×2)	2.25, 2.36
	Flavone	−4.32	TYR505	1.85
	20-Hydroxyecdysone	−4.02	LYS523	1.57
			GLU503 (×2)	2.02, 3.03
			GLY521 (×2)	2.05, 2.34
			TYR505	2.25
	Kaempferol 3-gentiobioside-7-rhamnoside	−2.21	GLY521	1.78
			ASN512 (×2)	1.86, 5.25
			GLN509 (×2)	1.98, 2.00
	Dextrin	−1.69	LYS523	1.91
			GLU503	1.96
	Polyphyllin E (RG)	−0.67	GLN509 (×2)	1.65, 1.99
			GLN503	2.22
EGFR	Spirostanol	−7.40	ASP160	1.90
			LYS50	1.96
	Diosgenin	−7.21	ASP160	1.93
			LYS50	1.97
	Pennogenin	−6.66	GLU67	1.66
	Pregnane-3,20-diol	−6.36	ARG146	1.87
			LEU93	2.21
			ALA48	2.46
	Prosapogenin A	−6.34	ARG146 (×2)	1.60, 1.81
			ASP160	1.74
			ASN147	2.97
	Diosmetin	−5.28	MET98	1.92
			ASP160	2.08
			GLN96	2.52
	Flavone	−4.90	MET98	1.67
	20-Hydroxyecdysone	−4.59	ASP160	1.83
			PRO99 (×2)	1.96, 2.24
			CYS102	2.99
	Kaempferol 3-gentiobioside-7-rhamnoside	−2.53	ARG146	1.74
			ASP160	1.84
			CYS102	2.23
			MET98	2.28
			GLN96	2.76
	Dextrin	−2.17	PRO99 (×2)	2.10, 2.13
			ASP160	2.26
			CYS102	2.47
			GLY101	2.47
			MET98	2.29
SRC	Diosgenin tetraglycoside	−5.47	GLU37	1.95
			ARG34	4.83
	Spirostanol	−5.41	LYS59	2.11
			LYS62	2.62
	Pennogenin	−5.23	HIS60	1.73
			ILE73	1.75
	20-Hydroxyecdysone	−5.08	LYS59	1.87
			HIS60 (×3)	1.89, 2.22, 2.42
			VAL58	2.18
	Diosgenin	−4.92	LYS62	2.95
	Pregnane-3,20-diol	−4.54	HIS60	2.05
			GLU37	2.09
			LYS62	2.13
	Prosapogenin A	−3.90	LYS62	1.89
	Flavone	−3.74	LYS62	1.71
	Diosmetin	−3.63	GLY95	1.99
	Kaempferol 3-gentiobioside-7-rhamnoside	−2.52	LYS59 (×2)	1.91, 2.00
			ARG14	2.28
IL-6	Diosgenin tetraglycoside	−4.97	ARG161	2.16
	Spirostanol	−4.34	ASP16	1.97
			ARG161	4.48
	Diosgenin	−4.29	ASP16	1.98
			ARG161 (×2)	3.65, 3.98
	Pregnane-3,20-diol	−4.29	ARG12	2.29
			ARG161	2.90
	Diosmetin	−4.11	GLN157	1.94
			ARG161	1.96
	20-Hydroxyecdysone	−3.46	ASP16 (×2)	2.04, 2.14
	Prosapogenin A	−3.40	GLN157	1.95
AKT1	Prosapogenin A	−13.25	GLU84 (×2)	1.98, 2.15
			GLU16 (×2)	1.98, 2.19
			ARG272	2.01
			ARG85	2.82
	Spirostanol	−11.27	SER204	1.90
	Diosgenin	−11.10	SER204	1.85
			ASN53	4.60
	Pennogenin	−10.76	SER204	2.06
			THR81	2.39
	20-Hydroxyecdysone	−10.36	TYR271 (×2)	1.83, 2.82
			THR210 (×2)	1.84, 2.04
			GLN78	2.11
			ASP273 (×2)	2.31, 2.50
	Pregnane-3,20-diol	−9.81	VAL270	1.71
			THR210	2.04
	Diosmetin	−8.28	SER204	2.19
			LYS267	2.24
			THR210	2.50
	Flavone	−7.75	LYS267	2.15
	Kaempferol 3-gentiobioside-7-rhamnoside	−7.60	ASP273 (×2)	1.90, 2.30
			GLN58	2.06
			SER204	2.07
			GLU113	2.11
			THR81	2.26
			ASN198	2.51
			GLN78	2.60
			ASP291	2.88
	Dextrin	−5.16	THR210	1.82
			ASP273	2.21
			TYR271	2.80
			ARG272	2.92
			TRP79	2.98

Binding energy values are expressed in kcal/mol. Lower (more negative) binding energy indicates stronger binding affinity between the ligand and target protein. Interaction residues represent amino acid residues involved in conventional hydrogen bonding. Bond distances are presented in angstroms (Å).

## Data Availability

The original contributions presented in this study are included in the article/Supplementary Material. Further inquiries can be directed to the corresponding author.

## Supplementary Materials

The following supporting information can be downloaded at https://www.mdpi.com/article/10.3390/ijms27093874/s1.
