# Supplementary material for: Network Pharmacology and Molecular Docking-Based Approach Revealing the Potential Anticancer Compounds and Molecular Mechanisms of Paris polyphylla Against Colorectal Cancer
Source: Int J Mol Sci. 2026 Apr 27;27(9):3874. doi: 10.3390/ijms27093874 (PMC13163468; doi:10.3390/ijms27093874)
Supplement: Supplementary file 1 [file ijms-27-03874-s001.zip › ijms-4261289-supplementary.pdf]

Supplementary Material

Supplementary Figure

Figure S1. Molecular docking analysis of STAT3 with *Paris polyphylla* bioactive compounds.

Figure S2. Molecular docking analysis of EGFR with *Paris polyphylla* bioactive compounds.

Figure S3. Molecular docking analysis of SRC with *Paris polyphylla* bioactive compounds.

Figure S4. Molecular docking analysis of IL-6 with *Paris polyphylla* bioactive compounds.

Figure S5. Molecular docking analysis of AKT1 with *Paris polyphylla* bioactive compounds.

Figure S6. 2D molecular docking interaction diagram between STAT3 and pennogenin.

Figure S7. 2D molecular docking interaction diagram between STAT3 and diosgenin tetraglycoside.

Figure S8. 2D molecular docking interaction diagram between STAT3 and diosgenin.

Figure S9. 2D molecular docking interaction diagram between STAT3 and prosapogenin A.

Figure S10. 2D molecular docking interaction diagram between STAT3 and pregnane-3,20-diol.

Figure S11. 2D molecular docking interaction diagram between STAT3 and spirostanol.

Figure S12. 2D molecular docking interaction diagram between STAT3 and diosmetin.

Figure S13. 2D molecular docking interaction diagram between STAT3 and flavone.

Figure S14. 2D molecular docking interaction diagram between STAT3 and 20-hydroxyecdysone.

Figure S15. 2D molecular docking interaction diagram between STAT3 and kaempferol 3-gentiobioside-7-rhamnoside.

Figure S16. 2D molecular docking interaction diagram between STAT3 and dextrin.

Figure S17. 2D molecular docking interaction diagram between STAT3 and polyphyllin E (RG).

Figure S18. 2D molecular docking interaction diagram between EGFR and spirostanol.

Figure S19. 2D molecular docking interaction diagram between EGFR and diosgenin.

Figure S20. 2D molecular docking interaction diagram between EGFR and pennogenin.

Figure S21. 2D molecular docking interaction diagram between EGFR and pregnane-3,20-diol.

Figure S22. 2D molecular docking interaction diagram between EGFR and prosapogenin A.

Figure S23. 2D molecular docking interaction diagram between EGFR and diosmetin.

Figure S24. 2D molecular docking interaction diagram between EGFR and flavone.

Figure S25. 2D molecular docking interaction diagram between EGFR and 20-hydroxyecdysone.

Figure S26. 2D molecular docking interaction diagram between EGFR and kaempferol 3-gentiobioside-7-rhamnoside.

Figure S27. 2D molecular docking interaction diagram between EGFR and dextrin.

Figure S28. 2D molecular docking interaction diagram between SRC and diosgenin tetraglycoside.

Figure S29. 2D molecular docking interaction diagram between SRC and spirostanol.

Figure S30. 2D molecular docking interaction diagram between SRC and pennogenin.

Figure S31. 2D molecular docking interaction diagram between SRC and 20-hydroxyecdysone.

Figure S32. 2D molecular docking interaction diagram between SRC and diosgenin.

Figure S33. 2D molecular docking interaction diagram between SRC and pregnane-3,20-diol.

Figure S34. 2D molecular docking interaction diagram between SRC and prosapogenin A.

Figure S35. 2D molecular docking interaction diagram between SRC and flavone.

Figure S36. 2D molecular docking interaction diagram between SRC and diosmetin.

Figure S37. 2D molecular docking interaction diagram between SRC and kaempferol 3-gentiobioside-7-rhamnoside.

Figure S38. 2D molecular docking interaction diagram between IL-6 and diosgenin tetraglycoside.

Figure S39. 2D molecular docking interaction diagram between IL-6 and spirostanol.

Figure S40. 2D molecular docking interaction diagram between IL-6 and diosgenin.

Figure S41. 2D molecular docking interaction diagram between IL-6 and pregnane-3,20-diol.

Figure S42. 2D molecular docking interaction diagram between IL-6 and diosmetin.

Figure S43. 2D molecular docking interaction diagram between IL-6 and 20-hydroxyecdysone.

Figure S44. 2D molecular docking interaction diagram between IL-6 and prosapogenin A.

Figure S45. 2D molecular docking interaction diagram between AKT1 and prosapogenin A.

Figure S46. 2D molecular docking interaction diagram between AKT1 and spirostanol.

Figure S47. 2D molecular docking interaction diagram between AKT1 and diosgenin.

Figure S48. 2D molecular docking interaction diagram between AKT1 and pennogenin.

Figure S49. 2D molecular docking interaction diagram between AKT1 and 20-hydroxyecdysone.

Figure S50. 2D molecular docking interaction diagram between AKT1 and pregnane-3,20-diol.

Figure S51. 2D molecular docking interaction diagram between AKT1 and diosmetin.

Figure S52. 2D molecular docking interaction diagram between AKT1 and flavone.

Figure S53. 2D molecular docking interaction diagram between AKT1 and kaempferol 3-gentiobioside-7-rhamnoside.

Figure S54. 2D molecular docking interaction diagram between AKT1 and dextrin.

Figure S55. Workflow of the network pharmacology analysis of *Paris polyphylla* against colorectal cancer.

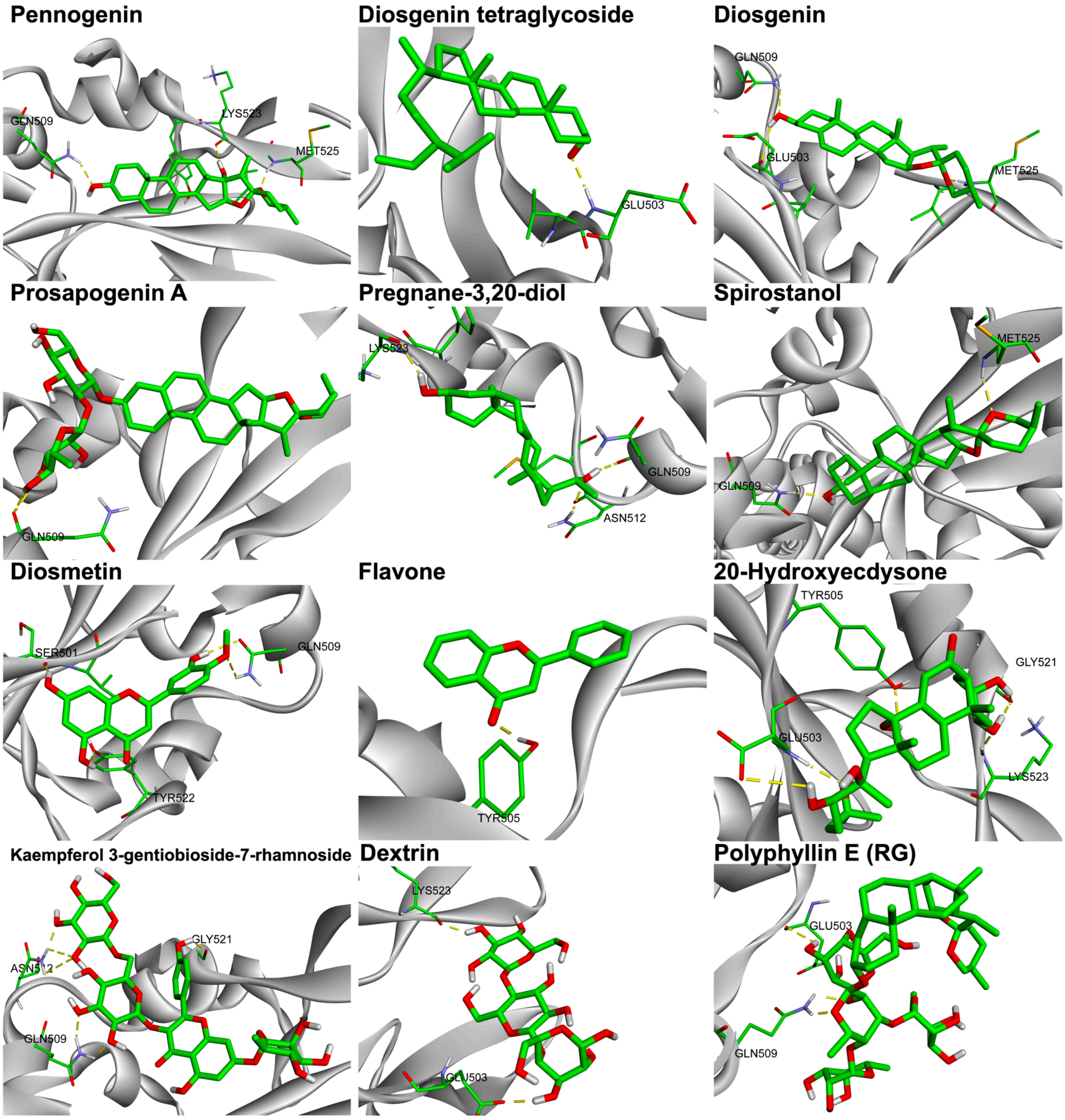

Figure S1. Molecular docking analysis of STAT3 with *Paris polyphylla* bioactive compounds. Three-dimensional binding conformations of STAT3 with pennogenin, diosgenin tetraglycoside, diosgenin, prosapogenin A, pregnane-3,20-diol, spirostanol, diosmetin, flavone, 20-hydroxyecdysone, kaempferol 3-gentiobioside-7-rhamnoside, dextrin, and polyphyllin E (RG). Carbon atoms are shown in green, oxygen in red, nitrogen in purple, sulfur in orange, and hydrogen in white. Yellow dashed lines indicate hydrogen bond interactions.

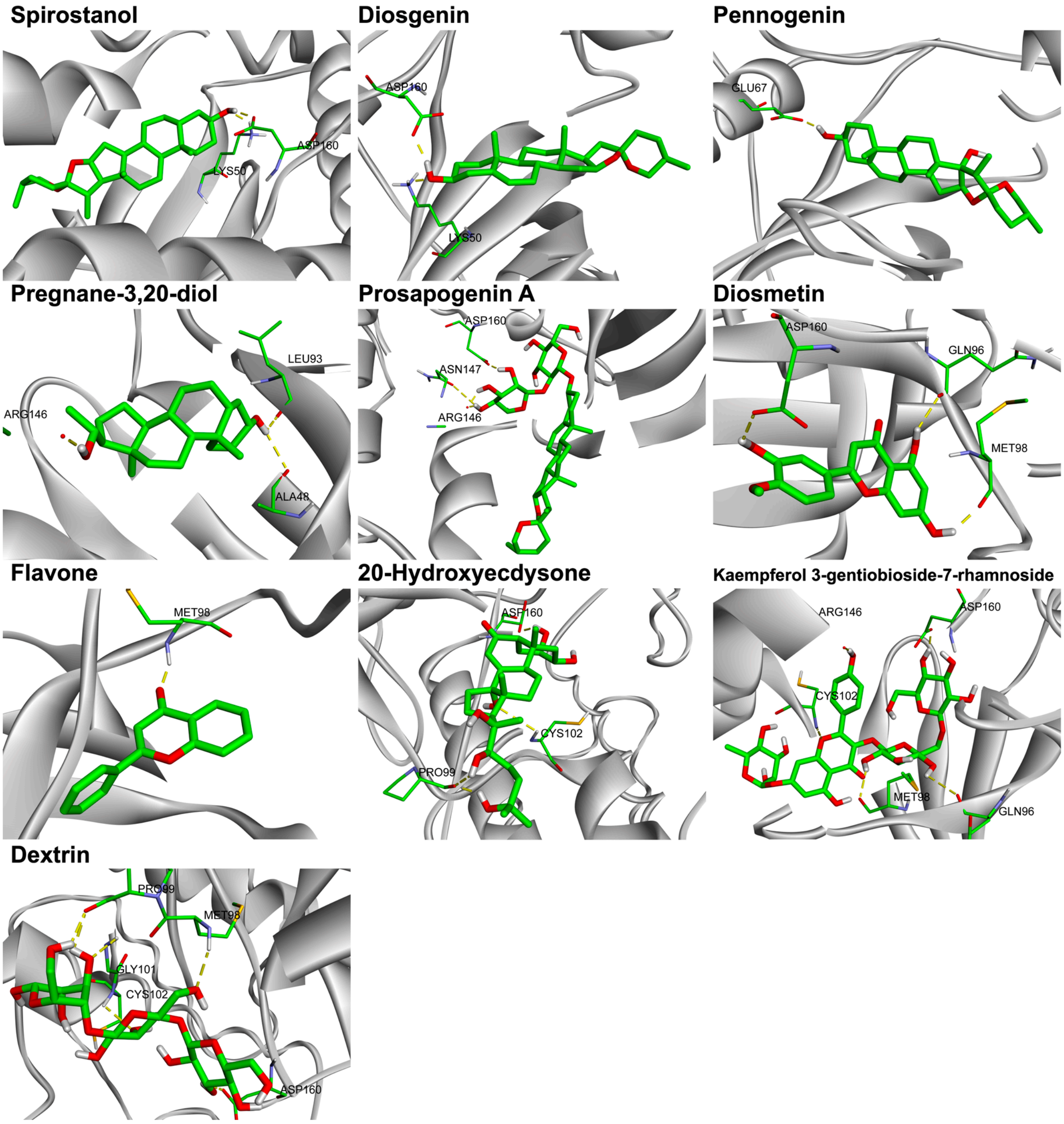

Figure S2. Molecular docking analysis of EGFR with *Paris polyphylla* bioactive compounds. Three-dimensional binding conformations of EGFR with spirostanol, diosgenin, pennogenin, pregnane-3,20-diol, prosapogenin A, diosmetin, flavone, 20-hydroxyecdysone, kaempferol 3-gentiobioside-7-rhamnoside, and dextrin. Carbon atoms are shown in green, oxygen in red, nitrogen in purple, sulfur in orange, and hydrogen in white. Yellow dashed lines indicate hydrogen bond interactions.

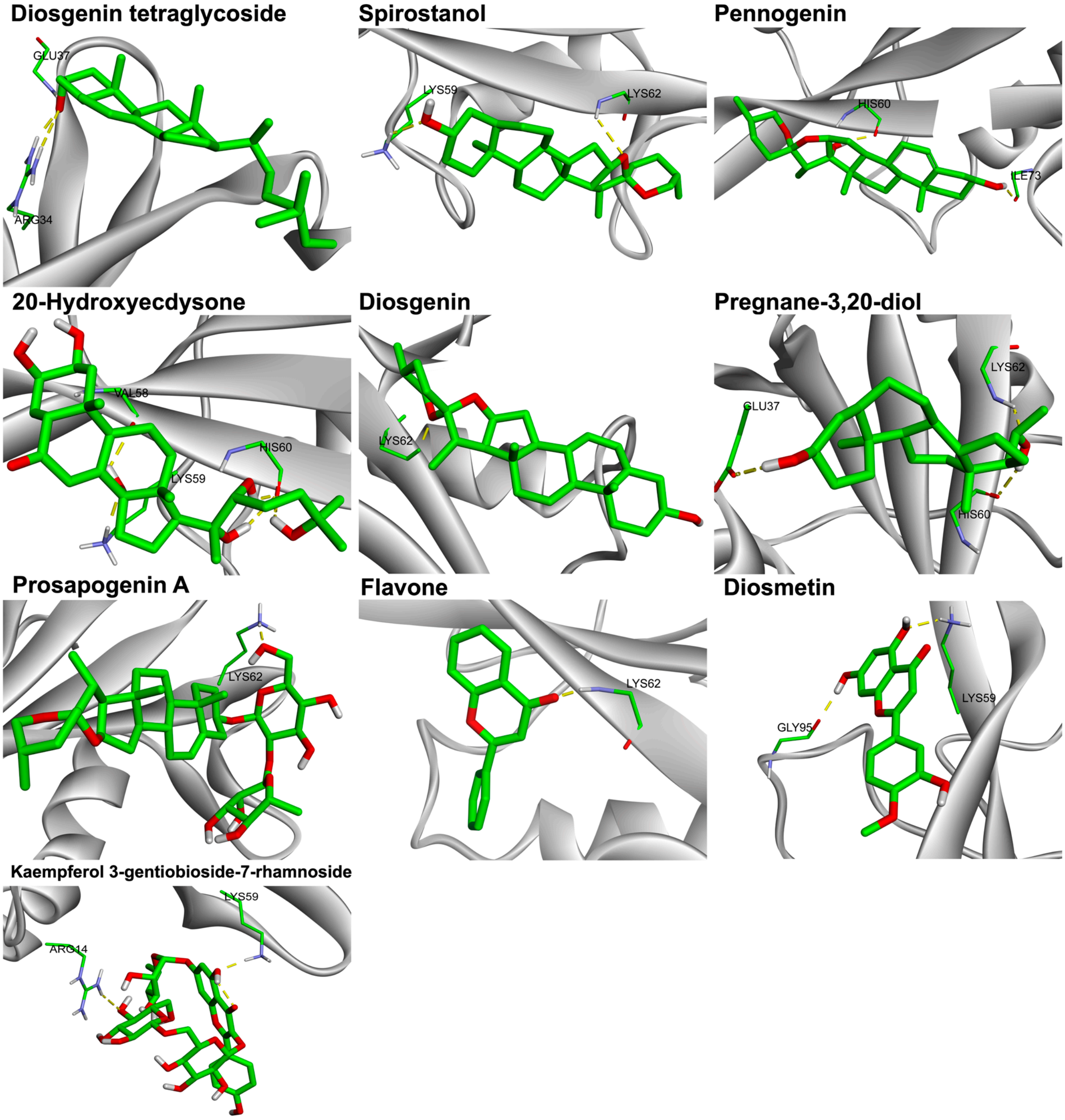

Figure S3. Molecular docking analysis of SRC with *Paris polyphylla* bioactive compounds. Three-dimensional binding conformations of SRC with diosgenin tetraglycoside, spirostanol, pennogenin, 20-hydroxyecdysone, diosgenin, pregnane-3,20-diol, prosapogenin A, flavone, diosmetin, and kaempferol 3-gentiobioside-7-rhamnoside. Carbon atoms are shown in green, oxygen in red, nitrogen in purple, and hydrogen in white. Yellow dashed lines indicate hydrogen bond interactions.

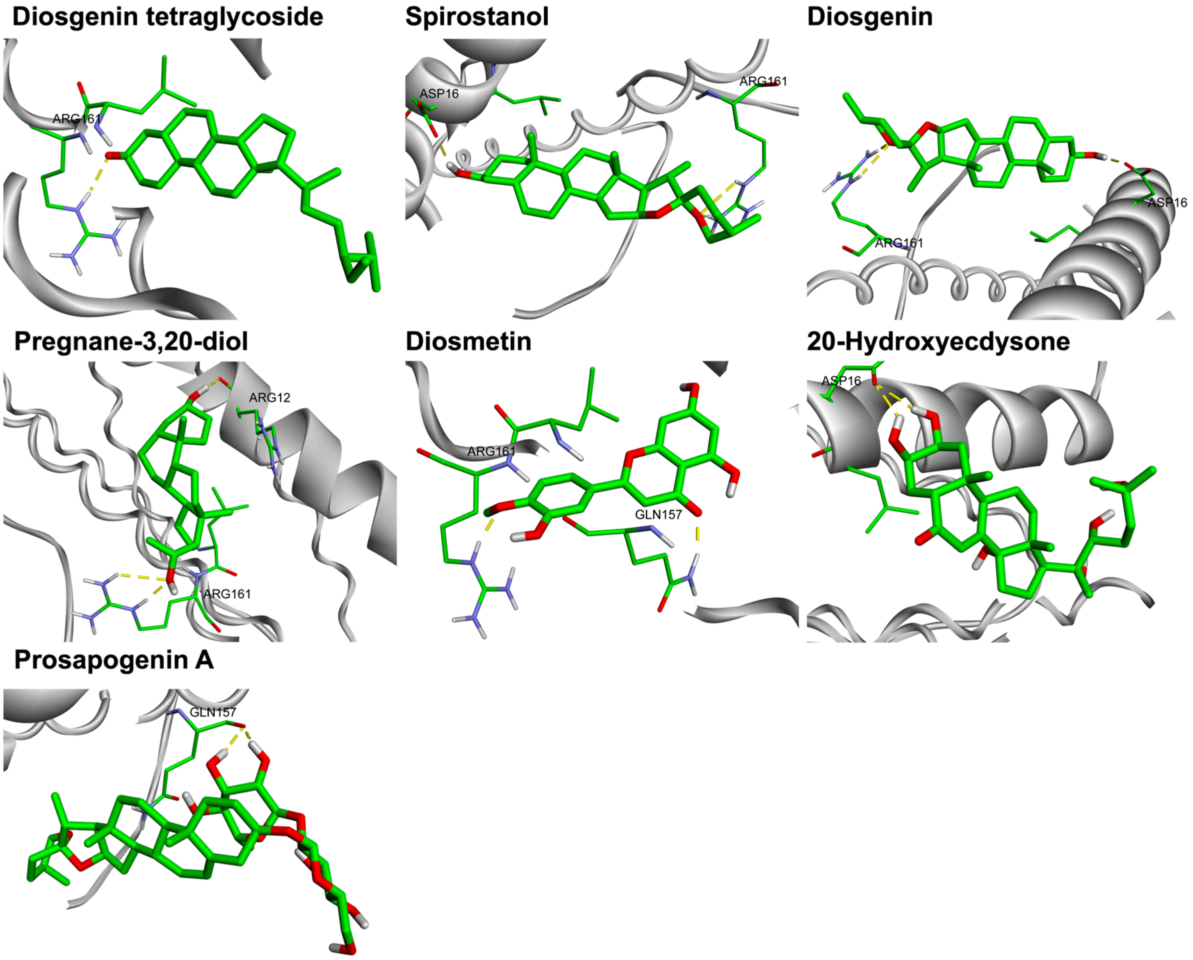

Figure S4. Molecular docking analysis of IL-6 with *Paris polyphylla* bioactive compounds. Three-dimensional binding conformations of IL-6 with diosgenin tetraglycoside, spirostanol, diosgenin, pregnane-3,20-diol, diosmetin, 20-hydroxyecdysone, and prosapogenin A. Carbon atoms are shown in green, oxygen in red, nitrogen in purple, and hydrogen in white. Yellow dashed lines indicate hydrogen bond interactions.

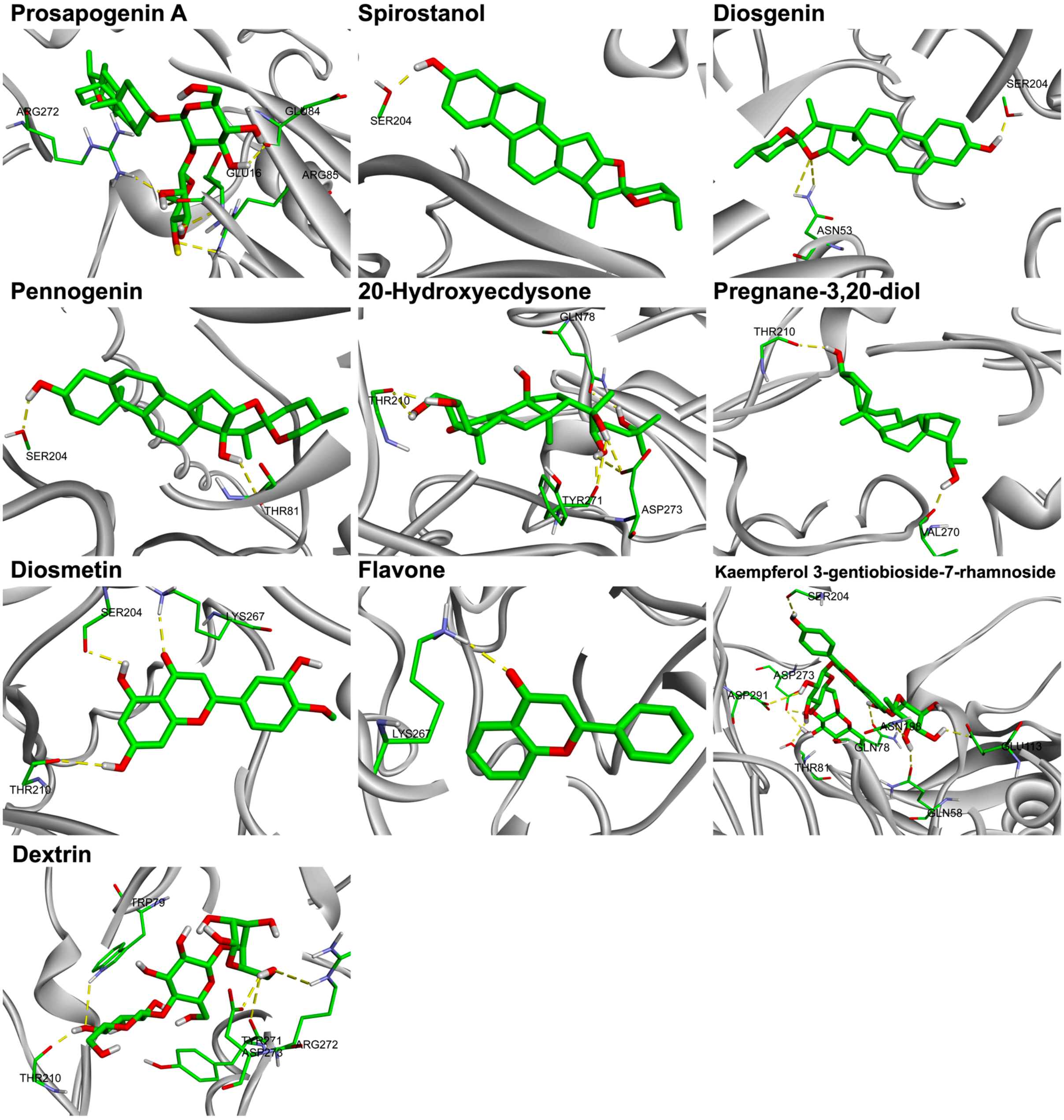

Figure S5. Molecular docking analysis of AKT1 with *Paris polyphylla* bioactive compounds. Three-dimensional binding conformations of AKT1 with prosapogenin A, spirostanol, diosgenin, pennogenin, 20-hydroxyecdysone, pregnane-3,20-diol, diosmetin, flavone, kaempferol 3-gentiobioside-7-rhamnoside, and dextrin. Carbon atoms are shown in green, oxygen in red, nitrogen in purple, and hydrogen in white. Yellow dashed lines indicate hydrogen bond interactions.

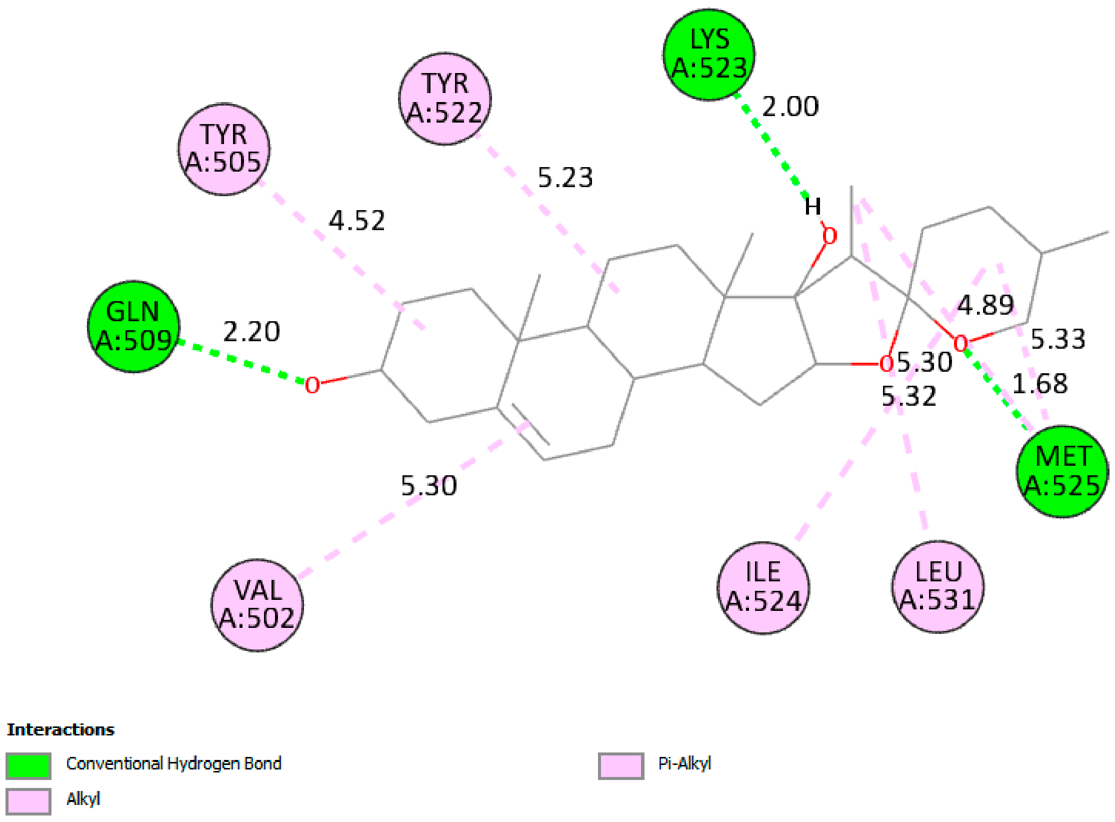

Figure S6. 2D molecular docking interaction diagram between STAT3 and pennogenin.

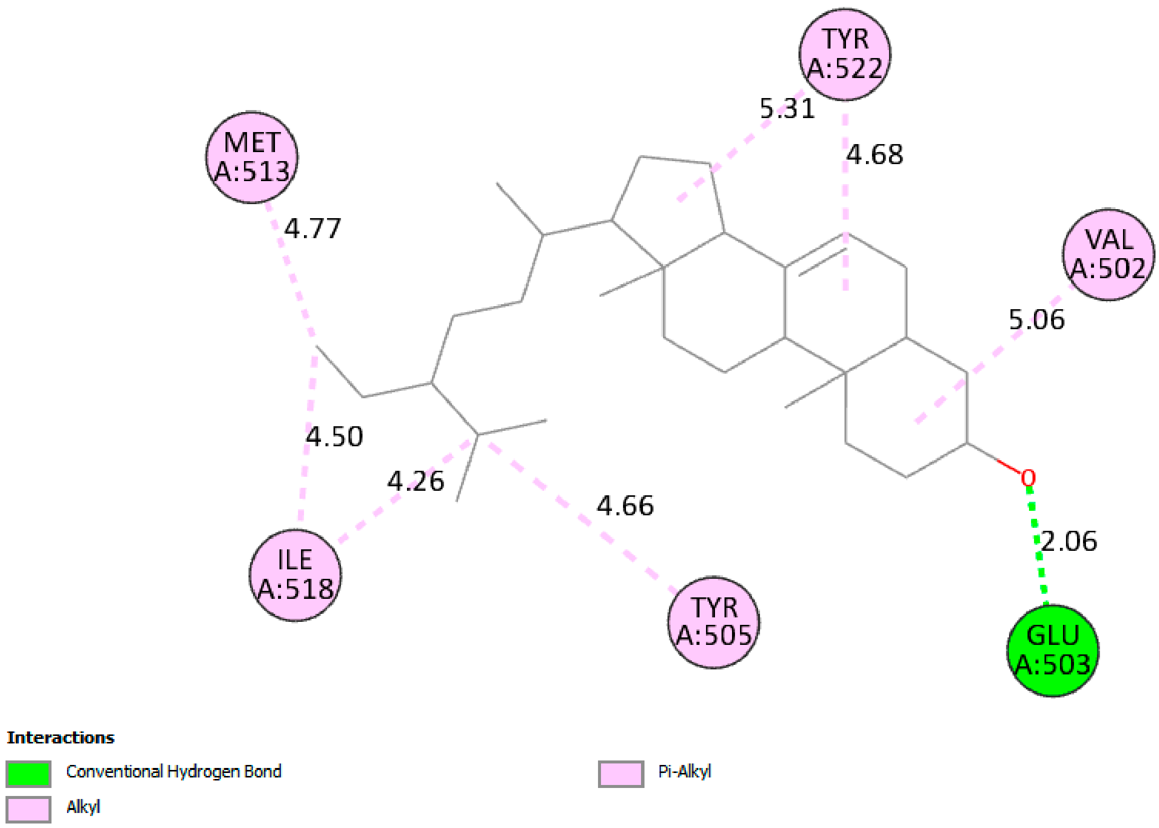

Figure S7. 2D molecular docking interaction diagram between STAT3 and diosgenin tetraglycoside.

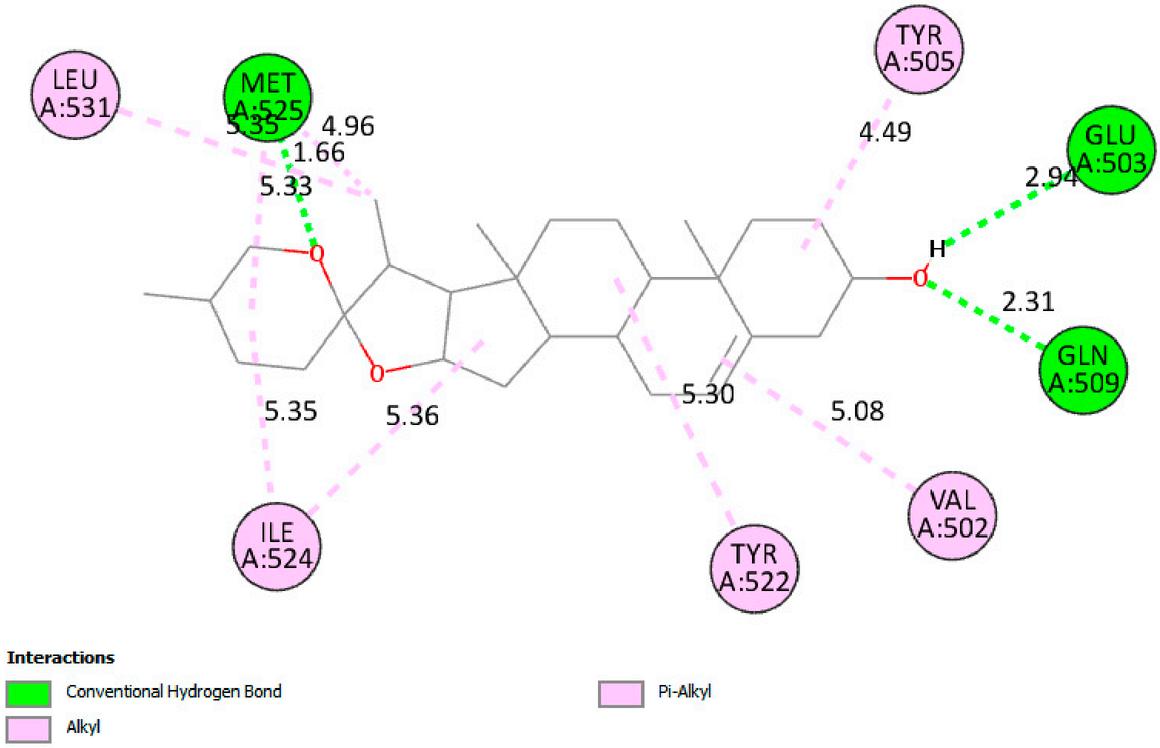

Figure S8. 2D molecular docking interaction diagram between STAT3 and diosgenin.

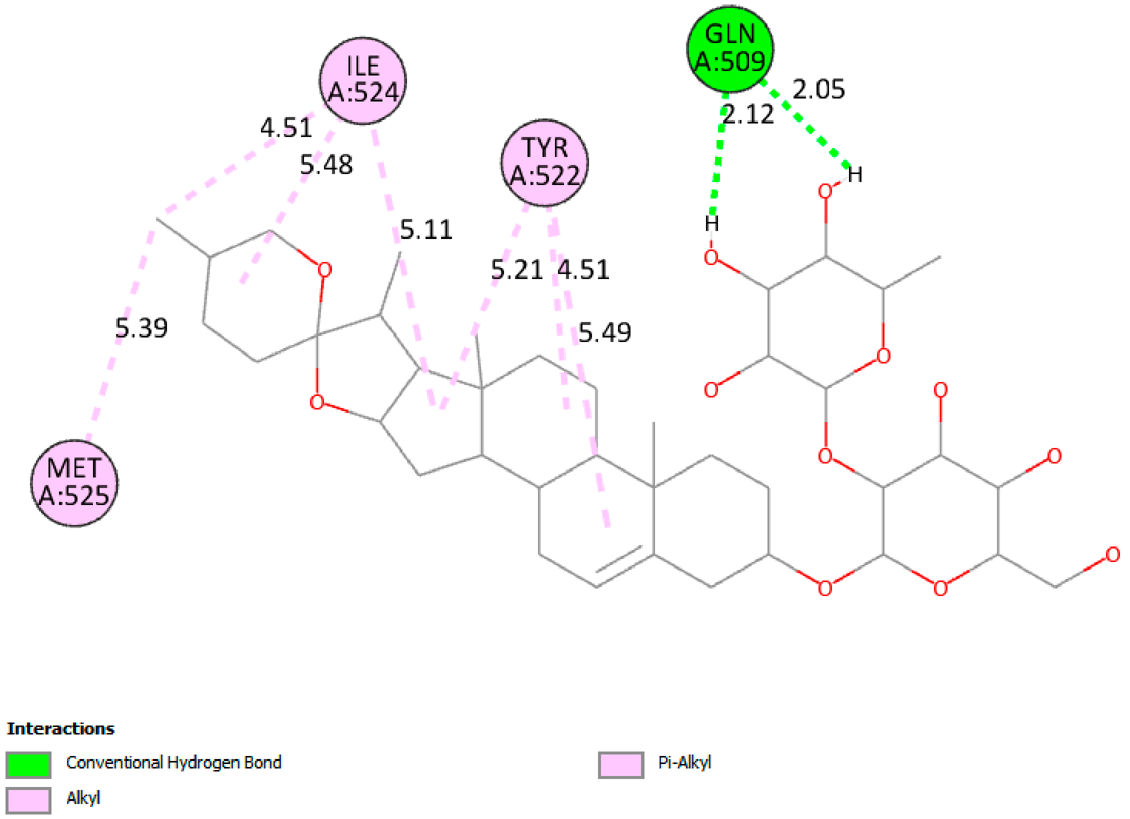

Figure S9. 2D molecular docking interaction diagram between STAT3 and prosapogenin A.

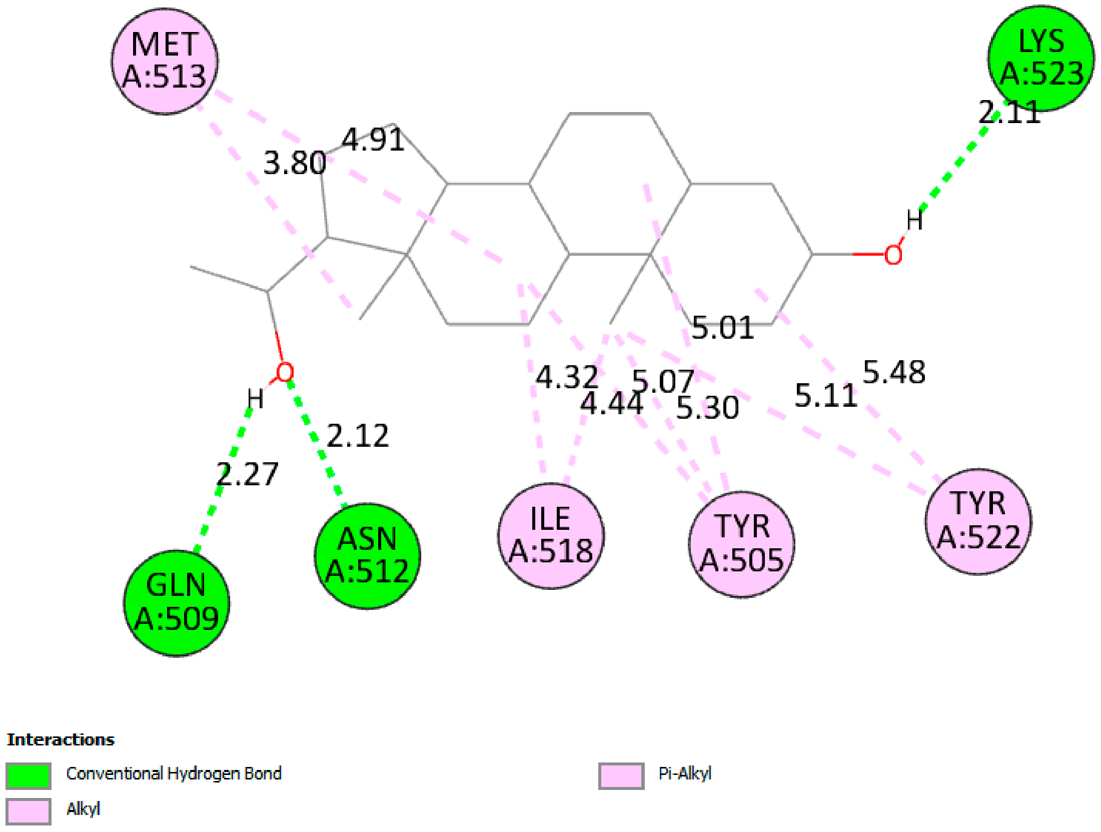

Figure S10. 2D molecular docking interaction diagram between STAT3 and pregnane-3,20-diol.

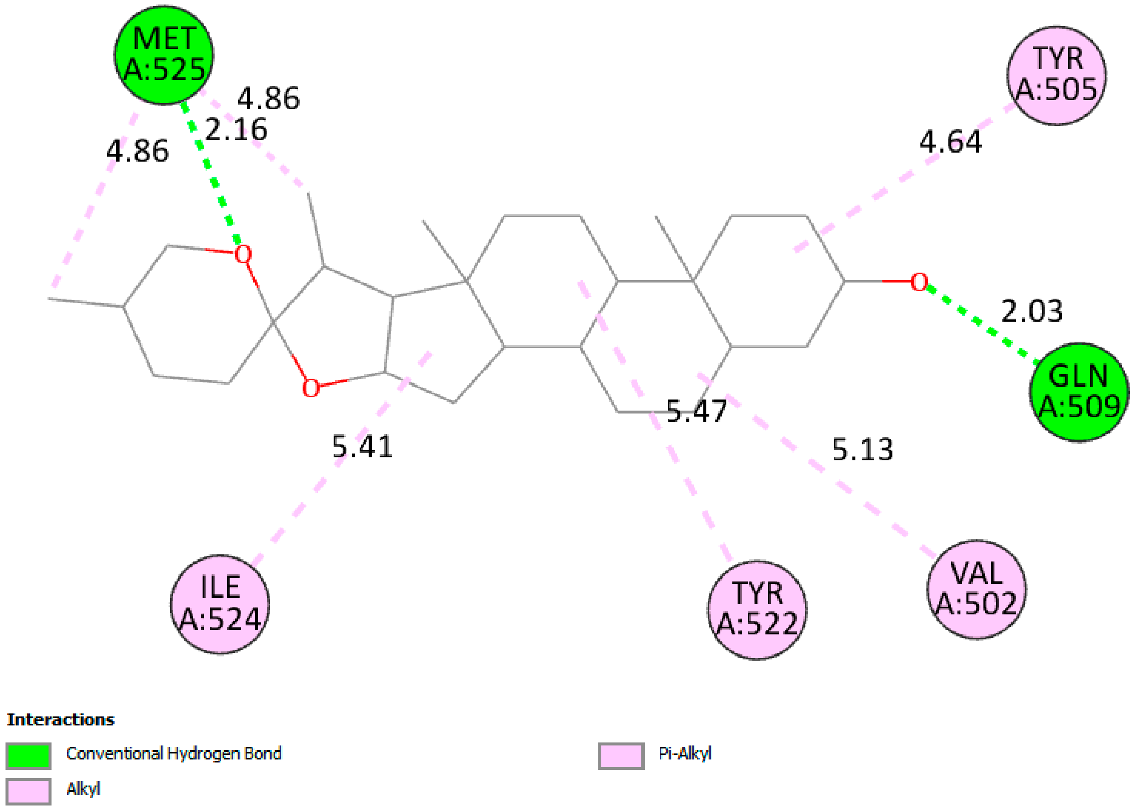

Figure S11. 2D molecular docking interaction diagram between STAT3 and spirostanol.

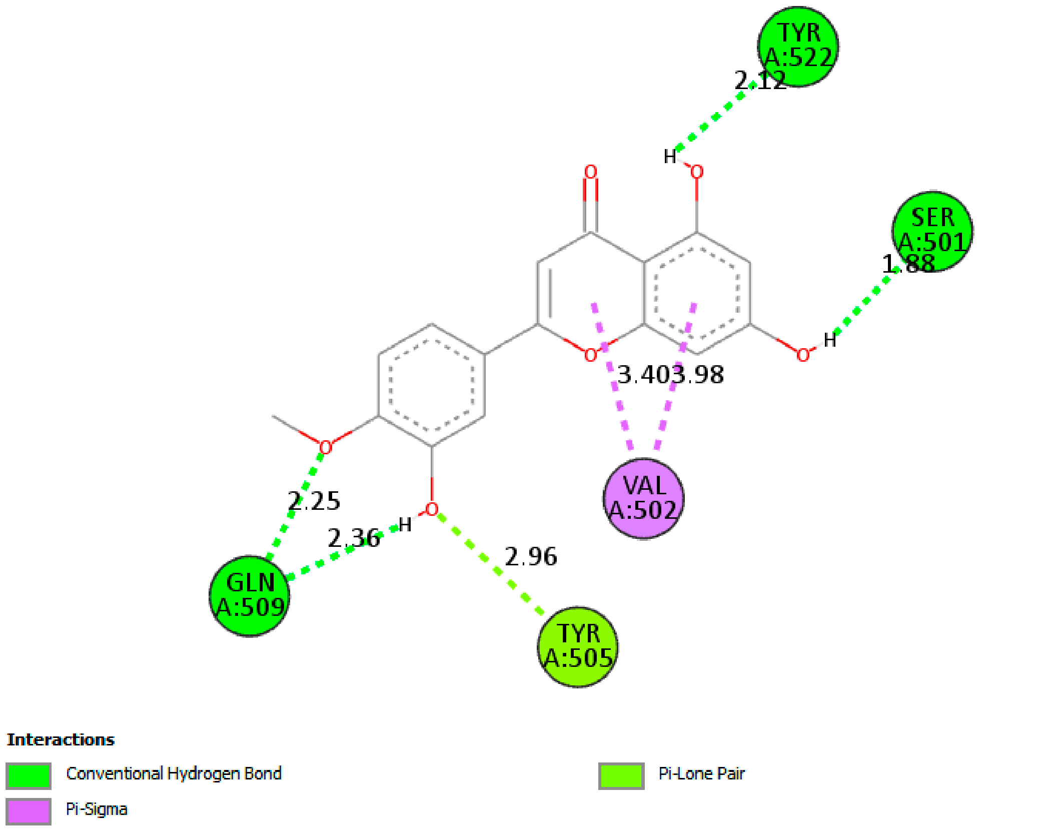

Figure S12. 2D molecular docking interaction diagram between STAT3 and diosmetin.

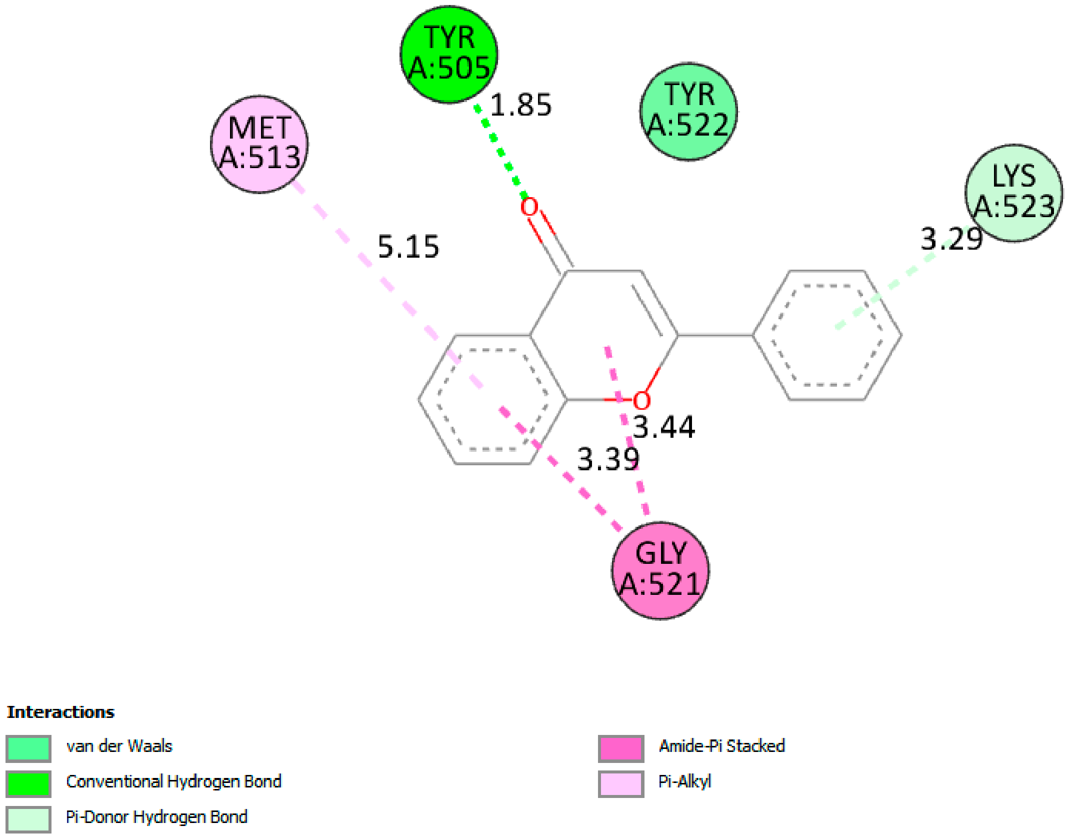

Figure S13. 2D molecular docking interaction diagram between STAT3 and flavone.

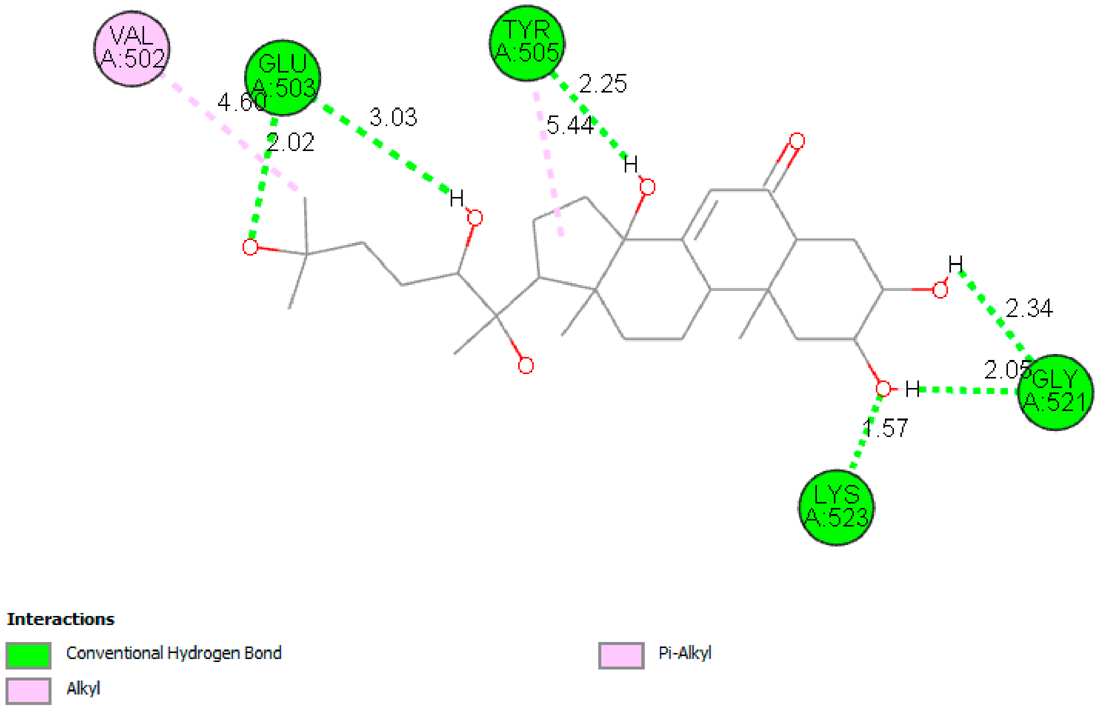

Figure S14. 2D molecular docking interaction diagram between STAT3 and 20-hydroxyecdysone.

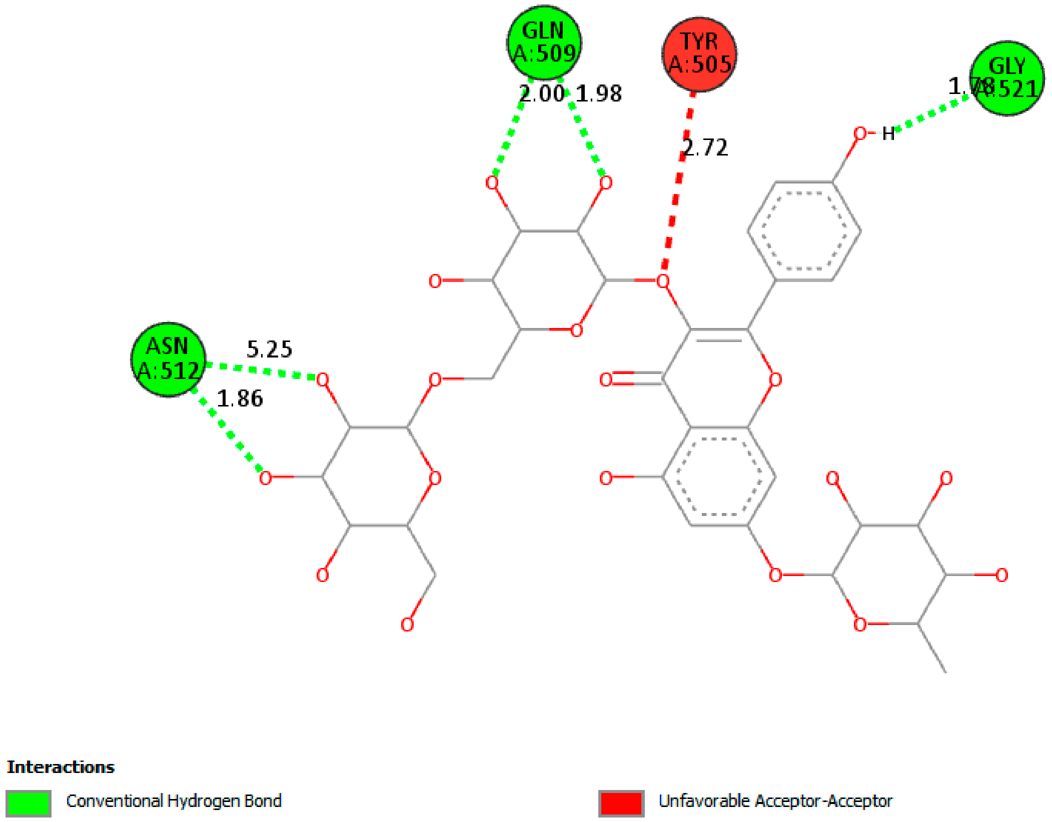

Figure S15. 2D molecular docking interaction diagram between STAT3 and kaempferol 3-gentiobioside-7-rhamnoside.

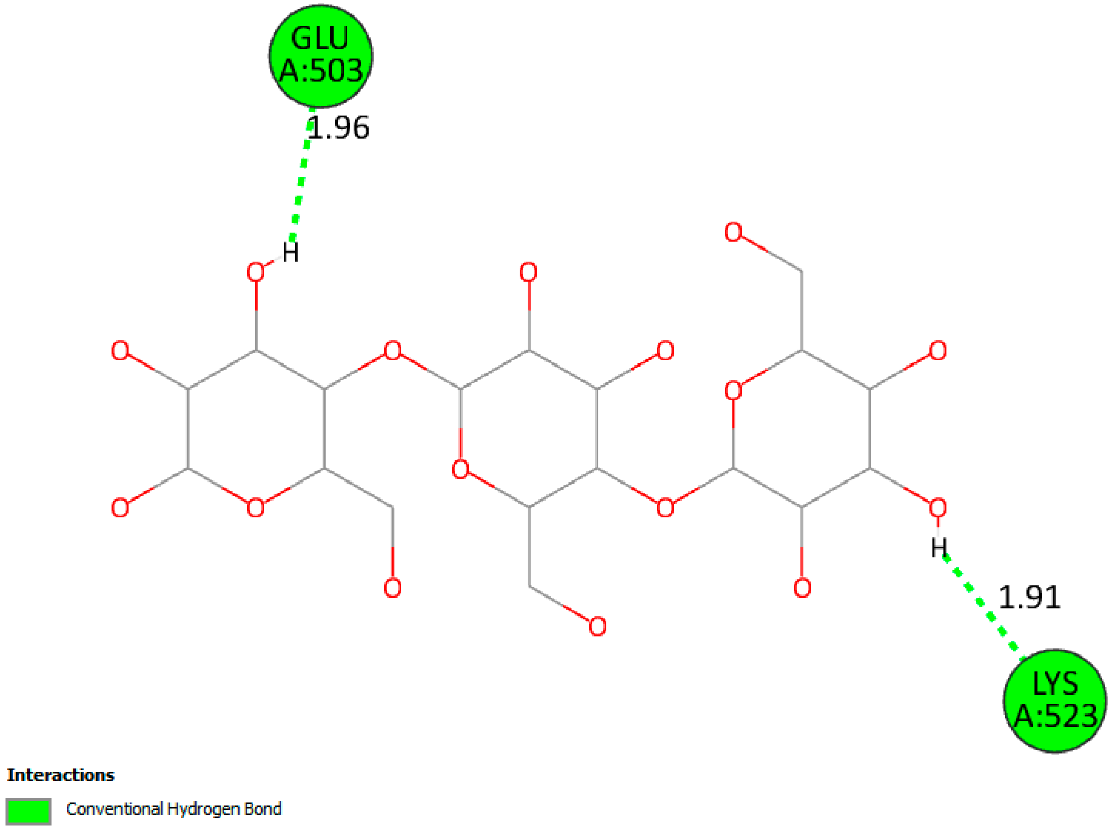

Figure S16. 2D molecular docking interaction diagram between STAT3 and dextrin.

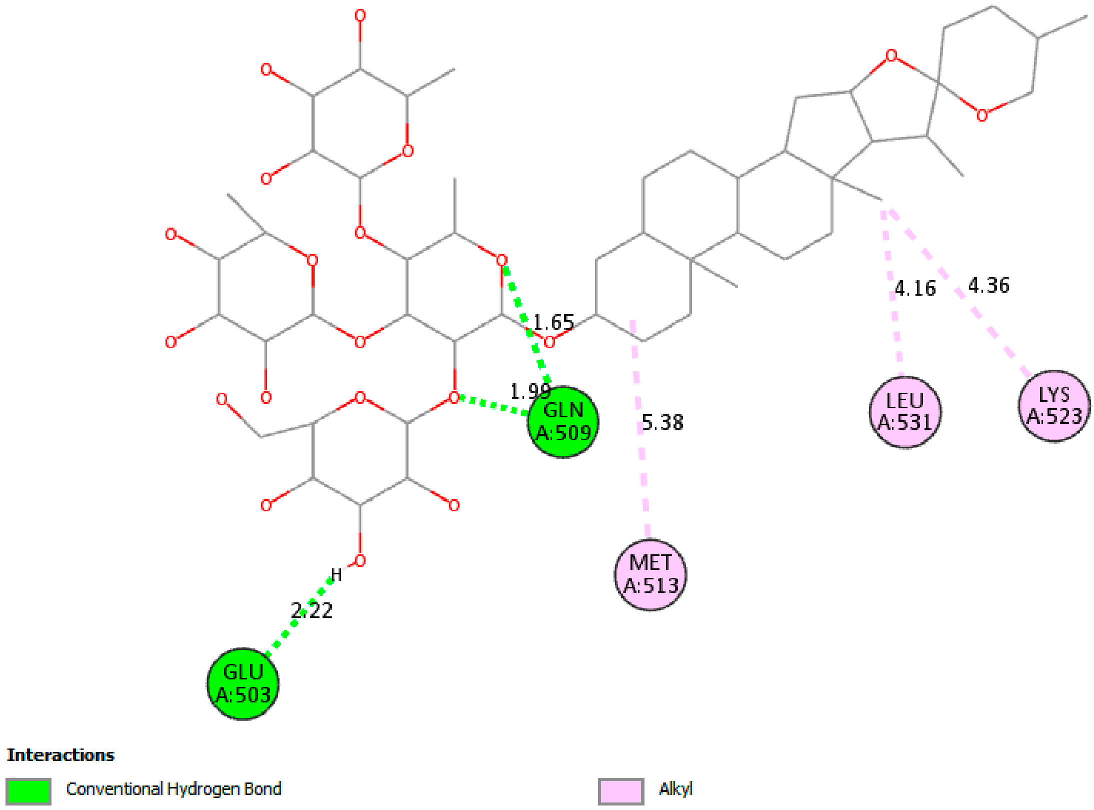

Figure S17. 2D molecular docking interaction diagram between STAT3 and polyphyllin E (RG).

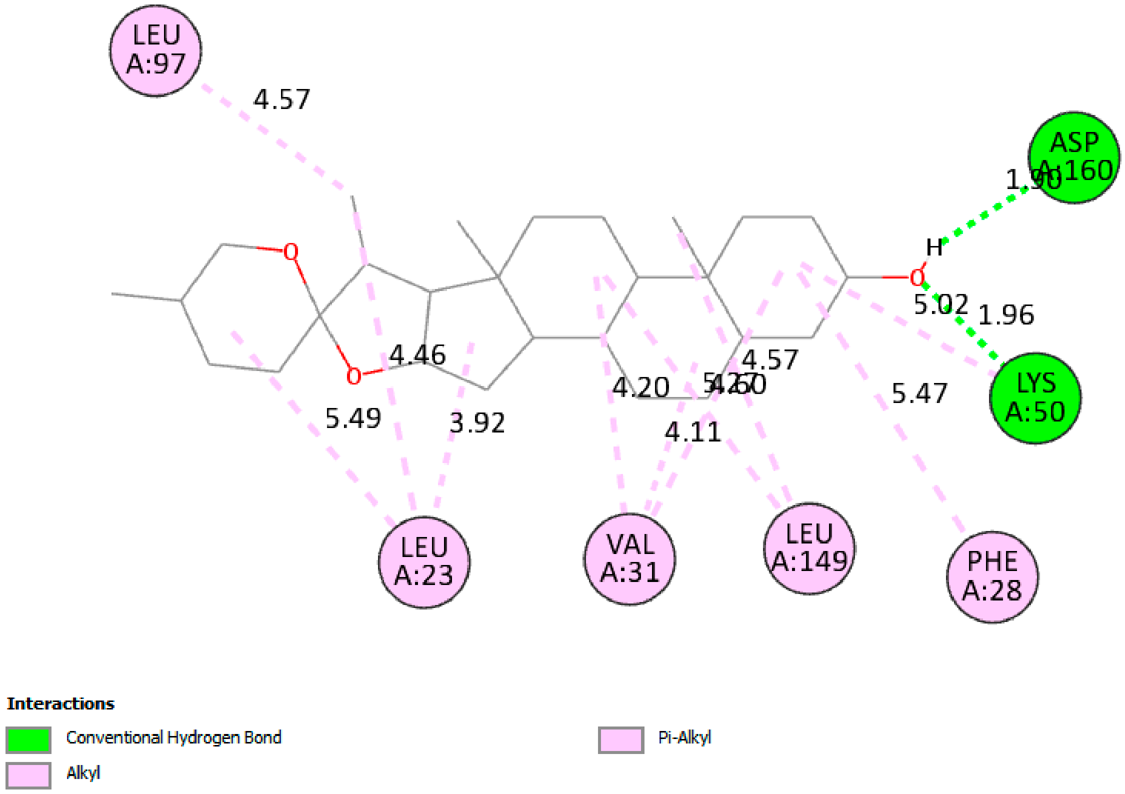

Figure S18. 2D molecular docking interaction diagram between EGFR and spirostanol.

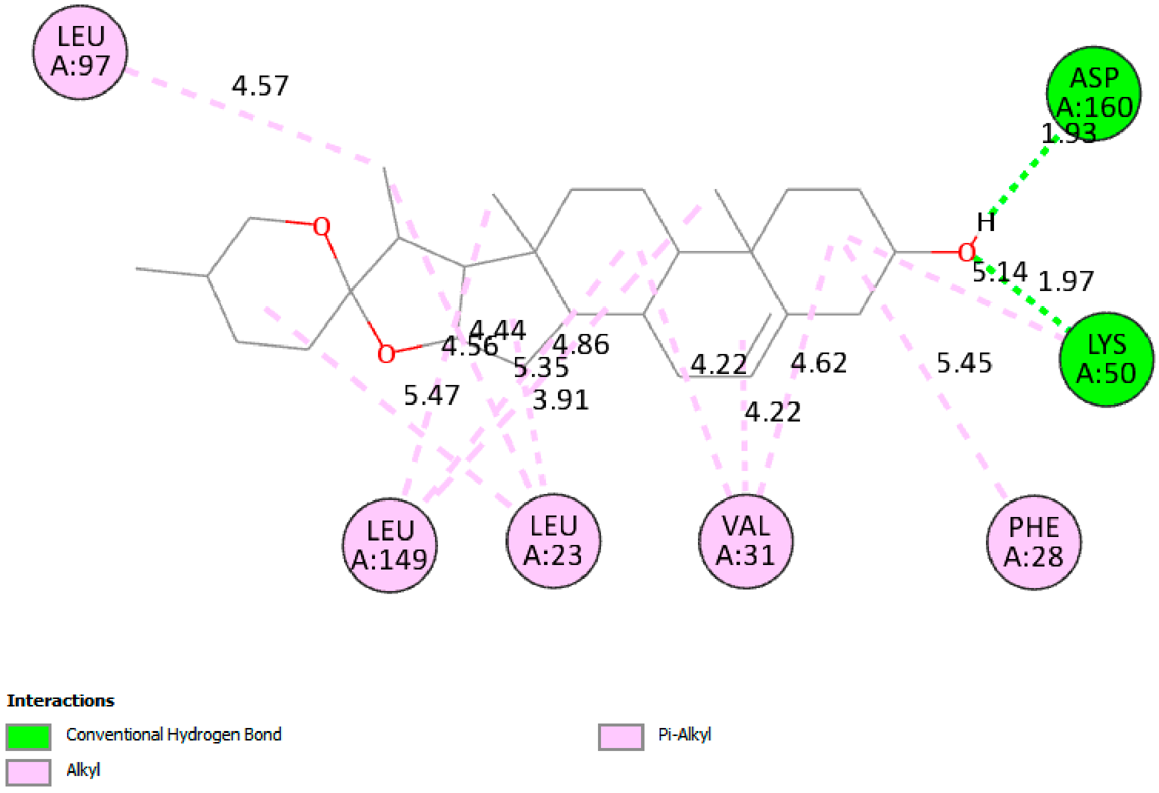

Figure S19. 2D molecular docking interaction diagram between EGFR and diosgenin.

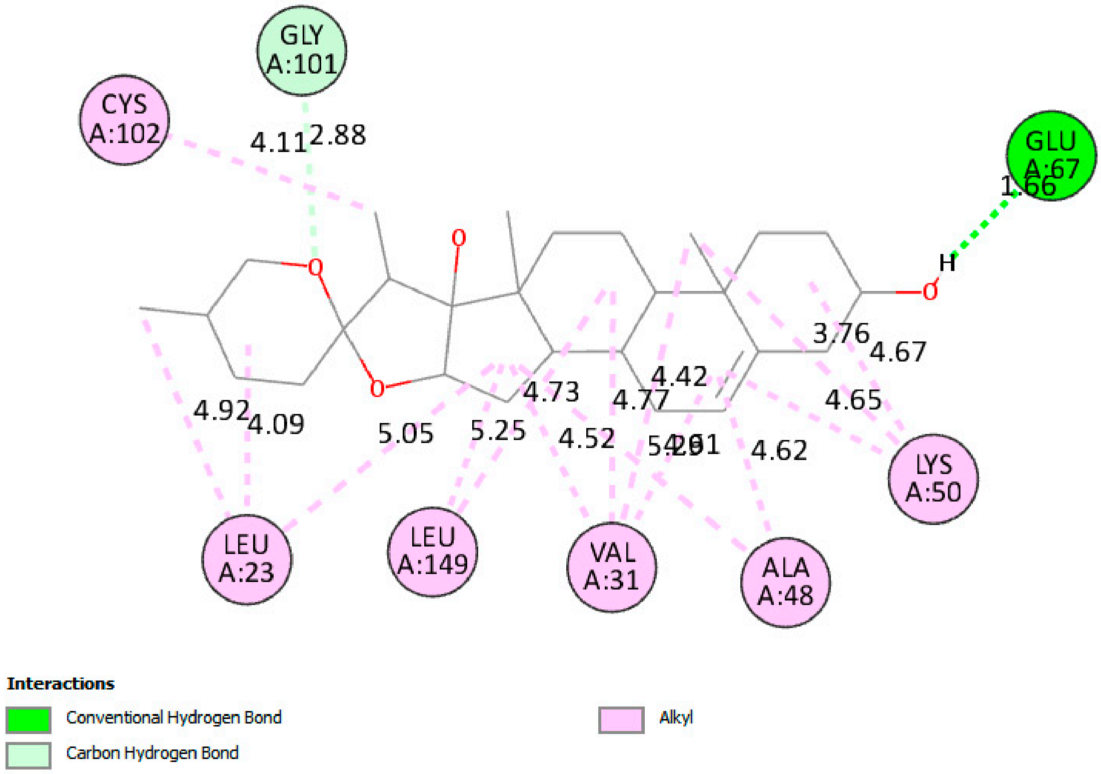

Figure S20. 2D molecular docking interaction diagram between EGFR and pennogenin.

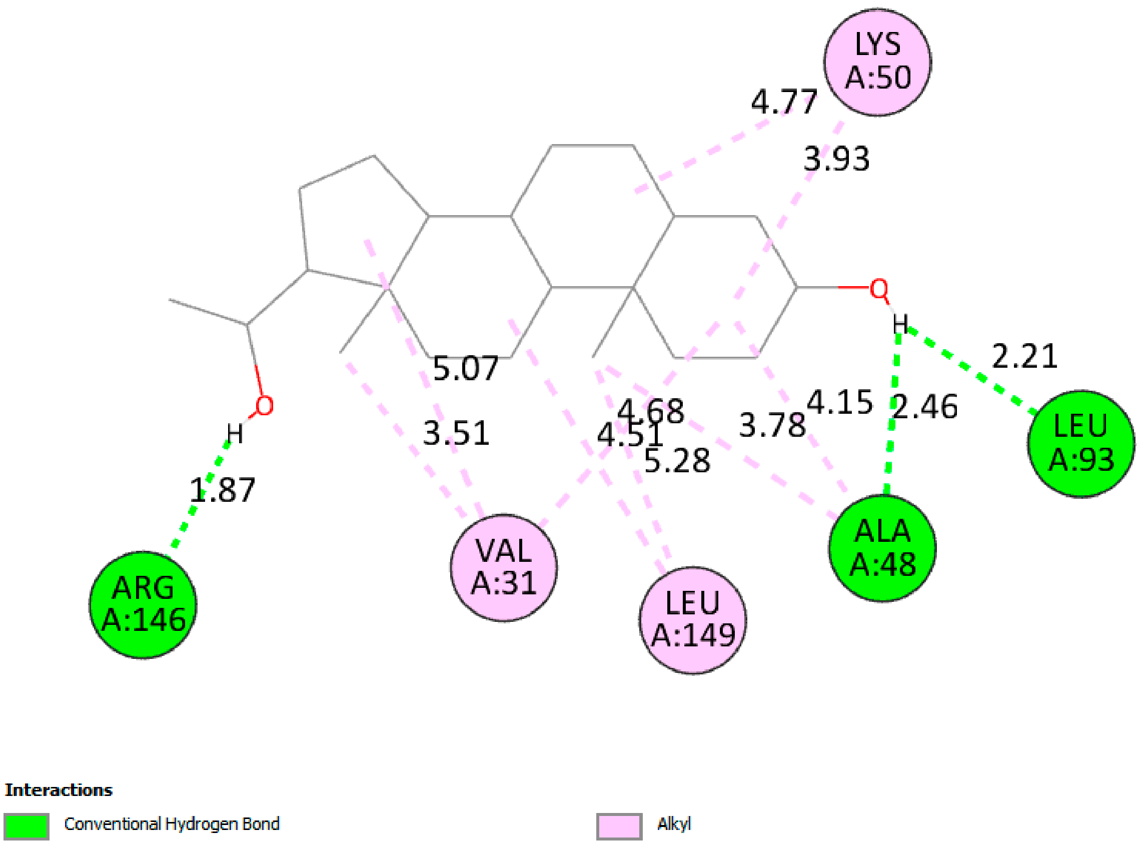

Figure S21. 2D molecular docking interaction diagram between EGFR and pregnane-3,20-diol.

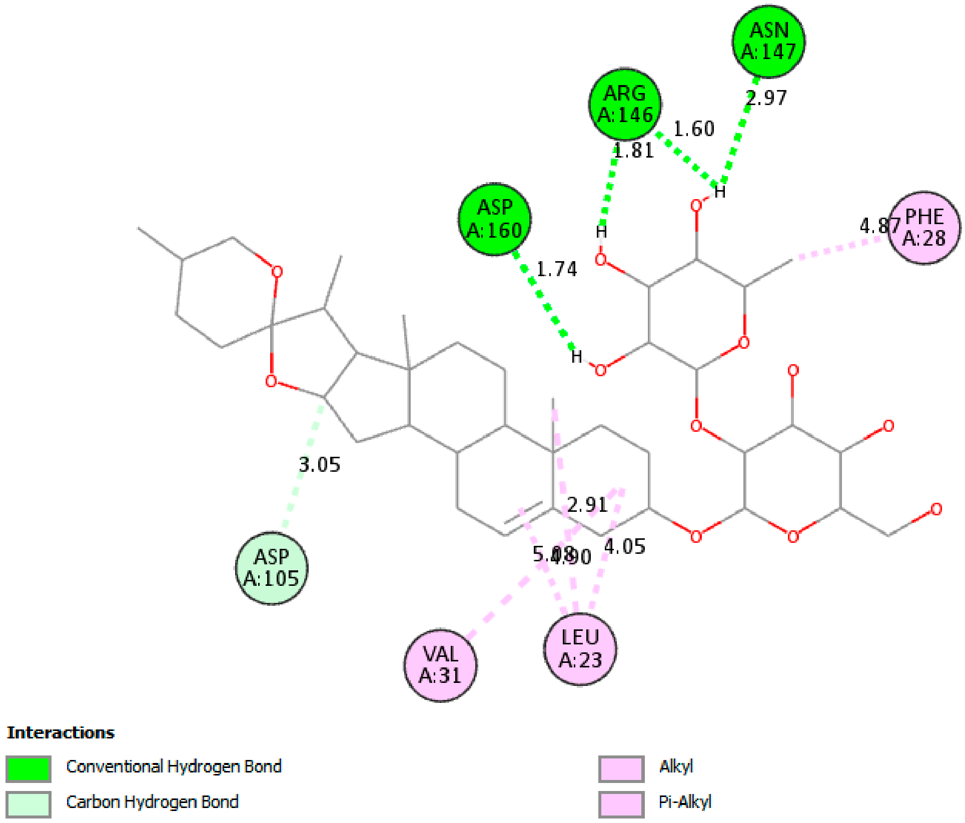

Figure S22. 2D molecular docking interaction diagram between EGFR and prosapogenin A.

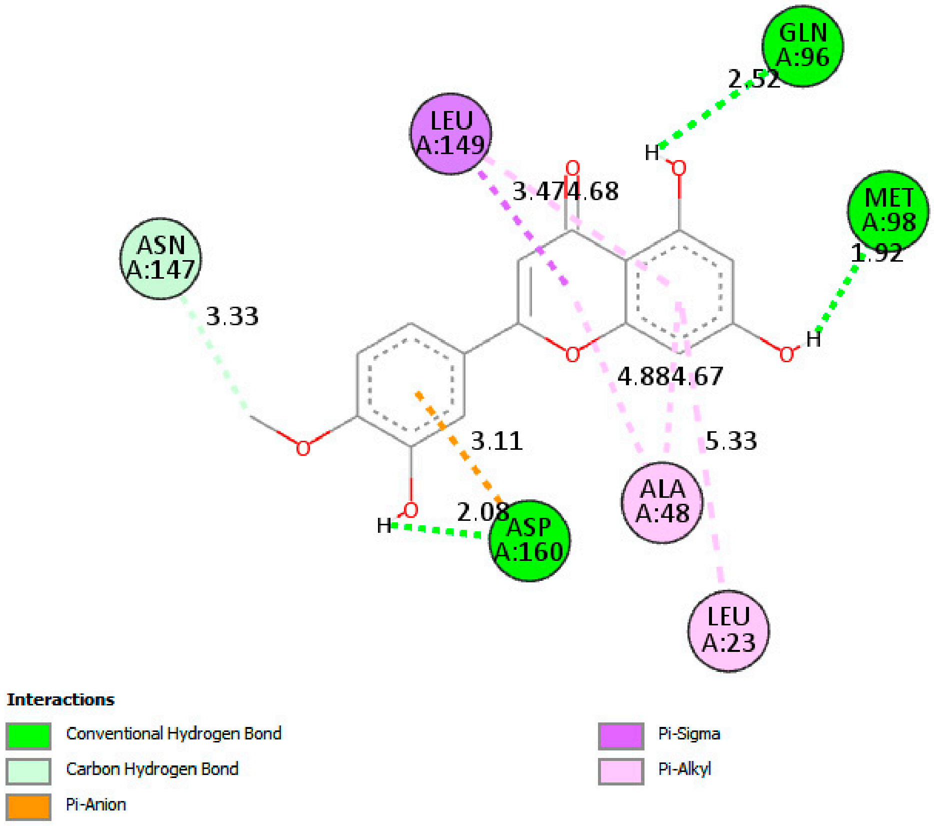

Figure S23. 2D molecular docking interaction diagram between EGFR and diosmetin.

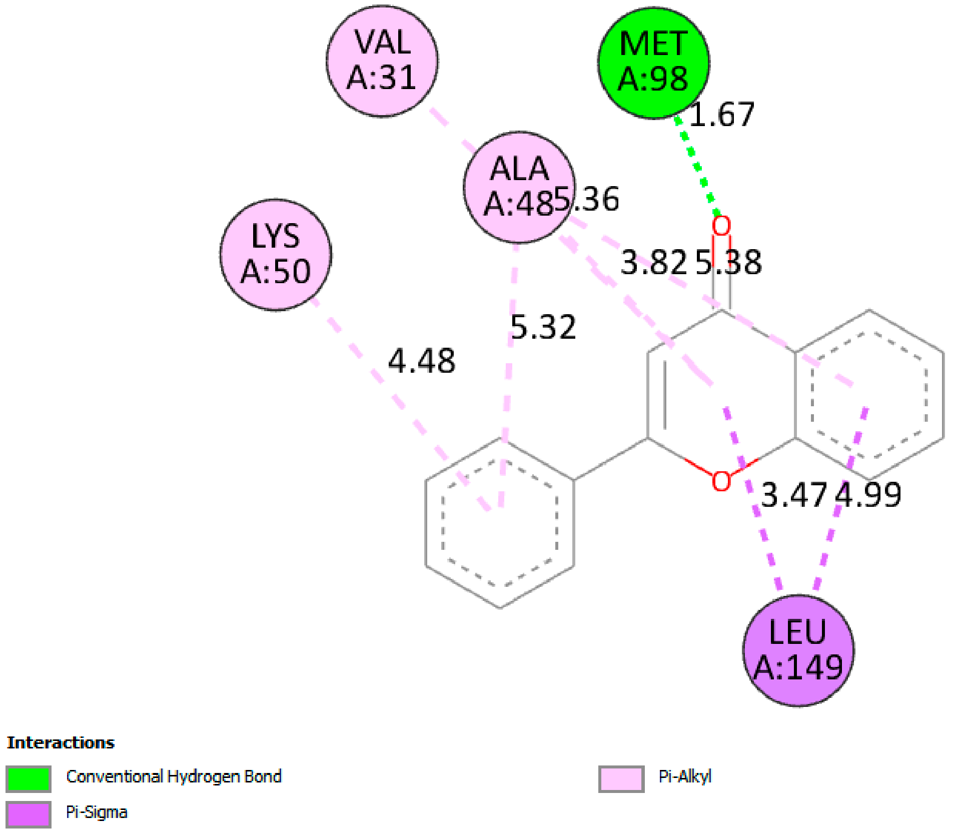

Figure S24. 2D molecular docking interaction diagram between EGFR and flavone.

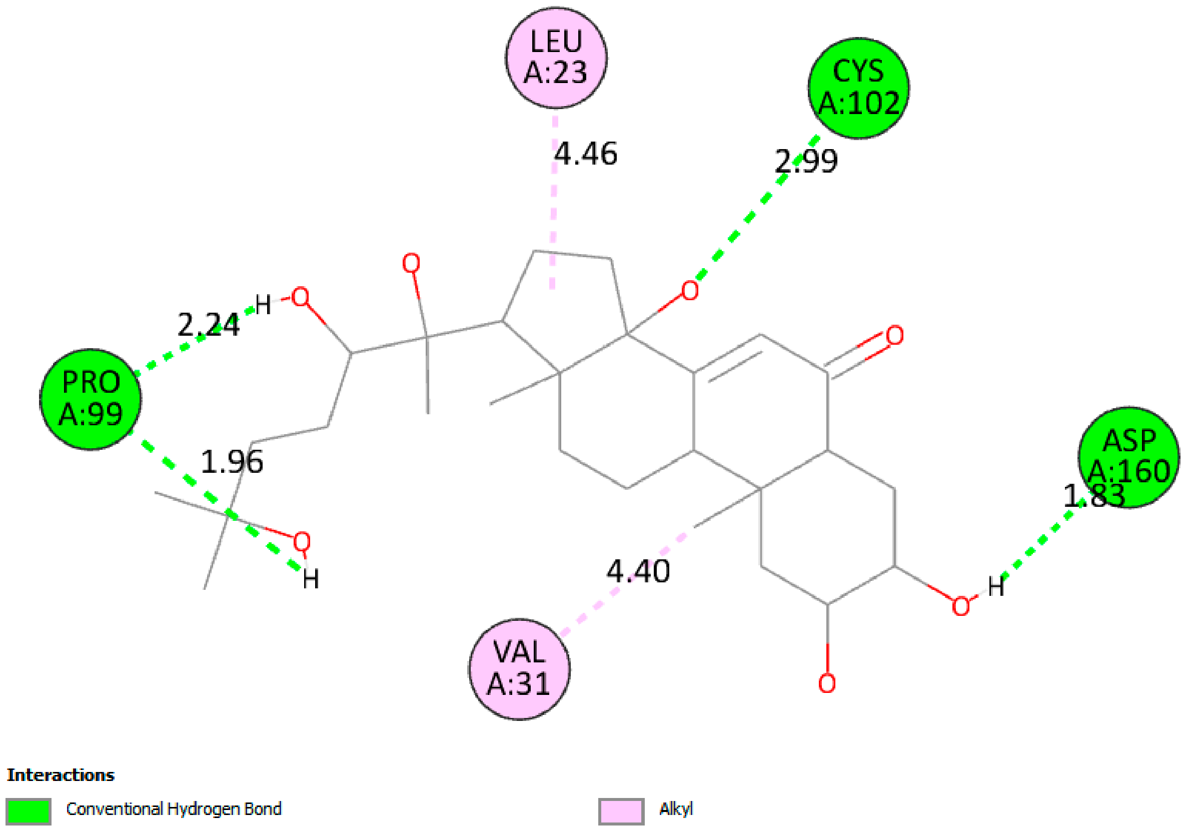

Figure S25. 2D molecular docking interaction diagram between EGFR and 20-hydroxyecdysone.

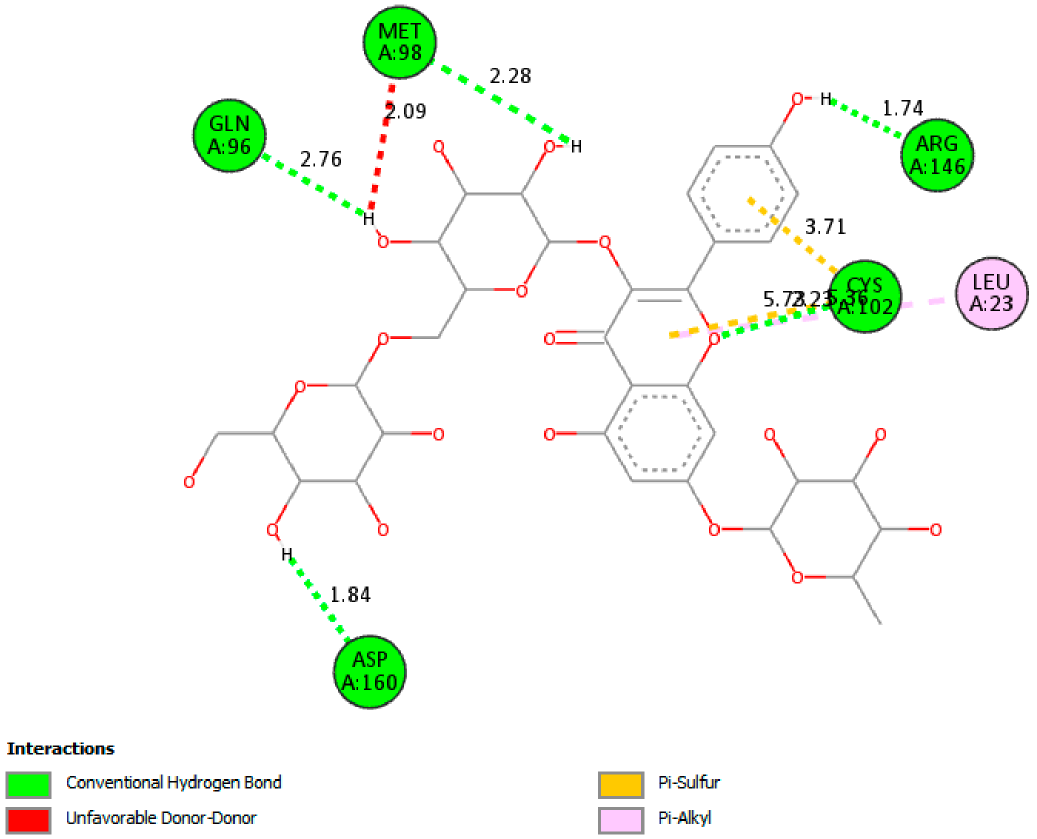

Figure S26. 2D molecular docking interaction diagram between EGFR and kaempferol 3-gentiobioside-7-rhamnoside.

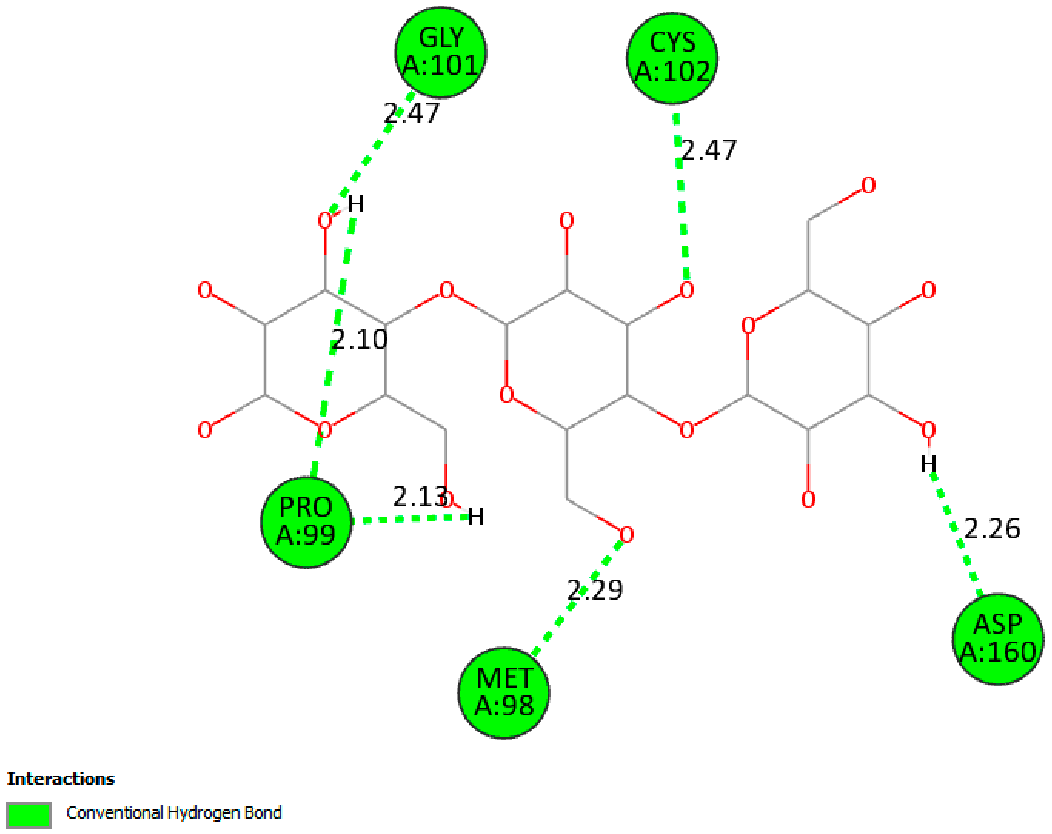

Figure S27. 2D molecular docking interaction diagram between EGFR and dextrin.

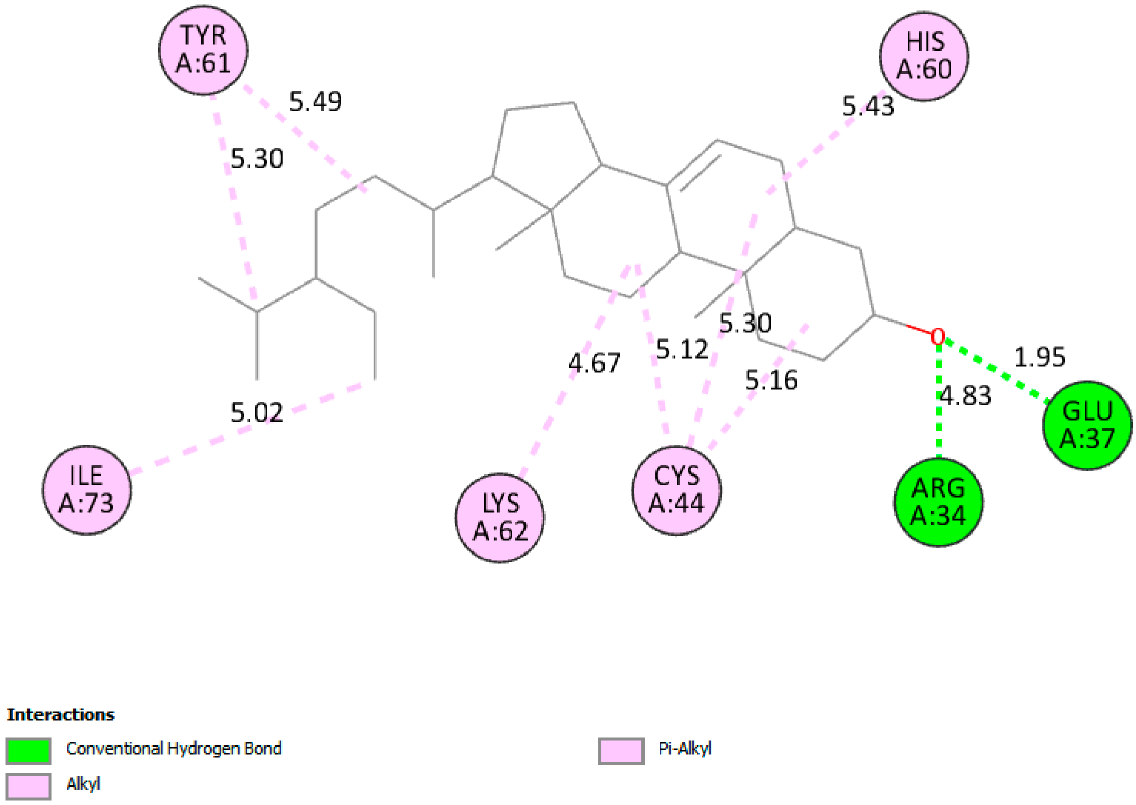

Figure S28. 2D molecular docking interaction diagram between SRC and diosgenin tetraglycoside.

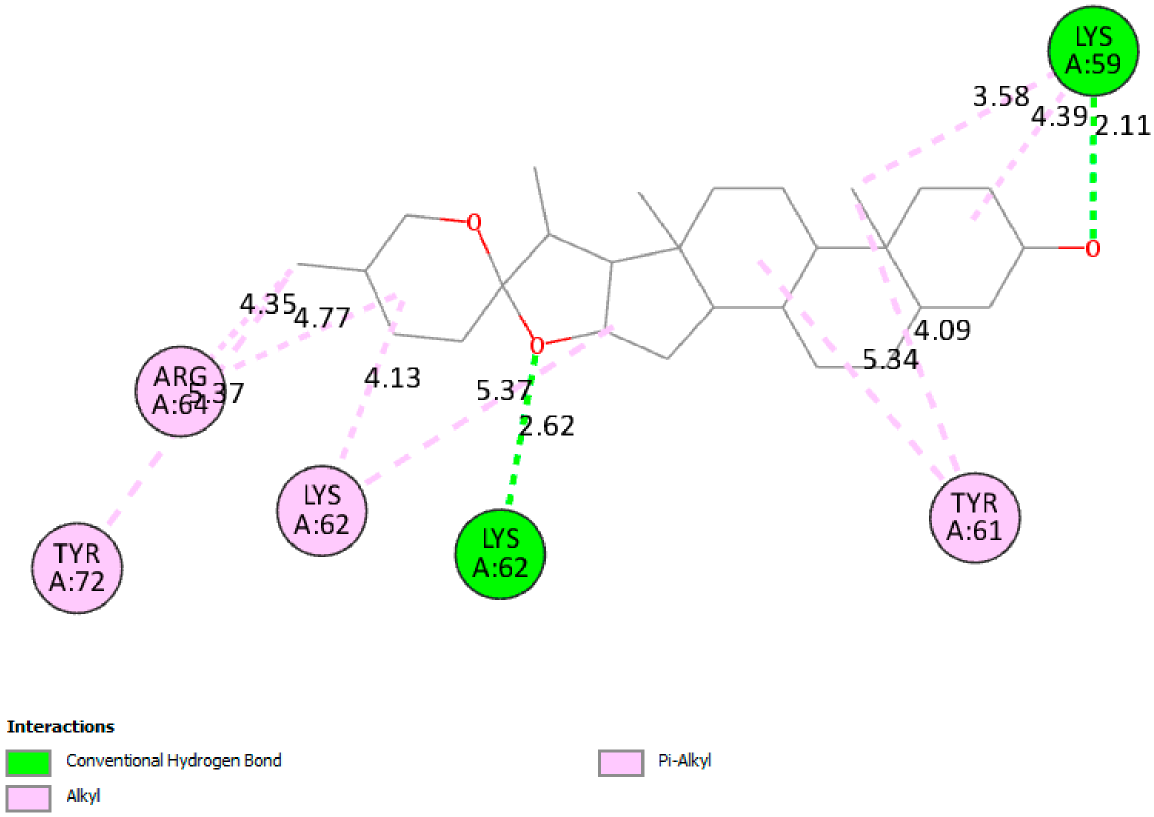

Figure S29. 2D molecular docking interaction diagram between SRC and spirostanol.

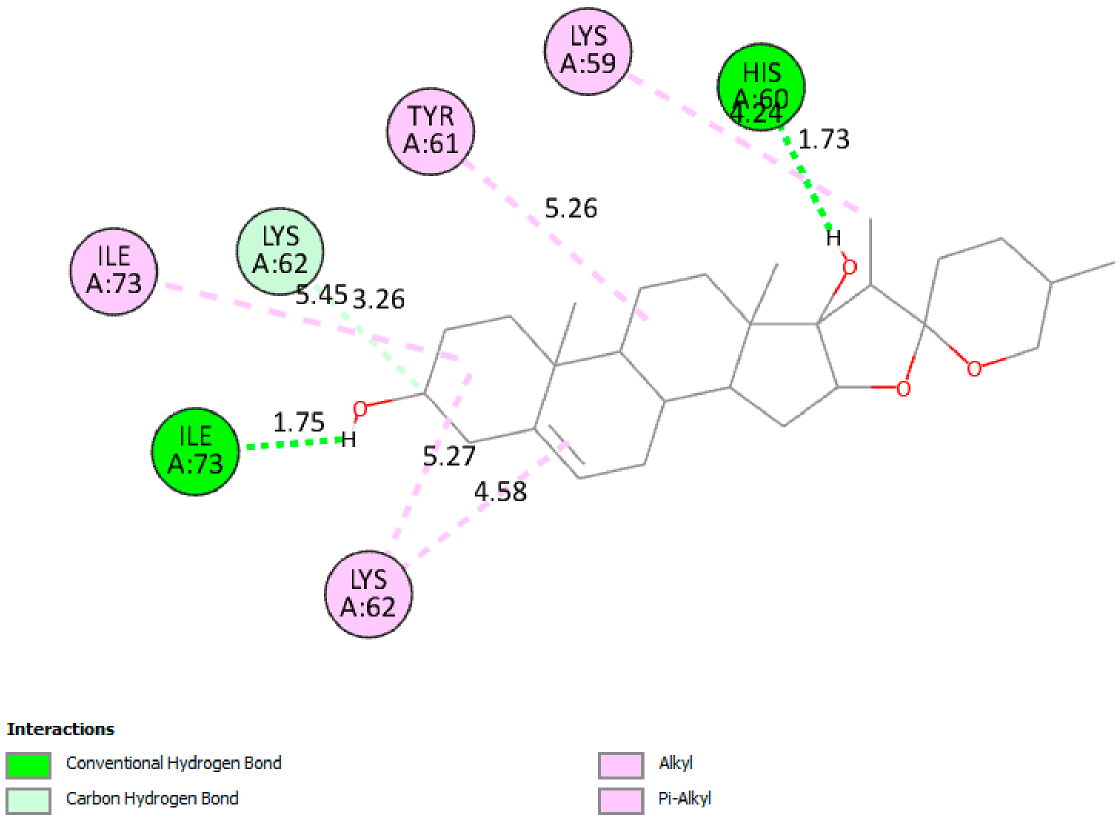

Figure S30. 2D molecular docking interaction diagram between SRC and pennogenin.

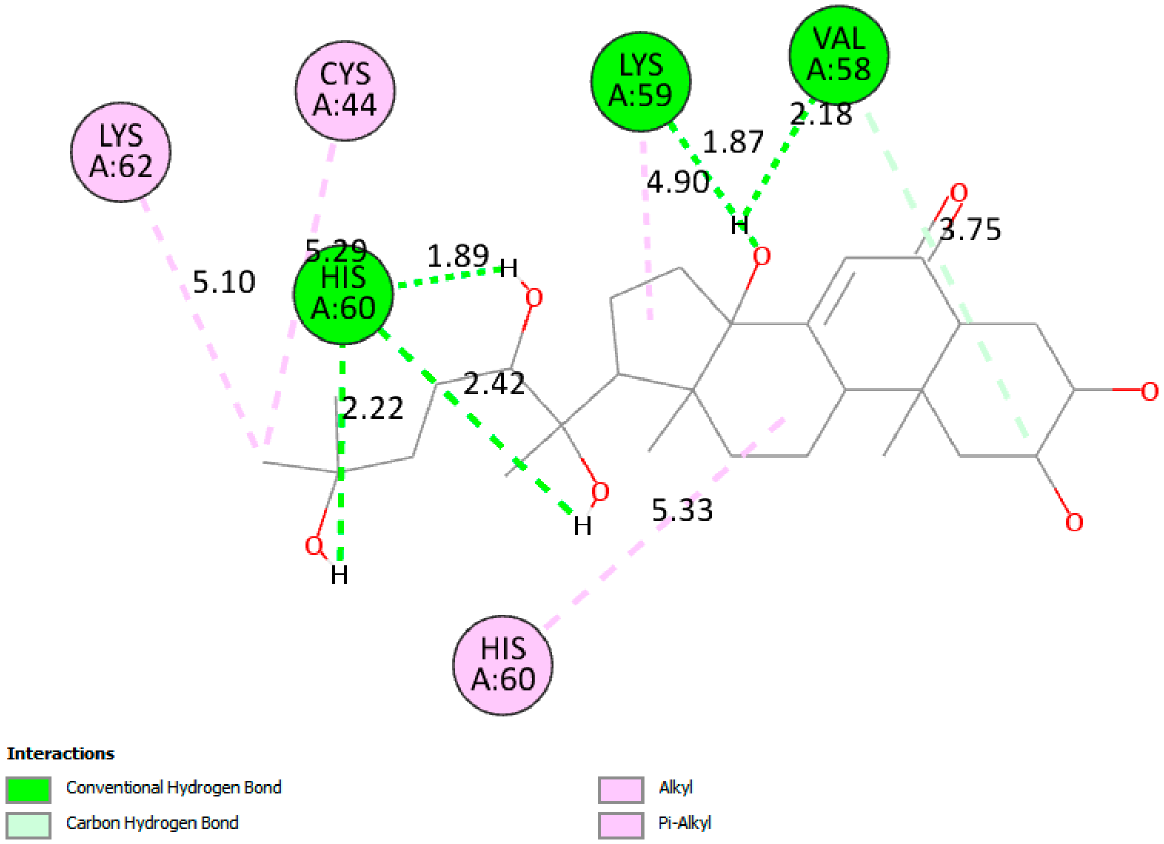

Figure S31. 2D molecular docking interaction diagram between SRC and 20-hydroxyecdysone.

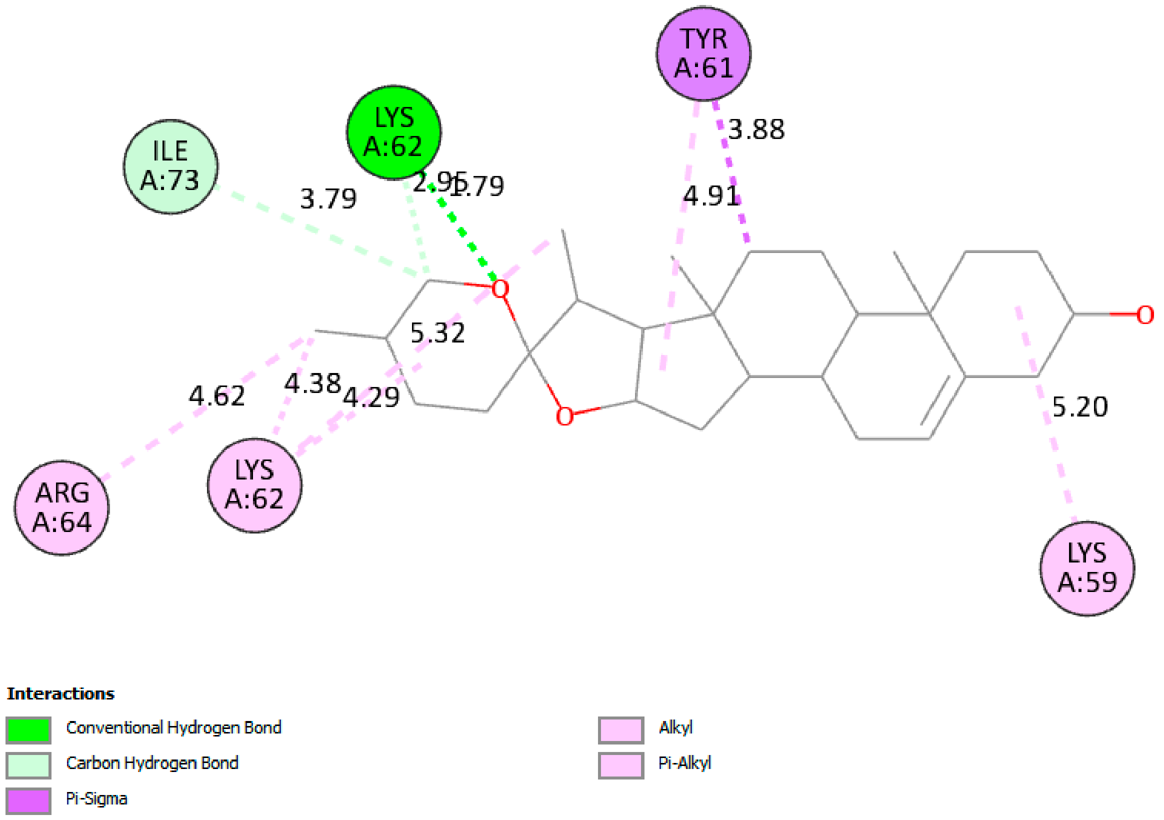

Figure S32. 2D molecular docking interaction diagram between SRC and diosgenin.

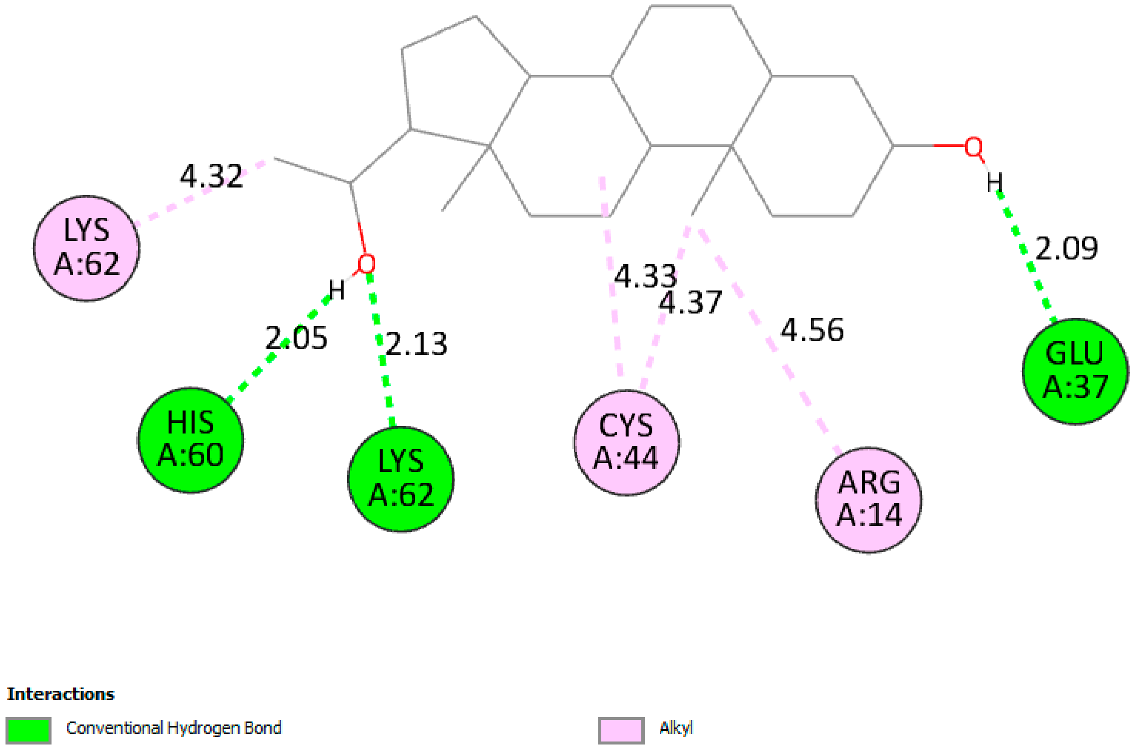

Figure S33. 2D molecular docking interaction diagram between SRC and pregnane-3,20-diol.

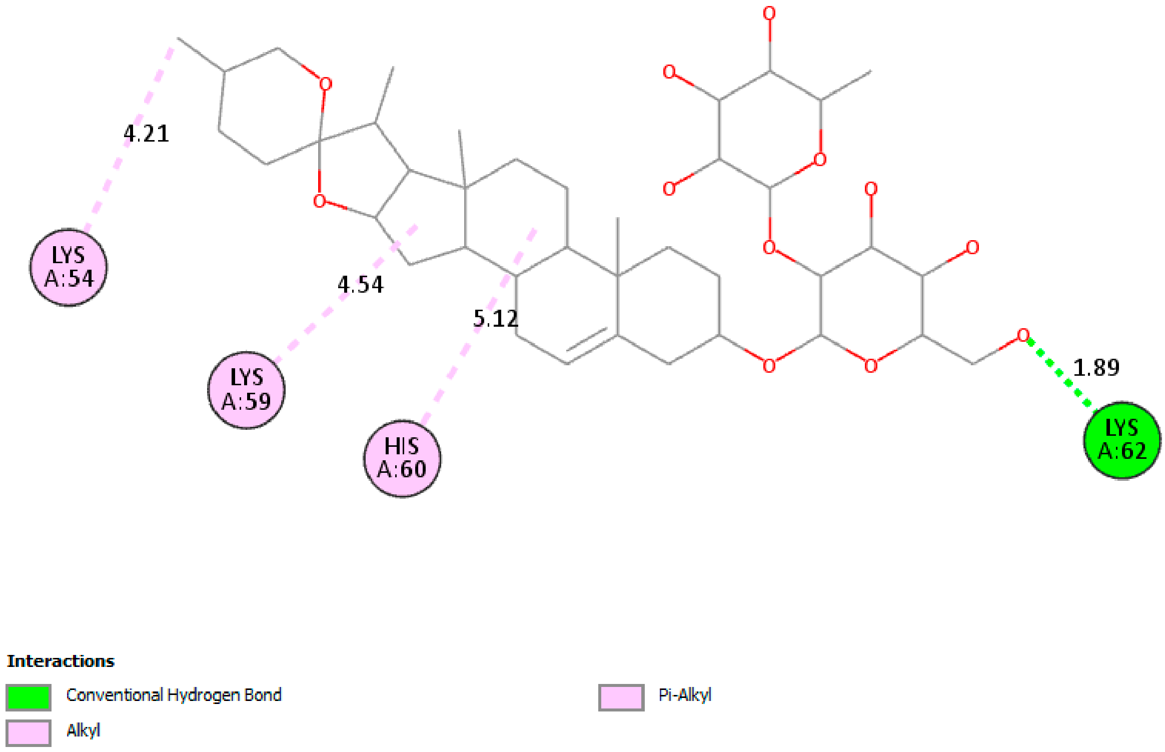

Figure S34. 2D molecular docking interaction diagram between SRC and prosapogenin A.

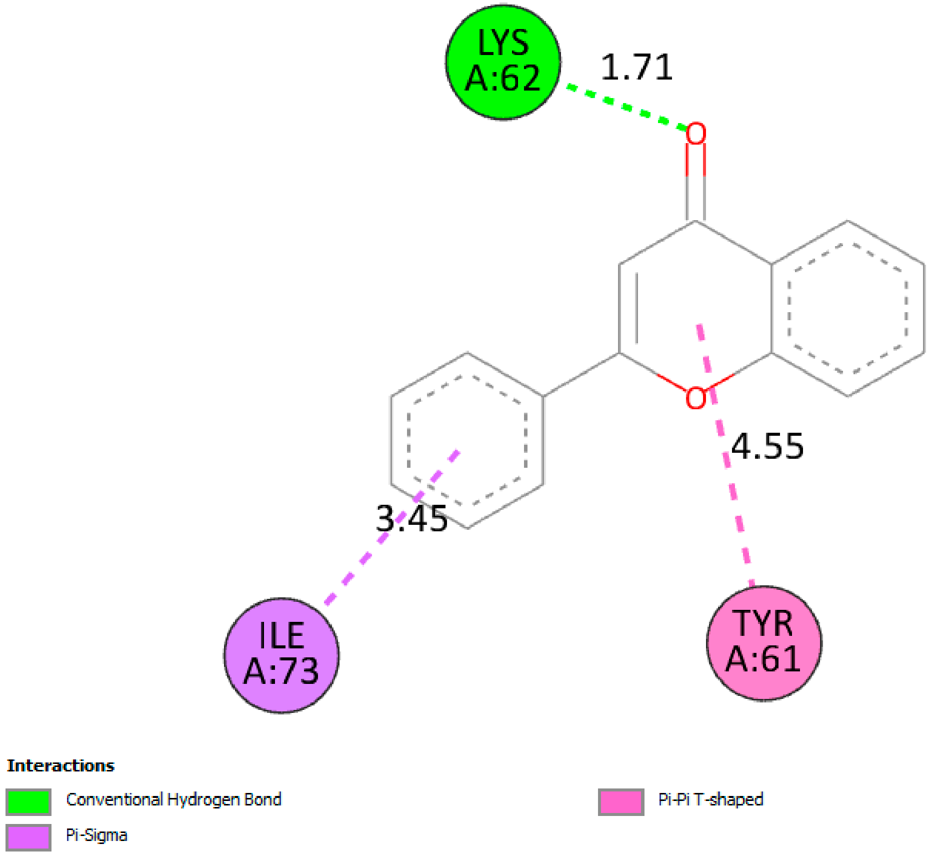

Figure S35. 2D molecular docking interaction diagram between SRC and flavone.

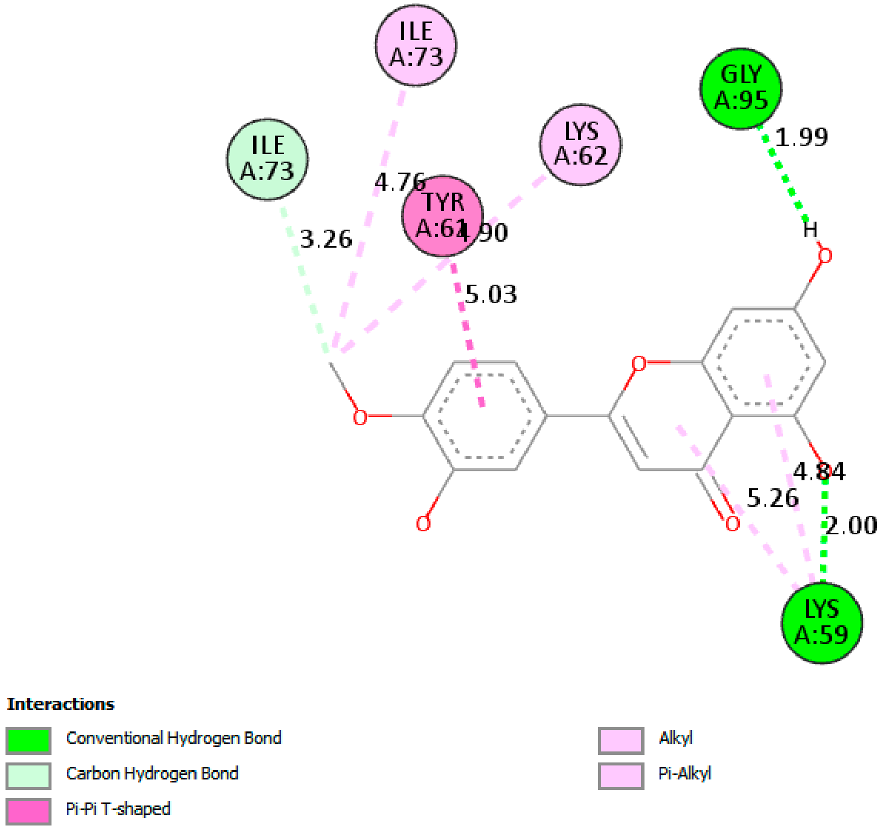

Figure S36. 2D molecular docking interaction diagram between SRC and diosmetin.

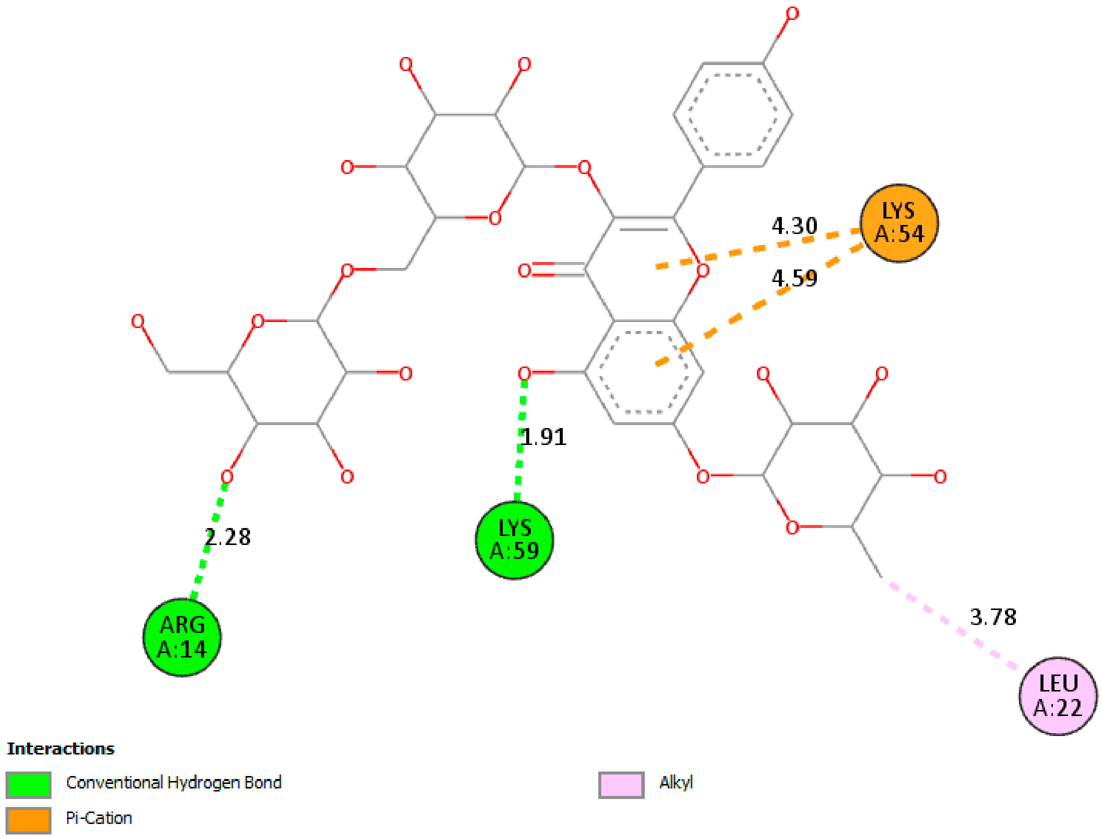

Figure S37. 2D molecular docking interaction diagram between SRC and kaempferol 3-gentiobioside-7-rhamnoside.

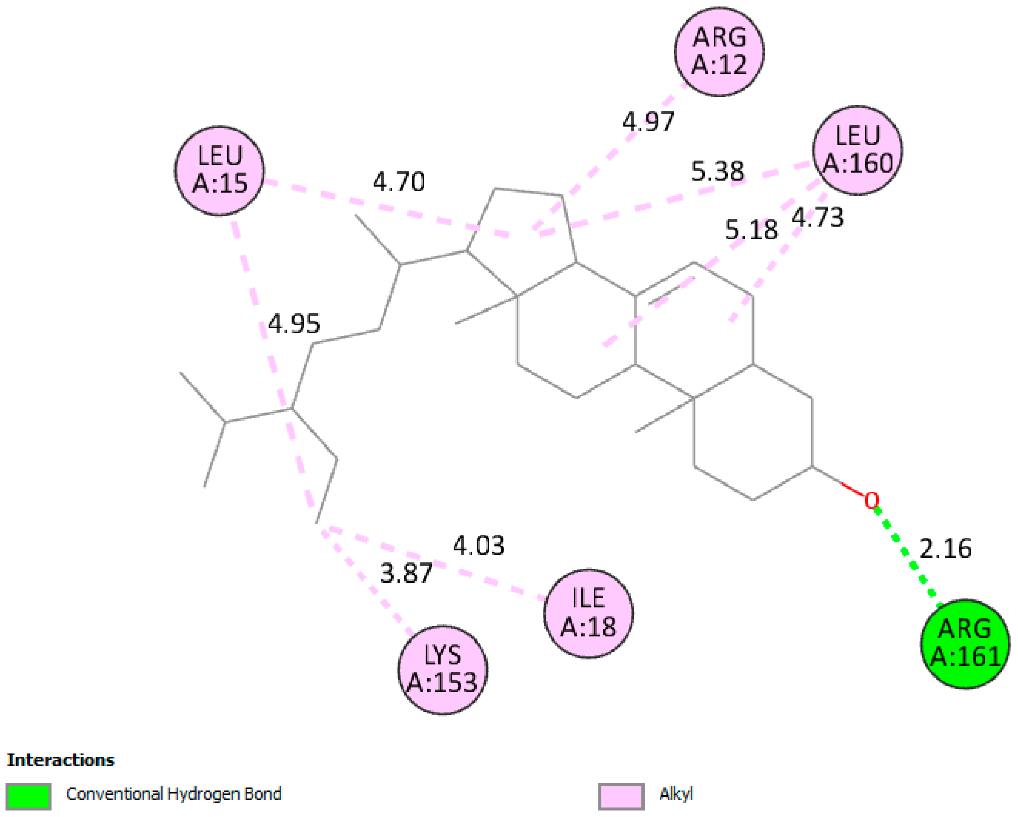

Figure S38. 2D molecular docking interaction diagram between IL-6 and diosgenin tetraglycoside.

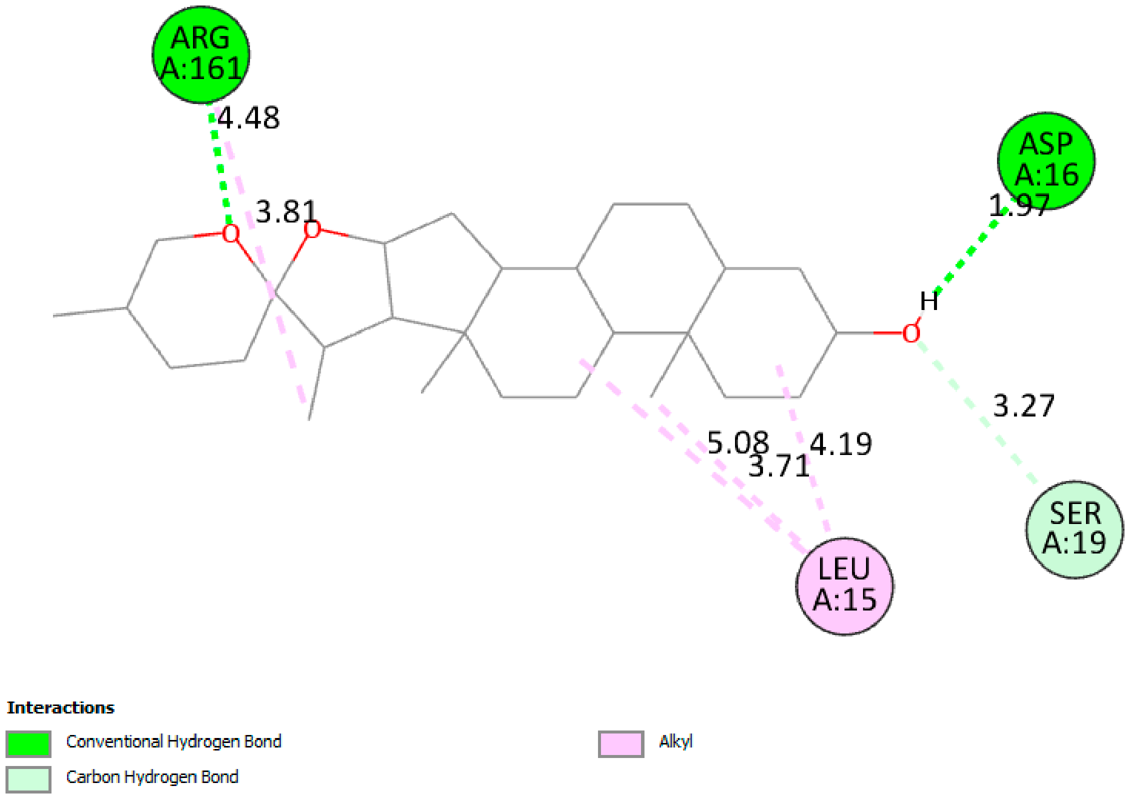

Figure S39. 2D molecular docking interaction diagram between IL-6 and spirostanol.

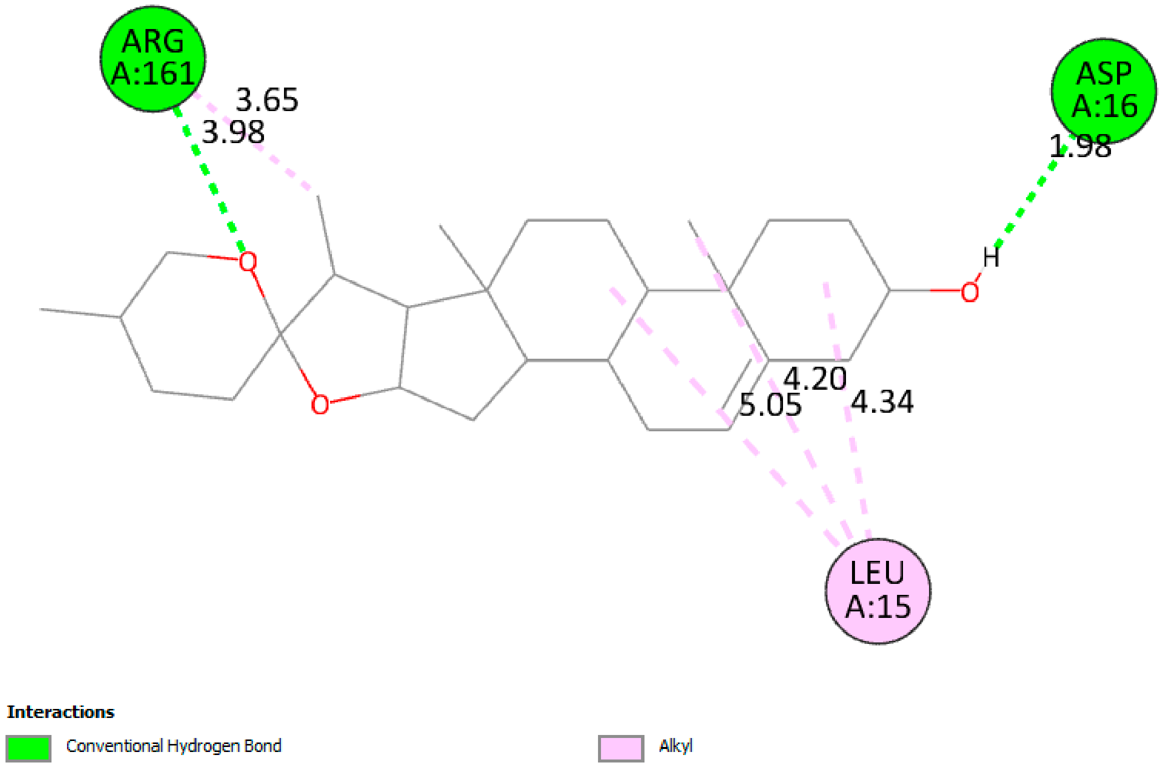

Figure S40. 2D molecular docking interaction diagram between IL-6 and diosgenin.

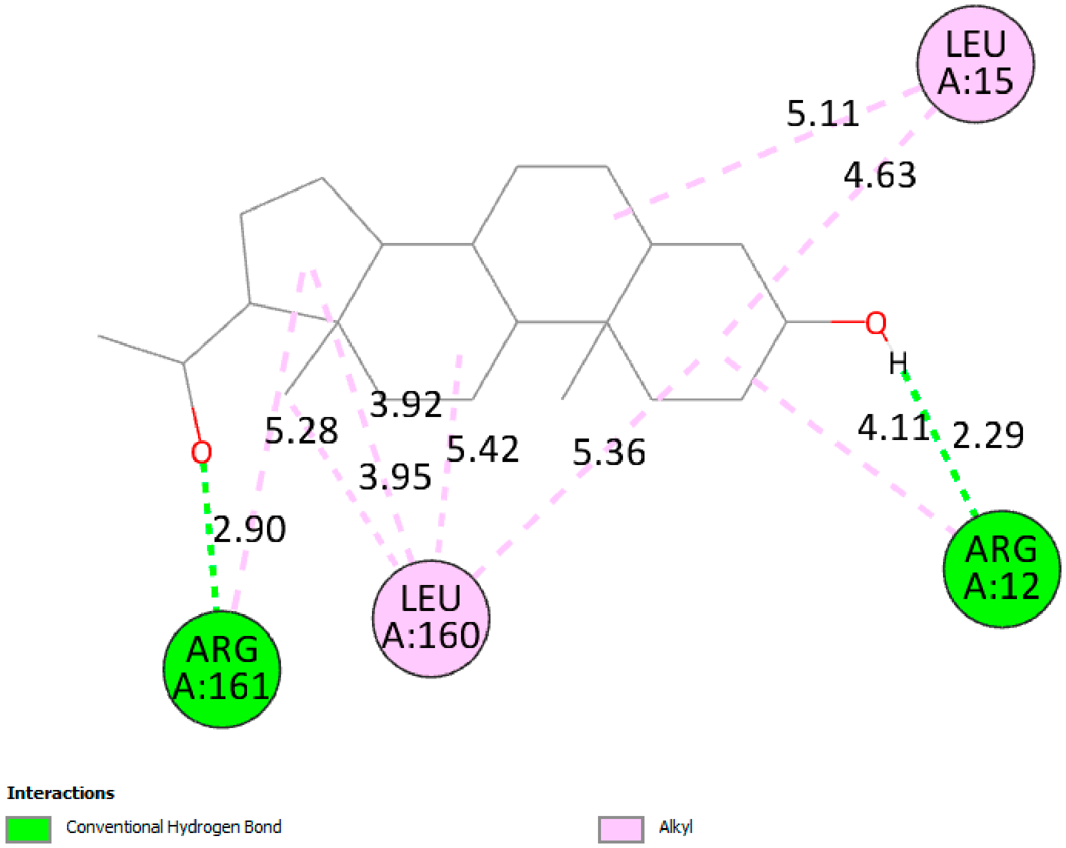

Figure S41. 2D molecular docking interaction diagram between IL-6 and pregnane-3,20-diol.

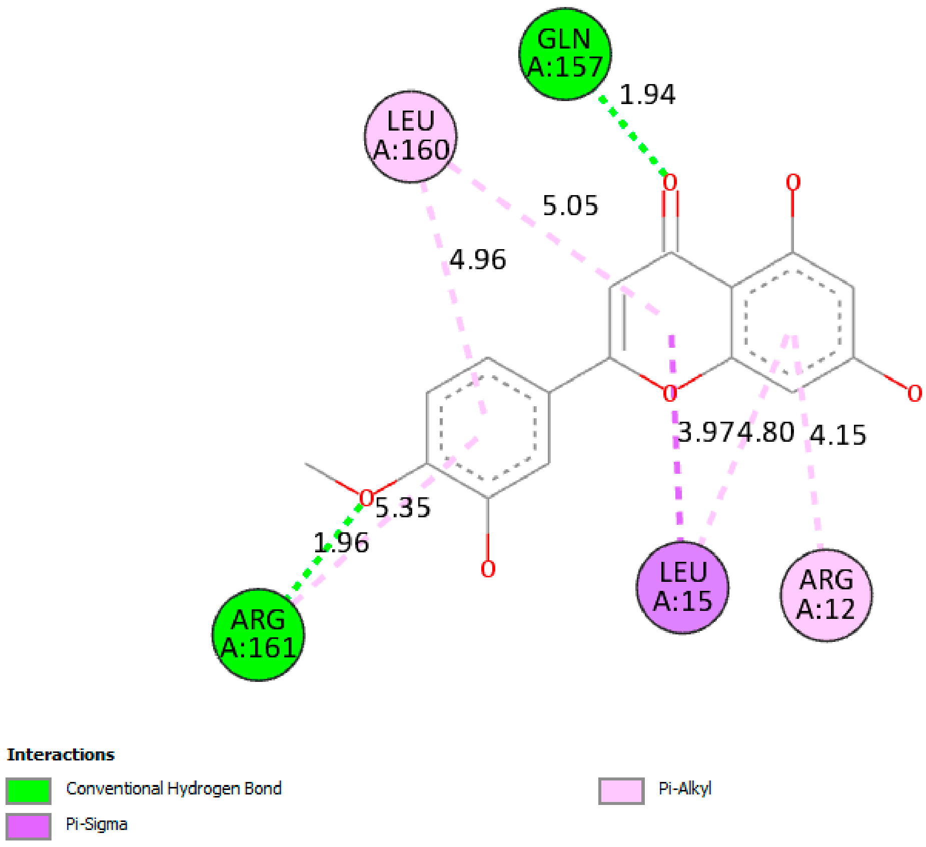

Figure S42. 2D molecular docking interaction diagram between IL-6 and diosmetin.

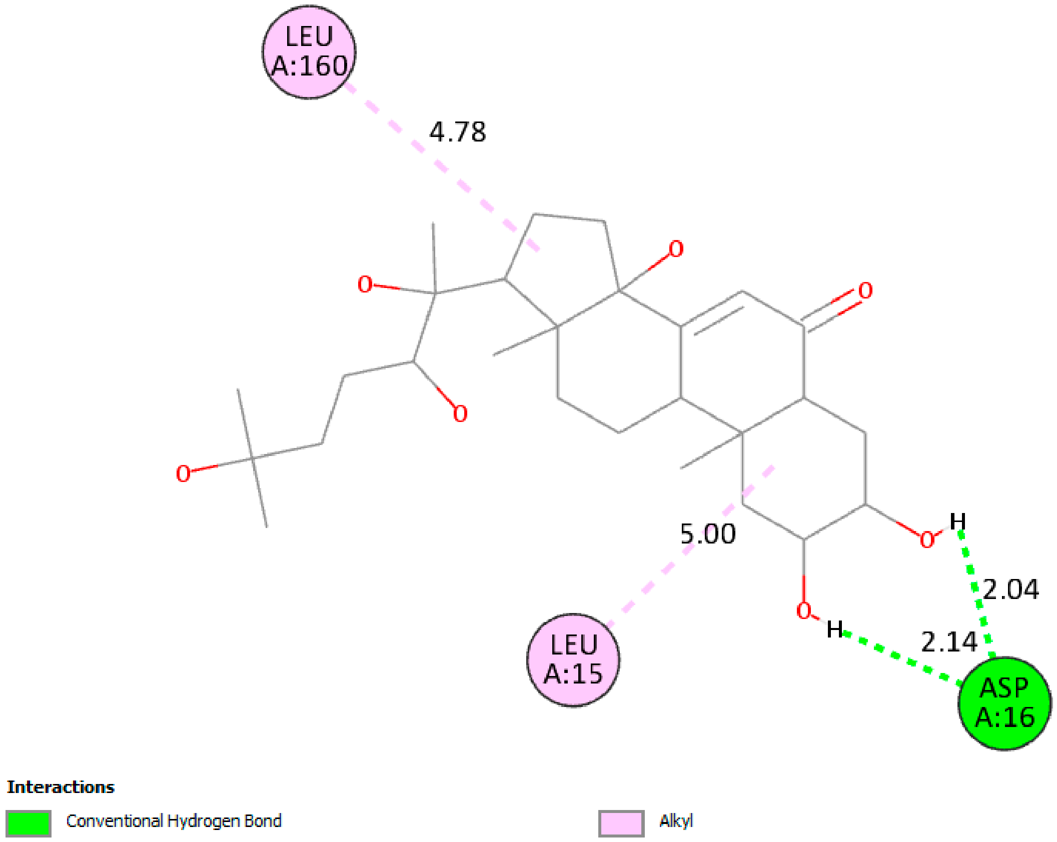

Figure S43. 2D molecular docking interaction diagram between IL-6 and 20-hydroxyecdysone.

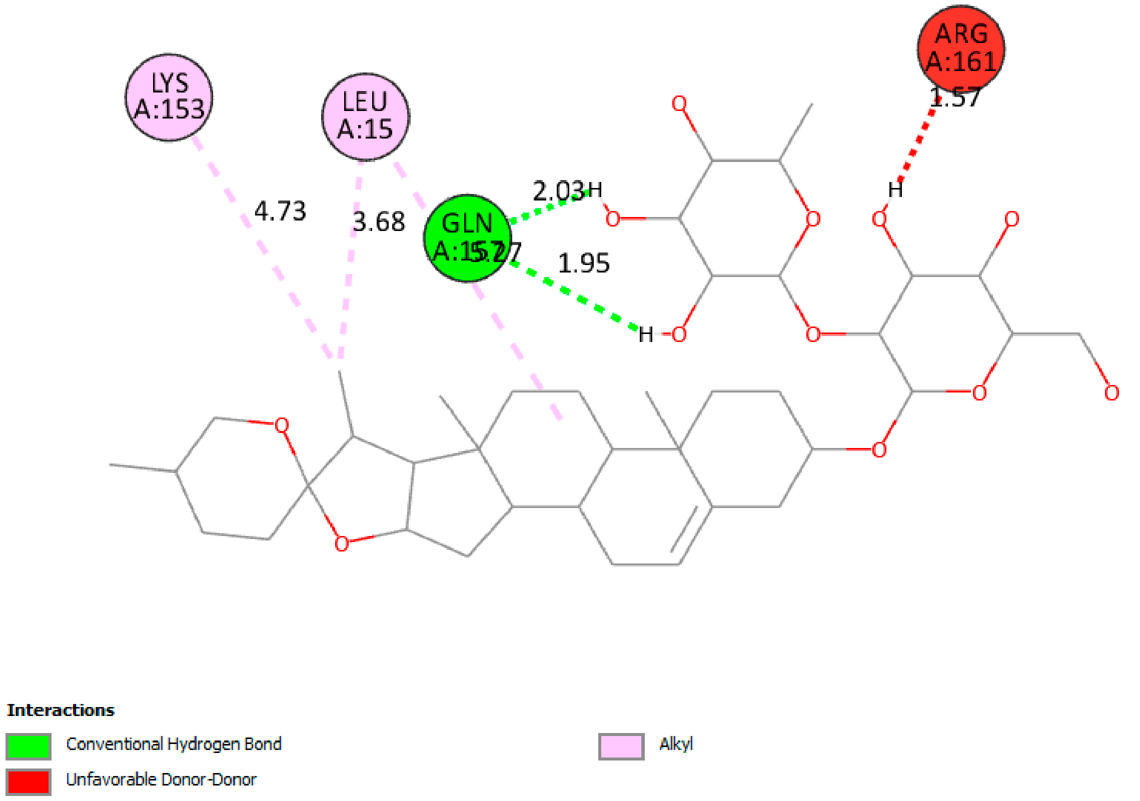

Figure S44. 2D molecular docking interaction diagram between IL-6 and prosapogenin A.

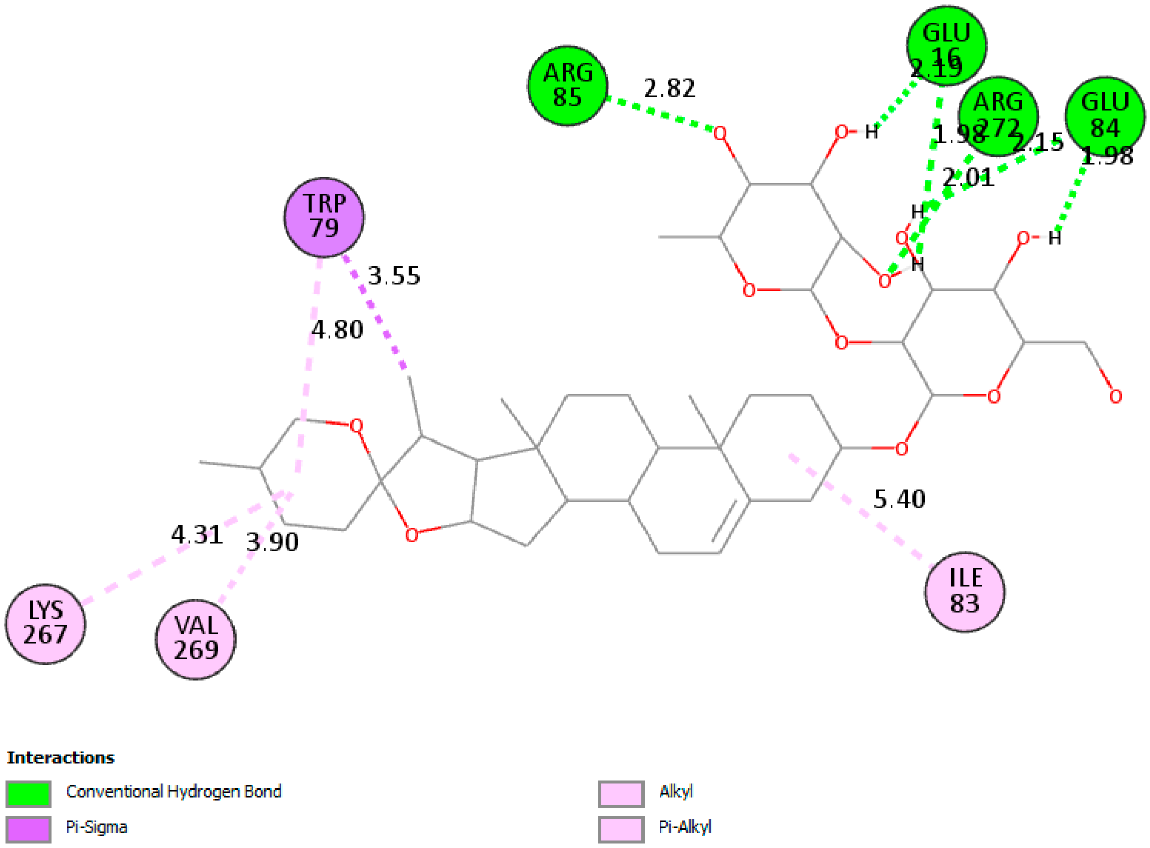

Figure S45. 2D molecular docking interaction diagram between AKT1 and prosapogenin A.

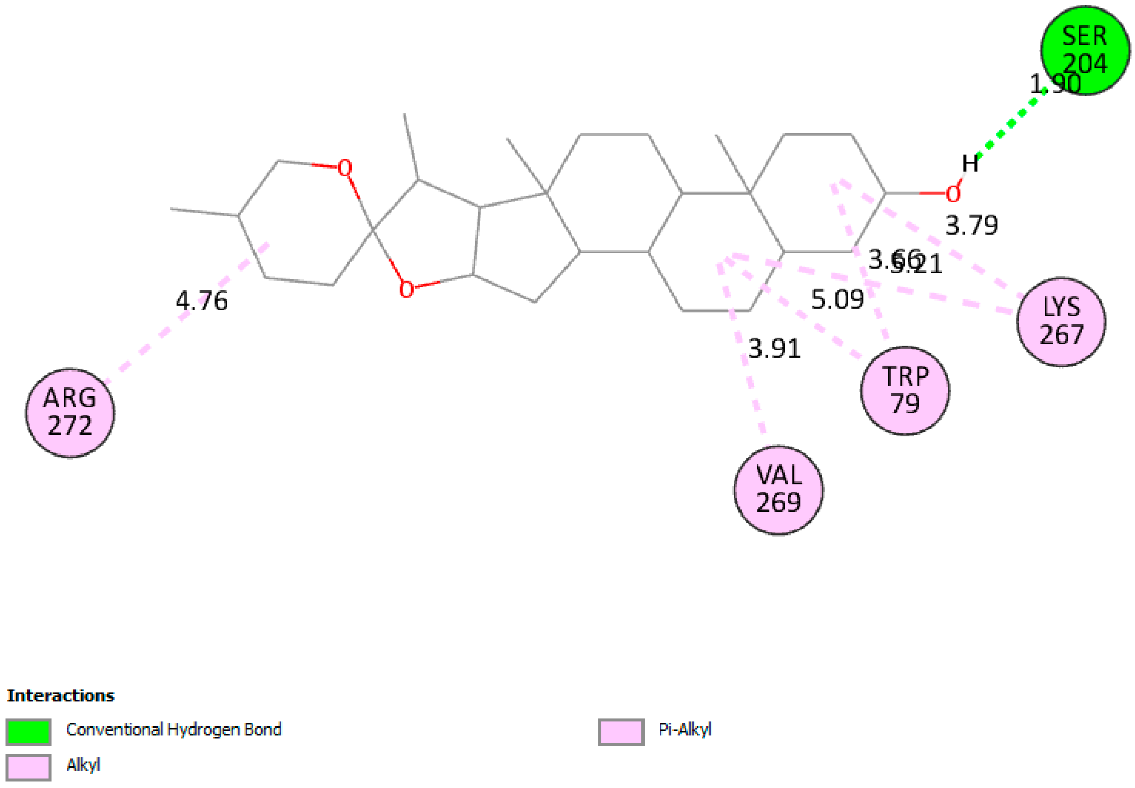

Figure S46. 2D molecular docking interaction diagram between AKT1 and spirostanol.

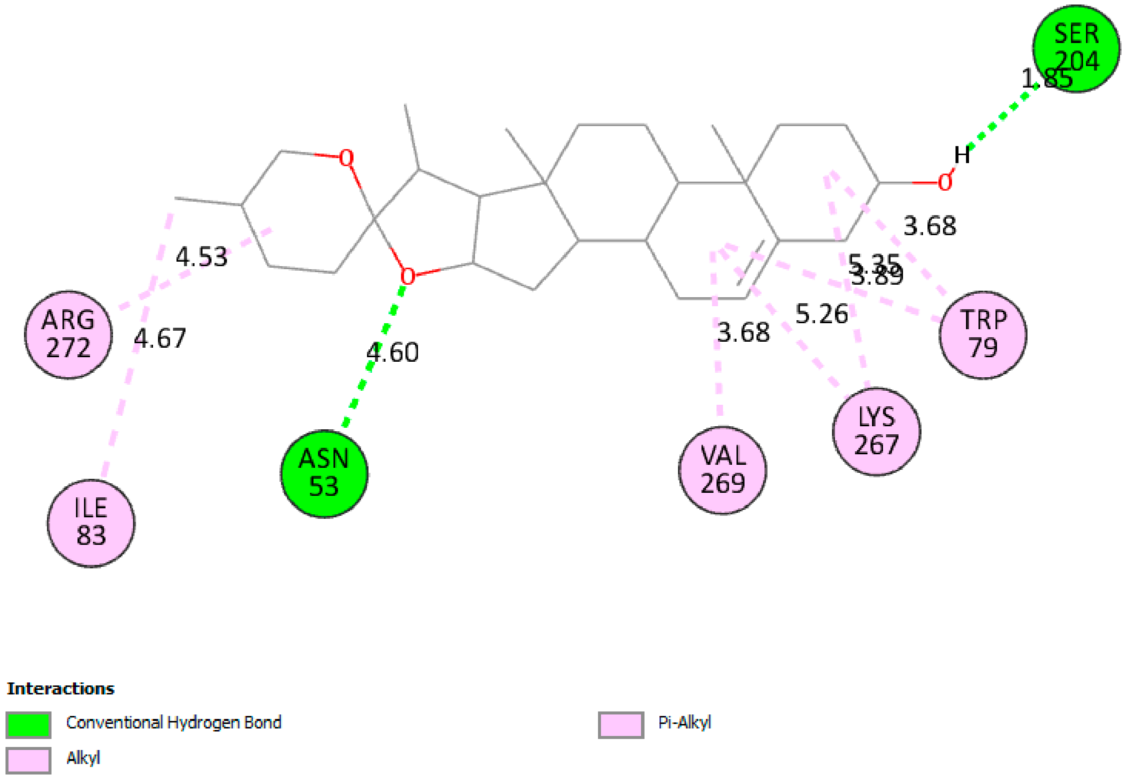

Figure S47. 2D molecular docking interaction diagram between AKT1 and diosgenin.

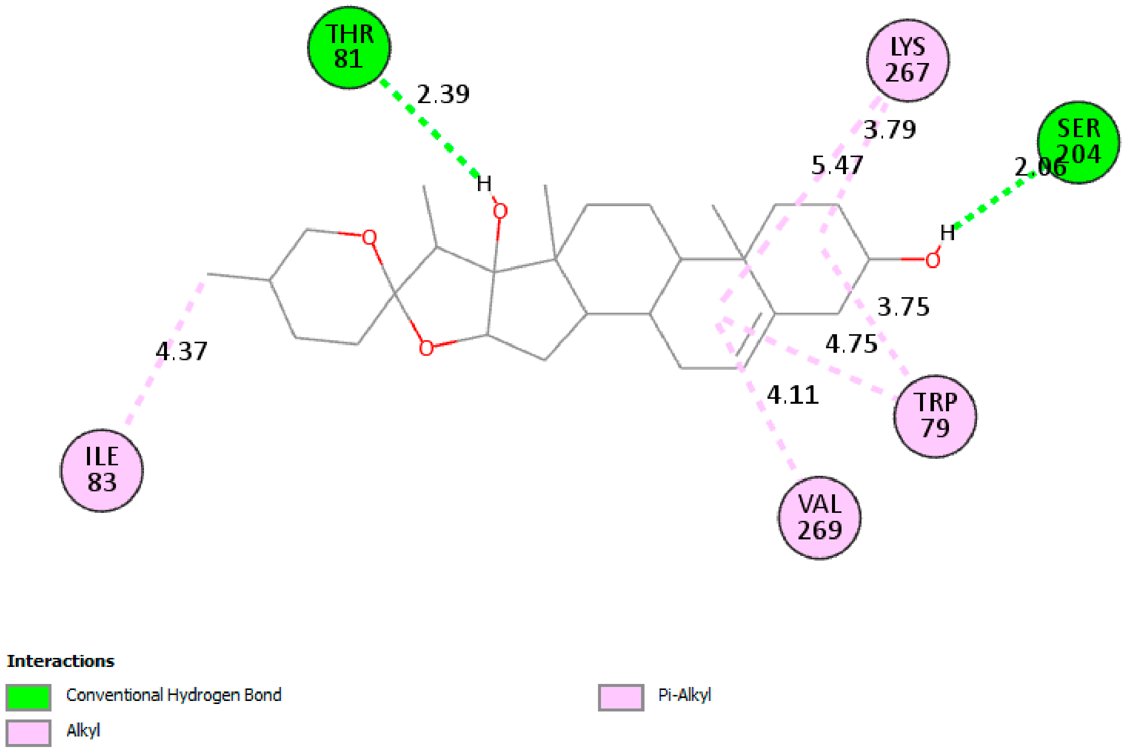

Figure S48. 2D molecular docking interaction diagram between AKT1 and pennogenin.

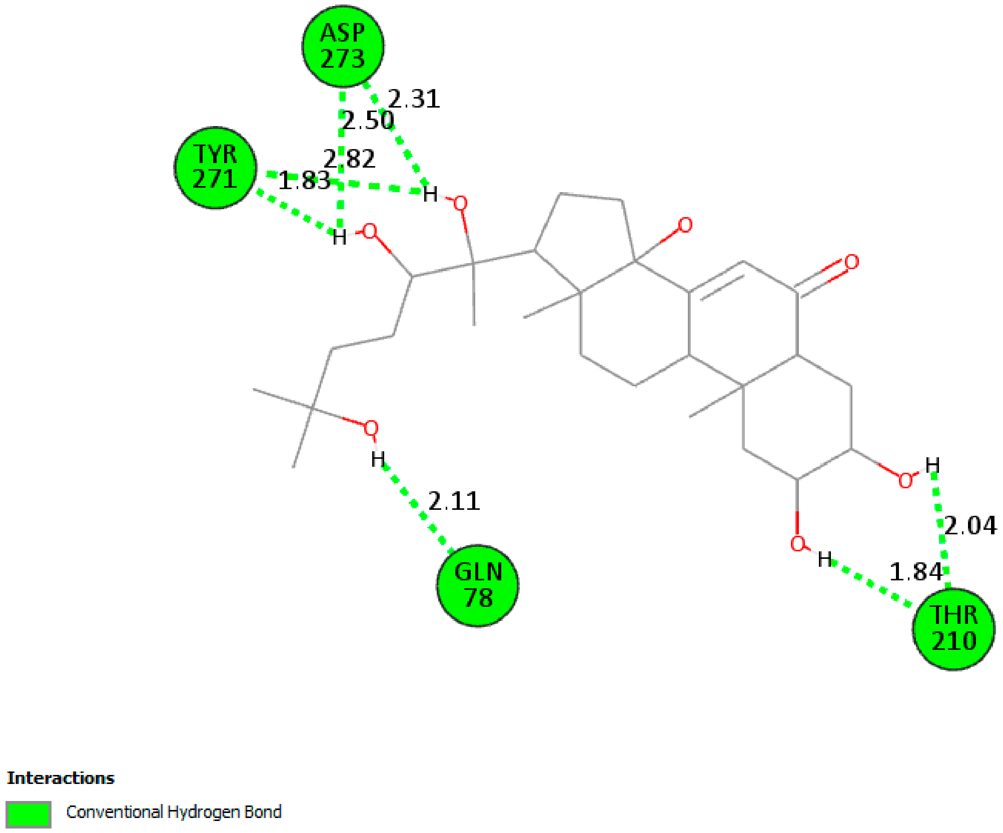

Figure S49. 2D molecular docking interaction diagram between AKT1 and 20-hydroxyecdysone.

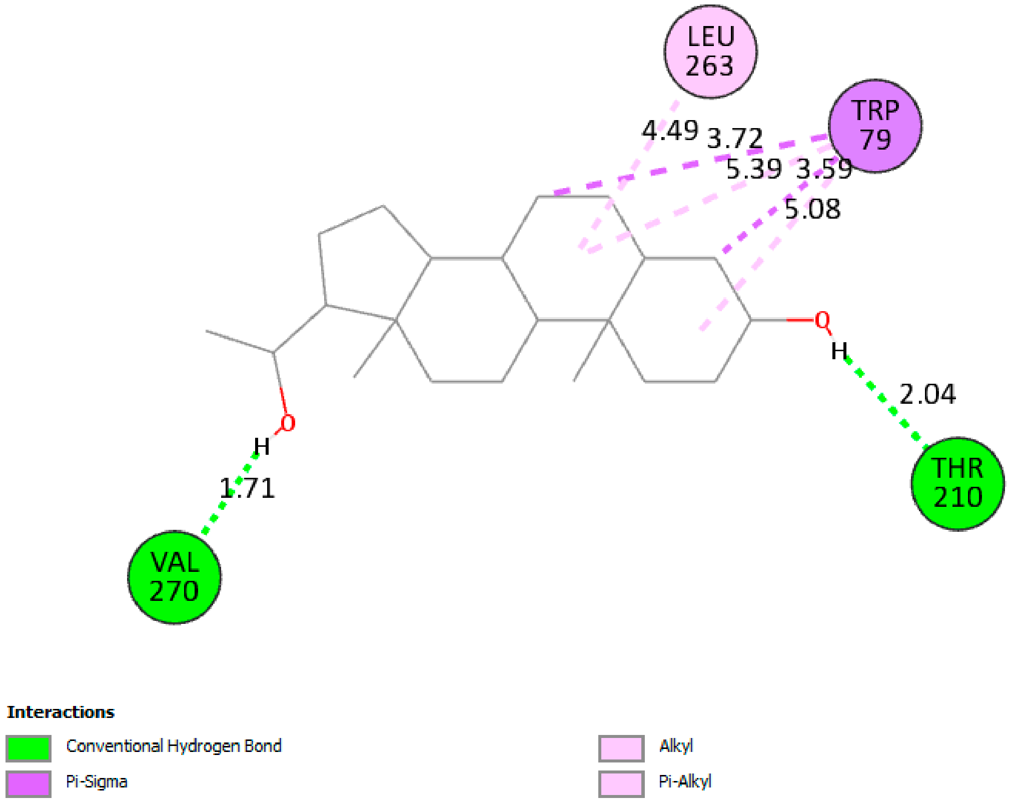

Figure S50. 2D molecular docking interaction diagram between AKT1 and pregnane-3,20-diol.

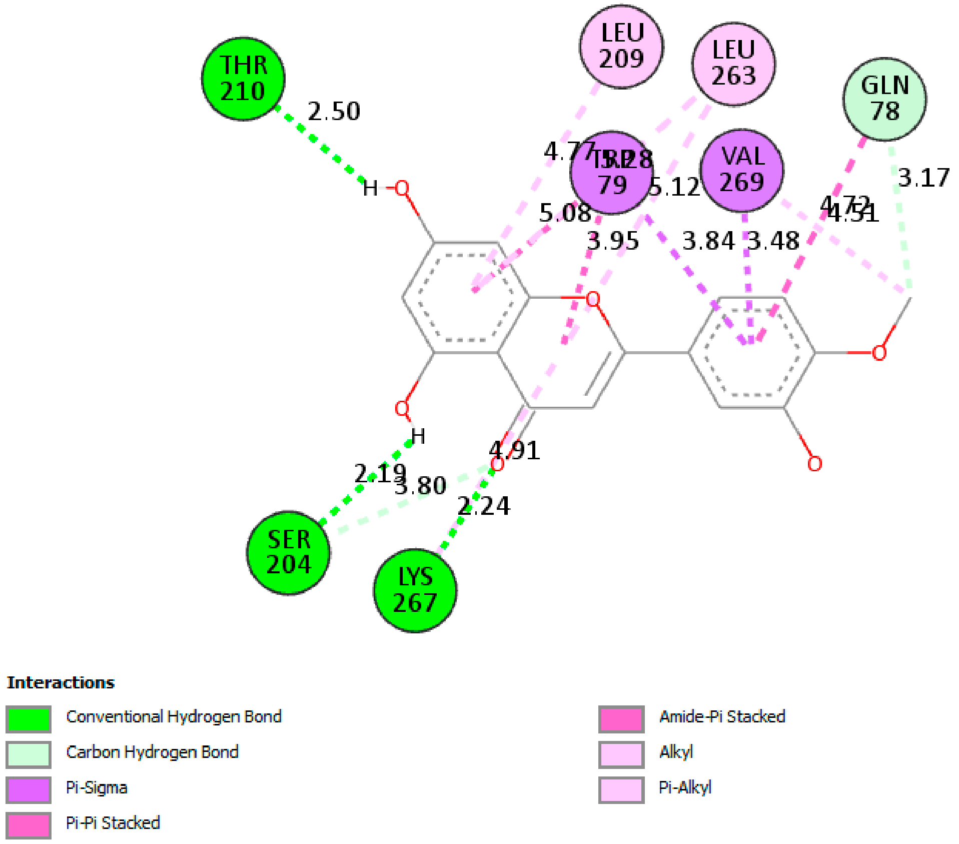

Figure S51. 2D molecular docking interaction diagram between AKT1 and diosmetin.

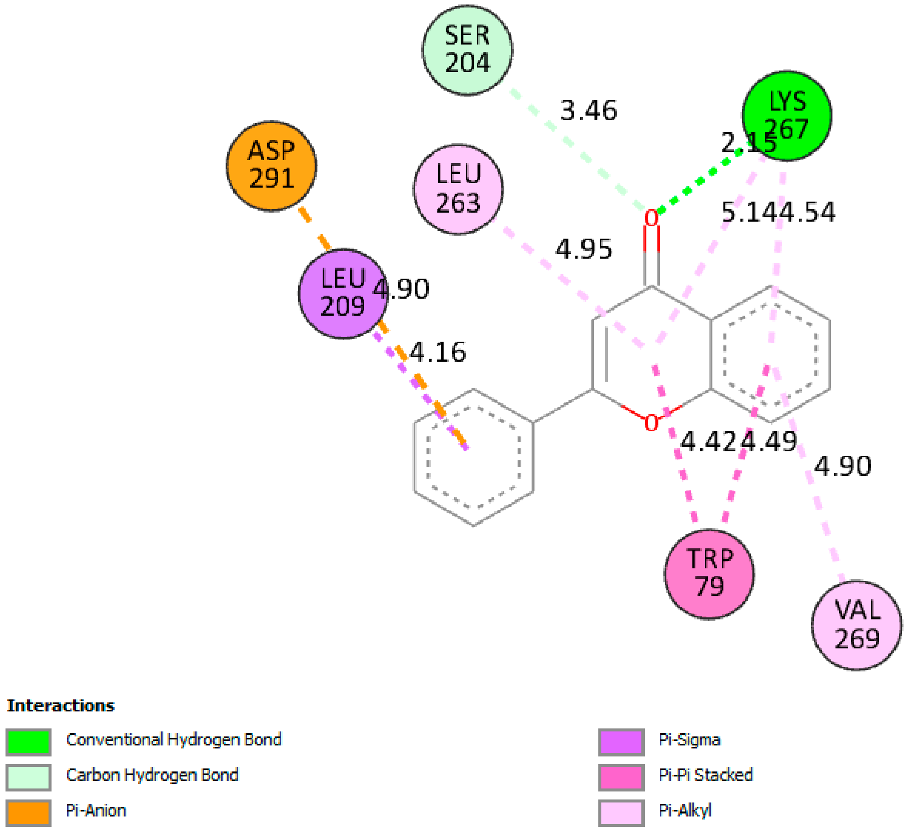

Figure S52. 2D molecular docking interaction diagram between AKT1 and flavone.

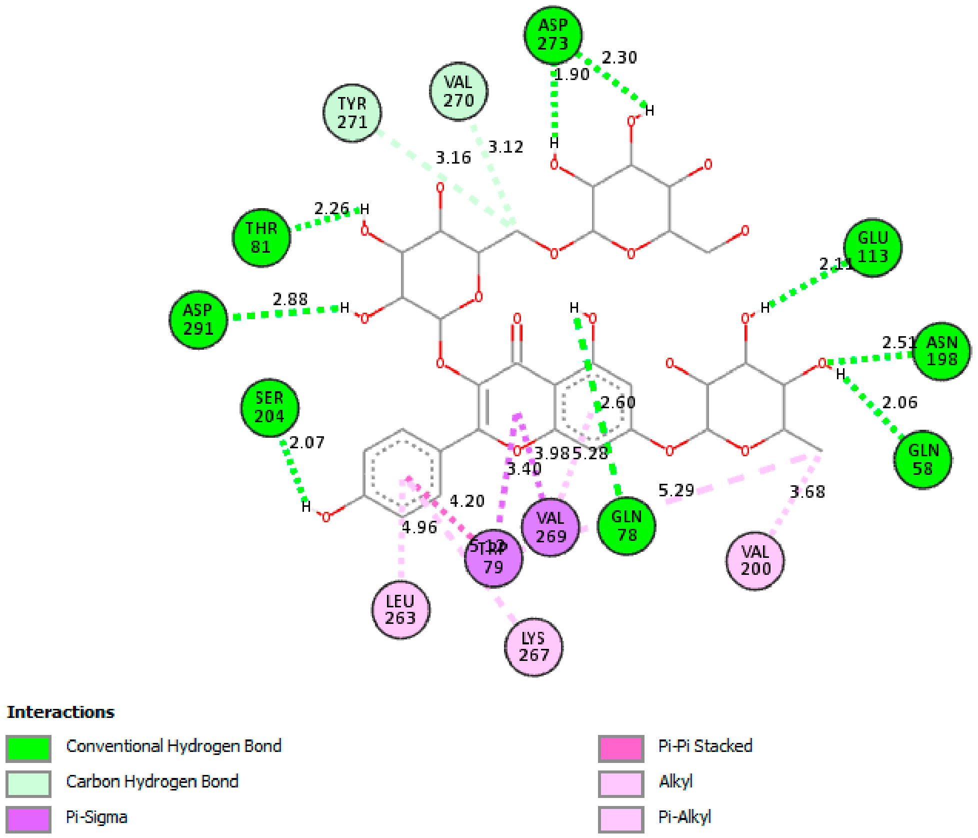

Figure S53. 2D molecular docking interaction diagram between AKT1 and kaempferol 3-gentiobioside-7-rhamnoside.

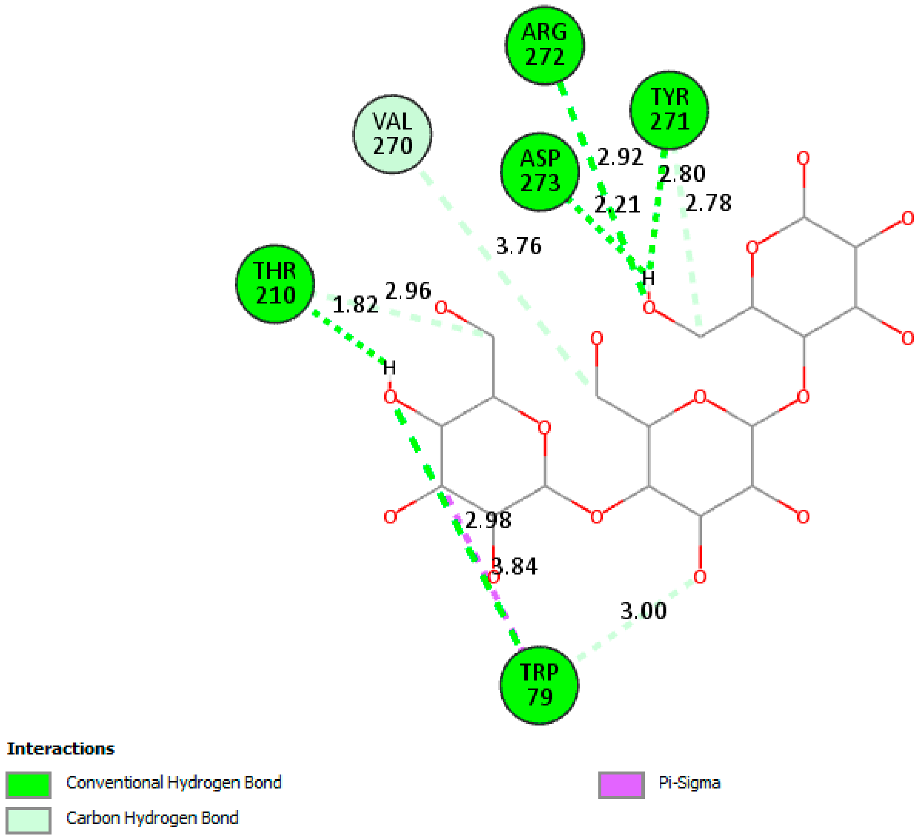

Figure S54. 2D molecular docking interaction diagram between AKT1 and dextrin.

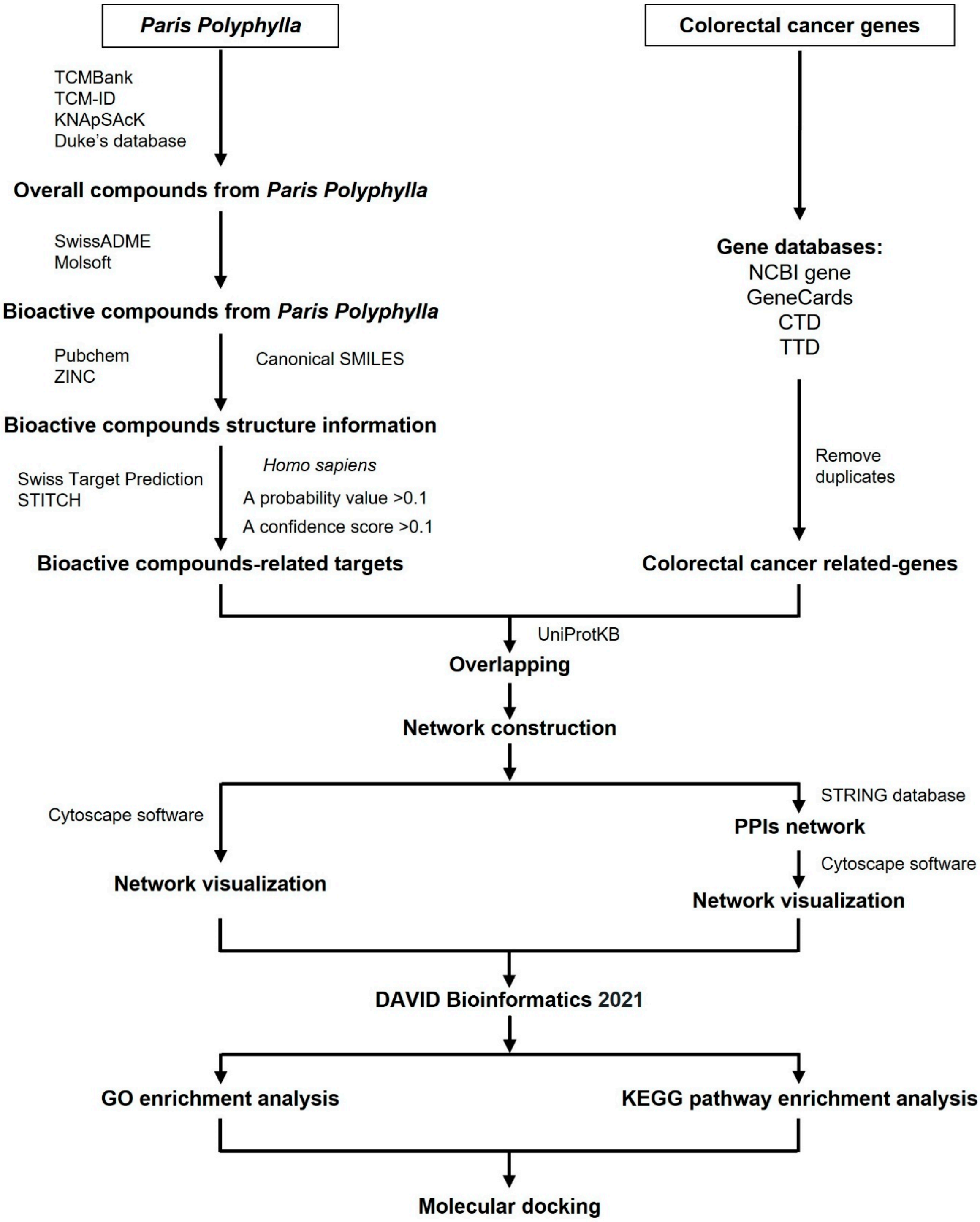

Figure S55. Workflow of the network pharmacology analysis of *Paris polyphylla* against colorectal cancer.

Supplementary Table

Table S1. List of reported colorectal cancer-related genes.

Table S2. Degree centrality analysis of the compound–target interaction network of *Paris polyphylla* in colorectal cancer.

Table S3. Protein–protein interaction (PPI) network data, including degree, betweenness centrality, and closeness centrality.

Table S4. Data of genes target and KEGG pathway network construction.

Table S1. List of reported colorectal cancer-related genes.

No.	Symbol	Uniprot Name	Database
1	TP53	P53_HUMAN	NCBI
2	TGFB1	TGFB1_HUMAN	NCBI
3	BRAF	BRAF_HUMAN	NCBI
4	AKT1	AKT1_HUMAN	NCBI
5	CTNNB1	CTNB1_HUMAN	NCBI
6	MYC	MYC_HUMAN	NCBI
7	EGFR	EGFR_HUMAN	NCBI
8	CDH1	CADH1_HUMAN	NCBI
9	SMAD7	SMAD7_HUMAN	NCBI
10	TNF	TNFA_HUMAN	NCBI
11	DCC	DCC_HUMAN	NCBI
12	IL6	IL6_HUMAN	NCBI
13	VEGFA	VEGFA_HUMAN	NCBI
14	CCND1	CCND1_HUMAN	NCBI
15	PIK3CA	PK3CA_HUMAN	NCBI
16	CDKN1A	CDN1A_HUMAN	NCBI
17	MTHFR	MTHR_HUMAN	NCBI
18	TLR2	TLR2_HUMAN	NCBI
19	APOE	APOE_HUMAN	NCBI
20	HIF1A	HIF1A_HUMAN	NCBI
21	ERBB2	ERBB2_HUMAN	NCBI
22	IL10	IL10_HUMAN	NCBI
23	STAT3	STAT3_HUMAN	NCBI
24	BRCA1	BRCA1_HUMAN	NCBI
25	SRC	SRC_HUMAN	NCBI
26	KRAS	RASK_HUMAN	NCBI
27	MLH1	MLH1_HUMAN	NCBI
28	VDR	VDR_HUMAN	NCBI
29	MMP9	MMP9_HUMAN	NCBI
30	CD274	PD1L1_HUMAN	NCBI
31	CRP	CRP_HUMAN	NCBI
32	ADIPOQ	ADIPO_HUMAN	NCBI
33	APC	APC_HUMAN	NCBI
34	ESR1	ESR1_HUMAN	NCBI
35	ABCB1	MDR1_HUMAN	NCBI
36	NFKB1	NFKB1_HUMAN	NCBI
37	IL1B	IL1B_HUMAN	NCBI
38	PTEN	PTEN_HUMAN	NCBI
39	CDKN2A	CDN2A_HUMAN	NCBI

No.	Symbol	Uniprot Name	Database
**40**	CDKN2A	ARF_HUMAN	NCBI
**41**	TLR4	TLR4_HUMAN	NCBI
**42**	BAX	BAX_HUMAN	NCBI
**43**	PTGS2	PGH2_HUMAN	NCBI
**44**	TCF7L2	TF7L2_HUMAN	NCBI
**45**	TERT	TERT_HUMAN	NCBI
**46**	CXCL8	IL8_HUMAN	NCBI
**47**	GATA3	GATA3_HUMAN	NCBI
**48**	MTOR	MTOR_HUMAN	NCBI
**49**	PPARG	PPARG_HUMAN	NCBI
**50**	NOS3	NOS3_HUMAN	NCBI
**51**	AURKA	AURKA_HUMAN	NCBI
**52**	IGF1	IGF1_HUMAN	NCBI
**53**	EP300	EP300_HUMAN	NCBI
**54**	APP	A4_HUMAN	NCBI
**55**	LEP	LEP_HUMAN	NCBI
**56**	BCL2	BCL2_HUMAN	NCBI
**57**	BRCA2	BRCA2_HUMAN	NCBI
**58**	CXCR4	CXCR4_HUMAN	NCBI
**59**	NFE2L2	NF2L2_HUMAN	NCBI
**60**	MDM2	MDM2_HUMAN	NCBI
**61**	GSTM1	GSTM1_HUMAN	NCBI
**62**	BDNF	BDNF_HUMAN	NCBI
**63**	IL17A	IL17_HUMAN	NCBI
**64**	MMP2	MMP2_HUMAN	NCBI
**65**	SIRT1	SIR1_HUMAN	NCBI
**66**	ACE	ACE_HUMAN	NCBI
**67**	TPX2	TPX2_HUMAN	NCBI
**68**	AR	ANDR_HUMAN	NCBI
**69**	COMT	COMT_HUMAN	NCBI
**70**	JAK2	JAK2_HUMAN	NCBI
**71**	MAPK1	MK01_HUMAN	NCBI
**72**	HMGB1	HMGB1_HUMAN	NCBI
**73**	NRAS	RASN_HUMAN	NCBI
**74**	CFTR	CFTR_HUMAN	NCBI
**75**	CCL2	CCL2_HUMAN	NCBI
**76**	CTLA4	CTLA4_HUMAN	NCBI
**77**	CD44	CD44_HUMAN	NCBI
**78**	BIRC5	BIRC5_HUMAN	NCBI
**79**	TGFBR2	TGFR2_HUMAN	NCBI
**80**	EZH2	EZH2_HUMAN	NCBI

No.	Symbol	Uniprot Name	Database
**81**	NOTCH1	NOTC1_HUMAN	NCBI
**82**	GSTT1	GSTT1_HUMAN	NCBI
**83**	MET	MET_HUMAN	NCBI
**84**	GSTP1	GSTP1_HUMAN	NCBI
**85**	CXCL12	SDF1_HUMAN	NCBI
**86**	IFNG	IFNG_HUMAN	NCBI
**87**	BMP2	BMP2_HUMAN	NCBI
**88**	FOXP3	FOXP3_HUMAN	NCBI
**89**	FGF23	FGF23_HUMAN	NCBI
**90**	PDCD1	PDCD1_HUMAN	NCBI
**91**	SPP1	OSTP_HUMAN	NCBI
**92**	YAP1	YAP1_HUMAN	NCBI
**93**	NLRP3	NLRP3_HUMAN	NCBI
**94**	BMP4	BMP4_HUMAN	NCBI
**95**	GREM1	GREM1_HUMAN	NCBI
**96**	RELA	TF65_HUMAN	NCBI
**97**	CCR5	CCR5_HUMAN	NCBI
**98**	XRCC1	XRCC1_HUMAN	NCBI
**99**	ESR2	ESR2_HUMAN	NCBI
**100**	GSK3B	GSK3B_HUMAN	NCBI
**101**	SLC6A4	SC6A4_HUMAN	NCBI
**102**	ATM	ATM_HUMAN	NCBI
**103**	PARP1	PARP1_HUMAN	NCBI
**104**	FGFR3	FGFR3_HUMAN	NCBI
**105**	IGF1R	IGF1R_HUMAN	NCBI
**106**	HLA-B	HLAB_HUMAN	NCBI
**107**	HMOX1	HMOX1_HUMAN	NCBI
**108**	TNFRSF1A	TNR1A_HUMAN	NCBI
**109**	FAS	TNR6_HUMAN	NCBI
**110**	ACE2	ACE2_HUMAN	NCBI
**111**	ITGB1	ITB1_HUMAN	NCBI
**112**	MUC1	MUC1_HUMAN	NCBI
**113**	SERPINE1	PAI1_HUMAN	NCBI
**114**	HLA-G	HLAG_HUMAN	NCBI
**115**	NOD2	NOD2_HUMAN	NCBI
**116**	ICAM1	ICAM1_HUMAN	NCBI
**117**	ABCG2	ABCG2_HUMAN	NCBI
**118**	RAC1	RAC1_HUMAN	NCBI
**119**	LCN2	NGAL_HUMAN	NCBI
**120**	CCND2	CCND2_HUMAN	NCBI
**121**	CYP1A1	CP1A1_HUMAN	NCBI

No.	Symbol	Uniprot Name	Database
**122**	ITGB3	ITB3_HUMAN	NCBI
**123**	SP1	SP1_HUMAN	NCBI
**124**	KDR	VGFR2_HUMAN	NCBI
**125**	MMP1	MMP1_HUMAN	NCBI
**126**	AGER	RAGE_HUMAN	NCBI
**127**	CDKN1B	CDN1B_HUMAN	NCBI
**128**	SOD1	SODC_HUMAN	NCBI
**129**	CAV1	CAV1_HUMAN	NCBI
**130**	TNFSF10	TNF10_HUMAN	NCBI
**131**	CD4	CD4_HUMAN	NCBI
**132**	MGMT	MGMT_HUMAN	NCBI
**133**	PTK2	FAK1_HUMAN	NCBI
**134**	FTO	FTO_HUMAN	NCBI
**135**	PON1	PON1_HUMAN	NCBI
**136**	HFE	HFE_HUMAN	NCBI
**137**	LGALS3	LEG3_HUMAN	NCBI
**138**	IL4	IL4_HUMAN	NCBI
**139**	KIT	KIT_HUMAN	NCBI
**140**	IL18	IL18_HUMAN	NCBI
**141**	RB1	RB_HUMAN	NCBI
**142**	UGT1A1	UD11_HUMAN	NCBI
**143**	CASP3	CASP3_HUMAN	NCBI
**144**	ALDH2	ALDH2_HUMAN	NCBI
**145**	MIF	MIF_HUMAN	NCBI
**146**	JUN	JUN_HUMAN	NCBI
**147**	MAPK14	MK14_HUMAN	NCBI
**148**	ABCC2	MRP2_HUMAN	NCBI
**149**	MKI67	KI67_HUMAN	NCBI
**150**	AGT	ANGT_HUMAN	NCBI
**151**	NR3C1	GCR_HUMAN	NCBI
**152**	CASP8	CASP8_HUMAN	NCBI
**153**	GDF15	GDF15_HUMAN	NCBI
**154**	SOX2	SOX2_HUMAN	NCBI
**155**	SLC2A1	GTR1_HUMAN	NCBI
**156**	EIF3H	EIF3H_HUMAN	NCBI
**157**	ERCC2	ERCC2_HUMAN	NCBI
**158**	SOD2	SODM_HUMAN	NCBI
**159**	HLA-A	HLAA_HUMAN	NCBI
**160**	NAT2	ARY2_HUMAN	NCBI
**161**	SMAD4	SMAD4_HUMAN	NCBI
**162**	TP63	P63_HUMAN	NCBI

No.	Symbol	Uniprot Name	Database
**163**	MSH2	MSH2_HUMAN	NCBI
**164**	HGF	HGF_HUMAN	NCBI
**165**	RHOA	RHOA_HUMAN	NCBI
**166**	HRAS	RASH_HUMAN	NCBI
**167**	CCL5	CCL5_HUMAN	NCBI
**168**	VWF	VWF_HUMAN	NCBI
**169**	RAD51	RAD51_HUMAN	NCBI
**170**	FOXO3	FOXO3_HUMAN	NCBI
**171**	MAPK3	MK03_HUMAN	NCBI
**172**	E2F1	E2F1_HUMAN	NCBI
**173**	FLT1	VGFR1_HUMAN	NCBI
**174**	F5	FA5_HUMAN	NCBI
**175**	FN1	FINC_HUMAN	NCBI
**176**	IGF2	IGF2_HUMAN	NCBI
**177**	RET	RET_HUMAN	NCBI
**178**	HLA-DQB1	DQB1_HUMAN	NCBI
**179**	STAT1	STAT1_HUMAN	NCBI
**180**	ERCC1	ERCC1_HUMAN	NCBI
**181**	PRNP	APRIO_HUMAN	NCBI
**182**	PRNP	PRIO_HUMAN	NCBI
**183**	HSPA5	BIP_HUMAN	NCBI
**184**	TWIST1	TWST1_HUMAN	NCBI
**185**	PROM1	PROM1_HUMAN	NCBI
**186**	IGFBP3	IBP3_HUMAN	NCBI
**187**	APEX1	APEX1_HUMAN	NCBI
**188**	CYP2C9	CP2C9_HUMAN	NCBI
**189**	DNMT1	DNMT1_HUMAN	NCBI
**190**	INS	INS_HUMAN	NCBI
**191**	POU5F1	PO5F1_HUMAN	NCBI
**192**	TIMP1	TIMP1_HUMAN	NCBI
**193**	EGF	EGF_HUMAN	NCBI
**194**	OGG1	OGG1_HUMAN	NCBI
**195**	ADRB2	ADRB2_HUMAN	NCBI
**196**	PRKN	PRKN_HUMAN	NCBI
**197**	FOXM1	FOXM1_HUMAN	NCBI
**198**	CHEK2	CHK2_HUMAN	NCBI
**199**	IDH1	IDHC_HUMAN	NCBI
**200**	EDN1	EDN1_HUMAN	NCBI
**201**	GHRL	GHRL_HUMAN	NCBI
**202**	DICER1	DICER_HUMAN	NCBI
**203**	RUNX1	RUNX1_HUMAN	NCBI

No.	Symbol	Uniprot Name	Database
**204**	NOS2	NOS2_HUMAN	NCBI
**205**	BCL2L1	B2CL1_HUMAN	NCBI
**206**	BSG	BASI_HUMAN	NCBI
**207**	IL1RN	IL1RA_HUMAN	NCBI
**208**	TP73	P73_HUMAN	NCBI
**209**	CYP2E1	CP2E1_HUMAN	NCBI
**210**	PKM	KPYM_HUMAN	NCBI
**211**	CREB1	CREB1_HUMAN	NCBI
**212**	MCL1	MCL1_HUMAN	NCBI
**213**	TYMS	TYSY_HUMAN	NCBI
**214**	ABO	BGAT_HUMAN	NCBI
**215**	VIM	VIME_HUMAN	NCBI
**216**	ALK	ALK_HUMAN	NCBI
**217**	CYP3A4	CP3A4_HUMAN	NCBI
**218**	GJB2	CXB2_HUMAN	NCBI
**219**	ZEB1	ZEB1_HUMAN	NCBI
**220**	PTPN11	PTN11_HUMAN	NCBI
**221**	AHR	AHR_HUMAN	NCBI
**222**	HSP90AA1	HS90A_HUMAN	NCBI
**223**	CHI3L1	CH3L1_HUMAN	NCBI
**224**	PLK1	PLK1_HUMAN	NCBI
**225**	SNAI1	SNAI1_HUMAN	NCBI
**226**	CDC42	CDC42_HUMAN	NCBI
**227**	PPARGC1A	PRGC1_HUMAN	NCBI
**228**	LEPR	LEPR_HUMAN	NCBI
**229**	HDAC1	HDAC1_HUMAN	NCBI
**230**	DPP4	DPP4_HUMAN	NCBI
**231**	MECP2	MECP2_HUMAN	NCBI
**232**	AGTR1	AGTR1_HUMAN	NCBI
**233**	BECN1	BECN1_HUMAN	NCBI
**234**	SMAD3	SMAD3_HUMAN	NCBI
**235**	IL33	IL33_HUMAN	NCBI
**236**	CDK1	CDK1_HUMAN	NCBI
**237**	CASR	CASR_HUMAN	NCBI
**238**	CD14	CD14_HUMAN	NCBI
**239**	NPM1	NPM_HUMAN	NCBI
**240**	RETN	RETN_HUMAN	NCBI
**241**	SOX9	SOX9_HUMAN	NCBI
**242**	NAMPT	NAMPT_HUMAN	NCBI
**243**	CLU	CLUS_HUMAN	NCBI
**244**	NQO1	NQO1_HUMAN	NCBI

No.	Symbol	Uniprot Name	Database
**245**	SCN5A	SCN5A_HUMAN	NCBI
**246**	FMR1	FMR1_HUMAN	NCBI
**247**	CXCL10	CXL10_HUMAN	NCBI
**248**	FOXO1	FOXO1_HUMAN	NCBI
**249**	TLR9	TLR9_HUMAN	NCBI
**250**	WT1	WT1_HUMAN	NCBI
**251**	SERPINA1	A1AT_HUMAN	NCBI
**252**	IL1A	IL1A_HUMAN	NCBI
**253**	HLA-C	HLAC_HUMAN	NCBI
**254**	TPH2	TPH2_HUMAN	NCBI
**255**	LAMA5	LAMA5_HUMAN	NCBI
**256**	EZR	EZRI_HUMAN	NCBI
**257**	BMI1	BMI1_HUMAN	NCBI
**258**	HAVCR2	HAVR2_HUMAN	NCBI
**259**	KLF4	KLF4_HUMAN	NCBI
**260**	COL1A1	CO1A1_HUMAN	NCBI
**261**	MMP7	MMP7_HUMAN	NCBI
**262**	PCSK9	PCSK9_HUMAN	NCBI
**263**	TNFRSF11B	TR11B_HUMAN	NCBI
**264**	MMP3	MMP3_HUMAN	NCBI
**265**	FGF2	FGF2_HUMAN	NCBI
**266**	TGFBR1	TGFR1_HUMAN	NCBI
**267**	PLAUR	UPAR_HUMAN	NCBI
**268**	GJA1	CXA1_HUMAN	NCBI
**269**	F3	TF_HUMAN	NCBI
**270**	ABCA1	ABCA1_HUMAN	NCBI
**271**	CST3	CYTC_HUMAN	NCBI
**272**	POLE	DPOE1_HUMAN	NCBI
**273**	IL13	IL13_HUMAN	NCBI
**274**	VHL	VHL_HUMAN	NCBI
**275**	ALB	ALBU_HUMAN	NCBI
**276**	MAPK8	MK08_HUMAN	NCBI
**277**	INSR	INSR_HUMAN	NCBI
**278**	FGFR2	FGFR2_HUMAN	NCBI
**279**	ERBB3	ERBB3_HUMAN	NCBI
**280**	MPO	PERM_HUMAN	NCBI
**281**	SQSTM1	SQSTM_HUMAN	NCBI
**282**	TGM2	TGM2_HUMAN	NCBI
**283**	KCNH2	KCNH2_HUMAN	NCBI
**284**	FBXW7	FBXW7_HUMAN	NCBI
**285**	RUNX3	RUNX3_HUMAN	NCBI

No.	Symbol	Uniprot Name	Database
**286**	YBX1	YBOX1_HUMAN	NCBI
**287**	HSPB1	HSPB1_HUMAN	NCBI
**288**	GNAS	GNAS3_HUMAN	NCBI
**289**	GNAS	GNAS2_HUMAN	NCBI
**290**	GNAS	ALEX_HUMAN	NCBI
**291**	GNAS	GNAS1_HUMAN	NCBI
**292**	DNMT3B	DNM3B_HUMAN	NCBI
**293**	ANGPT2	ANGP2_HUMAN	NCBI
**294**	HMGA2	HMGA2_HUMAN	NCBI
**295**	DNMT3A	DNM3A_HUMAN	NCBI
**296**	SMAD2	SMAD2_HUMAN	NCBI
**297**	TIMP2	TIMP2_HUMAN	NCBI
**298**	IDO1	I23O1_HUMAN	NCBI
**299**	PGR	PRGR_HUMAN	NCBI
**300**	PLAU	UROK_HUMAN	NCBI
**301**	IL12B	IL12B_HUMAN	NCBI
**302**	ENG	EGLN_HUMAN	NCBI
**303**	PLA2G2A	PA2GA_HUMAN	NCBI
**304**	RASSF1	RASF1_HUMAN	NCBI
**305**	IL2	IL2_HUMAN	NCBI
**306**	CETP	CETP_HUMAN	NCBI
**307**	MICA	MICA_HUMAN	NCBI
**308**	XRCC3	XRCC3_HUMAN	NCBI
**309**	OPRM1	OPRM_HUMAN	NCBI
**310**	DKK1	DKK1_HUMAN	NCBI
**311**	IRS1	IRS1_HUMAN	NCBI
**312**	SMARCA4	SMCA4_HUMAN	NCBI
**313**	ABL1	ABL1_HUMAN	NCBI
**314**	FGFR1	FGFR1_HUMAN	NCBI
**315**	GRN	GRN_HUMAN	NCBI
**316**	KL	KLOT_HUMAN	NCBI
**317**	CREBBP	CBP_HUMAN	NCBI
**318**	FCGR3A	FCG3A_HUMAN	NCBI
**319**	CYP1B1	CP1B1_HUMAN	NCBI
**320**	CSNK2A1	CSK21_HUMAN	NCBI
**321**	WNT5A	WNT5A_HUMAN	NCBI
**322**	EGR1	EGR1_HUMAN	NCBI
**323**	TRAF6	TRAF6_HUMAN	NCBI
**324**	PRKCA	KPCA_HUMAN	NCBI
**325**	VEGFC	VEGFC_HUMAN	NCBI
**326**	CDX2	CDX2_HUMAN	NCBI

No.	Symbol	Uniprot Name	Database
**327**	LGALS1	LEG1_HUMAN	NCBI
**328**	NF1	NF1_HUMAN	NCBI
**329**	C3	CO3_HUMAN	NCBI
**330**	SOCS3	SOCS3_HUMAN	NCBI
**331**	CYP3A5	CP3A5_HUMAN	NCBI
**332**	EPCAM	EPCAM_HUMAN	NCBI
**333**	FASN	FAS_HUMAN	NCBI
**334**	ATF1	ATF1_HUMAN	NCBI
**335**	EPAS1	EPAS1_HUMAN	NCBI
**336**	HDAC2	HDAC2_HUMAN	NCBI
**337**	P2RX7	P2RX7_HUMAN	NCBI
**338**	ARID1A	ARI1A_HUMAN	NCBI
**339**	TLR3	TLR3_HUMAN	NCBI
**340**	EPHA2	EPHA2_HUMAN	NCBI
**341**	PRKAA1	AAPK1_HUMAN	NCBI
**342**	YY1	TYY1_HUMAN	NCBI
**343**	ALDH1A1	AL1A1_HUMAN	NCBI
**344**	BUB1B	BUB1B_HUMAN	NCBI
**345**	AXIN2	AXIN2_HUMAN	NCBI
**346**	PCNA	PCNA_HUMAN	NCBI
**347**	THBS1	TSP1_HUMAN	NCBI
**348**	FEN1	FEN1_HUMAN	NCBI
**349**	GLI1	GLI1_HUMAN	NCBI
**350**	MMP14	MMP14_HUMAN	NCBI
**351**	KDM1A	KDM1A_HUMAN	NCBI
**352**	NFKBIA	IKBA_HUMAN	NCBI
**353**	SYK	KSYK_HUMAN	NCBI
**354**	TOP2A	TOP2A_HUMAN	NCBI
**355**	ELAVL1	ELAV1_HUMAN	NCBI
**356**	S100A9	S10A9_HUMAN	NCBI
**357**	FASLG	TNFL6_HUMAN	NCBI
**358**	CALCA	CALC_HUMAN	NCBI
**359**	CALCA	CALCA_HUMAN	NCBI
**360**	SHH	SHH_HUMAN	NCBI
**361**	KCNQ1	KCNQ1_HUMAN	NCBI
**362**	METTL3	MTA70_HUMAN	NCBI
**363**	CD9	CD9_HUMAN	NCBI
**364**	CBL	CBL_HUMAN	NCBI
**365**	CYP1A2	CP1A2_HUMAN	NCBI
**366**	GC	VTDB_HUMAN	NCBI
**367**	S100A4	S10A4_HUMAN	NCBI

No.	Symbol	Uniprot Name	Database
**368**	PRKDC	PRKDC_HUMAN	NCBI
**369**	PIK3R1	P85A_HUMAN	NCBI
**370**	ADAMTS13	ATS13_HUMAN	NCBI
**371**	XIAP	XIAP_HUMAN	NCBI
**372**	FCGR2A	FCG2A_HUMAN	NCBI
**373**	RUNX2	RUNX2_HUMAN	NCBI
**374**	DLC1	RHG07_HUMAN	NCBI
**375**	L1CAM	L1CAM_HUMAN	NCBI
**376**	ANXA2	ANXA2_HUMAN	NCBI
**377**	MUTYH	MUTYH_HUMAN	NCBI
**378**	HNF1A	HNF1A_HUMAN	NCBI
**379**	CNR1	CNR1_HUMAN	NCBI
**380**	GALNT12	GLT12_HUMAN	NCBI
**381**	MSH6	MSH6_HUMAN	NCBI
**382**	HP	HPT_HUMAN	NCBI
**383**	CD40	TNR5_HUMAN	NCBI
**384**	SPARC	SPRC_HUMAN	NCBI
**385**	SNAI2	SNAI2_HUMAN	NCBI
**386**	KEAP1	KEAP1_HUMAN	NCBI
**387**	MTDH	LYRIC_HUMAN	NCBI
**388**	STK11	STK11_HUMAN	NCBI
**389**	CD36	CD36_HUMAN	NCBI
**390**	ERBB4	ERBB4_HUMAN	NCBI
**391**	KMT2A	KMT2A_HUMAN	NCBI
**392**	PTX3	PTX3_HUMAN	NCBI
**393**	PAK1	PAK1_HUMAN	NCBI
**394**	PDGFRA	PGFRA_HUMAN	NCBI
**395**	ADAM17	ADA17_HUMAN	NCBI
**396**	CD24	CD24_HUMAN	NCBI
**397**	NRG1	NRG1_HUMAN	NCBI
**398**	TNFAIP3	TNAP3_HUMAN	NCBI
**399**	HSPA4	HSP74_HUMAN	NCBI
**400**	PPARD	PPARD_HUMAN	NCBI
**401**	NOTCH3	NOTC3_HUMAN	NCBI
**402**	IL2RA	IL2RA_HUMAN	NCBI
**403**	B2M	B2MG_HUMAN	NCBI
**404**	POSTN	POSTN_HUMAN	NCBI
**405**	ITGAV	ITAV_HUMAN	NCBI
**406**	CHEK1	CHK1_HUMAN	NCBI
**407**	CALR	CALR_HUMAN	NCBI
**408**	PRKCD	KPCD_HUMAN	NCBI

No.	Symbol	Uniprot Name	Database
**409**	XPC	XPC_HUMAN	NCBI
**410**	MYD88	MYD88_HUMAN	NCBI
**411**	ITGB2	ITB2_HUMAN	NCBI
**412**	APOBEC3G	ABC3G_HUMAN	NCBI
**413**	FADS1	FADS1_HUMAN	NCBI
**414**	CD40LG	CD40L_HUMAN	NCBI
**415**	IL15	IL15_HUMAN	NCBI
**416**	TXN	THIO_HUMAN	NCBI
**417**	SOCS1	SOCS1_HUMAN	NCBI
**418**	FUS	FUS_HUMAN	NCBI
**419**	HNF4A	HNF4A_HUMAN	NCBI
**420**	IL22	IL22_HUMAN	NCBI
**421**	FUT2	FUT2_HUMAN	NCBI
**422**	PITX1	PITX1_HUMAN	NCBI
**423**	VCP	TERA_HUMAN	NCBI
**424**	FADS2	FADS2_HUMAN	NCBI
**425**	S100B	S100B_HUMAN	NCBI
**426**	GRB2	GRB2_HUMAN	NCBI
**427**	TRPV1	TRPV1_HUMAN	NCBI
**428**	ABCC1	MRP1_HUMAN	NCBI
**429**	SDC1	SDC1_HUMAN	NCBI
**430**	CEACAM5	CEAM5_HUMAN	NCBI
**431**	HAMP	HEPC_HUMAN	NCBI
**432**	MLH3	MLH3_HUMAN	NCBI
**433**	PDGFRB	PGFRB_HUMAN	NCBI
**434**	F7	FA7_HUMAN	NCBI
**435**	CDKN2B	CDN2B_HUMAN	NCBI
**436**	CA9	CAH9_HUMAN	NCBI
**437**	ADH1B	ADH1B_HUMAN	NCBI
**438**	VCAM1	VCAM1_HUMAN	NCBI
**439**	GH1	SOMA_HUMAN	NCBI
**440**	SKP2	SKP2_HUMAN	NCBI
**441**	ATR	ATR_HUMAN	NCBI
**442**	RAF1	RAF1_HUMAN	NCBI
**443**	NBN	NBN_HUMAN	NCBI
**444**	ITGA5	ITA5_HUMAN	NCBI
**445**	HSPD1	CH60_HUMAN	NCBI
**446**	CCNB1	CCNB1_HUMAN	NCBI
**447**	MYH9	MYH9_HUMAN	NCBI
**448**	ANXA1	ANXA1_HUMAN	NCBI
**449**	EPO	EPO_HUMAN	NCBI

No.	Symbol	Uniprot Name	Database
**450**	MAP2K1	MP2K1_HUMAN	NCBI
**451**	IL23R	IL23R_HUMAN	NCBI
**452**	F2RL1	PAR2_HUMAN	NCBI
**453**	CCR2	CCR2_HUMAN	NCBI
**454**	ZEB2	ZEB2_HUMAN	NCBI
**455**	ADAR	DSRAD_HUMAN	NCBI
**456**	NDRG1	NDRG1_HUMAN	NCBI
**457**	TET2	TET2_HUMAN	NCBI
**458**	DPYD	DPYD_HUMAN	NCBI
**459**	PARK7	PARK7_HUMAN	NCBI
**460**	TNNT2	TNNT2_HUMAN	NCBI
**461**	STIM1	STIM1_HUMAN	NCBI
**462**	LRP1	LRP1_HUMAN	NCBI
**463**	PINK1	PINK1_HUMAN	NCBI
**464**	PML	PML_HUMAN	NCBI
**465**	PIN1	PIN1_HUMAN	NCBI
**466**	MMP13	MMP13_HUMAN	NCBI
**467**	FOS	FOS_HUMAN	NCBI
**468**	ETS1	ETS1_HUMAN	NCBI
**469**	TNFRSF1B	TNR1B_HUMAN	NCBI
**470**	IGF2BP3	IF2B3_HUMAN	NCBI
**471**	AHSG	FETUA_HUMAN	NCBI
**472**	IDH2	IDHP_HUMAN	NCBI
**473**	SPHK1	SPHK1_HUMAN	NCBI
**474**	IKZF1	IKZF1_HUMAN	NCBI
**475**	TOP1	TOP1_HUMAN	NCBI
**476**	HMGA1	HMGA1_HUMAN	NCBI
**477**	AFP	FETA_HUMAN	NCBI
**478**	PAX6	PAX6_HUMAN	NCBI
**479**	IL23A	IL23A_HUMAN	NCBI
**480**	PGF	PLGF_HUMAN	NCBI
**481**	NME1	NDKA_HUMAN	NCBI
**482**	ATP7B	ATP7B_HUMAN	NCBI
**483**	POLD1	DPOD1_HUMAN	NCBI
**484**	FLCN	FLCN_HUMAN	NCBI
**485**	ROCK1	ROCK1_HUMAN	NCBI
**486**	CLDN1	CLD1_HUMAN	NCBI
**487**	TIMP3	TIMP3_HUMAN	NCBI
**488**	BCL2L11	B2L11_HUMAN	NCBI
**489**	KLRK1	NKG2D_HUMAN	NCBI
**490**	BUB1	BUB1_HUMAN	NCBI

No.	Symbol	Uniprot Name	Database
**491**	SIRT3	SIR3_HUMAN	NCBI
**492**	HSPA8	HSP7C_HUMAN	NCBI
**493**	MUC16	MUC16_HUMAN	NCBI
**494**	MME	NEP_HUMAN	NCBI
**495**	NRP1	NRP1_HUMAN	NCBI
**496**	PPIA	PPIA_HUMAN	NCBI
**497**	PDCD4	PDCD4_HUMAN	NCBI
**498**	CAMP	CAMP_HUMAN	NCBI
**499**	CD276	CD276_HUMAN	NCBI
**500**	ANGPTL4	ANGL4_HUMAN	NCBI
**501**	ADM	ADML_HUMAN	NCBI
**502**	NT5E	5NTD_HUMAN	NCBI
**503**	FNDC5	FNDC5_HUMAN	NCBI
**504**	PRMT5	ANM5_HUMAN	NCBI
**505**	CYP24A1	CP24A_HUMAN	NCBI
**506**	SHBG	SHBG_HUMAN	NCBI
**507**	MTRR	MTRR_HUMAN	NCBI
**508**	FLNA	FLNA_HUMAN	NCBI
**509**	CD209	CD209_HUMAN	NCBI
**510**	CXCR2	CXCR2_HUMAN	NCBI
**511**	BRD4	BRD4_HUMAN	NCBI
**512**	HPSE	HPSE_HUMAN	NCBI
**513**	XRCC6	XRCC6_HUMAN	NCBI
**514**	IL21	IL21_HUMAN	NCBI
**515**	XPO1	XPO1_HUMAN	NCBI
**516**	CASP9	CASP9_HUMAN	NCBI
**517**	SLCO1B1	SO1B1_HUMAN	NCBI
**518**	UCHL1	UCHL1_HUMAN	NCBI
**519**	CEBPB	CEBPB_HUMAN	NCBI
**520**	BACE1	BACE1_HUMAN	NCBI
**521**	SMARCB1	SNF5_HUMAN	NCBI
**522**	FHIT	FHIT_HUMAN	NCBI
**523**	ADORA2A	AA2AR_HUMAN	NCBI
**524**	KISS1	KISS1_HUMAN	NCBI
**525**	GZMB	GRAB_HUMAN	NCBI
**526**	FKBP5	FKBP5_HUMAN	NCBI
**527**	TTN	TITIN_HUMAN	NCBI
**528**	JAG1	JAG1_HUMAN	NCBI
**529**	AXL	UFO_HUMAN	NCBI
**530**	FOXA1	FOXA1_HUMAN	NCBI
**531**	F2R	PAR1_HUMAN	NCBI

No.	Symbol	Uniprot Name	Database
**532**	CXCR3	CXCR3_HUMAN	NCBI
**533**	NANOG	NANOG_HUMAN	NCBI
**534**	OLR1	OLR1_HUMAN	NCBI
**535**	LDHA	LDHA_HUMAN	NCBI
**536**	CAT	CATA_HUMAN	NCBI
**537**	GNB3	GBB3_HUMAN	NCBI
**538**	TSC2	TSC2_HUMAN	NCBI
**539**	CXCL1	GROA_HUMAN	NCBI
**540**	WWTR1	WWTR1_HUMAN	NCBI
**541**	LEF1	LEF1_HUMAN	NCBI
**542**	ERG	ERG_HUMAN	NCBI
**543**	APOC3	APOC3_HUMAN	NCBI
**544**	IL4R	IL4RA_HUMAN	NCBI
**545**	MC1R	MSHR_HUMAN	NCBI
**546**	CRYAB	CRYAB_HUMAN	NCBI
**547**	PTPRC	PTPRC_HUMAN	NCBI
**548**	NCAM1	NCAM1_HUMAN	NCBI
**549**	JAK1	JAK1_HUMAN	NCBI
**550**	PTPN1	PTN1_HUMAN	NCBI
**551**	SELE	LYAM2_HUMAN	NCBI
**552**	SELP	LYAM3_HUMAN	NCBI
**553**	RIPK1	RIPK1_HUMAN	NCBI
**554**	AURKB	AURKB_HUMAN	NCBI
**555**	SOX4	SOX4_HUMAN	NCBI
**556**	SHC1	SHC1_HUMAN	NCBI
**557**	CTCF	CTCF_HUMAN	NCBI
**558**	HSF1	HSF1_HUMAN	NCBI
**559**	TFRC	TFR1_HUMAN	NCBI
**560**	BAP1	BAP1_HUMAN	NCBI
**561**	LTF	TRFL_HUMAN	NCBI
**562**	ITGA2	ITA2_HUMAN	NCBI
**563**	CBS	CBS_HUMAN	NCBI
**564**	PXN	PAXI_HUMAN	NCBI
**565**	CCNE1	CCNE1_HUMAN	NCBI
**566**	TGFB2	TGFB2_HUMAN	NCBI
**567**	EIF2AK2	E2AK2_HUMAN	NCBI
**568**	NF2	MERL_HUMAN	NCBI
**569**	NES	NEST_HUMAN	NCBI
**570**	STMN1	STMN1_HUMAN	NCBI
**571**	NTRK1	NTRK1_HUMAN	NCBI
**572**	CALM1	CALM1_HUMAN	NCBI

No.	Symbol	Uniprot Name	Database
**573**	FGB	FIBB_HUMAN	NCBI
**574**	CD34	CD34_HUMAN	NCBI
**575**	IRF1	IRF1_HUMAN	NCBI
**576**	UHRF1	UHRF1_HUMAN	NCBI
**577**	UCP2	UCP2_HUMAN	NCBI
**578**	IFNB1	IFNB_HUMAN	NCBI
**579**	PTH	PTHY_HUMAN	NCBI
**580**	FABP4	FABP4_HUMAN	NCBI
**581**	CD28	CD28_HUMAN	NCBI
**582**	SUMO1	SUMO1_HUMAN	NCBI
**583**	TNC	TENA_HUMAN	NCBI
**584**	XRCC5	XRCC5_HUMAN	NCBI
**585**	IKBKB	IKKB_HUMAN	NCBI
**586**	S100A8	S10A8_HUMAN	NCBI
**587**	CD163	C163A_HUMAN	NCBI
**588**	POLG	DPOG1_HUMAN	NCBI
**589**	LOX	LYOX_HUMAN	NCBI
**590**	INHBA	INHBA_HUMAN	NCBI
**591**	MYB	MYB_HUMAN	NCBI
**592**	CEACAM1	CEAM1_HUMAN	NCBI
**593**	SPINK1	ISK1_HUMAN	NCBI
**594**	ORAI1	ORAI1_HUMAN	NCBI
**595**	TGFBI	BGH3_HUMAN	NCBI
**596**	TNFRSF10B	TR10B_HUMAN	NCBI
**597**	LGR5	LGR5_HUMAN	NCBI
**598**	BST2	BST2_HUMAN	NCBI
**599**	MUC5AC	MUC5A_HUMAN	NCBI
**600**	KPNA2	IMA1_HUMAN	NCBI
**601**	AGO2	AGO2_HUMAN	NCBI
**602**	STUB1	CHIP_HUMAN	NCBI
**603**	IRF3	IRF3_HUMAN	NCBI
**604**	MTR	METH_HUMAN	NCBI
**605**	MRE11	MRE11_HUMAN	NCBI
**606**	PTPN6	PTN6_HUMAN	NCBI
**607**	SREBF1	SRBP1_HUMAN	NCBI
**608**	SATB1	SATB1_HUMAN	NCBI
**609**	IL17F	IL17F_HUMAN	NCBI
**610**	PKD1	PKD1_HUMAN	NCBI
**611**	BCL6	BCL6_HUMAN	NCBI
**612**	PIK3CG	PK3CG_HUMAN	NCBI
**613**	WNK1	WNK1_HUMAN	NCBI

No.	Symbol	Uniprot Name	Database
**614**	MEN1	MEN1_HUMAN	NCBI
**615**	GPER1	GPER1_HUMAN	NCBI
**616**	NR1I2	NR1I2_HUMAN	NCBI
**617**	NCL	NUCL_HUMAN	NCBI
**618**	NOX4	NOX4_HUMAN	NCBI
**619**	FGA	FIBA_HUMAN	NCBI
**620**	IL12A	IL12A_HUMAN	NCBI
**621**	SERPINB5	SPB5_HUMAN	NCBI
**622**	EPHX1	HYEP_HUMAN	NCBI
**623**	HDAC6	HDAC6_HUMAN	NCBI
**624**	XBP1	XBP1_HUMAN	NCBI
**625**	SERPINF1	PEDF_HUMAN	NCBI
**626**	PEBP1	PEBP1_HUMAN	NCBI
**627**	PDGFB	PDGFB_HUMAN	NCBI
**628**	NTRK2	NTRK2_HUMAN	NCBI
**629**	RXRA	RXRA_HUMAN	NCBI
**630**	NPY	NPY_HUMAN	NCBI
**631**	PTBP1	PTBP1_HUMAN	NCBI
**632**	IL27	IL27A_HUMAN	NCBI
**633**	RPS6KB1	KS6B1_HUMAN	NCBI
**634**	CD38	CD38_HUMAN	NCBI
**635**	ADAM10	ADA10_HUMAN	NCBI
**636**	PTTG1	PTTG1_HUMAN	NCBI
**637**	CTSB	CATB_HUMAN	NCBI
**638**	IGF2BP2	IF2B2_HUMAN	NCBI
**639**	PPP2CA	PP2AA_HUMAN	NCBI
**640**	CFLAR	CFLAR_HUMAN	NCBI
**641**	TSLP	TSLP_HUMAN	NCBI
**642**	ALOX5	LOX5_HUMAN	NCBI
**643**	NCOA3	NCOA3_HUMAN	NCBI
**644**	PRMT1	ANM1_HUMAN	NCBI
**645**	STAT6	STAT6_HUMAN	NCBI
**646**	CHRNA7	ACHA7_HUMAN	NCBI
**647**	BTK	BTK_HUMAN	NCBI
**648**	CDK6	CDK6_HUMAN	NCBI
**649**	HDAC3	HDAC3_HUMAN	NCBI
**650**	KLF5	KLF5_HUMAN	NCBI
**651**	CYP2A6	CP2A6_HUMAN	NCBI
**652**	FGFR4	FGFR4_HUMAN	NCBI
**653**	TBK1	TBK1_HUMAN	NCBI
**654**	ELN	ELN_HUMAN	NCBI

No.	Symbol	Uniprot Name	Database
**655**	GATA2	GATA2_HUMAN	NCBI
**656**	GAPDH	G3P_HUMAN	NCBI
**657**	CX3CR1	CX3C1_HUMAN	NCBI
**658**	EPDR1	EPDR1_HUMAN	NCBI
**659**	USP7	UBP7_HUMAN	NCBI
**660**	CHGA	CMGA_HUMAN	NCBI
**661**	COL1A2	CO1A2_HUMAN	NCBI
**662**	STAT5A	STA5A_HUMAN	NCBI
**663**	IGFBP1	IBP1_HUMAN	NCBI
**664**	GPX1	GPX1_HUMAN	NCBI
**665**	ANO1	ANO1_HUMAN	NCBI
**666**	HLA-E	HLAE_HUMAN	NCBI
**667**	HNRNPK	HNRPK_HUMAN	NCBI
**668**	CCL20	CCL20_HUMAN	NCBI
**669**	ID1	ID1_HUMAN	NCBI
**670**	SIRT2	SIR2_HUMAN	NCBI
**671**	PRKCB	KPCB_HUMAN	NCBI
**672**	IL7R	IL7RA_HUMAN	NCBI
**673**	NGFR	TNR16_HUMAN	NCBI
**674**	TP53BP1	TP53B_HUMAN	NCBI
**675**	GSN	GELS_HUMAN	NCBI
**676**	IGFBP2	IBP2_HUMAN	NCBI
**677**	SIRT6	SIR6_HUMAN	NCBI
**678**	HTRA1	HTRA1_HUMAN	NCBI
**679**	NAT1	ARY1_HUMAN	NCBI
**680**	HNRNPA1	ROA1_HUMAN	NCBI
**681**	PMS2	PMS2_HUMAN	NCBI
**682**	RAN	RAN_HUMAN	NCBI
**683**	CDH2	CADH2_HUMAN	NCBI
**684**	TRPV4	TRPV4_HUMAN	NCBI
**685**	ITGB4	ITB4_HUMAN	NCBI
**686**	CTTN	SRC8_HUMAN	NCBI
**687**	WRN	WRN_HUMAN	NCBI
**688**	TMPRSS2	TMPS2_HUMAN	NCBI
**689**	KRT18	K1C18_HUMAN	NCBI
**690**	MTA1	MTA1_HUMAN	NCBI
**691**	ATF3	ATF3_HUMAN	NCBI
**692**	RHPN2	RHPN2_HUMAN	NCBI
**693**	CD47	CD47_HUMAN	NCBI
**694**	FSCN1	FSCN1_HUMAN	NCBI
**695**	TCF4	ITF2_HUMAN	NCBI

No.	Symbol	Uniprot Name	Database
**696**	CHUK	IKKA_HUMAN	NCBI
**697**	SAA1	SAA1_HUMAN	NCBI
**698**	CTSD	CATD_HUMAN	NCBI
**699**	GATA4	GATA4_HUMAN	NCBI
**700**	KRT19	K1C19_HUMAN	NCBI
**701**	SLC19A1	S19A1_HUMAN	NCBI
**702**	EWSR1	EWS_HUMAN	NCBI
**703**	AKT2	AKT2_HUMAN	NCBI
**704**	ILK	ILK_HUMAN	NCBI
**705**	ITLN1	ITLN1_HUMAN	NCBI
**706**	CA2	CAH2_HUMAN	NCBI
**707**	MEIS1	MEIS1_HUMAN	NCBI
**708**	ENO1	ENOA_HUMAN	NCBI
**709**	CUL1	CUL1_HUMAN	NCBI
**710**	DUSP10	DUS10_HUMAN	NCBI
**711**	TREM1	TREM1_HUMAN	NCBI
**712**	PTP4A3	TP4A3_HUMAN	NCBI
**713**	FUT3	FUT3_HUMAN	NCBI
**714**	BMP7	BMP7_HUMAN	NCBI
**715**	CLOCK	CLOCK_HUMAN	NCBI
**716**	CDK9	CDK9_HUMAN	NCBI
**717**	FARP1	FARP1_HUMAN	NCBI
**718**	PTCH1	PTC1_HUMAN	NCBI
**719**	CDK5	CDK5_HUMAN	NCBI
**720**	CCR7	CCR7_HUMAN	NCBI
**721**	SDHB	SDHB_HUMAN	NCBI
**722**	NTN1	NET1_HUMAN	NCBI
**723**	ITGA6	ITA6_HUMAN	NCBI
**724**	MAP1LC3A	MLP3A_HUMAN	NCBI
**725**	PTPRJ	PTPRJ_HUMAN	NCBI
**726**	RARRES2	RARR2_HUMAN	NCBI
**727**	GATA1	GATA1_HUMAN	NCBI
**728**	HSD11B1	DHI1_HUMAN	NCBI
**729**	SF3B1	SF3B1_HUMAN	NCBI
**730**	HNF1B	HNF1B_HUMAN	NCBI
**731**	NR4A1	NR4A1_HUMAN	NCBI
**732**	IL32	IL32_HUMAN	NCBI
**733**	ERCC5	ERCC5_HUMAN	NCBI
**734**	LYN	LYN_HUMAN	NCBI
**735**	MFN2	MFN2_HUMAN	NCBI
**736**	NR3C2	MCR_HUMAN	NCBI

No.	Symbol	Uniprot Name	Database
**737**	MUC2	MUC2_HUMAN	NCBI
**738**	HK2	HXK2_HUMAN	NCBI
**739**	TYMP	TYPH_HUMAN	NCBI
**740**	LRP5	LRP5_HUMAN	NCBI
**741**	HSP90B1	ENPL_HUMAN	NCBI
**742**	ARG1	ARGI1_HUMAN	NCBI
**743**	ATF4	ATF4_HUMAN	NCBI
**744**	CASP1	CASP1_HUMAN	NCBI
**745**	PDPN	PDPN_HUMAN	NCBI
**746**	TUBB3	TBB3_HUMAN	NCBI
**747**	RYR2	RYR2_HUMAN	NCBI
**748**	PLCG1	PLCG1_HUMAN	NCBI
**749**	PIM1	PIM1_HUMAN	NCBI
**750**	CSF2	CSF2_HUMAN	NCBI
**751**	DAPK1	DAPK1_HUMAN	NCBI
**752**	CSF1R	CSF1R_HUMAN	NCBI
**753**	TJP1	ZO1_HUMAN	NCBI
**754**	CYLD	CYLD_HUMAN	NCBI
**755**	PRDX1	PRDX1_HUMAN	NCBI
**756**	RARB	RARB_HUMAN	NCBI
**757**	COL3A1	CO3A1_HUMAN	NCBI
**758**	FURIN	FURIN_HUMAN	NCBI
**759**	GPC3	GPC3_HUMAN	NCBI
**760**	CD55	DAF_HUMAN	NCBI
**761**	NOTCH2	NOTC2_HUMAN	NCBI
**762**	DDX3X	DDX3X_HUMAN	NCBI
**763**	PLCE1	PLCE1_HUMAN	NCBI
**764**	ANGPT1	ANGP1_HUMAN	NCBI
**765**	MACC1	MACC1_HUMAN	NCBI
**766**	SLC7A5	LAT1_HUMAN	NCBI
**767**	BLM	BLM_HUMAN	NCBI
**768**	NR1H4	NR1H4_HUMAN	NCBI
**769**	ANG	ANGI_HUMAN	NCBI
**770**	MMP8	MMP8_HUMAN	NCBI
**771**	EHMT2	EHMT2_HUMAN	NCBI
**772**	CRHR1	CRFR1_HUMAN	NCBI
**773**	AICDA	AICDA_HUMAN	NCBI
**774**	AXIN1	AXIN1_HUMAN	NCBI
**775**	CTNND1	CTND1_HUMAN	NCBI
**776**	TXNIP	TXNIP_HUMAN	NCBI
**777**	PRDX2	PRDX2_HUMAN	NCBI

No.	Symbol	Uniprot Name	Database
**778**	ARRB2	ARRB2_HUMAN	NCBI
**779**	TEK	TIE2_HUMAN	NCBI
**780**	FLT4	VGFR3_HUMAN	NCBI
**781**	TCF3	TFE2_HUMAN	NCBI
**782**	LIPC	LIPC_HUMAN	NCBI
**783**	SFRP1	SFRP1_HUMAN	NCBI
**784**	CSF1	CSF1_HUMAN	NCBI
**785**	SLC7A11	XCT_HUMAN	NCBI
**786**	STAT4	STAT4_HUMAN	NCBI
**787**	ADAM12	ADA12_HUMAN	NCBI
**788**	NR1H3	NR1H3_HUMAN	NCBI
**789**	FABP2	FABPI_HUMAN	NCBI
**790**	ALOX15	LOX15_HUMAN	NCBI
**791**	BAG3	BAG3_HUMAN	NCBI
**792**	ATP2A2	AT2A2_HUMAN	NCBI
**793**	PLCB1	PLCB1_HUMAN	NCBI
**794**	BBC3	BBC3B_HUMAN	NCBI
**795**	BBC3	BBC3_HUMAN	NCBI
**796**	CXCL5	CXCL5_HUMAN	NCBI
**797**	ETV6	ETV6_HUMAN	NCBI
**798**	TBP	TBP_HUMAN	NCBI
**799**	ACKR3	ACKR3_HUMAN	NCBI
**800**	FAP	SEPR_HUMAN	NCBI
**801**	KITLG	SCF_HUMAN	NCBI
**802**	ADIPOR1	PAQR1_HUMAN	NCBI
**803**	PTHLH	PTHR_HUMAN	NCBI
**804**	PRKCE	KPCE_HUMAN	NCBI
**805**	TNFRSF10A	TR10A_HUMAN	NCBI
**806**	DDIT3	DDIT3_HUMAN	NCBI
**807**	MAD2L1	MD2L1_HUMAN	NCBI
**808**	RAD50	RAD50_HUMAN	NCBI
**809**	KAT2B	KAT2B_HUMAN	NCBI
**810**	CFL1	COF1_HUMAN	NCBI
**811**	PRKCZ	KPCZ_HUMAN	NCBI
**812**	ESM1	ESM1_HUMAN	NCBI
**813**	IGF2BP1	IF2B1_HUMAN	NCBI
**814**	EIF4EBP1	4EBP1_HUMAN	NCBI
**815**	MCAM	MUC18_HUMAN	NCBI
**816**	CYP27B1	CP27B_HUMAN	NCBI
**817**	POLB	DPOLB_HUMAN	NCBI
**818**	TFPI	TFPI1_HUMAN	NCBI

No.	Symbol	Uniprot Name	Database
**819**	AREG	AREG_HUMAN	NCBI
**820**	NDRG2	NDRG2_HUMAN	NCBI
**821**	ATG16L1	A16L1_HUMAN	NCBI
**822**	TRIM21	RO52_HUMAN	NCBI
**823**	PTK2B	FAK2_HUMAN	NCBI
**824**	VTCN1	VTCN1_HUMAN	NCBI
**825**	MDM4	MDM4_HUMAN	NCBI
**826**	CUL4A	CUL4A_HUMAN	NCBI
**827**	HSPA9	GRP75_HUMAN	NCBI
**828**	LGALS9	LEG9_HUMAN	NCBI
**829**	FADD	FADD_HUMAN	NCBI
**830**	PTGS1	PGH1_HUMAN	NCBI
**831**	STAT5B	STA5B_HUMAN	NCBI
**832**	TRPM7	TRPM7_HUMAN	NCBI
**833**	WNT1	WNT1_HUMAN	NCBI
**834**	IL6ST	IL6RB_HUMAN	NCBI
**835**	TNFRSF11A	TNR11_HUMAN	NCBI
**836**	DDB1	DDB1_HUMAN	NCBI
**837**	XRCC4	XRCC4_HUMAN	NCBI
**838**	ENO2	ENOG_HUMAN	NCBI
**839**	NFATC1	NFAC1_HUMAN	NCBI
**840**	CCNA2	CCNA2_HUMAN	NCBI
**841**	CXCL13	CXL13_HUMAN	NCBI
**842**	TET1	TET1_HUMAN	NCBI
**843**	HNRNPA2B1	ROA2_HUMAN	NCBI
**844**	SULT1A1	ST1A1_HUMAN	NCBI
**845**	KLF6	KLF6_HUMAN	NCBI
**846**	PTPN12	PTN12_HUMAN	NCBI
**847**	PF4	PLF4_HUMAN	NCBI
**848**	CDC20	CDC20_HUMAN	NCBI
**849**	TSPO	TSPOB_HUMAN	NCBI
**850**	TSPO	TSPO_HUMAN	NCBI
**851**	PLA2G4A	PA24A_HUMAN	NCBI
**852**	ALCAM	CD166_HUMAN	NCBI
**853**	PRF1	PERF_HUMAN	NCBI
**854**	GDNF	GDNF_HUMAN	NCBI
**855**	RAB5A	RAB5A_HUMAN	NCBI
**856**	ARF6	ARF6_HUMAN	NCBI
**857**	DAXX	DAXX_HUMAN	NCBI
**858**	TFAM	TFAM_HUMAN	NCBI
**859**	DCN	PGS2_HUMAN	NCBI

No.	Symbol	Uniprot Name	Database
**860**	IRAK1	IRAK1_HUMAN	NCBI
**861**	TSC1	TSC1_HUMAN	NCBI
**862**	MDK	MK_HUMAN	NCBI
**863**	EEF1A1	EF1A1_HUMAN	NCBI
**864**	DEFB1	DEFB1_HUMAN	NCBI
**865**	FOXC1	FOXC1_HUMAN	NCBI
**866**	ENPP2	ENPP2_HUMAN	NCBI
**867**	KPNB1	IMB1_HUMAN	NCBI
**868**	ATG5	ATG5_HUMAN	NCBI
**869**	EDNRB	EDNRB_HUMAN	NCBI
**870**	FANCD2	FACD2_HUMAN	NCBI
**871**	TLR5	TLR5_HUMAN	NCBI
**872**	AGTR2	AGTR2_HUMAN	NCBI
**873**	LCT	LPH_HUMAN	NCBI
**874**	CDH13	CAD13_HUMAN	NCBI
**875**	TGFA	TGFA_HUMAN	NCBI
**876**	IRS2	IRS2_HUMAN	NCBI
**877**	ERAP1	ERAP1_HUMAN	NCBI
**878**	TERF2	TERF2_HUMAN	NCBI
**879**	COMP	COMP_HUMAN	NCBI
**880**	PDIA3	PDIA3_HUMAN	NCBI
**881**	CDKN1C	CDN1C_HUMAN	NCBI
**882**	FOLH1	FOLH1_HUMAN	NCBI
**883**	DDX5	DDX5_HUMAN	NCBI
**884**	TNFSF12	TNF12_HUMAN	NCBI
**885**	PTPA	PTPA_HUMAN	NCBI
**886**	TIAM1	TIAM1_HUMAN	NCBI
**887**	TGFB3	TGFB3_HUMAN	NCBI
**888**	MUC4	MUC4_HUMAN	NCBI
**889**	NFKB2	NFKB2_HUMAN	NCBI
**890**	ERCC4	XPF_HUMAN	NCBI
**891**	WNT3A	WNT3A_HUMAN	NCBI
**892**	BIRC3	BIRC3_HUMAN	NCBI
**893**	SATB2	SATB2_HUMAN	NCBI
**894**	CD82	CD82_HUMAN	NCBI
**895**	NEDD4L	NED4L_HUMAN	NCBI
**896**	FOXP1	FOXP1_HUMAN	NCBI
**897**	SPI1	SPI1_HUMAN	NCBI
**898**	CUL3	CUL3_HUMAN	NCBI
**899**	GAS6	GAS6_HUMAN	NCBI
**900**	TRPC6	TRPC6_HUMAN	NCBI

No.	Symbol	Uniprot Name	Database
**901**	TFF3	TFF3_HUMAN	NCBI
**902**	PDE4D	PDE4D_HUMAN	NCBI
**903**	CIP2A	CIP2A_HUMAN	NCBI
**904**	PKD2	PKD2_HUMAN	NCBI
**905**	MAP3K5	M3K5_HUMAN	NCBI
**906**	ULK1	ULK1_HUMAN	NCBI
**907**	LAMP2	LAMP2_HUMAN	NCBI
**908**	NTRK3	NTRK3_HUMAN	NCBI
**909**	CTSL	CATL1_HUMAN	NCBI
**910**	CCL3	CCL3_HUMAN	NCBI
**911**	ITGA3	ITA3_HUMAN	NCBI
**912**	FUT1	FUT1_HUMAN	NCBI
**913**	TACSTD2	TACD2_HUMAN	NCBI
**914**	SCD	SCD_HUMAN	NCBI
**915**	TAC1	TKN1_HUMAN	NCBI
**916**	KAT5	KAT5_HUMAN	NCBI
**917**	SLC16A1	MOT1_HUMAN	NCBI
**918**	CHRNA5	ACHA5_HUMAN	NCBI
**919**	DHX9	DHX9_HUMAN	NCBI
**920**	HSP90AB1	HS90B_HUMAN	NCBI
**921**	HSD11B2	DHI2_HUMAN	NCBI
**922**	ATXN3	ATX3_HUMAN	NCBI
**923**	DUSP1	DUS1_HUMAN	NCBI
**924**	DIABLO	DBLOH_HUMAN	NCBI
**925**	AIM2	AIM2_HUMAN	NCBI
**926**	TACR1	NK1R_HUMAN	NCBI
**927**	SLC3A2	4F2_HUMAN	NCBI
**928**	CIITA	C2TA_HUMAN	NCBI
**929**	AKR1B1	ALDR_HUMAN	NCBI
**930**	TRPM8	TRPM8_HUMAN	NCBI
**931**	ROS1	ROS1_HUMAN	NCBI
**932**	DCLK1	DCLK1_HUMAN	NCBI
**933**	XPA	XPA_HUMAN	NCBI
**934**	TPM1	TPM1_HUMAN	NCBI
**935**	OGT	OGT1_HUMAN	NCBI
**936**	CCR6	CCR6_HUMAN	NCBI
**937**	LRP6	LRP6_HUMAN	NCBI
**938**	ERCC6	ERCC6_HUMAN	NCBI
**939**	MECOM	MECOM_HUMAN	NCBI
**940**	COPS5	CSN5_HUMAN	NCBI
**941**	TFPI2	TFPI2_HUMAN	NCBI

No.	Symbol	Uniprot Name	Database
**942**	SFN	1433S_HUMAN	NCBI
**943**	PITX2	PITX2_HUMAN	NCBI
**944**	BIRC2	BIRC2_HUMAN	NCBI
**945**	CD247	CD3Z_HUMAN	NCBI
**946**	TFAP2A	AP2A_HUMAN	NCBI
**947**	KIF11	KIF11_HUMAN	NCBI
**948**	SFRP2	SFRP2_HUMAN	NCBI
**949**	MTNR1B	MTR1B_HUMAN	NCBI
**950**	PDPK1	PDPK1_HUMAN	NCBI
**951**	SIX1	SIX1_HUMAN	NCBI
**952**	SDC2	SDC2_HUMAN	NCBI
**953**	SALL4	SALL4_HUMAN	NCBI
**954**	MAPK7	MK07_HUMAN	NCBI
**955**	PADI4	PADI4_HUMAN	NCBI
**956**	ATF2	ATF2_HUMAN	NCBI
**957**	GPX4	GPX4_HUMAN	NCBI
**958**	S1PR1	S1PR1_HUMAN	NCBI
**959**	GRK2	ARBK1_HUMAN	NCBI
**960**	BDKRB2	BKRB2_HUMAN	NCBI
**961**	IL9	IL9_HUMAN	NCBI
**962**	ABCC4	MRP4_HUMAN	NCBI
**963**	BAD	BAD_HUMAN	NCBI
**964**	SLC11A1	NRAM1_HUMAN	NCBI
**965**	EIF2AK3	E2AK3_HUMAN	NCBI
**966**	EPHB4	EPHB4_HUMAN	NCBI
**967**	SCG5	7B2_HUMAN	NCBI
**968**	ASXL1	ASXL1_HUMAN	NCBI
**969**	HMGCR	HMDH_HUMAN	NCBI
**970**	CAPN10	CAN10_HUMAN	NCBI
**971**	SLC30A8	ZNT8_HUMAN	NCBI
**972**	SMPD1	ASM_HUMAN	NCBI
**973**	IGF2R	MPRI_HUMAN	NCBI
**974**	DYRK1A	DYR1A_HUMAN	NCBI
**975**	TRAF3	TRAF3_HUMAN	NCBI
**976**	DDR1	DDR1_HUMAN	NCBI
**977**	LASP1	LASP1_HUMAN	NCBI
**978**	PFN1	PROF1_HUMAN	NCBI
**979**	EPHB2	EPHB2_HUMAN	NCBI
**980**	IL11	IL11_HUMAN	NCBI
**981**	SET	SET_HUMAN	NCBI
**982**	BNIP3	BNIP3_HUMAN	NCBI

No.	Symbol	Uniprot Name	Database
**983**	GSTA1	GSTA1_HUMAN	NCBI
**984**	VCAN	CSPG2_HUMAN	NCBI
**985**	NCOA1	NCOA1_HUMAN	NCBI
**986**	CADM1	CADM1_HUMAN	NCBI
**987**	ESRRA	ERR1_HUMAN	NCBI
**988**	APAF1	APAF_HUMAN	NCBI
**989**	UBE2C	UBE2C_HUMAN	NCBI
**990**	AGR2	AGR2_HUMAN	NCBI
**991**	HNRNPD	HNRPD_HUMAN	NCBI
**992**	RECK	RECK_HUMAN	NCBI
**993**	DNM2	DYN2_HUMAN	NCBI
**994**	ROCK2	ROCK2_HUMAN	NCBI
**995**	TBX21	TBX21_HUMAN	NCBI
**996**	PAX5	PAX5_HUMAN	NCBI
**997**	HOXA10	HXA10_HUMAN	NCBI
**998**	TNFSF13	TNF13_HUMAN	NCBI
**999**	ARNT	ARNT_HUMAN	NCBI
**1000**	IGFBP7	IBP7_HUMAN	NCBI
**1001**	PTGDR	PD2R_HUMAN	NCBI
**1002**	HLA-DRA	DRA_HUMAN	NCBI
**1003**	LATS2	LATS2_HUMAN	NCBI
**1004**	SOS1	SOS1_HUMAN	NCBI
**1005**	SOD3	SODE_HUMAN	NCBI
**1006**	JUP	PLAK_HUMAN	NCBI
**1007**	IL16	IL16_HUMAN	NCBI
**1008**	CD86	CD86_HUMAN	NCBI
**1009**	E2F3	E2F3_HUMAN	NCBI
**1010**	FTL	FRIL_HUMAN	NCBI
**1011**	RASA1	RASA1_HUMAN	NCBI
**1012**	PRSS1	TRY1_HUMAN	NCBI
**1013**	CXCR1	CXCR1_HUMAN	NCBI
**1014**	MCM7	MCM7_HUMAN	NCBI
**1015**	COL4A5	CO4A5_HUMAN	NCBI
**1016**	NR4A2	NR4A2_HUMAN	NCBI
**1017**	RTN4	RTN4_HUMAN	NCBI
**1018**	HIPK2	HIPK2_HUMAN	NCBI
**1019**	SORT1	SORT_HUMAN	NCBI
**1020**	LOXL1	LOXL1_HUMAN	NCBI
**1021**	SSTR2	SSR2_HUMAN	NCBI
**1022**	CRK	CRK_HUMAN	NCBI
**1023**	CXCL16	CXL16_HUMAN	NCBI

No.	Symbol	Uniprot Name	Database
**1024**	HES1	HES1_HUMAN	NCBI
**1025**	SEMA3A	SEM3A_HUMAN	NCBI
**1026**	AKT3	AKT3_HUMAN	NCBI
**1027**	PPM1D	PPM1D_HUMAN	NCBI
**1028**	UPF1	RENT1_HUMAN	NCBI
**1029**	NUMB	NUMB_HUMAN	NCBI
**1030**	BCAR1	BCAR1_HUMAN	NCBI
**1031**	ZFP36	TTP_HUMAN	NCBI
**1032**	LGALS3BP	LG3BP_HUMAN	NCBI
**1033**	LIG4	DNLI4_HUMAN	NCBI
**1034**	IL24	IL24_HUMAN	NCBI
**1035**	CXCL9	CXCL9_HUMAN	NCBI
**1036**	ADD1	ADDA_HUMAN	NCBI
**1037**	FHL2	FHL2_HUMAN	NCBI
**1038**	CSK	CSK_HUMAN	NCBI
**1039**	LOXL2	LOXL2_HUMAN	NCBI
**1040**	SRSF3	SRSF3_HUMAN	NCBI
**1041**	TNFSF15	TNF15_HUMAN	NCBI
**1042**	PDX1	PDX1_HUMAN	NCBI
**1043**	MCM2	MCM2_HUMAN	NCBI
**1044**	GLI2	GLI2_HUMAN	NCBI
**1045**	LAMC2	LAMC2_HUMAN	NCBI
**1046**	CSNK2B	CSK2B_HUMAN	NCBI
**1047**	WEE1	WEE1_HUMAN	NCBI
**1048**	SLC1A5	AAAT_HUMAN	NCBI
**1049**	KMT2D	KMT2D_HUMAN	NCBI
**1050**	PDCD1LG2	PD1L2_HUMAN	NCBI
**1051**	CANX	CALX_HUMAN	NCBI
**1052**	MAOB	AOFB_HUMAN	NCBI
**1053**	PGK1	PGK1_HUMAN	NCBI
**1054**	PPP1CA	PP1A_HUMAN	NCBI
**1055**	SMAD1	SMAD1_HUMAN	NCBI
**1056**	SLIT2	SLIT2_HUMAN	NCBI
**1057**	RPSA	RSSA_HUMAN	NCBI
**1058**	LAG3	LAG3_HUMAN	NCBI
**1059**	MSLN	MSLN_HUMAN	NCBI
**1060**	PTGER4	PE2R4_HUMAN	NCBI
**1061**	HPGD	PGDH_HUMAN	NCBI
**1062**	PYCARD	ASC_HUMAN	NCBI
**1063**	SLPI	SLPI_HUMAN	NCBI
**1064**	CCL4	CCL4_HUMAN	NCBI

No.	Symbol	Uniprot Name	Database
**1065**	ADH1C	ADH1G_HUMAN	NCBI
**1066**	ATF6	ATF6A_HUMAN	NCBI
**1067**	MSX1	MSX1_HUMAN	NCBI
**1068**	CAPN1	CAN1_HUMAN	NCBI
**1069**	MST1R	RON_HUMAN	NCBI
**1070**	POU2F1	PO2F1_HUMAN	NCBI
**1071**	BID	BID_HUMAN	NCBI
**1072**	LIN28B	LN28B_HUMAN	NCBI
**1073**	BRIP1	FANCJ_HUMAN	NCBI
**1074**	ANPEP	AMPN_HUMAN	NCBI
**1075**	RAB7A	RAB7A_HUMAN	NCBI
**1076**	MAP1LC3B	MLP3B_HUMAN	NCBI
**1077**	XRCC2	XRCC2_HUMAN	NCBI
**1078**	UGT2B7	UD2B7_HUMAN	NCBI
**1079**	PFKFB3	F263_HUMAN	NCBI
**1080**	TRAP1	TRAP1_HUMAN	NCBI
**1081**	KHDRBS1	KHDR1_HUMAN	NCBI
**1082**	GATA6	GATA6_HUMAN	NCBI
**1083**	USP22	UBP22_HUMAN	NCBI
**1084**	FLI1	FLI1_HUMAN	NCBI
**1085**	ABCB4	MDR3_HUMAN	NCBI
**1086**	SPOP	SPOP_HUMAN	NCBI
**1087**	FOSL1	FOSL1_HUMAN	NCBI
**1088**	PSCA	PSCA_HUMAN	NCBI
**1089**	MMP12	MMP12_HUMAN	NCBI
**1090**	EGLN1	EGLN1_HUMAN	NCBI
**1091**	TFF1	TFF1_HUMAN	NCBI
**1092**	LIN28A	LN28A_HUMAN	NCBI
**1093**	TRPM2	TRPM2_HUMAN	NCBI
**1094**	MERTK	MERTK_HUMAN	NCBI
**1095**	HSPA1B	HS71B_HUMAN	NCBI
**1096**	DLG1	DLG1_HUMAN	NCBI
**1097**	CEACAM6	CEAM6_HUMAN	NCBI
**1098**	BAG1	BAG1_HUMAN	NCBI
**1099**	PDGFA	PDGFA_HUMAN	NCBI
**1100**	GLS	GLSK_HUMAN	NCBI
**1101**	DEK	DEK_HUMAN	NCBI
**1102**	SFPQ	SFPQ_HUMAN	NCBI
**1103**	MAP2K4	MP2K4_HUMAN	NCBI
**1104**	AQP5	AQP5_HUMAN	NCBI
**1105**	CD74	HG2A_HUMAN	NCBI

No.	Symbol	Uniprot Name	Database
**1106**	KCNN4	KCNN4_HUMAN	NCBI
**1107**	TXNRD1	TRXR1_HUMAN	NCBI
**1108**	HOXA9	HXA9_HUMAN	NCBI
**1109**	DROSHA	RNC_HUMAN	NCBI
**1110**	CD80	CD80_HUMAN	NCBI
**1111**	SLCO1B3	SO1B3_HUMAN	NCBI
**1112**	TTK	TTK_HUMAN	NCBI
**1113**	CRKL	CRKL_HUMAN	NCBI
**1114**	C5AR1	C5AR1_HUMAN	NCBI
**1115**	GLI3	GLI3_HUMAN	NCBI
**1116**	LCAT	LCAT_HUMAN	NCBI
**1117**	SRSF2	SRSF2_HUMAN	NCBI
**1118**	CD63	CD63_HUMAN	NCBI
**1119**	PRLR	PRLR_HUMAN	NCBI
**1120**	BIN1	BIN1_HUMAN	NCBI
**1121**	EFEMP1	FBLN3_HUMAN	NCBI
**1122**	FOLR1	FOLR1_HUMAN	NCBI
**1123**	SMARCA2	SMCA2_HUMAN	NCBI
**1124**	S100P	S100P_HUMAN	NCBI
**1125**	KLF2	KLF2_HUMAN	NCBI
**1126**	PIK3CD	PK3CD_HUMAN	NCBI
**1127**	PVR	PVR_HUMAN	NCBI
**1128**	AKR1B10	AK1BA_HUMAN	NCBI
**1129**	TRIM25	TRI25_HUMAN	NCBI
**1130**	ELK1	ELK1_HUMAN	NCBI
**1131**	MGP	MGP_HUMAN	NCBI
**1132**	SLC22A5	S22A5_HUMAN	NCBI
**1133**	SERPINA12	SPA12_HUMAN	NCBI
**1134**	YWHAG	1433G_HUMAN	NCBI
**1135**	RUNX1T1	MTG8_HUMAN	NCBI
**1136**	PTGES	PTGES_HUMAN	NCBI
**1137**	GOLM1	GOLM1_HUMAN	NCBI
**1138**	SETD2	SETD2_HUMAN	NCBI
**1139**	GHSR	GHSR_HUMAN	NCBI
**1140**	TNFRSF6B	TNF6B_HUMAN	NCBI
**1141**	MICB	MICB_HUMAN	NCBI
**1142**	AGA	ASPG_HUMAN	NCBI
**1143**	TRIB3	TRIB3_HUMAN	NCBI
**1144**	NLRP1	NLRP1_HUMAN	NCBI
**1145**	DKC1	DKC1_HUMAN	NCBI
**1146**	ILF3	ILF3_HUMAN	NCBI

No.	Symbol	Uniprot Name	Database
**1147**	FGF7	FGF7_HUMAN	NCBI
**1148**	NONO	NONO_HUMAN	NCBI
**1149**	MAPK9	MK09_HUMAN	NCBI
**1150**	PER2	PER2_HUMAN	NCBI
**1151**	FPR2	FPR2_HUMAN	NCBI
**1152**	GLUD1	DHE3_HUMAN	NCBI
**1153**	FOXC2	FOXC2_HUMAN	NCBI
**1154**	MS4A1	CD20_HUMAN	NCBI
**1155**	XDH	XDH_HUMAN	NCBI
**1156**	ACVRL1	ACVL1_HUMAN	NCBI
**1157**	FOXA2	FOXA2_HUMAN	NCBI
**1158**	RAB11A	RB11A_HUMAN	NCBI
**1159**	STK4	STK4_HUMAN	NCBI
**1160**	VASP	VASP_HUMAN	NCBI
**1161**	TRPC1	TRPC1_HUMAN	NCBI
**1162**	PROX1	PROX1_HUMAN	NCBI
**1163**	AZGP1	ZA2G_HUMAN	NCBI
**1164**	TAS2R38	T2R38_HUMAN	NCBI
**1165**	PLD1	PLD1_HUMAN	NCBI
**1166**	NEK2	NEK2_HUMAN	NCBI
**1167**	NRP2	NRP2_HUMAN	NCBI
**1168**	HAX1	HAX1_HUMAN	NCBI
**1169**	CTBP1	CTBP1_HUMAN	NCBI
**1170**	HNRNPU	HNRPU_HUMAN	NCBI
**1171**	ICOS	ICOS_HUMAN	NCBI
**1172**	NCOA2	NCOA2_HUMAN	NCBI
**1173**	COL5A1	CO5A1_HUMAN	NCBI
**1174**	LIMA1	LIMA1_HUMAN	NCBI
**1175**	MAP2K2	MP2K2_HUMAN	NCBI
**1176**	PODXL	PODXL_HUMAN	NCBI
**1177**	ZBTB16	ZBT16_HUMAN	NCBI
**1178**	NOTCH4	NOTC4_HUMAN	NCBI
**1179**	NNMT	NNMT_HUMAN	NCBI
**1180**	PIK3CB	PK3CB_HUMAN	NCBI
**1181**	CASP7	CASP7_HUMAN	NCBI
**1182**	CCL18	CCL18_HUMAN	NCBI
**1183**	EXO1	EXO1_HUMAN	NCBI
**1184**	ALOX12	LOX12_HUMAN	NCBI
**1185**	SNCG	SYUG_HUMAN	NCBI
**1186**	SIGMAR1	SGMR1_HUMAN	NCBI
**1187**	CGA	GLHA_HUMAN	NCBI

No.	Symbol	Uniprot Name	Database
**1188**	TLN1	TLN1_HUMAN	NCBI
**1189**	BACH1	BACH1_HUMAN	NCBI
**1190**	KDM5B	KDM5B_HUMAN	NCBI
**1191**	PTPN2	PTN2_HUMAN	NCBI
**1192**	RORA	RORA_HUMAN	NCBI
**1193**	LILRB1	LIRB1_HUMAN	NCBI
**1194**	TLR1	TLR1_HUMAN	NCBI
**1195**	RICTOR	RICTR_HUMAN	NCBI
**1196**	BCL3	BCL3_HUMAN	NCBI
**1197**	PAK2	PAK2_HUMAN	NCBI
**1198**	DHFR	DYR_HUMAN	NCBI
**1199**	SETDB1	SETB1_HUMAN	NCBI
**1200**	CCL21	CCL21_HUMAN	NCBI
**1201**	THBS2	TSP2_HUMAN	NCBI
**1202**	TNFRSF9	TNR9_HUMAN	NCBI
**1203**	CLDN4	CLD4_HUMAN	NCBI
**1204**	TPT1	TCTP_HUMAN	NCBI
**1205**	SMC1A	SMC1A_HUMAN	NCBI
**1206**	HOXB13	HXB13_HUMAN	NCBI
**1207**	PBK	TOPK_HUMAN	NCBI
**1208**	CHIT1	CHIT1_HUMAN	NCBI
**1209**	PRDX6	PRDX6_HUMAN	NCBI
**1210**	THPO	TPO_HUMAN	NCBI
**1211**	MSH3	MSH3_HUMAN	NCBI
**1212**	IFNAR1	INAR1_HUMAN	NCBI
**1213**	RRM2	RIR2_HUMAN	NCBI
**1214**	KDM6A	KDM6A_HUMAN	NCBI
**1215**	IGFBP5	IBP5_HUMAN	NCBI
**1216**	STC1	STC1_HUMAN	NCBI
**1217**	PSME3	PSME3_HUMAN	NCBI
**1218**	RAB27A	RB27A_HUMAN	NCBI
**1219**	MYBL2	MYBB_HUMAN	NCBI
**1220**	NUMA1	NUMA1_HUMAN	NCBI
**1221**	ZBTB7A	ZBT7A_HUMAN	NCBI
**1222**	STAT2	STAT2_HUMAN	NCBI
**1223**	HCK	HCK_HUMAN	NCBI
**1224**	ALDOA	ALDOA_HUMAN	NCBI
**1225**	WIF1	WIF1_HUMAN	NCBI
**1226**	CSNK2A2	CSK22_HUMAN	NCBI
**1227**	RBPJ	SUH_HUMAN	NCBI
**1228**	KDM6B	KDM6B_HUMAN	NCBI

No.	Symbol	Uniprot Name	Database
**1229**	KRT20	K1C20_HUMAN	NCBI
**1230**	MAX	MAX_HUMAN	NCBI
**1231**	UNG	UNG_HUMAN	NCBI
**1232**	KRT17	K1C17_HUMAN	NCBI
**1233**	HSD17B1	DHB1_HUMAN	NCBI
**1234**	CD151	CD151_HUMAN	NCBI
**1235**	DMBT1	DMBT1_HUMAN	NCBI
**1236**	TIGIT	TIGIT_HUMAN	NCBI
**1237**	MYOD1	MYOD1_HUMAN	NCBI
**1238**	SERPINH1	SERPH_HUMAN	NCBI
**1239**	SUMO2	SUMO2_HUMAN	NCBI
**1240**	RUVBL1	RUVB1_HUMAN	NCBI
**1241**	MFGE8	MFGM_HUMAN	NCBI
**1242**	TERF1	TERF1_HUMAN	NCBI
**1243**	PLD2	PLD2_HUMAN	NCBI
**1244**	NSD2	NSD2_HUMAN	NCBI
**1245**	COL4A3	CO4A3_HUMAN	NCBI
**1246**	TNFRSF4	TNR4_HUMAN	NCBI
**1247**	PRKAA2	AAPK2_HUMAN	NCBI
**1248**	F11R	JAM1_HUMAN	NCBI
**1249**	BIRC7	BIRC7_HUMAN	NCBI
**1250**	GPR55	GPR55_HUMAN	NCBI
**1251**	ROR1	ROR1_HUMAN	NCBI
**1252**	NCK1	NCK1_HUMAN	NCBI
**1253**	SELENOP	SEPP1_HUMAN	NCBI
**1254**	FZR1	FZR1_HUMAN	NCBI
**1255**	ST6GAL1	SIAT1_HUMAN	NCBI
**1256**	CEMIP	CEMIP_HUMAN	NCBI
**1257**	PAWR	PAWR_HUMAN	NCBI
**1258**	TK1	KITH_HUMAN	NCBI
**1259**	COL17A1	COHA1_HUMAN	NCBI
**1260**	SMO	SMO_HUMAN	NCBI
**1261**	PAK4	PAK4_HUMAN	NCBI
**1262**	FPR1	FPR1_HUMAN	NCBI
**1263**	VEGFD	VEGFD_HUMAN	NCBI
**1264**	OAS1	OAS1_HUMAN	NCBI
**1265**	RRM1	RIR1_HUMAN	NCBI
**1266**	SMURF2	SMUF2_HUMAN	NCBI
**1267**	CARM1	CARM1_HUMAN	NCBI
**1268**	PCBP1	PCBP1_HUMAN	NCBI
**1269**	VIP	VIP_HUMAN	NCBI

No.	Symbol	Uniprot Name	Database
**1270**	TMSB4X	TYB4_HUMAN	NCBI
**1271**	EIF4A3	IF4A3_HUMAN	NCBI
**1272**	ALKBH5	ALKB5_HUMAN	NCBI
**1273**	CD8A	CD8A_HUMAN	NCBI
**1274**	EIF5A	IF5A1_HUMAN	NCBI
**1275**	HMMR	HMMR_HUMAN	NCBI
**1276**	SOX11	SOX11_HUMAN	NCBI
**1277**	DLL4	DLL4_HUMAN	NCBI
**1278**	RPL18	RL18_HUMAN	NCBI
**1279**	FGF19	FGF19_HUMAN	NCBI
**1280**	MAPRE1	MARE1_HUMAN	NCBI
**1281**	GAB2	GAB2_HUMAN	NCBI
**1282**	CD226	CD226_HUMAN	NCBI
**1283**	COL11A1	COBA1_HUMAN	NCBI
**1284**	UGT1A9	UD19_HUMAN	NCBI
**1285**	PCBP2	PCBP2_HUMAN	NCBI
**1286**	EFNB2	EFNB2_HUMAN	NCBI
**1287**	CHD4	CHD4_HUMAN	NCBI
**1288**	NEDD8	NEDD8_HUMAN	NCBI
**1289**	BMPR1A	BMR1A_HUMAN	NCBI
**1290**	RGS2	RGS2_HUMAN	NCBI
**1291**	RPTOR	RPTOR_HUMAN	NCBI
**1292**	TRPV6	TRPV6_HUMAN	NCBI
**1293**	PTGER2	PE2R2_HUMAN	NCBI
**1294**	SUZ12	SUZ12_HUMAN	NCBI
**1295**	RBBP4	RBBP4_HUMAN	NCBI
**1296**	FOXE1	FOXE1_HUMAN	NCBI
**1297**	BGN	PGS1_HUMAN	NCBI
**1298**	IFNA2	IFNA2_HUMAN	NCBI
**1299**	CUL4B	CUL4B_HUMAN	NCBI
**1300**	RPS3	RS3_HUMAN	NCBI
**1301**	NR2F2	COT2_HUMAN	NCBI
**1302**	CSNK1A1	KC1A_HUMAN	NCBI
**1303**	NUDT1	8ODP_HUMAN	NCBI
**1304**	DDX21	DDX21_HUMAN	NCBI
**1305**	PKLR	KPYR_HUMAN	NCBI
**1306**	HTRA2	HTRA2_HUMAN	NCBI
**1307**	SDC4	SDC4_HUMAN	NCBI
**1308**	GAST	GAST_HUMAN	NCBI
**1309**	ANGPTL3	ANGL3_HUMAN	NCBI
**1310**	CLDN7	CLD7_HUMAN	NCBI

No.	Symbol	Uniprot Name	Database
**1311**	KIF2C	KIF2C_HUMAN	NCBI
**1312**	MT2A	MT2_HUMAN	NCBI
**1313**	CBX3	CBX3_HUMAN	NCBI
**1314**	PSMD10	PSD10_HUMAN	NCBI
**1315**	ADAM9	ADAM9_HUMAN	NCBI
**1316**	OLFM4	OLFM4_HUMAN	NCBI
**1317**	PMAIP1	APR_HUMAN	NCBI
**1318**	LRG1	A2GL_HUMAN	NCBI
**1319**	MT-ND1	NU1M_HUMAN	NCBI
**1320**	ATP6AP2	RENR_HUMAN	NCBI
**1321**	TRAF1	TRAF1_HUMAN	NCBI
**1322**	SENP1	SENP1_HUMAN	NCBI
**1323**	SULT2B1	ST2B1_HUMAN	NCBI
**1324**	ROR2	ROR2_HUMAN	NCBI
**1325**	CDH3	CADH3_HUMAN	NCBI
**1326**	NR1H2	NR1H2_HUMAN	NCBI
**1327**	PNPLA2	PLPL2_HUMAN	NCBI
**1328**	SCNN1B	SCNNB_HUMAN	NCBI
**1329**	CES1	EST1_HUMAN	NCBI
**1330**	MSI1	MSI1H_HUMAN	NCBI
**1331**	CYCS	CYC_HUMAN	NCBI
**1332**	GAL	GALA_HUMAN	NCBI
**1333**	RORC	RORG_HUMAN	NCBI
**1334**	RIPK2	RIPK2_HUMAN	NCBI
**1335**	RAB1A	RAB1A_HUMAN	NCBI
**1336**	RPS6	RS6_HUMAN	NCBI
**1337**	SLC25A13	S2513_HUMAN	NCBI
**1338**	MASP2	MASP2_HUMAN	NCBI
**1339**	KAT2A	KAT2A_HUMAN	NCBI
**1340**	DOT1L	DOT1L_HUMAN	NCBI
**1341**	ACLY	ACLY_HUMAN	NCBI
**1342**	GOLPH3	GOLP3_HUMAN	NCBI
**1343**	ACTC1	ACTC_HUMAN	NCBI
**1344**	ST14	ST14_HUMAN	NCBI
**1345**	RAD52	RAD52_HUMAN	NCBI
**1346**	HDAC5	HDAC5_HUMAN	NCBI
**1347**	SLC2A3	GTR3_HUMAN	NCBI
**1348**	YTHDF2	YTHD2_HUMAN	NCBI
**1349**	LATS1	LATS1_HUMAN	NCBI
**1350**	RELB	RELB_HUMAN	NCBI
**1351**	TCN2	TCO2_HUMAN	NCBI

No.	Symbol	Uniprot Name	Database
**1352**	CXCR5	CXCR5_HUMAN	NCBI
**1353**	INPPL1	SHIP2_HUMAN	NCBI
**1354**	KCNA3	KCNA3_HUMAN	NCBI
**1355**	KRT7	K2C7_HUMAN	NCBI
**1356**	AMACR	AMACR_HUMAN	NCBI
**1357**	UBR5	UBR5_HUMAN	NCBI
**1358**	E2F4	E2F4_HUMAN	NCBI
**1359**	CTSG	CATG_HUMAN	NCBI
**1360**	ATG7	ATG7_HUMAN	NCBI
**1361**	CHFR	CHFR_HUMAN	NCBI
**1362**	CLIC1	CLIC1_HUMAN	NCBI
**1363**	CCK	CCKN_HUMAN	NCBI
**1364**	MYO6	MYO6_HUMAN	NCBI
**1365**	FABP5	FABP5_HUMAN	NCBI
**1366**	FUT4	FUT4_HUMAN	NCBI
**1367**	PAFAH1B1	LIS1_HUMAN	NCBI
**1368**	HNRNPL	HNRPL_HUMAN	NCBI
**1369**	GDF2	GDF2_HUMAN	NCBI
**1370**	IRAK4	IRAK4_HUMAN	NCBI
**1371**	CDC6	CDC6_HUMAN	NCBI
**1372**	EIF4A1	IF4A1_HUMAN	NCBI
**1373**	METTL14	MET14_HUMAN	NCBI
**1374**	NODAL	NODAL_HUMAN	NCBI
**1375**	MCC	CRCM_HUMAN	NCBI
**1376**	DDC	DDC_HUMAN	NCBI
**1377**	ACP1	PPAC_HUMAN	NCBI
**1378**	CTHRC1	CTHR1_HUMAN	NCBI
**1379**	LIPG	LIPG_HUMAN	NCBI
**1380**	HK1	HXK1_HUMAN	NCBI
**1381**	SEMA4D	SEM4D_HUMAN	NCBI
**1382**	CYP27A1	CP27A_HUMAN	NCBI
**1383**	KLK6	KLK6_HUMAN	NCBI
**1384**	SDCBP	SDCB1_HUMAN	NCBI
**1385**	SLC22A2	S22A2_HUMAN	NCBI
**1386**	STAU1	STAU1_HUMAN	NCBI
**1387**	DPP6	DPP6_HUMAN	NCBI
**1388**	IMPDH2	IMDH2_HUMAN	NCBI
**1389**	ETV4	ETV4_HUMAN	NCBI
**1390**	ARHGDIA	GDIR1_HUMAN	NCBI
**1391**	CUL5	CUL5_HUMAN	NCBI
**1392**	NTS	NEUT_HUMAN	NCBI

No.	Symbol	Uniprot Name	Database
**1393**	HHEX	HHEX_HUMAN	NCBI
**1394**	LY96	LY96_HUMAN	NCBI
**1395**	RECQL4	RECQ4_HUMAN	NCBI
**1396**	RHOB	RHOB_HUMAN	NCBI
**1397**	SESN2	SESN2_HUMAN	NCBI
**1398**	CTSC	CATC_HUMAN	NCBI
**1399**	SPTAN1	SPTN1_HUMAN	NCBI
**1400**	MED1	MED1_HUMAN	NCBI
**1401**	TAGLN	TAGL_HUMAN	NCBI
**1402**	ITGA1	ITA1_HUMAN	NCBI
**1403**	PLIN2	PLIN2_HUMAN	NCBI
**1404**	LAMB3	LAMB3_HUMAN	NCBI
**1405**	SULF1	SULF1_HUMAN	NCBI
**1406**	DIAPH1	DIAP1_HUMAN	NCBI
**1407**	BCOR	BCOR_HUMAN	NCBI
**1408**	MAP3K1	M3K1_HUMAN	NCBI
**1409**	FOXF1	FOXF1_HUMAN	NCBI
**1410**	SND1	SND1_HUMAN	NCBI
**1411**	PBRM1	PB1_HUMAN	NCBI
**1412**	CDK7	CDK7_HUMAN	NCBI
**1413**	CCR1	CCR1_HUMAN	NCBI
**1414**	PPP1R13L	IASPP_HUMAN	NCBI
**1415**	CAMK2G	KCC2G_HUMAN	NCBI
**1416**	S100A10	S10AA_HUMAN	NCBI
**1417**	CCDC88A	GRDN_HUMAN	NCBI
**1418**	MEF2A	MEF2A_HUMAN	NCBI
**1419**	PHGDH	SERA_HUMAN	NCBI
**1420**	CXCL14	CXL14_HUMAN	NCBI
**1421**	AKAP12	AKA12_HUMAN	NCBI
**1422**	SERPINB3	SPB3_HUMAN	NCBI
**1423**	CSNK1E	KC1E_HUMAN	NCBI
**1424**	SSRP1	SSRP1_HUMAN	NCBI
**1425**	TOPBP1	TOPB1_HUMAN	NCBI
**1426**	RAD18	RAD18_HUMAN	NCBI
**1427**	STC2	STC2_HUMAN	NCBI
**1428**	SUV39H1	SUV91_HUMAN	NCBI
**1429**	SOAT1	SOAT1_HUMAN	NCBI
**1430**	DDB2	DDB2_HUMAN	NCBI
**1431**	ORM1	A1AG1_HUMAN	NCBI
**1432**	STIP1	STIP1_HUMAN	NCBI
**1433**	MAP3K14	M3K14_HUMAN	NCBI

No.	Symbol	Uniprot Name	Database
**1434**	MVP	MVP_HUMAN	NCBI
**1435**	PTPN13	PTN13_HUMAN	NCBI
**1436**	SLC5A1	SC5A1_HUMAN	NCBI
**1437**	DDR2	DDR2_HUMAN	NCBI
**1438**	ODC1	DCOR_HUMAN	NCBI
**1439**	CDCP1	CDCP1_HUMAN	NCBI
**1440**	FBP1	F16P1_HUMAN	NCBI
**1441**	USP14	UBP14_HUMAN	NCBI
**1442**	HDGF	HDGF_HUMAN	NCBI
**1443**	DDX17	DDX17_HUMAN	NCBI
**1444**	MARCKS	MARCS_HUMAN	NCBI
**1445**	LIMK1	LIMK1_HUMAN	NCBI
**1446**	MCM3	MCM3_HUMAN	NCBI
**1447**	DUOX2	DUOX2_HUMAN	NCBI
**1448**	RNF2	RING2_HUMAN	NCBI
**1449**	CTNNBIP1	CNBP1_HUMAN	NCBI
**1450**	KDM3A	KDM3A_HUMAN	NCBI
**1451**	CCL22	CCL22_HUMAN	NCBI
**1452**	RHEB	RHEB_HUMAN	NCBI
**1453**	MAP3K8	M3K8_HUMAN	NCBI
**1454**	GSDMD	GSDMD_HUMAN	NCBI
**1455**	ADORA2B	AA2BR_HUMAN	NCBI
**1456**	GCLC	GSH1_HUMAN	NCBI
**1457**	SLC22A4	S22A4_HUMAN	NCBI
**1458**	CSE1L	XPO2_HUMAN	NCBI
**1459**	POLD3	DPOD3_HUMAN	NCBI
**1460**	RAD21	RAD21_HUMAN	NCBI
**1461**	EIF3A	EIF3A_HUMAN	NCBI
**1462**	CPT2	CPT2_HUMAN	NCBI
**1463**	SCRIB	SCRIB_HUMAN	NCBI
**1464**	SLC11A2	NRAM2_HUMAN	NCBI
**1465**	SLC1A1	EAA3_HUMAN	NCBI
**1466**	ADIPOR2	PAQR2_HUMAN	NCBI
**1467**	MSI2	MSI2H_HUMAN	NCBI
**1468**	SMARCA5	SMCA5_HUMAN	NCBI
**1469**	ADAMTS5	ATS5_HUMAN	NCBI
**1470**	DAB2IP	DAB2P_HUMAN	NCBI
**1471**	ECE1	ECE1_HUMAN	NCBI
**1472**	FUT8	FUT8_HUMAN	NCBI
**1473**	CYP2R1	CP2R1_HUMAN	NCBI
**1474**	FLOT1	FLOT1_HUMAN	NCBI

No.	Symbol	Uniprot Name	Database
**1475**	FUT6	FUT6_HUMAN	NCBI
**1476**	SIRPA	SHPS1_HUMAN	NCBI
**1477**	KISS1R	KISSR_HUMAN	NCBI
**1478**	RAD54B	RA54B_HUMAN	NCBI
**1479**	EEF1A2	EF1A2_HUMAN	NCBI
**1480**	SIRT7	SIR7_HUMAN	NCBI
**1481**	NR2C2	NR2C2_HUMAN	NCBI
**1482**	HNRNPH1	HNRH1_HUMAN	NCBI
**1483**	DPYSL2	DPYL2_HUMAN	NCBI
**1484**	ARID1B	ARI1B_HUMAN	NCBI
**1485**	LAMC1	LAMC1_HUMAN	NCBI
**1486**	ACSL4	ACSL4_HUMAN	NCBI
**1487**	DVL2	DVL2_HUMAN	NCBI
**1488**	TUBA1B	TBA1B_HUMAN	NCBI
**1489**	PHB2	PHB2_HUMAN	NCBI
**1490**	ATP13A2	AT132_HUMAN	NCBI
**1491**	BHLHE40	BHE40_HUMAN	NCBI
**1492**	BTG2	BTG2_HUMAN	NCBI
**1493**	AIMP1	AIMP1_HUMAN	NCBI
**1494**	IL17RA	I17RA_HUMAN	NCBI
**1495**	FXR1	FXR1_HUMAN	NCBI
**1496**	WTAP	FL2D_HUMAN	NCBI
**1497**	RNF43	RNF43_HUMAN	NCBI
**1498**	QKI	QKI_HUMAN	NCBI
**1499**	DCK	DCK_HUMAN	NCBI
**1500**	FKBP4	FKBP4_HUMAN	NCBI
**1501**	ZMIZ1	ZMIZ1_HUMAN	NCBI
**1502**	RACGAP1	RGAP1_HUMAN	NCBI
**1503**	KMT2C	KMT2C_HUMAN	NCBI
**1504**	HNRNPM	HNRPM_HUMAN	NCBI
**1505**	TNFAIP6	TSG6_HUMAN	NCBI
**1506**	SLC16A4	MOT5_HUMAN	NCBI
**1507**	CBFB	PEBB_HUMAN	NCBI
**1508**	INHBB	INHBB_HUMAN	NCBI
**1509**	ECT2	ECT2_HUMAN	NCBI
**1510**	CUX1	CUX1_HUMAN	NCBI
**1511**	CUX1	CASP_HUMAN	NCBI
**1512**	TEAD4	TEAD4_HUMAN	NCBI
**1513**	CSPG4	CSPG4_HUMAN	NCBI
**1514**	ABCC3	MRP3_HUMAN	NCBI
**1515**	PLS3	PLST_HUMAN	NCBI

No.	Symbol	Uniprot Name	Database
**1516**	AOC3	AOC3_HUMAN	NCBI
**1517**	MMP11	MMP11_HUMAN	NCBI
**1518**	NDUFA13	NDUAD_HUMAN	NCBI
**1519**	ID4	ID4_HUMAN	NCBI
**1520**	ING1	ING1_HUMAN	NCBI
**1521**	MTAP	MTAP_HUMAN	NCBI
**1522**	ITGB6	ITB6_HUMAN	NCBI
**1523**	KCNH1	KCNH1_HUMAN	NCBI
**1524**	LAPTM4B	LAP4B_HUMAN	NCBI
**1525**	HSPB8	HSPB8_HUMAN	NCBI
**1526**	SAT1	SAT1_HUMAN	NCBI
**1527**	LUM	LUM_HUMAN	NCBI
**1528**	HOXB7	HXB7_HUMAN	NCBI
**1529**	SIRT5	SIR5_HUMAN	NCBI
**1530**	CTBP2	CTBP2_HUMAN	NCBI
**1531**	MCM6	MCM6_HUMAN	NCBI
**1532**	FOXQ1	FOXQ1_HUMAN	NCBI
**1533**	S100A11	S10AB_HUMAN	NCBI
**1534**	FOXO4	FOXO4_HUMAN	NCBI
**1535**	SOCS2	SOCS2_HUMAN	NCBI
**1536**	TACC3	TACC3_HUMAN	NCBI
**1537**	BAG6	BAG6_HUMAN	NCBI
**1538**	REG4	REG4_HUMAN	NCBI
**1539**	BCAP31	BAP31_HUMAN	NCBI
**1540**	EPB41L2	E41L2_HUMAN	NCBI
**1541**	MTNR1A	MTR1A_HUMAN	NCBI
**1542**	SHMT2	GLYM_HUMAN	NCBI
**1543**	TIA1	TIA1_HUMAN	NCBI
**1544**	SMYD3	SMYD3_HUMAN	NCBI
**1545**	PTK7	PTK7_HUMAN	NCBI
**1546**	ETS2	ETS2_HUMAN	NCBI
**1547**	NPC1L1	NPCL1_HUMAN	NCBI
**1548**	CCAR2	CCAR2_HUMAN	NCBI
**1549**	TNIP1	TNIP1_HUMAN	NCBI
**1550**	MBNL1	MBNL1_HUMAN	NCBI
**1551**	KHSRP	FUBP2_HUMAN	NCBI
**1552**	PIWIL1	PIWL1_HUMAN	NCBI
**1553**	ACACA	ACACA_HUMAN	NCBI
**1554**	ASPM	ASPM_HUMAN	NCBI
**1555**	TNKS	TNKS1_HUMAN	NCBI
**1556**	SCARB2	SCRB2_HUMAN	NCBI

No.	Symbol	Uniprot Name	Database
**1557**	SUCLA2	SUCB1_HUMAN	NCBI
**1558**	SLC22A3	S22A3_HUMAN	NCBI
**1559**	UGT1A6	UD16_HUMAN	NCBI
**1560**	PER1	PER1_HUMAN	NCBI
**1561**	IFITM1	IFM1_HUMAN	NCBI
**1562**	SETD7	SETD7_HUMAN	NCBI
**1563**	PARD3	PARD3_HUMAN	NCBI
**1564**	PDK1	PDK1_HUMAN	NCBI
**1565**	ANGPTL2	ANGL2_HUMAN	NCBI
**1566**	TRIM24	TIF1A_HUMAN	NCBI
**1567**	DNAJA1	DNJA1_HUMAN	NCBI
**1568**	ZC3H12A	ZC12A_HUMAN	NCBI
**1569**	ATP2B4	AT2B4_HUMAN	NCBI
**1570**	NDC80	NDC80_HUMAN	NCBI
**1571**	ELF3	ELF3_HUMAN	NCBI
**1572**	ADORA3	AA3R_HUMAN	NCBI
**1573**	SOX17	SOX17_HUMAN	NCBI
**1574**	FBLN5	FBLN5_HUMAN	NCBI
**1575**	EGFL7	EGFL7_HUMAN	NCBI
**1576**	USP10	UBP10_HUMAN	NCBI
**1577**	FBLN1	FBLN1_HUMAN	NCBI
**1578**	GSDME	GSDME_HUMAN	NCBI
**1579**	TPSAB1	TRYB1_HUMAN	NCBI
**1580**	DNASE1	DNAS1_HUMAN	NCBI
**1581**	PSMD2	PSMD2_HUMAN	NCBI
**1582**	NTSR1	NTR1_HUMAN	NCBI
**1583**	DUSP4	DUS4_HUMAN	NCBI
**1584**	EIF2AK4	E2AK4_HUMAN	NCBI
**1585**	EGLN3	EGLN3_HUMAN	NCBI
**1586**	TAPBP	TPSN_HUMAN	NCBI
**1587**	SLC12A2	S12A2_HUMAN	NCBI
**1588**	NEIL1	NEIL1_HUMAN	NCBI
**1589**	AHCY	SAHH_HUMAN	NCBI
**1590**	TLR10	TLR10_HUMAN	NCBI
**1591**	PROZ	PROZ_HUMAN	NCBI
**1592**	CCR4	CCR4_HUMAN	NCBI
**1593**	MBD2	MBD2_HUMAN	NCBI
**1594**	DDX39B	DX39B_HUMAN	NCBI
**1595**	REG1A	REG1A_HUMAN	NCBI
**1596**	PKHD1	PKHD1_HUMAN	NCBI
**1597**	GRP	GRP_HUMAN	NCBI

No.	Symbol	Uniprot Name	Database
**1598**	INSIG2	INSI2_HUMAN	NCBI
**1599**	PRKAR2A	KAP2_HUMAN	NCBI
**1600**	PTMA	PTMA_HUMAN	NCBI
**1601**	SPHK2	SPHK2_HUMAN	NCBI
**1602**	MLXIPL	MLXPL_HUMAN	NCBI
**1603**	F2RL3	PAR4_HUMAN	NCBI
**1604**	APOD	APOD_HUMAN	NCBI
**1605**	LIFR	LIFR_HUMAN	NCBI
**1606**	RAD23B	RD23B_HUMAN	NCBI
**1607**	MCM5	MCM5_HUMAN	NCBI
**1608**	IL1F10	IL1FA_HUMAN	NCBI
**1609**	MTA2	MTA2_HUMAN	NCBI
**1610**	TRIP13	PCH2_HUMAN	NCBI
**1611**	UBD	UBD_HUMAN	NCBI
**1612**	NRIP1	NRIP1_HUMAN	NCBI
**1613**	FAT1	FAT1_HUMAN	NCBI
**1614**	DDX6	DDX6_HUMAN	NCBI
**1615**	SLC4A4	S4A4_HUMAN	NCBI
**1616**	DDIT4	DDIT4_HUMAN	NCBI
**1617**	KLRC1	NKG2A_HUMAN	NCBI
**1618**	S100A2	S10A2_HUMAN	NCBI
**1619**	CASK	CSKP_HUMAN	NCBI
**1620**	EREG	EREG_HUMAN	NCBI
**1621**	SLC46A1	PCFT_HUMAN	NCBI
**1622**	KCNN3	KCNN3_HUMAN	NCBI
**1623**	IL13RA2	I13R2_HUMAN	NCBI
**1624**	EYA1	EYA1_HUMAN	NCBI
**1625**	CLDN3	CLD3_HUMAN	NCBI
**1626**	DDX1	DDX1_HUMAN	NCBI
**1627**	SLC15A1	S15A1_HUMAN	NCBI
**1628**	VLDLR	VLDLR_HUMAN	NCBI
**1629**	DVL1	DVL1_HUMAN	NCBI
**1630**	AQP9	AQP9_HUMAN	NCBI
**1631**	CELF1	CELF1_HUMAN	NCBI
**1632**	FZD7	FZD7_HUMAN	NCBI
**1633**	MAGEA3	MAGA3_HUMAN	NCBI
**1634**	MAD1L1	MD1L1_HUMAN	NCBI
**1635**	KDM4B	KDM4B_HUMAN	NCBI
**1636**	ANOS1	KALM_HUMAN	NCBI
**1637**	TYRO3	TYRO3_HUMAN	NCBI
**1638**	FGF9	FGF9_HUMAN	NCBI

No.	Symbol	Uniprot Name	Database
**1639**	RALA	RALA_HUMAN	NCBI
**1640**	HAS2	HYAS2_HUMAN	NCBI
**1641**	VTI1A	VTI1A_HUMAN	NCBI
**1642**	COL10A1	COAA1_HUMAN	NCBI
**1643**	CASP10	CASPA_HUMAN	NCBI
**1644**	PELP1	PELP1_HUMAN	NCBI
**1645**	GNA13	GNA13_HUMAN	NCBI
**1646**	PPP2R2A	2ABA_HUMAN	NCBI
**1647**	CLDN2	CLD2_HUMAN	NCBI
**1648**	FZD4	FZD4_HUMAN	NCBI
**1649**	IL25	IL25_HUMAN	NCBI
**1650**	KLK10	KLK10_HUMAN	NCBI
**1651**	NUPR1	NUPR1_HUMAN	NCBI
**1652**	CDK12	CDK12_HUMAN	NCBI
**1653**	SYNCRIP	HNRPQ_HUMAN	NCBI
**1654**	ACVR2A	AVR2A_HUMAN	NCBI
**1655**	ERO1A	ERO1A_HUMAN	NCBI
**1656**	NAT10	NAT10_HUMAN	NCBI
**1657**	NLRP7	NALP7_HUMAN	NCBI
**1658**	STOML2	STML2_HUMAN	NCBI
**1659**	NFYA	NFYA_HUMAN	NCBI
**1660**	HDAC7	HDAC7_HUMAN	NCBI
**1661**	UGT1A7	UD17_HUMAN	NCBI
**1662**	CYSLTR1	CLTR1_HUMAN	NCBI
**1663**	GUCY2C	GUC2C_HUMAN	NCBI
**1664**	HNRNPF	HNRPF_HUMAN	NCBI
**1665**	PSMA7	PSA7_HUMAN	NCBI
**1666**	TIMELESS	TIM_HUMAN	NCBI
**1667**	KCNA1	KCNA1_HUMAN	NCBI
**1668**	USP15	UBP15_HUMAN	NCBI
**1669**	PLSCR1	PLS1_HUMAN	NCBI
**1670**	ASNS	ASNS_HUMAN	NCBI
**1671**	CRY1	CRY1_HUMAN	NCBI
**1672**	OTUB1	OTUB1_HUMAN	NCBI
**1673**	IL36G	IL36G_HUMAN	NCBI
**1674**	ESRRG	ERR3_HUMAN	NCBI
**1675**	TBL1XR1	TBL1R_HUMAN	NCBI
**1676**	TGIF1	TGIF1_HUMAN	NCBI
**1677**	WNT4	WNT4_HUMAN	NCBI
**1678**	ANXA3	ANXA3_HUMAN	NCBI
**1679**	KMT5A	KMT5A_HUMAN	NCBI

No.	Symbol	Uniprot Name	Database
**1680**	HMGB2	HMGB2_HUMAN	NCBI
**1681**	PRKRA	PRKRA_HUMAN	NCBI
**1682**	TNFSF9	TNFL9_HUMAN	NCBI
**1683**	AMFR	AMFR_HUMAN	NCBI
**1684**	LTC4S	LTC4S_HUMAN	NCBI
**1685**	MYRF	MYRF_HUMAN	NCBI
**1686**	PLAGL1	PLAL1_HUMAN	NCBI
**1687**	CCT3	TCPG_HUMAN	NCBI
**1688**	SYP	SYPH_HUMAN	NCBI
**1689**	TDP1	TYDP1_HUMAN	NCBI
**1690**	PTGIS	PTGIS_HUMAN	NCBI
**1691**	MELK	MELK_HUMAN	NCBI
**1692**	LAMB1	LAMB1_HUMAN	NCBI
**1693**	WNT7A	WNT7A_HUMAN	NCBI
**1694**	CXCL2	CXCL2_HUMAN	NCBI
**1695**	KDM5A	KDM5A_HUMAN	NCBI
**1696**	ACTL6A	ACL6A_HUMAN	NCBI
**1697**	HAO1	HAOX1_HUMAN	NCBI
**1698**	TGFB1I1	TGFI1_HUMAN	NCBI
**1699**	PDK4	PDK4_HUMAN	NCBI
**1700**	SH2B3	SH2B3_HUMAN	NCBI
**1701**	MYH11	MYH11_HUMAN	NCBI
**1702**	MAFB	MAFB_HUMAN	NCBI
**1703**	ARID5B	ARI5B_HUMAN	NCBI
**1704**	GJA5	CXA5_HUMAN	NCBI
**1705**	SNAP23	SNP23_HUMAN	NCBI
**1706**	SMARCE1	SMCE1_HUMAN	NCBI
**1707**	ST8SIA2	SIA8B_HUMAN	NCBI
**1708**	XAF1	XAF1_HUMAN	NCBI
**1709**	GDF11	GDF11_HUMAN	NCBI
**1710**	CEBPD	CEBPD_HUMAN	NCBI
**1711**	KIF4A	KIF4A_HUMAN	NCBI
**1712**	TNFAIP8L2	TP8L2_HUMAN	NCBI
**1713**	PLOD2	PLOD2_HUMAN	NCBI
**1714**	FUBP1	FUBP1_HUMAN	NCBI
**1715**	CRLF2	CRLF2_HUMAN	NCBI
**1716**	YTHDF1	YTHD1_HUMAN	NCBI
**1717**	TDG	TDG_HUMAN	NCBI
**1718**	AHNAK	AHNK_HUMAN	NCBI
**1719**	ADGRG1	AGRG1_HUMAN	NCBI
**1720**	DVL3	DVL3_HUMAN	NCBI

No.	Symbol	Uniprot Name	Database
**1721**	CAMKK2	KKCC2_HUMAN	NCBI
**1722**	TAB1	TAB1_HUMAN	NCBI
**1723**	COL6A3	CO6A3_HUMAN	NCBI
**1724**	TPD52	TPD52_HUMAN	NCBI
**1725**	LILRB2	LIRB2_HUMAN	NCBI
**1726**	RAD51AP1	R51A1_HUMAN	NCBI
**1727**	PIAS3	PIAS3_HUMAN	NCBI
**1728**	LPAR1	LPAR1_HUMAN	NCBI
**1729**	STK3	STK3_HUMAN	NCBI
**1730**	TCL1A	TCL1A_HUMAN	NCBI
**1731**	TUBB4B	TBB4B_HUMAN	NCBI
**1732**	PRPS1	PRPS1_HUMAN	NCBI
**1733**	CRHR2	CRFR2_HUMAN	NCBI
**1734**	CCL19	CCL19_HUMAN	NCBI
**1735**	VASH1	VASH1_HUMAN	NCBI
**1736**	VSIR	VISTA_HUMAN	NCBI
**1737**	BCAT1	BCAT1_HUMAN	NCBI
**1738**	ATAD3A	ATD3A_HUMAN	NCBI
**1739**	E2F2	E2F2_HUMAN	NCBI
**1740**	CMKLR1	CML1_HUMAN	NCBI
**1741**	LRIG1	LRIG1_HUMAN	NCBI
**1742**	ALDH1A3	AL1A3_HUMAN	NCBI
**1743**	TARBP2	TRBP2_HUMAN	NCBI
**1744**	GPC1	GPC1_HUMAN	NCBI
**1745**	ACD	ACD_HUMAN	NCBI
**1746**	WNT2	WNT2_HUMAN	NCBI
**1747**	RTEL1	RTEL1_HUMAN	NCBI
**1748**	B4GALT1	B4GT1_HUMAN	NCBI
**1749**	USP11	UBP11_HUMAN	NCBI
**1750**	HOXA5	HXA5_HUMAN	NCBI
**1751**	EPHA3	EPHA3_HUMAN	NCBI
**1752**	ACTR2	ARP2_HUMAN	NCBI
**1753**	LAMTOR5	LTOR5_HUMAN	NCBI
**1754**	LGMN	LGMN_HUMAN	NCBI
**1755**	DACH1	DACH1_HUMAN	NCBI
**1756**	CCL7	CCL7_HUMAN	NCBI
**1757**	PDCD6	PDCD6_HUMAN	NCBI
**1758**	NECTIN4	NECT4_HUMAN	NCBI
**1759**	PEX5	PEX5_HUMAN	NCBI
**1760**	MAP3K11	M3K11_HUMAN	NCBI
**1761**	ATOX1	ATOX1_HUMAN	NCBI

No.	Symbol	Uniprot Name	Database
**1762**	CCT2	TCPB_HUMAN	NCBI
**1763**	TJP2	ZO2_HUMAN	NCBI
**1764**	MAP4K4	M4K4_HUMAN	NCBI
**1765**	JUND	JUND_HUMAN	NCBI
**1766**	HLA-F	HLAF_HUMAN	NCBI
**1767**	PROK1	PROK1_HUMAN	NCBI
**1768**	SMAD6	SMAD6_HUMAN	NCBI
**1769**	NUP62	NUP62_HUMAN	NCBI
**1770**	S1PR2	S1PR2_HUMAN	NCBI
**1771**	MPG	3MG_HUMAN	NCBI
**1772**	ERRFI1	ERRFI_HUMAN	NCBI
**1773**	ADGRE5	AGRE5_HUMAN	NCBI
**1774**	SFRP5	SFRP5_HUMAN	NCBI
**1775**	CDC7	CDC7_HUMAN	NCBI
**1776**	MAP2K6	MP2K6_HUMAN	NCBI
**1777**	ATAD2	ATAD2_HUMAN	NCBI
**1778**	MED13L	MD13L_HUMAN	NCBI
**1779**	SYNE1	SYNE1_HUMAN	NCBI
**1780**	CTCFL	CTCFL_HUMAN	NCBI
**1781**	POLQ	DPOLQ_HUMAN	NCBI
**1782**	HOXA13	HXA13_HUMAN	NCBI
**1783**	BUB3	BUB3_HUMAN	NCBI
**1784**	EYA4	EYA4_HUMAN	NCBI
**1785**	EED	EED_HUMAN	NCBI
**1786**	LCP1	PLSL_HUMAN	NCBI
**1787**	DSC2	DSC2_HUMAN	NCBI
**1788**	RBM3	RBM3_HUMAN	NCBI
**1789**	PYCR1	P5CR1_HUMAN	NCBI
**1790**	ENAH	ENAH_HUMAN	NCBI
**1791**	RGN	RGN_HUMAN	NCBI
**1792**	CRY2	CRY2_HUMAN	NCBI
**1793**	TCF7	TCF7_HUMAN	NCBI
**1794**	TGM3	TGM3_HUMAN	NCBI
**1795**	ARHGEF7	ARHG7_HUMAN	NCBI
**1796**	RPLP0	RLA0_HUMAN	NCBI
**1797**	PTGDR2	PD2R2_HUMAN	NCBI
**1798**	CAVIN1	CAVN1_HUMAN	NCBI
**1799**	MGAT5	MGT5A_HUMAN	NCBI
**1800**	PADI2	PADI2_HUMAN	NCBI
**1801**	NPR1	ANPRA_HUMAN	NCBI
**1802**	CCT6A	TCPZ_HUMAN	NCBI

No.	Symbol	Uniprot Name	Database
**1803**	ACAT1	THIL_HUMAN	NCBI
**1804**	CASP4	CASP4_HUMAN	NCBI
**1805**	KLF9	KLF9_HUMAN	NCBI
**1806**	TBX1	TBX1_HUMAN	NCBI
**1807**	MSX2	MSX2_HUMAN	NCBI
**1808**	DHX15	DHX15_HUMAN	NCBI
**1809**	NLRC5	NLRC5_HUMAN	NCBI
**1810**	CSMD1	CSMD1_HUMAN	NCBI
**1811**	NKX2-3	NKX23_HUMAN	NCBI
**1812**	EPS8	EPS8_HUMAN	NCBI
**1813**	KCNB1	KCNB1_HUMAN	NCBI
**1814**	FUCA1	FUCO_HUMAN	NCBI
**1815**	CUBN	CUBN_HUMAN	NCBI
**1816**	HLTF	HLTF_HUMAN	NCBI
**1817**	XPO5	XPO5_HUMAN	NCBI
**1818**	TERF2IP	TE2IP_HUMAN	NCBI
**1819**	SUMO3	SUMO3_HUMAN	NCBI
**1820**	GALNT2	GALT2_HUMAN	NCBI
**1821**	KDM5C	KDM5C_HUMAN	NCBI
**1822**	GFPT1	GFPT1_HUMAN	NCBI
**1823**	EFNA1	EFNA1_HUMAN	NCBI
**1824**	ABI1	ABI1_HUMAN	NCBI
**1825**	TRIM27	TRI27_HUMAN	NCBI
**1826**	PEG10	PEG10_HUMAN	NCBI
**1827**	HTR2B	5HT2B_HUMAN	NCBI
**1828**	IGFBP6	IBP6_HUMAN	NCBI
**1829**	NFIB	NFIB_HUMAN	NCBI
**1830**	TNS3	TENS3_HUMAN	NCBI
**1831**	WNT10A	WN10A_HUMAN	NCBI
**1832**	REG3A	REG3A_HUMAN	NCBI
**1833**	LTBP2	LTBP2_HUMAN	NCBI
**1834**	DIP2B	DIP2B_HUMAN	NCBI
**1835**	TRPV3	TRPV3_HUMAN	NCBI
**1836**	TSPAN8	TSN8_HUMAN	NCBI
**1837**	TKTL1	TKTL1_HUMAN	NCBI
**1838**	BCLAF1	BCLF1_HUMAN	NCBI
**1839**	PRDM2	PRDM2_HUMAN	NCBI
**1840**	LARP1	LARP1_HUMAN	NCBI
**1841**	BCL2L12	B2L12_HUMAN	NCBI
**1842**	CACYBP	CYBP_HUMAN	NCBI
**1843**	ANTXR1	ANTR1_HUMAN	NCBI

No.	Symbol	Uniprot Name	Database
**1844**	ANLN	ANLN_HUMAN	NCBI
**1845**	RAP1B	RAP1B_HUMAN	NCBI
**1846**	ALDOB	ALDOB_HUMAN	NCBI
**1847**	PCDH10	PCD10_HUMAN	NCBI
**1848**	NECTIN2	NECT2_HUMAN	NCBI
**1849**	SKIL	SKIL_HUMAN	NCBI
**1850**	HOXB9	HXB9_HUMAN	NCBI
**1851**	PRSS8	PRSS8_HUMAN	NCBI
**1852**	VANGL1	VANG1_HUMAN	NCBI
**1853**	MT-CO2	COX2_HUMAN	NCBI
**1854**	HSPE1	CH10_HUMAN	NCBI
**1855**	CLCN3	CLCN3_HUMAN	NCBI
**1856**	RPL3	RL3_HUMAN	NCBI
**1857**	CDH17	CAD17_HUMAN	NCBI
**1858**	VPS4A	VPS4A_HUMAN	NCBI
**1859**	SH2B1	SH2B1_HUMAN	NCBI
**1860**	GALNT3	GALT3_HUMAN	NCBI
**1861**	KLK7	KLK7_HUMAN	NCBI
**1862**	PSMD9	PSMD9_HUMAN	NCBI
**1863**	SPRY4	SPY4_HUMAN	NCBI
**1864**	GSR	GSHR_HUMAN	NCBI
**1865**	HIC1	HIC1_HUMAN	NCBI
**1866**	PLCD1	PLCD1_HUMAN	NCBI
**1867**	SLCO2A1	SO2A1_HUMAN	NCBI
**1868**	YBX3	YBOX3_HUMAN	NCBI
**1869**	ADRM1	ADRM1_HUMAN	NCBI
**1870**	DEPTOR	DPTOR_HUMAN	NCBI
**1871**	TNNT1	TNNT1_HUMAN	NCBI
**1872**	ZNF148	ZN148_HUMAN	NCBI
**1873**	ANP32A	AN32A_HUMAN	NCBI
**1874**	NTHL1	NTH_HUMAN	NCBI
**1875**	SRA1	SRA1_HUMAN	NCBI
**1876**	SELENBP1	SBP1_HUMAN	NCBI
**1877**	TRIM33	TRI33_HUMAN	NCBI
**1878**	TCF12	HTF4_HUMAN	NCBI
**1879**	GRHL2	GRHL2_HUMAN	NCBI
**1880**	HIF1AN	HIF1N_HUMAN	NCBI
**1881**	SLC14A1	UT1_HUMAN	NCBI
**1882**	RBCK1	HOIL1_HUMAN	NCBI
**1883**	SRSF6	SRSF6_HUMAN	NCBI
**1884**	PLK2	PLK2_HUMAN	NCBI

No.	Symbol	Uniprot Name	Database
**1885**	PCK1	PCKGC_HUMAN	NCBI
**1886**	HSPB2	HSPB2_HUMAN	NCBI
**1887**	SELENOS	SELS_HUMAN	NCBI
**1888**	SEMA3F	SEM3F_HUMAN	NCBI
**1889**	EPHA4	EPHA4_HUMAN	NCBI
**1890**	ATP8B1	AT8B1_HUMAN	NCBI
**1891**	TUFM	EFTU_HUMAN	NCBI
**1892**	TIGAR	TIGAR_HUMAN	NCBI
**1893**	GFI1	GFI1_HUMAN	NCBI
**1894**	PHLPP1	PHLP1_HUMAN	NCBI
**1895**	RARG	RARG_HUMAN	NCBI
**1896**	MCU	MCU_HUMAN	NCBI
**1897**	LGALS4	LEG4_HUMAN	NCBI
**1898**	SEL1L	SE1L1_HUMAN	NCBI
**1899**	ABCB5	ABCB5_HUMAN	NCBI
**1900**	SLC10A2	NTCP2_HUMAN	NCBI
**1901**	PC	PYC_HUMAN	NCBI
**1902**	FERMT3	URP2_HUMAN	NCBI
**1903**	ITM2B	ITM2B_HUMAN	NCBI
**1904**	NAF1	NAF1_HUMAN	NCBI
**1905**	FGF8	FGF8_HUMAN	NCBI
**1906**	PUF60	PUF60_HUMAN	NCBI
**1907**	NLK	NLK_HUMAN	NCBI
**1908**	NCF4	NCF4_HUMAN	NCBI
**1909**	ETV1	ETV1_HUMAN	NCBI
**1910**	EEF1D	EF1D_HUMAN	NCBI
**1911**	IRF2	IRF2_HUMAN	NCBI
**1912**	CDC5L	CDC5L_HUMAN	NCBI
**1913**	RND3	RND3_HUMAN	NCBI
**1914**	ADNP	ADNP_HUMAN	NCBI
**1915**	LILRB4	LIRB4_HUMAN	NCBI
**1916**	HVCN1	HVCN1_HUMAN	NCBI
**1917**	ELMO1	ELMO1_HUMAN	NCBI
**1918**	MAD2L2	MD2L2_HUMAN	NCBI
**1919**	CAPRIN1	CAPR1_HUMAN	NCBI
**1920**	APTX	APTX_HUMAN	NCBI
**1921**	BIRC6	BIRC6_HUMAN	NCBI
**1922**	SPOCK1	TICN1_HUMAN	NCBI
**1923**	SPINT1	SPIT1_HUMAN	NCBI
**1924**	ARHGEF2	ARHG2_HUMAN	NCBI
**1925**	EN2	HME2_HUMAN	NCBI

No.	Symbol	Uniprot Name	Database
**1926**	ABCC11	MRP8_HUMAN	NCBI
**1927**	HTATIP2	HTAI2_HUMAN	NCBI
**1928**	INPP4B	INP4B_HUMAN	NCBI
**1929**	RBFOX1	RFOX1_HUMAN	NCBI
**1930**	CLIC4	CLIC4_HUMAN	NCBI
**1931**	TAGLN2	TAGL2_HUMAN	NCBI
**1932**	RPS15A	RS15A_HUMAN	NCBI
**1933**	IREB2	IREB2_HUMAN	NCBI
**1934**	CYP4A11	CP4AB_HUMAN	NCBI
**1935**	BCAM	BCAM_HUMAN	NCBI
**1936**	AFDN	AFAD_HUMAN	NCBI
**1937**	FBXW11	FBW1B_HUMAN	NCBI
**1938**	ANXA4	ANXA4_HUMAN	NCBI
**1939**	TFF2	TFF2_HUMAN	NCBI
**1940**	MMP19	MMP19_HUMAN	NCBI
**1941**	BAG2	BAG2_HUMAN	NCBI
**1942**	PRMT6	ANM6_HUMAN	NCBI
**1943**	MAP2K3	MP2K3_HUMAN	NCBI
**1944**	KAT7	KAT7_HUMAN	NCBI
**1945**	TOX3	TOX3_HUMAN	NCBI
**1946**	SETD1A	SET1A_HUMAN	NCBI
**1947**	ESRP1	ESRP1_HUMAN	NCBI
**1948**	TRAF5	TRAF5_HUMAN	NCBI
**1949**	PINX1	PINX1_HUMAN	NCBI
**1950**	AKAP9	AKAP9_HUMAN	NCBI
**1951**	KIF20A	KI20A_HUMAN	NCBI
**1952**	GAP43	NEUM_HUMAN	NCBI
**1953**	KIF2A	KIF2A_HUMAN	NCBI
**1954**	IRS4	IRS4_HUMAN	NCBI
**1955**	HYAL1	HYAL1_HUMAN	NCBI
**1956**	ADAMTS9	ATS9_HUMAN	NCBI
**1957**	OSMR	OSMR_HUMAN	NCBI
**1958**	KPNA3	IMA4_HUMAN	NCBI
**1959**	MEF2D	MEF2D_HUMAN	NCBI
**1960**	CYP46A1	CP46A_HUMAN	NCBI
**1961**	FES	FES_HUMAN	NCBI
**1962**	SULF2	SULF2_HUMAN	NCBI
**1963**	ANXA11	ANX11_HUMAN	NCBI
**1964**	TRIP6	TRIP6_HUMAN	NCBI
**1965**	FPGS	FOLC_HUMAN	NCBI
**1966**	AQP8	AQP8_HUMAN	NCBI

No.	Symbol	Uniprot Name	Database
**1967**	TKT	TKT_HUMAN	NCBI
**1968**	CES2	EST2_HUMAN	NCBI
**1969**	HADHB	ECHB_HUMAN	NCBI
**1970**	RECQL5	RECQ5_HUMAN	NCBI
**1971**	ERP29	ERP29_HUMAN	NCBI
**1972**	MAPK12	MK12_HUMAN	NCBI
**1973**	KDM4C	KDM4C_HUMAN	NCBI
**1974**	EPHB6	EPHB6_HUMAN	NCBI
**1975**	CHKA	CHKA_HUMAN	NCBI
**1976**	ITGA7	ITA7_HUMAN	NCBI
**1977**	COPS6	CSN6_HUMAN	NCBI
**1978**	SIRT4	SIR4_HUMAN	NCBI
**1979**	UCHL5	UCHL5_HUMAN	NCBI
**1980**	RPS6KA5	KS6A5_HUMAN	NCBI
**1981**	ANK2	ANK2_HUMAN	NCBI
**1982**	PPP2R2B	2ABB_HUMAN	NCBI
**1983**	RPS27	RS27_HUMAN	NCBI
**1984**	KIF14	KIF14_HUMAN	NCBI
**1985**	UBE2M	UBC12_HUMAN	NCBI
**1986**	RPN2	RPN2_HUMAN	NCBI
**1987**	KLK5	KLK5_HUMAN	NCBI
**1988**	SYNJ2	SYNJ2_HUMAN	NCBI
**1989**	UMPS	UMPS_HUMAN	NCBI
**1990**	PDGFD	PDGFD_HUMAN	NCBI
**1991**	DPP3	DPP3_HUMAN	NCBI
**1992**	TXNDC5	TXND5_HUMAN	NCBI
**1993**	VAPA	VAPA_HUMAN	NCBI
**1994**	RNF20	BRE1A_HUMAN	NCBI
**1995**	TMPRSS3	TMPS3_HUMAN	NCBI
**1996**	TRPC5	TRPC5_HUMAN	NCBI
**1997**	MTHFD2	MTDC_HUMAN	NCBI
**1998**	NOLC1	NOLC1_HUMAN	NCBI
**1999**	TMPRSS4	TMPS4_HUMAN	NCBI
**2000**	SMYD2	SMYD2_HUMAN	NCBI
**2001**	FANCM	FANCM_HUMAN	NCBI
**2002**	TINCR	A0A1B0GTR7_HUMAN	NCBI
**2003**	KDM2A	KDM2A_HUMAN	NCBI
**2004**	CLDN6	CLD6_HUMAN	NCBI
**2005**	RPS7	RS7_HUMAN	NCBI
**2006**	CHD1L	CHD1L_HUMAN	NCBI
**2007**	TINF2	TINF2_HUMAN	NCBI

No.	Symbol	Uniprot Name	Database
**2008**	MGLL	MGLL_HUMAN	NCBI
**2009**	MT-ND2	NU2M_HUMAN	NCBI
**2010**	BTG1	BTG1_HUMAN	NCBI
**2011**	CACNA1G	CAC1G_HUMAN	NCBI
**2012**	SRGN	SRGN_HUMAN	NCBI
**2013**	NOB1	NOB1_HUMAN	NCBI
**2014**	TCF21	TCF21_HUMAN	NCBI
**2015**	USP4	UBP4_HUMAN	NCBI
**2016**	ERBIN	ERBIN_HUMAN	NCBI
**2017**	CDC45	CDC45_HUMAN	NCBI
**2018**	ARID2	ARID2_HUMAN	NCBI
**2019**	CNBP	CNBP_HUMAN	NCBI
**2020**	ZMPSTE24	FACE1_HUMAN	NCBI
**2021**	E2F7	E2F7_HUMAN	NCBI
**2022**	ADAM8	ADAM8_HUMAN	NCBI
**2023**	TFCP2	TFCP2_HUMAN	NCBI
**2024**	ABHD5	ABHD5_HUMAN	NCBI
**2025**	ACACB	ACACB_HUMAN	NCBI
**2026**	PNN	PININ_HUMAN	NCBI
**2027**	FOXK1	FOXK1_HUMAN	NCBI
**2028**	FAF1	FAF1_HUMAN	NCBI
**2029**	PTPRF	PTPRF_HUMAN	NCBI
**2030**	DSC3	DSC3_HUMAN	NCBI
**2031**	EPHA1	EPHA1_HUMAN	NCBI
**2032**	USP39	UBP39_HUMAN	NCBI
**2033**	SRI	SORCN_HUMAN	NCBI
**2034**	IL36A	IL36A_HUMAN	NCBI
**2035**	YTHDC1	YTDC1_HUMAN	NCBI
**2036**	ETV5	ETV5_HUMAN	NCBI
**2037**	PSAT1	SERC_HUMAN	NCBI
**2038**	COPB2	COPB2_HUMAN	NCBI
**2039**	MAP2K5	MP2K5_HUMAN	NCBI
**2040**	ABCC5	MRP5_HUMAN	NCBI
**2041**	SQLE	ERG1_HUMAN	NCBI
**2042**	CYSLTR2	CLTR2_HUMAN	NCBI
**2043**	RPS20	RS20_HUMAN	NCBI
**2044**	IRAK3	IRAK3_HUMAN	NCBI
**2045**	KLRD1	KLRD1_HUMAN	NCBI
**2046**	CPE	CBPE_HUMAN	NCBI
**2047**	ZFX	ZFX_HUMAN	NCBI
**2048**	ACO2	ACON_HUMAN	NCBI

No.	Symbol	Uniprot Name	Database
**2049**	SLC34A2	NPT2B_HUMAN	NCBI
**2050**	REV1	REV1_HUMAN	NCBI
**2051**	ZNF217	ZN217_HUMAN	NCBI
**2052**	THBS4	TSP4_HUMAN	NCBI
**2053**	KRT6A	K2C6A_HUMAN	NCBI
**2054**	GFRA1	GFRA1_HUMAN	NCBI
**2055**	TES	TES_HUMAN	NCBI
**2056**	PLAC1	PLAC1_HUMAN	NCBI
**2057**	FLII	FLII_HUMAN	NCBI
**2058**	DBP	DBP_HUMAN	NCBI
**2059**	FRZB	SFRP3_HUMAN	NCBI
**2060**	PRSS2	TRY2_HUMAN	NCBI
**2061**	VEGFB	VEGFB_HUMAN	NCBI
**2062**	RAD17	RAD17_HUMAN	NCBI
**2063**	CORO1C	COR1C_HUMAN	NCBI
**2064**	CFD	CFAD_HUMAN	NCBI
**2065**	CLDN18	CLD18_HUMAN	NCBI
**2066**	PIWIL2	PIWL2_HUMAN	NCBI
**2067**	SPARCL1	SPRL1_HUMAN	NCBI
**2068**	ICOSLG	ICOSL_HUMAN	NCBI
**2069**	MAGEA1	MAGA1_HUMAN	NCBI
**2070**	SPAG5	SPAG5_HUMAN	NCBI
**2071**	GGH	GGH_HUMAN	NCBI
**2072**	DTL	DTL_HUMAN	NCBI
**2073**	MNX1	MNX1_HUMAN	NCBI
**2074**	SRSF9	SRSF9_HUMAN	NCBI
**2075**	ADM2	ADM2_HUMAN	NCBI
**2076**	FAM83H	FA83H_HUMAN	NCBI
**2077**	P4HA1	P4HA1_HUMAN	NCBI
**2078**	SF3B3	SF3B3_HUMAN	NCBI
**2079**	UBA2	SAE2_HUMAN	NCBI
**2080**	SLC26A2	S26A2_HUMAN	NCBI
**2081**	KNL1	KNL1_HUMAN	NCBI
**2082**	RPS24	RS24_HUMAN	NCBI
**2083**	RBM4	RBM4_HUMAN	NCBI
**2084**	EIF5A2	IF5A2_HUMAN	NCBI
**2085**	UVRAG	UVRAG_HUMAN	NCBI
**2086**	TWIST2	TWST2_HUMAN	NCBI
**2087**	PLXNB1	PLXB1_HUMAN	NCBI
**2088**	PIK3R3	P55G_HUMAN	NCBI
**2089**	MTA3	MTA3_HUMAN	NCBI

No.	Symbol	Uniprot Name	Database
**2090**	SOX7	SOX7_HUMAN	NCBI
**2091**	ECHS1	ECHM_HUMAN	NCBI
**2092**	AURKC	AURKC_HUMAN	NCBI
**2093**	PRRX1	PRRX1_HUMAN	NCBI
**2094**	MCRS1	MCRS1_HUMAN	NCBI
**2095**	FSTL3	FSTL3_HUMAN	NCBI
**2096**	GIPC1	GIPC1_HUMAN	NCBI
**2097**	GZMA	GRAA_HUMAN	NCBI
**2098**	GSTO2	GSTO2_HUMAN	NCBI
**2099**	STRAP	STRAP_HUMAN	NCBI
**2100**	ARHGAP35	RHG35_HUMAN	NCBI
**2101**	DLG2	DLG2_HUMAN	NCBI
**2102**	MUC6	MUC6_HUMAN	NCBI
**2103**	PABPC4	PABP4_HUMAN	NCBI
**2104**	HHLA2	HHLA2_HUMAN	NCBI
**2105**	SPTBN2	SPTN2_HUMAN	NCBI
**2106**	EIF3I	EIF3I_HUMAN	NCBI
**2107**	ATG14	BAKOR_HUMAN	NCBI
**2108**	HMGB3	HMGB3_HUMAN	NCBI
**2109**	MKNK1	MKNK1_HUMAN	NCBI
**2110**	WRAP53	TCAB1_HUMAN	NCBI
**2111**	SERPINI1	NEUS_HUMAN	NCBI
**2112**	FOSL2	FOSL2_HUMAN	NCBI
**2113**	USP28	UBP28_HUMAN	NCBI
**2114**	KLF8	KLF8_HUMAN	NCBI
**2115**	KLF15	KLF15_HUMAN	NCBI
**2116**	RETREG1	RETR1_HUMAN	NCBI
**2117**	SLC39A14	S39AE_HUMAN	NCBI
**2118**	SGPL1	SGPL1_HUMAN	NCBI
**2119**	CNTN1	CNTN1_HUMAN	NCBI
**2120**	PRKACB	KAPCB_HUMAN	NCBI
**2121**	AMBRA1	AMRA1_HUMAN	NCBI
**2122**	MAL	MAL_HUMAN	NCBI
**2123**	MAPKAP1	SIN1_HUMAN	NCBI
**2124**	SPART	SPART_HUMAN	NCBI
**2125**	PXDN	PXDN_HUMAN	NCBI
**2126**	HNRNPAB	ROAA_HUMAN	NCBI
**2127**	RIOX2	RIOX2_HUMAN	NCBI
**2128**	LMNB2	LMNB2_HUMAN	NCBI
**2129**	RRBP1	RRBP1_HUMAN	NCBI
**2130**	EIF3F	EIF3F_HUMAN	NCBI

No.	Symbol	Uniprot Name	Database
**2131**	LTB4R2	LT4R2_HUMAN	NCBI
**2132**	TIMP4	TIMP4_HUMAN	NCBI
**2133**	SLC5A8	SC5A8_HUMAN	NCBI
**2134**	SEC23B	SC23B_HUMAN	NCBI
**2135**	MEIS2	MEIS2_HUMAN	NCBI
**2136**	MN1	MN1_HUMAN	NCBI
**2137**	FAM3B	FAM3B_HUMAN	NCBI
**2138**	PPA1	IPYR_HUMAN	NCBI
**2139**	PAK5	PAK5_HUMAN	NCBI
**2140**	CAPNS1	CPNS1_HUMAN	NCBI
**2141**	KIF18A	KI18A_HUMAN	NCBI
**2142**	VAMP8	VAMP8_HUMAN	NCBI
**2143**	HOPX	HOP_HUMAN	NCBI
**2144**	TRIM59	TRI59_HUMAN	NCBI
**2145**	GSPT1	ERF3A_HUMAN	NCBI
**2146**	PHLPP2	PHLP2_HUMAN	NCBI
**2147**	RAE1	RAE1L_HUMAN	NCBI
**2148**	TRAF3IP2	CIKS_HUMAN	NCBI
**2149**	IFIT3	IFIT3_HUMAN	NCBI
**2150**	BPTF	BPTF_HUMAN	NCBI
**2151**	INSIG1	INSI1_HUMAN	NCBI
**2152**	TNS4	TENS4_HUMAN	NCBI
**2153**	VMP1	VMP1_HUMAN	NCBI
**2154**	FKBP10	FKB10_HUMAN	NCBI
**2155**	SLFN11	SLN11_HUMAN	NCBI
**2156**	CDCA8	BOREA_HUMAN	NCBI
**2157**	TENM3	TEN3_HUMAN	NCBI
**2158**	C1GALT1	C1GLT_HUMAN	NCBI
**2159**	PPP2R5C	2A5G_HUMAN	NCBI
**2160**	ATOH1	ATOH1_HUMAN	NCBI
**2161**	DAP3	RT29_HUMAN	NCBI
**2162**	AMER1	AMER1_HUMAN	NCBI
**2163**	UNC5C	UNC5C_HUMAN	NCBI
**2164**	VSNL1	VISL1_HUMAN	NCBI
**2165**	RAB27B	RB27B_HUMAN	NCBI
**2166**	PIP5K1C	PI51C_HUMAN	NCBI
**2167**	RBFOX2	RFOX2_HUMAN	NCBI
**2168**	NUCKS1	NUCKS_HUMAN	NCBI
**2169**	PROK2	PROK2_HUMAN	NCBI
**2170**	GRIN2D	NMDE4_HUMAN	NCBI
**2171**	ZIC2	ZIC2_HUMAN	NCBI

No.	Symbol	Uniprot Name	Database
**2172**	SCG2	SCG2_HUMAN	NCBI
**2173**	HACE1	HACE1_HUMAN	NCBI
**2174**	NUSAP1	NUSAP_HUMAN	NCBI
**2175**	ATG4B	ATG4B_HUMAN	NCBI
**2176**	RAB1B	RAB1B_HUMAN	NCBI
**2177**	CDKN3	CDKN3_HUMAN	NCBI
**2178**	MLLT3	AF9_HUMAN	NCBI
**2179**	DSC1	DSC1_HUMAN	NCBI
**2180**	POFUT1	OFUT1_HUMAN	NCBI
**2181**	FOXD3	FOXD3_HUMAN	NCBI
**2182**	RBBP6	RBBP6_HUMAN	NCBI
**2183**	LETM1	LETM1_HUMAN	NCBI
**2184**	IGHA1	IGHA1_HUMAN	NCBI
**2185**	CNOT7	CNOT7_HUMAN	NCBI
**2186**	TFDP1	TFDP1_HUMAN	NCBI
**2187**	RAB14	RAB14_HUMAN	NCBI
**2188**	WDR62	WDR62_HUMAN	NCBI
**2189**	TFAP4	TFAP4_HUMAN	NCBI
**2190**	WASF3	WASF3_HUMAN	NCBI
**2191**	CDO1	CDO1_HUMAN	NCBI
**2192**	AATF	AATF_HUMAN	NCBI
**2193**	PLAGL2	PLAL2_HUMAN	NCBI
**2194**	PMEPA1	PMEPA_HUMAN	NCBI
**2195**	KIF15	KIF15_HUMAN	NCBI
**2196**	NID1	NID1_HUMAN	NCBI
**2197**	CA4	CAH4_HUMAN	NCBI
**2198**	PRM1	HSP1_HUMAN	NCBI
**2199**	ACADS	ACADS_HUMAN	NCBI
**2200**	ACYP2	ACYP2_HUMAN	NCBI
**2201**	CAPG	CAPG_HUMAN	NCBI
**2202**	PTPRN	PTPRN_HUMAN	NCBI
**2203**	GATA5	GATA5_HUMAN	NCBI
**2204**	SHOC2	SHOC2_HUMAN	NCBI
**2205**	CCL15	CCL15_HUMAN	NCBI
**2206**	LPAR2	LPAR2_HUMAN	NCBI
**2207**	RSPO1	RSPO1_HUMAN	NCBI
**2208**	GPX2	GPX2_HUMAN	NCBI
**2209**	DUT	DUT_HUMAN	NCBI
**2210**	RFC5	RFC5_HUMAN	NCBI
**2211**	RSL1D1	RL1D1_HUMAN	NCBI
**2212**	HMGCS2	HMCS2_HUMAN	NCBI

No.	Symbol	Uniprot Name	Database
**2213**	PBX3	PBX3_HUMAN	NCBI
**2214**	TNFAIP8	TFIP8_HUMAN	NCBI
**2215**	GART	PUR2_HUMAN	NCBI
**2216**	PCK2	PCKGM_HUMAN	NCBI
**2217**	HSPA12A	HS12A_HUMAN	NCBI
**2218**	USP25	UBP25_HUMAN	NCBI
**2219**	ALDH1B1	AL1B1_HUMAN	NCBI
**2220**	PRSS3	TRY3_HUMAN	NCBI
**2221**	SLAMF7	SLAF7_HUMAN	NCBI
**2222**	ANAPC11	APC11_HUMAN	NCBI
**2223**	SLC2A5	GTR5_HUMAN	NCBI
**2224**	TM4SF1	T4S1_HUMAN	NCBI
**2225**	NPAS2	NPAS2_HUMAN	NCBI
**2226**	HYAL2	HYAL2_HUMAN	NCBI
**2227**	RALY	RALY_HUMAN	NCBI
**2228**	DUSP22	DUS22_HUMAN	NCBI
**2229**	ACSL5	ACSL5_HUMAN	NCBI
**2230**	MORC2	MORC2_HUMAN	NCBI
**2231**	SIGLEC9	SIGL9_HUMAN	NCBI
**2232**	MAGEA4	MAGA4_HUMAN	NCBI
**2233**	TDO2	T23O_HUMAN	NCBI
**2234**	MMP26	MMP26_HUMAN	NCBI
**2235**	PFN2	PROF2_HUMAN	NCBI
**2236**	BCL9	BCL9_HUMAN	NCBI
**2237**	JARID2	JARD2_HUMAN	NCBI
**2238**	BRF1	TF3B_HUMAN	NCBI
**2239**	CCL1	CCL1_HUMAN	NCBI
**2240**	CTPS1	PYRG1_HUMAN	NCBI
**2241**	PTPN14	PTN14_HUMAN	NCBI
**2242**	VPS4B	VPS4B_HUMAN	NCBI
**2243**	BICC1	BICC1_HUMAN	NCBI
**2244**	SNX9	SNX9_HUMAN	NCBI
**2245**	MYO1B	MYO1B_HUMAN	NCBI
**2246**	DYRK1B	DYR1B_HUMAN	NCBI
**2247**	HJURP	HJURP_HUMAN	NCBI
**2248**	ARHGEF12	ARHGC_HUMAN	NCBI
**2249**	PPP2R1B	2AAB_HUMAN	NCBI
**2250**	PRKAG1	AAKG1_HUMAN	NCBI
**2251**	DLGAP5	DLGP5_HUMAN	NCBI
**2252**	RRAS	RRAS_HUMAN	NCBI
**2253**	EOMES	EOMES_HUMAN	NCBI

No.	Symbol	Uniprot Name	Database
**2254**	HPSE2	HPSE2_HUMAN	NCBI
**2255**	CYTH2	CYH2_HUMAN	NCBI
**2256**	HOXD10	HXD10_HUMAN	NCBI
**2257**	PES1	PESC_HUMAN	NCBI
**2258**	KIF3A	KIF3A_HUMAN	NCBI
**2259**	MMP16	MMP16_HUMAN	NCBI
**2260**	CTSH	CATH_HUMAN	NCBI
**2261**	ACIN1	ACINU_HUMAN	NCBI
**2262**	LIMK2	LIMK2_HUMAN	NCBI
**2263**	SLC7A1	CTR1_HUMAN	NCBI
**2264**	CXCL3	CXCL3_HUMAN	NCBI
**2265**	NLRP12	NAL12_HUMAN	NCBI
**2266**	DYRK2	DYRK2_HUMAN	NCBI
**2267**	CA8	CAH8_HUMAN	NCBI
**2268**	FZD5	FZD5_HUMAN	NCBI
**2269**	NDUFS1	NDUS1_HUMAN	NCBI
**2270**	HNRNPDL	HNRDL_HUMAN	NCBI
**2271**	METTL16	MET16_HUMAN	NCBI
**2272**	BCCIP	BCCIP_HUMAN	NCBI
**2273**	HSD17B12	DHB12_HUMAN	NCBI
**2274**	SF3A1	SF3A1_HUMAN	NCBI
**2275**	NUP88	NUP88_HUMAN	NCBI
**2276**	SORBS1	SRBS1_HUMAN	NCBI
**2277**	CASP8AP2	C8AP2_HUMAN	NCBI
**2278**	NFIC	NFIC_HUMAN	NCBI
**2279**	ACY1	ACY1_HUMAN	NCBI
**2280**	TRIM44	TRI44_HUMAN	NCBI
**2281**	SGO1	SGO1_HUMAN	NCBI
**2282**	WNT3	WNT3_HUMAN	NCBI
**2283**	FGF6	FGF6_HUMAN	NCBI
**2284**	NFATC3	NFAC3_HUMAN	NCBI
**2285**	NLRX1	NLRX1_HUMAN	NCBI
**2286**	FGF13	FGF13_HUMAN	NCBI
**2287**	TNKS2	TNKS2_HUMAN	NCBI
**2288**	CDX1	CDX1_HUMAN	NCBI
**2289**	SIM2	SIM2_HUMAN	NCBI
**2290**	TUBB6	TBB6_HUMAN	NCBI
**2291**	BAMBI	BAMBI_HUMAN	NCBI
**2292**	SYNE2	SYNE2_HUMAN	NCBI
**2293**	TUSC3	TUSC3_HUMAN	NCBI
**2294**	HEY1	HEY1_HUMAN	NCBI

No.	Symbol	Uniprot Name	Database
**2295**	CEACAM3	CEAM3_HUMAN	NCBI
**2296**	GLDC	GCSP_HUMAN	NCBI
**2297**	PTPRK	PTPRK_HUMAN	NCBI
**2298**	EPHA7	EPHA7_HUMAN	NCBI
**2299**	RPL27	RL27_HUMAN	NCBI
**2300**	CELF2	CELF2_HUMAN	NCBI
**2301**	PLOD3	PLOD3_HUMAN	NCBI
**2302**	WNT2B	WNT2B_HUMAN	NCBI
**2303**	RBM15	RBM15_HUMAN	NCBI
**2304**	RPL23A	RL23A_HUMAN	NCBI
**2305**	DHODH	PYRD_HUMAN	NCBI
**2306**	RASSF2	RASF2_HUMAN	NCBI
**2307**	EPHB1	EPHB1_HUMAN	NCBI
**2308**	MELTF	TRFM_HUMAN	NCBI
**2309**	CPEB4	CPEB4_HUMAN	NCBI
**2310**	S100A14	S10AE_HUMAN	NCBI
**2311**	IBSP	SIAL_HUMAN	NCBI
**2312**	ST13	F10A1_HUMAN	NCBI
**2313**	ARID3A	ARI3A_HUMAN	NCBI
**2314**	MBD1	MBD1_HUMAN	NCBI
**2315**	MTUS1	MTUS1_HUMAN	NCBI
**2316**	CBX7	CBX7_HUMAN	NCBI
**2317**	RPL28	RL28_HUMAN	NCBI
**2318**	WIPF1	WIPF1_HUMAN	NCBI
**2319**	RSPO3	RSPO3_HUMAN	NCBI
**2320**	EMP1	EMP1_HUMAN	NCBI
**2321**	FOXK2	FOXK2_HUMAN	NCBI
**2322**	ETFA	ETFA_HUMAN	NCBI
**2323**	ASCL2	ASCL2_HUMAN	NCBI
**2324**	DPP9	DPP9_HUMAN	NCBI
**2325**	CTNNA3	CTNA3_HUMAN	NCBI
**2326**	AJUBA	AJUBA_HUMAN	NCBI
**2327**	SCTR	SCTR_HUMAN	NCBI
**2328**	POLR2F	RPAB2_HUMAN	NCBI
**2329**	RCC2	RCC2_HUMAN	NCBI
**2330**	HTRA3	HTRA3_HUMAN	NCBI
**2331**	GEMIN4	GEMI4_HUMAN	NCBI
**2332**	FMNL2	FMNL2_HUMAN	NCBI
**2333**	SIGLEC15	SIG15_HUMAN	NCBI
**2334**	OGN	MIME_HUMAN	NCBI
**2335**	RPL31	RL31_HUMAN	NCBI

No.	Symbol	Uniprot Name	Database
**2336**	CRTC2	CRTC2_HUMAN	NCBI
**2337**	ME1	MAOX_HUMAN	NCBI
**2338**	GDI1	GDIA_HUMAN	NCBI
**2339**	SEC23A	SC23A_HUMAN	NCBI
**2340**	FAN1	FAN1_HUMAN	NCBI
**2341**	GSS	GSHB_HUMAN	NCBI
**2342**	KLK8	KLK8_HUMAN	NCBI
**2343**	PHLDA2	PHLA2_HUMAN	NCBI
**2344**	SNX1	SNX1_HUMAN	NCBI
**2345**	UQCRC2	QCR2_HUMAN	NCBI
**2346**	MAPK13	MK13_HUMAN	NCBI
**2347**	SLC5A7	SC5A7_HUMAN	NCBI
**2348**	COL12A1	COCA1_HUMAN	NCBI
**2349**	RSPO2	RSPO2_HUMAN	NCBI
**2350**	ABCF1	ABCF1_HUMAN	NCBI
**2351**	EIF2A	EIF2A_HUMAN	NCBI
**2352**	TRAPPC9	TPPC9_HUMAN	NCBI
**2353**	IL13RA1	I13R1_HUMAN	NCBI
**2354**	ADH7	ADH7_HUMAN	NCBI
**2355**	RPS6KA6	KS6A6_HUMAN	NCBI
**2356**	NEIL2	NEIL2_HUMAN	NCBI
**2357**	RNF41	RNF41_HUMAN	NCBI
**2358**	CDK5RAP3	CK5P3_HUMAN	NCBI
**2359**	ADGRG6	AGRG6_HUMAN	NCBI
**2360**	MED19	MED19_HUMAN	NCBI
**2361**	RYR3	RYR3_HUMAN	NCBI
**2362**	BTF3	BTF3_HUMAN	NCBI
**2363**	DIAPH3	DIAP3_HUMAN	NCBI
**2364**	RASGRP1	GRP1_HUMAN	NCBI
**2365**	DIS3	RRP44_HUMAN	NCBI
**2366**	AEBP1	AEBP1_HUMAN	NCBI
**2367**	E2F5	E2F5_HUMAN	NCBI
**2368**	EFEMP2	FBLN4_HUMAN	NCBI
**2369**	HBP1	HBP1_HUMAN	NCBI
**2370**	NOVA1	NOVA1_HUMAN	NCBI
**2371**	NFYB	NFYB_HUMAN	NCBI
**2372**	NUAK1	NUAK1_HUMAN	NCBI
**2373**	SNRPA1	RU2A_HUMAN	NCBI
**2374**	CST1	CYTN_HUMAN	NCBI
**2375**	BATF2	BATF2_HUMAN	NCBI
**2376**	CTSV	CATL2_HUMAN	NCBI

No.	Symbol	Uniprot Name	Database
**2377**	SUGT1	SGT1_HUMAN	NCBI
**2378**	RALB	RALB_HUMAN	NCBI
**2379**	PLA2G10	PA2GX_HUMAN	NCBI
**2380**	CBX8	CBX8_HUMAN	NCBI
**2381**	PRMT3	ANM3_HUMAN	NCBI
**2382**	PELI1	PELI1_HUMAN	NCBI
**2383**	NFKBIZ	IKBZ_HUMAN	NCBI
**2384**	NCR2	NCTR2_HUMAN	NCBI
**2385**	RFC2	RFC2_HUMAN	NCBI
**2386**	LRAT	LRAT_HUMAN	NCBI
**2387**	KRT15	K1C15_HUMAN	NCBI
**2388**	TACR2	NK2R_HUMAN	NCBI
**2389**	MYO10	MYO10_HUMAN	NCBI
**2390**	KSR1	KSR1_HUMAN	NCBI
**2391**	RAB5C	RAB5C_HUMAN	NCBI
**2392**	OLA1	OLA1_HUMAN	NCBI
**2393**	LOXL4	LOXL4_HUMAN	NCBI
**2394**	MIB1	MIB1_HUMAN	NCBI
**2395**	HSPB6	HSPB6_HUMAN	NCBI
**2396**	C1GALT1C1	C1GLC_HUMAN	NCBI
**2397**	LARS2	SYLM_HUMAN	NCBI
**2398**	USP21	UBP21_HUMAN	NCBI
**2399**	UGT1A8	UD18_HUMAN	NCBI
**2400**	TCF7L1	TF7L1_HUMAN	NCBI
**2401**	FGF20	FGF20_HUMAN	NCBI
**2402**	KLK11	KLK11_HUMAN	NCBI
**2403**	KLF7	KLF7_HUMAN	NCBI
**2404**	RARRES1	TIG1_HUMAN	NCBI
**2405**	RAB22A	RB22A_HUMAN	NCBI
**2406**	NTF4	NTF4_HUMAN	NCBI
**2407**	HIPK3	HIPK3_HUMAN	NCBI
**2408**	SPIN1	SPIN1_HUMAN	NCBI
**2409**	FGF12	FGF12_HUMAN	NCBI
**2410**	STAB2	STAB2_HUMAN	NCBI
**2411**	RAP2A	RAP2A_HUMAN	NCBI
**2412**	CDK2AP1	CDKA1_HUMAN	NCBI
**2413**	SRSF10	SRS10_HUMAN	NCBI
**2414**	TOX	TOX_HUMAN	NCBI
**2415**	SEMA3B	SEM3B_HUMAN	NCBI
**2416**	CCNG2	CCNG2_HUMAN	NCBI
**2417**	TCN1	TCO1_HUMAN	NCBI

No.	Symbol	Uniprot Name	Database
**2418**	ITGB8	ITB8_HUMAN	NCBI
**2419**	BRAP	BRAP_HUMAN	NCBI
**2420**	PYGO2	PYGO2_HUMAN	NCBI
**2421**	HOXC6	HXC6_HUMAN	NCBI
**2422**	NDRG4	NDRG4_HUMAN	NCBI
**2423**	COPS8	CSN8_HUMAN	NCBI
**2424**	SRPK2	SRPK2_HUMAN	NCBI
**2425**	RTRAF	RTRAF_HUMAN	NCBI
**2426**	CIAPIN1	CPIN1_HUMAN	NCBI
**2427**	GAS1	GAS1_HUMAN	NCBI
**2428**	MYL9	MYL9_HUMAN	NCBI
**2429**	BLNK	BLNK_HUMAN	NCBI
**2430**	LPAR3	LPAR3_HUMAN	NCBI
**2431**	FOXF2	FOXF2_HUMAN	NCBI
**2432**	LRRC32	LRC32_HUMAN	NCBI
**2433**	TIE1	TIE1_HUMAN	NCBI
**2434**	NMI	NMI_HUMAN	NCBI
**2435**	SEC61B	SC61B_HUMAN	NCBI
**2436**	SHOX2	SHOX2_HUMAN	NCBI
**2437**	FGL1	FGL1_HUMAN	NCBI
**2438**	TNIK	TNIK_HUMAN	NCBI
**2439**	CDK16	CDK16_HUMAN	NCBI
**2440**	BRD3	BRD3_HUMAN	NCBI
**2441**	PNKD	PNKD_HUMAN	NCBI
**2442**	ADAM28	ADA28_HUMAN	NCBI
**2443**	SIK2	SIK2_HUMAN	NCBI
**2444**	FOXD1	FOXD1_HUMAN	NCBI
**2445**	YTHDF3	YTHD3_HUMAN	NCBI
**2446**	CASP5	CASP5_HUMAN	NCBI
**2447**	USP36	UBP36_HUMAN	NCBI
**2448**	CPM	CBPM_HUMAN	NCBI
**2449**	CLCA1	CLCA1_HUMAN	NCBI
**2450**	TAB3	TAB3_HUMAN	NCBI
**2451**	LLGL1	L2GL1_HUMAN	NCBI
**2452**	NUP210	PO210_HUMAN	NCBI
**2453**	MYCBP2	MYCB2_HUMAN	NCBI
**2454**	DOCK7	DOCK7_HUMAN	NCBI
**2455**	SMARCA1	SMCA1_HUMAN	NCBI
**2456**	HASPIN	HASP_HUMAN	NCBI
**2457**	TLX1	TLX1_HUMAN	NCBI
**2458**	MYCL	MYCL_HUMAN	NCBI

No.	Symbol	Uniprot Name	Database
**2459**	DEPDC1	DEP1A_HUMAN	NCBI
**2460**	COL8A1	CO8A1_HUMAN	NCBI
**2461**	HOXC10	HXC10_HUMAN	NCBI
**2462**	IL17RB	I17RB_HUMAN	NCBI
**2463**	ZMYND8	ZMYD8_HUMAN	NCBI
**2464**	DUSP5	DUS5_HUMAN	NCBI
**2465**	GPSM2	GPSM2_HUMAN	NCBI
**2466**	DPY30	DPY30_HUMAN	NCBI
**2467**	NMU	NMU_HUMAN	NCBI
**2468**	ADD2	ADDB_HUMAN	NCBI
**2469**	RAB21	RAB21_HUMAN	NCBI
**2470**	NEK6	NEK6_HUMAN	NCBI
**2471**	CTSZ	CATZ_HUMAN	NCBI
**2472**	HERC5	HERC5_HUMAN	NCBI
**2473**	GLRX3	GLRX3_HUMAN	NCBI
**2474**	RPS6KA2	KS6A2_HUMAN	NCBI
**2475**	RPRD1B	RPR1B_HUMAN	NCBI
**2476**	DNAAF4	DAAF4_HUMAN	NCBI
**2477**	WNT7B	WNT7B_HUMAN	NCBI
**2478**	STK24	STK24_HUMAN	NCBI
**2479**	GRIA4	GRIA4_HUMAN	NCBI
**2480**	MICAL1	MICA1_HUMAN	NCBI
**2481**	PARVA	PARVA_HUMAN	NCBI
**2482**	TBX20	TBX20_HUMAN	NCBI
**2483**	AFF2	AFF2_HUMAN	NCBI
**2484**	RNF40	BRE1B_HUMAN	NCBI
**2485**	DKK2	DKK2_HUMAN	NCBI
**2486**	CMTM6	CKLF6_HUMAN	NCBI
**2487**	TRIP10	CIP4_HUMAN	NCBI
**2488**	ADCY9	ADCY9_HUMAN	NCBI
**2489**	TMEM97	SGMR2_HUMAN	NCBI
**2490**	HOXB5	HXB5_HUMAN	NCBI
**2491**	SKA3	SKA3_HUMAN	NCBI
**2492**	MFAP5	MFAP5_HUMAN	NCBI
**2493**	PFKFB2	F262_HUMAN	NCBI
**2494**	GPR35	GPR35_HUMAN	NCBI
**2495**	RAP2B	RAP2B_HUMAN	NCBI
**2496**	P4HA2	P4HA2_HUMAN	NCBI
**2497**	VIRMA	VIR_HUMAN	NCBI
**2498**	NLGN1	NLGN1_HUMAN	NCBI
**2499**	AK2	KAD2_HUMAN	NCBI

No.	Symbol	Uniprot Name	Database
**2500**	CDCA2	CDCA2_HUMAN	NCBI
**2501**	HSF4	HSF4_HUMAN	NCBI
**2502**	RASAL2	NGAP_HUMAN	NCBI
**2503**	NRCAM	NRCAM_HUMAN	NCBI
**2504**	MCCC2	MCCB_HUMAN	NCBI
**2505**	ATP6V0C	VATL_HUMAN	NCBI
**2506**	GTPBP4	GTPB4_HUMAN	NCBI
**2507**	RASGRF1	RGRF1_HUMAN	NCBI
**2508**	IDO2	I23O2_HUMAN	NCBI
**2509**	PDGFRL	PGFRL_HUMAN	NCBI
**2510**	MPDZ	MPDZ_HUMAN	NCBI
**2511**	FANCF	FANCF_HUMAN	NCBI
**2512**	MARVELD2	MALD2_HUMAN	NCBI
**2513**	ALAS1	HEM1_HUMAN	NCBI
**2514**	IST1	IST1_HUMAN	NCBI
**2515**	SEMA4A	SEM4A_HUMAN	NCBI
**2516**	LYAR	LYAR_HUMAN	NCBI
**2517**	GRHL3	GRHL3_HUMAN	NCBI
**2518**	AGFG1	AGFG1_HUMAN	NCBI
**2519**	FNDC3B	FND3B_HUMAN	NCBI
**2520**	PHLDA1	PHLA1_HUMAN	NCBI
**2521**	MGST1	MGST1_HUMAN	NCBI
**2522**	DCUN1D1	DCNL1_HUMAN	NCBI
**2523**	CPEB1	CPEB1_HUMAN	NCBI
**2524**	VNN1	VNN1_HUMAN	NCBI
**2525**	COL4A6	CO4A6_HUMAN	NCBI
**2526**	KLF12	KLF12_HUMAN	NCBI
**2527**	LDB1	LDB1_HUMAN	NCBI
**2528**	STRA6	STRA6_HUMAN	NCBI
**2529**	BANP	BANP_HUMAN	NCBI
**2530**	RBM38	RBM38_HUMAN	NCBI
**2531**	SSH1	SSH1_HUMAN	NCBI
**2532**	CTNNBL1	CTBL1_HUMAN	NCBI
**2533**	CREB5	CREB5_HUMAN	NCBI
**2534**	ALDOC	ALDOC_HUMAN	NCBI
**2535**	USP47	UBP47_HUMAN	NCBI
**2536**	SEMA5A	SEM5A_HUMAN	NCBI
**2537**	FBLN2	FBLN2_HUMAN	NCBI
**2538**	FXYD5	FXYD5_HUMAN	NCBI
**2539**	PDHX	ODPX_HUMAN	NCBI
**2540**	CNOT3	CNOT3_HUMAN	NCBI

No.	Symbol	Uniprot Name	Database
**2541**	HACD3	HACD3_HUMAN	NCBI
**2542**	RGS6	RGS6_HUMAN	NCBI
**2543**	CCL25	CCL25_HUMAN	NCBI
**2544**	PCGF2	PCGF2_HUMAN	NCBI
**2545**	PLXNB2	PLXB2_HUMAN	NCBI
**2546**	LAMTOR1	LTOR1_HUMAN	NCBI
**2547**	GREB1	GREB1_HUMAN	NCBI
**2548**	GRB14	GRB14_HUMAN	NCBI
**2549**	ZNF384	ZN384_HUMAN	NCBI
**2550**	RBM17	SPF45_HUMAN	NCBI
**2551**	HMGN5	HMGN5_HUMAN	NCBI
**2552**	MZF1	MZF1_HUMAN	NCBI
**2553**	FZD3	FZD3_HUMAN	NCBI
**2554**	NDN	NECD_HUMAN	NCBI
**2555**	OTX1	OTX1_HUMAN	NCBI
**2556**	BOP1	BOP1_HUMAN	NCBI
**2557**	GPSM1	GPSM1_HUMAN	NCBI
**2558**	UHRF2	UHRF2_HUMAN	NCBI
**2559**	SIPA1	SIPA1_HUMAN	NCBI
**2560**	RGS16	RGS16_HUMAN	NCBI
**2561**	GPR15	GPR15_HUMAN	NCBI
**2562**	KIN	KIN17_HUMAN	NCBI
**2563**	GNB5	GNB5_HUMAN	NCBI
**2564**	ZBTB7B	ZBT7B_HUMAN	NCBI
**2565**	SERPINA10	ZPI_HUMAN	NCBI
**2566**	LACTB	LACTB_HUMAN	NCBI
**2567**	AOX1	AOXA_HUMAN	NCBI
**2568**	PGM3	AGM1_HUMAN	NCBI
**2569**	S100A16	S10AG_HUMAN	NCBI
**2570**	SAV1	SAV1_HUMAN	NCBI
**2571**	RIN1	RIN1_HUMAN	NCBI
**2572**	SOCS6	SOCS6_HUMAN	NCBI
**2573**	DHX58	DHX58_HUMAN	NCBI
**2574**	SLC38A1	S38A1_HUMAN	NCBI
**2575**	BTG3	BTG3_HUMAN	NCBI
**2576**	GLCCI1	GLCI1_HUMAN	NCBI
**2577**	GALNT1	GALT1_HUMAN	NCBI
**2578**	ARHGAP26	RHG26_HUMAN	NCBI
**2579**	FAM3C	FAM3C_HUMAN	NCBI
**2580**	NXN	NXN_HUMAN	NCBI
**2581**	OSBP	OSBP1_HUMAN	NCBI

No.	Symbol	Uniprot Name	Database
**2582**	EMX2	EMX2_HUMAN	NCBI
**2583**	RRAD	RAD_HUMAN	NCBI
**2584**	NLRP6	NLRP6_HUMAN	NCBI
**2585**	IL17RD	I17RD_HUMAN	NCBI
**2586**	ZG16B	PAUF_HUMAN	NCBI
**2587**	SEC22B	SC22B_HUMAN	NCBI
**2588**	ORM2	A1AG2_HUMAN	NCBI
**2589**	DACT1	DACT1_HUMAN	NCBI
**2590**	DAAM1	DAAM1_HUMAN	NCBI
**2591**	SIVA1	SIVA_HUMAN	NCBI
**2592**	SETD1B	SET1B_HUMAN	NCBI
**2593**	STX2	STX2_HUMAN	NCBI
**2594**	CDK10	CDK10_HUMAN	NCBI
**2595**	TTF1	TTF1_HUMAN	NCBI
**2596**	POM121	P121A_HUMAN	NCBI
**2597**	CCNG1	CCNG1_HUMAN	NCBI
**2598**	CYP2S1	CP2S1_HUMAN	NCBI
**2599**	SNX2	SNX2_HUMAN	NCBI
**2600**	GPC4	GPC4_HUMAN	NCBI
**2601**	WNT5B	WNT5B_HUMAN	NCBI
**2602**	SMS	SPSY_HUMAN	NCBI
**2603**	MT1G	MT1G_HUMAN	NCBI
**2604**	LYPD3	LYPD3_HUMAN	NCBI
**2605**	PDZK1IP1	PDZ1I_HUMAN	NCBI
**2606**	MAFG	MAFG_HUMAN	NCBI
**2607**	ESCO2	ESCO2_HUMAN	NCBI
**2608**	IFIT2	IFIT2_HUMAN	NCBI
**2609**	NEBL	NEBL_HUMAN	NCBI
**2610**	NCAPD2	CND1_HUMAN	NCBI
**2611**	IQGAP3	IQGA3_HUMAN	NCBI
**2612**	HSPA4L	HS74L_HUMAN	NCBI
**2613**	PMS1	PMS1_HUMAN	NCBI
**2614**	FOXP4	FOXP4_HUMAN	NCBI
**2615**	RGCC	RGCC_HUMAN	NCBI
**2616**	GALR1	GALR1_HUMAN	NCBI
**2617**	MATK	MATK_HUMAN	NCBI
**2618**	CDCA3	CDCA3_HUMAN	NCBI
**2619**	INA	AINX_HUMAN	NCBI
**2620**	RASSF6	RASF6_HUMAN	NCBI
**2621**	PRUNE1	PRUN1_HUMAN	NCBI
**2622**	ABCC10	MRP7_HUMAN	NCBI

No.	Symbol	Uniprot Name	Database
**2623**	DDX46	DDX46_HUMAN	NCBI
**2624**	TMCO1	TMCO1_HUMAN	NCBI
**2625**	LMO1	RBTN1_HUMAN	NCBI
**2626**	STYK1	STYK1_HUMAN	NCBI
**2627**	FAT4	FAT4_HUMAN	NCBI
**2628**	APBA1	APBA1_HUMAN	NCBI
**2629**	PLA2G4C	PA24C_HUMAN	NCBI
**2630**	THOC1	THOC1_HUMAN	NCBI
**2631**	PDLIM1	PDLI1_HUMAN	NCBI
**2632**	PRR11	PRR11_HUMAN	NCBI
**2633**	AKAP10	AKA10_HUMAN	NCBI
**2634**	PEAK1	PEAK1_HUMAN	NCBI
**2635**	SPIB	SPIB_HUMAN	NCBI
**2636**	TRIM2	TRIM2_HUMAN	NCBI
**2637**	SMUG1	SMUG1_HUMAN	NCBI
**2638**	KLK14	KLK14_HUMAN	NCBI
**2639**	SLC16A7	MOT2_HUMAN	NCBI
**2640**	PARP4	PARP4_HUMAN	NCBI
**2641**	RABGEF1	RABX5_HUMAN	NCBI
**2642**	HOXD3	HXD3_HUMAN	NCBI
**2643**	ACP2	PPAL_HUMAN	NCBI
**2644**	GTF2E1	T2EA_HUMAN	NCBI
**2645**	OAS2	OAS2_HUMAN	NCBI
**2646**	MYNN	MYNN_HUMAN	NCBI
**2647**	SIX2	SIX2_HUMAN	NCBI
**2648**	SEC62	SEC62_HUMAN	NCBI
**2649**	KHDRBS3	KHDR3_HUMAN	NCBI
**2650**	PSPH	SERB_HUMAN	NCBI
**2651**	KLF14	KLF14_HUMAN	NCBI
**2652**	SPRED2	SPRE2_HUMAN	NCBI
**2653**	FUBP3	FUBP3_HUMAN	NCBI
**2654**	SLC39A7	S39A7_HUMAN	NCBI
**2655**	ASIC2	ASIC2_HUMAN	NCBI
**2656**	ABCA2	ABCA2_HUMAN	NCBI
**2657**	SEMA3E	SEM3E_HUMAN	NCBI
**2658**	SLITRK1	SLIK1_HUMAN	NCBI
**2659**	NOL3	NOL3_HUMAN	NCBI
**2660**	CENPU	CENPU_HUMAN	NCBI
**2661**	GBP2	GBP2_HUMAN	NCBI
**2662**	CEACAM7	CEAM7_HUMAN	NCBI
**2663**	PPP2R5E	2A5E_HUMAN	NCBI

No.	Symbol	Uniprot Name	Database
**2664**	CPEB3	CPEB3_HUMAN	NCBI
**2665**	SRGAP2	SRGP2_HUMAN	NCBI
**2666**	LMTK3	LMTK3_HUMAN	NCBI
**2667**	RMI1	RMI1_HUMAN	NCBI
**2668**	GTF2IRD1	GT2D1_HUMAN	NCBI
**2669**	MARCKSL1	MRP_HUMAN	NCBI
**2670**	GPR37	GPR37_HUMAN	NCBI
**2671**	SLC25A10	DIC_HUMAN	NCBI
**2672**	PTBP3	PTBP3_HUMAN	NCBI
**2673**	RNF111	RN111_HUMAN	NCBI
**2674**	KDM5D	KDM5D_HUMAN	NCBI
**2675**	OSBPL3	OSBL3_HUMAN	NCBI
**2676**	MLLT10	AF10_HUMAN	NCBI
**2677**	ELF4	ELF4_HUMAN	NCBI
**2678**	MOB1A	MOB1A_HUMAN	NCBI
**2679**	EPHB3	EPHB3_HUMAN	NCBI
**2680**	RETNLB	RETNB_HUMAN	NCBI
**2681**	TRO	TROP_HUMAN	NCBI
**2682**	TM4SF5	T4S5_HUMAN	NCBI
**2683**	B3GALT5	B3GT5_HUMAN	NCBI
**2684**	SCARA5	SCAR5_HUMAN	NCBI
**2685**	TRIM16	TRI16_HUMAN	NCBI
**2686**	MLLT11	AF1Q_HUMAN	NCBI
**2687**	UQCRB	QCR7_HUMAN	NCBI
**2688**	PRDM14	PRD14_HUMAN	NCBI
**2689**	PYCR2	P5CR2_HUMAN	NCBI
**2690**	PATZ1	PATZ1_HUMAN	NCBI
**2691**	CLDN14	CLD14_HUMAN	NCBI
**2692**	AGGF1	AGGF1_HUMAN	NCBI
**2693**	TMEM8B	TMM8B_HUMAN	NCBI
**2694**	NPAT	NPAT_HUMAN	NCBI
**2695**	POLR1D	RPC22_HUMAN	NCBI
**2696**	POLR1D	RPAC2_HUMAN	NCBI
**2697**	BMP5	BMP5_HUMAN	NCBI
**2698**	STK25	STK25_HUMAN	NCBI
**2699**	DYNC1LI1	DC1L1_HUMAN	NCBI
**2700**	PPP1R13B	ASPP1_HUMAN	NCBI
**2701**	ACTBL2	ACTBL_HUMAN	NCBI
**2702**	BCAT2	BCAT2_HUMAN	NCBI
**2703**	GJC1	CXG1_HUMAN	NCBI
**2704**	PTBP2	PTBP2_HUMAN	NCBI

No.	Symbol	Uniprot Name	Database
**2705**	PARVB	PARVB_HUMAN	NCBI
**2706**	ITPKC	IP3KC_HUMAN	NCBI
**2707**	LY6E	LY6E_HUMAN	NCBI
**2708**	MPC1	MPC1_HUMAN	NCBI
**2709**	LRRFIP1	LRRF1_HUMAN	NCBI
**2710**	NFE2L3	NF2L3_HUMAN	NCBI
**2711**	OVOL2	OVOL2_HUMAN	NCBI
**2712**	ZNF24	ZNF24_HUMAN	NCBI
**2713**	AKAP4	AKAP4_HUMAN	NCBI
**2714**	TSC22D1	T22D1_HUMAN	NCBI
**2715**	CPNE3	CPNE3_HUMAN	NCBI
**2716**	GRIK3	GRIK3_HUMAN	NCBI
**2717**	DAPK2	DAPK2_HUMAN	NCBI
**2718**	PDP1	PDP1_HUMAN	NCBI
**2719**	LHPP	LHPP_HUMAN	NCBI
**2720**	ZFP36L2	TISD_HUMAN	NCBI
**2721**	FBXO22	FBX22_HUMAN	NCBI
**2722**	ONECUT1	HNF6_HUMAN	NCBI
**2723**	MEP1A	MEP1A_HUMAN	NCBI
**2724**	RGMA	RGMA_HUMAN	NCBI
**2725**	SOX13	SOX13_HUMAN	NCBI
**2726**	MEX3A	MEX3A_HUMAN	NCBI
**2727**	LRIG3	LRIG3_HUMAN	NCBI
**2728**	BRD1	BRD1_HUMAN	NCBI
**2729**	PLXND1	PLXD1_HUMAN	NCBI
**2730**	SGMS2	SMS2_HUMAN	NCBI
**2731**	BMP3	BMP3_HUMAN	NCBI
**2732**	CNOT2	CNOT2_HUMAN	NCBI
**2733**	ZNF281	ZN281_HUMAN	NCBI
**2734**	PLEKHA7	PKHA7_HUMAN	NCBI
**2735**	ITGBL1	ITGBL_HUMAN	NCBI
**2736**	DDX56	DDX56_HUMAN	NCBI
**2737**	USP44	UBP44_HUMAN	NCBI
**2738**	EEF1E1	MCA3_HUMAN	NCBI
**2739**	DDX27	DDX27_HUMAN	NCBI
**2740**	FABP6	FABP6_HUMAN	NCBI
**2741**	KIF3B	KIF3B_HUMAN	NCBI
**2742**	CCBE1	CCBE1_HUMAN	NCBI
**2743**	PLA2G3	PA2G3_HUMAN	NCBI
**2744**	KLF3	KLF3_HUMAN	NCBI
**2745**	TIFA	TIFA_HUMAN	NCBI

No.	Symbol	Uniprot Name	Database
**2746**	NFS1	NFS1_HUMAN	NCBI
**2747**	PLEKHG6	PKHG6_HUMAN	NCBI
**2748**	GPR4	GPR4_HUMAN	NCBI
**2749**	TUFT1	TUFT1_HUMAN	NCBI
**2750**	TELO2	TELO2_HUMAN	NCBI
**2751**	U2SURP	SR140_HUMAN	NCBI
**2752**	SHROOM2	SHRM2_HUMAN	NCBI
**2753**	SIK3	SIK3_HUMAN	NCBI
**2754**	ZNF750	ZN750_HUMAN	NCBI
**2755**	APC2	APCL_HUMAN	NCBI
**2756**	RHBDD1	RHBL4_HUMAN	NCBI
**2757**	COASY	COASY_HUMAN	NCBI
**2758**	PTPRR	PTPRR_HUMAN	NCBI
**2759**	UBE2J1	UB2J1_HUMAN	NCBI
**2760**	SLC25A22	GHC1_HUMAN	NCBI
**2761**	KIF18B	KI18B_HUMAN	NCBI
**2762**	STAG3	STAG3_HUMAN	NCBI
**2763**	TPBG	TPBG_HUMAN	NCBI
**2764**	PEBP4	PEBP4_HUMAN	NCBI
**2765**	UTP14A	UT14A_HUMAN	NCBI
**2766**	HPGDS	HPGDS_HUMAN	NCBI
**2767**	SLC9A2	SL9A2_HUMAN	NCBI
**2768**	NKD2	NKD2_HUMAN	NCBI
**2769**	ELOVL5	ELOV5_HUMAN	NCBI
**2770**	KDM3B	KDM3B_HUMAN	NCBI
**2771**	MARK1	MARK1_HUMAN	NCBI
**2772**	MCF2L	MCF2L_HUMAN	NCBI
**2773**	ALPK1	ALPK1_HUMAN	NCBI
**2774**	SYNPO2	SYNP2_HUMAN	NCBI
**2775**	ZNF703	ZN703_HUMAN	NCBI
**2776**	VPS16	VPS16_HUMAN	NCBI
**2777**	PRPS2	PRPS2_HUMAN	NCBI
**2778**	PLA1A	PLA1A_HUMAN	NCBI
**2779**	TRIM65	TRI65_HUMAN	NCBI
**2780**	DCBLD2	DCBD2_HUMAN	NCBI
**2781**	MRPS18B	RT18B_HUMAN	NCBI
**2782**	DKK4	DKK4_HUMAN	NCBI
**2783**	KLF17	KLF17_HUMAN	NCBI
**2784**	LDOC1	LDOC1_HUMAN	NCBI
**2785**	GINS4	SLD5_HUMAN	NCBI
**2786**	SRSF11	SRS11_HUMAN	NCBI

No.	Symbol	Uniprot Name	Database
**2787**	RINT1	RINT1_HUMAN	NCBI
**2788**	PTPRT	PTPRT_HUMAN	NCBI
**2789**	PTP4A2	TP4A2_HUMAN	NCBI
**2790**	ADAP1	ADAP1_HUMAN	NCBI
**2791**	MPZL1	MPZL1_HUMAN	NCBI
**2792**	PTPN9	PTN9_HUMAN	NCBI
**2793**	PNO1	PNO1_HUMAN	NCBI
**2794**	CYP2W1	CP2W1_HUMAN	NCBI
**2795**	ACADSB	ACDSB_HUMAN	NCBI
**2796**	HOXB1	HXB1_HUMAN	NCBI
**2797**	ALKBH2	ALKB2_HUMAN	NCBI
**2798**	PTGES2	PGES2_HUMAN	NCBI
**2799**	LZTS1	LZTS1_HUMAN	NCBI
**2800**	BOK	BOK_HUMAN	NCBI
**2801**	SMC5	SMC5_HUMAN	NCBI
**2802**	ANGPTL6	ANGL6_HUMAN	NCBI
**2803**	BZW2	5MP1_HUMAN	NCBI
**2804**	FZD8	FZD8_HUMAN	NCBI
**2805**	EFNB3	EFNB3_HUMAN	NCBI
**2806**	NOP14	NOP14_HUMAN	NCBI
**2807**	SETD3	SETD3_HUMAN	NCBI
**2808**	MIEN1	MIEN1_HUMAN	NCBI
**2809**	LCORL	LCORL_HUMAN	NCBI
**2810**	DUSP2	DUSP2_HUMAN	NCBI
**2811**	CDCA7	CDCA7_HUMAN	NCBI
**2812**	MYCBP	MYCBP_HUMAN	NCBI
**2813**	CDR2	CDR2_HUMAN	NCBI
**2814**	ARID3B	ARI3B_HUMAN	NCBI
**2815**	NIFK	MK67I_HUMAN	NCBI
**2816**	ATF7	ATF7_HUMAN	NCBI
**2817**	SOX8	SOX8_HUMAN	NCBI
**2818**	DHRS2	DHRS2_HUMAN	NCBI
**2819**	CEP78	CEP78_HUMAN	NCBI
**2820**	SNIP1	SNIP1_HUMAN	NCBI
**2821**	PRELP	PRELP_HUMAN	NCBI
**2822**	HOXB6	HXB6_HUMAN	NCBI
**2823**	NOC2L	NOC2L_HUMAN	NCBI
**2824**	CCNK	CCNK_HUMAN	NCBI
**2825**	CA11	CAH11_HUMAN	NCBI
**2826**	DACT2	DACT2_HUMAN	NCBI
**2827**	USP17L2	U17L2_HUMAN	NCBI

No.	Symbol	Uniprot Name	Database
**2828**	POU5F1B	P5F1B_HUMAN	NCBI
**2829**	CEP72	CEP72_HUMAN	NCBI
**2830**	NELFCD	NELFD_HUMAN	NCBI
**2831**	MKRN1	MKRN1_HUMAN	NCBI
**2832**	RAB3D	RAB3D_HUMAN	NCBI
**2833**	PLP2	PLP2_HUMAN	NCBI
**2834**	JAM2	JAM2_HUMAN	NCBI
**2835**	PFDN5	PFD5_HUMAN	NCBI
**2836**	DNER	DNER_HUMAN	NCBI
**2837**	EXOSC4	EXOS4_HUMAN	NCBI
**2838**	RIOK1	RIOK1_HUMAN	NCBI
**2839**	CDK3	CDK3_HUMAN	NCBI
**2840**	ARHGAP5	RHG05_HUMAN	NCBI
**2841**	POLE2	DPOE2_HUMAN	NCBI
**2842**	SCGB2A1	SG2A1_HUMAN	NCBI
**2843**	DNAJC2	DNJC2_HUMAN	NCBI
**2844**	ABLIM1	ABLM1_HUMAN	NCBI
**2845**	CRIP1	CRIP1_HUMAN	NCBI
**2846**	ARL4C	ARL4C_HUMAN	NCBI
**2847**	FAM98A	FA98A_HUMAN	NCBI
**2848**	TNFRSF19	TNR19_HUMAN	NCBI
**2849**	PFDN1	PFD1_HUMAN	NCBI
**2850**	SNX10	SNX10_HUMAN	NCBI
**2851**	CITED1	CITE1_HUMAN	NCBI
**2852**	ARL11	ARL11_HUMAN	NCBI
**2853**	KHK	KHK_HUMAN	NCBI
**2854**	GCNT2	GNT2A_HUMAN	NCBI
**2855**	GALR2	GALR2_HUMAN	NCBI
**2856**	MACROD2	MACD2_HUMAN	NCBI
**2857**	PLS1	PLSI_HUMAN	NCBI
**2858**	SRPX2	SRPX2_HUMAN	NCBI
**2859**	SOX12	SOX12_HUMAN	NCBI
**2860**	INTS6	INT6_HUMAN	NCBI
**2861**	TRIM23	TRI23_HUMAN	NCBI
**2862**	SAPCD2	SAPC2_HUMAN	NCBI
**2863**	EXOSC8	EXOS8_HUMAN	NCBI
**2864**	MPC2	MPC2_HUMAN	NCBI
**2865**	ARRDC3	ARRD3_HUMAN	NCBI
**2866**	ARHGAP29	RHG29_HUMAN	NCBI
**2867**	CLEC4A	CLC4A_HUMAN	NCBI
**2868**	RNF6	RNF6_HUMAN	NCBI

No.	Symbol	Uniprot Name	Database
**2869**	MED28	MED28_HUMAN	NCBI
**2870**	LHX4	LHX4_HUMAN	NCBI
**2871**	ZNF331	ZN331_HUMAN	NCBI
**2872**	CHPF	CHSS2_HUMAN	NCBI
**2873**	CLK2	CLK2_HUMAN	NCBI
**2874**	RNF114	RN114_HUMAN	NCBI
**2875**	NRBP1	NRBP_HUMAN	NCBI
**2876**	ADAMTS14	ATS14_HUMAN	NCBI
**2877**	GINS1	PSF1_HUMAN	NCBI
**2878**	CRTC3	CRTC3_HUMAN	NCBI
**2879**	CST4	CYTS_HUMAN	NCBI
**2880**	POGLUT1	PGLT1_HUMAN	NCBI
**2881**	EVL	EVL_HUMAN	NCBI
**2882**	TRIM47	TRI47_HUMAN	NCBI
**2883**	REPIN1	REPI1_HUMAN	NCBI
**2884**	HKDC1	HKDC1_HUMAN	NCBI
**2885**	CAB39	CAB39_HUMAN	NCBI
**2886**	ATG10	ATG10_HUMAN	NCBI
**2887**	HOXC9	HXC9_HUMAN	NCBI
**2888**	BMF	BMF_HUMAN	NCBI
**2889**	CHERP	CHERP_HUMAN	NCBI
**2890**	BAZ2B	BAZ2B_HUMAN	NCBI
**2891**	TAS2R16	T2R16_HUMAN	NCBI
**2892**	NMNAT2	NMNA2_HUMAN	NCBI
**2893**	PCGF1	PCGF1_HUMAN	NCBI
**2894**	SKAP1	SKAP1_HUMAN	NCBI
**2895**	PCDH8	PCDH8_HUMAN	NCBI
**2896**	BRIX1	BRX1_HUMAN	NCBI
**2897**	MT1F	MT1F_HUMAN	NCBI
**2898**	CUEDC2	CUED2_HUMAN	NCBI
**2899**	YARS2	SYYM_HUMAN	NCBI
**2900**	PHLDB2	PHLB2_HUMAN	NCBI
**2901**	WNT6	WNT6_HUMAN	NCBI
**2902**	MORC1	MORC1_HUMAN	NCBI
**2903**	SLC9A9	SL9A9_HUMAN	NCBI
**2904**	RRP9	U3IP2_HUMAN	NCBI
**2905**	NKX6-1	NKX61_HUMAN	NCBI
**2906**	FOXH1	FOXH1_HUMAN	NCBI
**2907**	HES6	HES6_HUMAN	NCBI
**2908**	CEP63	CEP63_HUMAN	NCBI
**2909**	TESC	CHP3_HUMAN	NCBI

No.	Symbol	Uniprot Name	Database
**2910**	EAF2	EAF2_HUMAN	NCBI
**2911**	DIS3L2	DI3L2_HUMAN	NCBI
**2912**	GSTT2	GST2_HUMAN	NCBI
**2913**	AZIN1	AZIN1_HUMAN	NCBI
**2914**	DEPDC1B	DEP1B_HUMAN	NCBI
**2915**	NR2F6	NR2F6_HUMAN	NCBI
**2916**	PCID2	PCID2_HUMAN	NCBI
**2917**	TLE4	TLE4_HUMAN	NCBI
**2918**	TNFAIP8L1	TP8L1_HUMAN	NCBI
**2919**	FMN2	FMN2_HUMAN	NCBI
**2920**	PDE7A	PDE7A_HUMAN	NCBI
**2921**	ADAMTS12	ATS12_HUMAN	NCBI
**2922**	CCL14	CCL14_HUMAN	NCBI
**2923**	WHRN	WHRN_HUMAN	NCBI
**2924**	CNPY2	CNPY2_HUMAN	NCBI
**2925**	TEP1	TEP1_HUMAN	NCBI
**2926**	SCUBE2	SCUB2_HUMAN	NCBI
**2927**	SH3GL3	SH3G3_HUMAN	NCBI
**2928**	PDE4DIP	MYOME_HUMAN	NCBI
**2929**	TP53INP2	T53I2_HUMAN	NCBI
**2930**	GINS3	PSF3_HUMAN	NCBI
**2931**	KCTD12	KCD12_HUMAN	NCBI
**2932**	SNTB1	SNTB1_HUMAN	NCBI
**2933**	HIPK1	HIPK1_HUMAN	NCBI
**2934**	GRINA	LFG1_HUMAN	NCBI
**2935**	SLC25A24	SCMC1_HUMAN	NCBI
**2936**	MMP21	MMP21_HUMAN	NCBI
**2937**	ADAM23	ADA23_HUMAN	NCBI
**2938**	KCNE4	KCNE4_HUMAN	NCBI
**2939**	TMEM59	TMM59_HUMAN	NCBI
**2940**	GOSR1	GOSR1_HUMAN	NCBI
**2941**	GET4	GET4_HUMAN	NCBI
**2942**	COPS7B	CSN7B_HUMAN	NCBI
**2943**	SOX15	SOX15_HUMAN	NCBI
**2944**	ZFP57	ZFP57_HUMAN	NCBI
**2945**	BUD23	BUD23_HUMAN	NCBI
**2946**	GUCA2A	GUC2A_HUMAN	NCBI
**2947**	UHMK1	UHMK1_HUMAN	NCBI
**2948**	MUC3A	MUC3A_HUMAN	NCBI
**2949**	TRAK1	TRAK1_HUMAN	NCBI
**2950**	CLEC10A	CLC10_HUMAN	NCBI

No.	Symbol	Uniprot Name	Database
**2951**	PHKB	KPBB_HUMAN	NCBI
**2952**	RPS27L	RS27L_HUMAN	NCBI
**2953**	RASSF8	RASF8_HUMAN	NCBI
**2954**	FSTL5	FSTL5_HUMAN	NCBI
**2955**	BLCAP	BLCAP_HUMAN	NCBI
**2956**	LHX6	LHX6_HUMAN	NCBI
**2957**	MYO1A	MYO1A_HUMAN	NCBI
**2958**	NOC3L	NOC3L_HUMAN	NCBI
**2959**	ARID4B	ARI4B_HUMAN	NCBI
**2960**	CCSER1	CCSE1_HUMAN	NCBI
**2961**	B9D2	B9D2_HUMAN	NCBI
**2962**	NPM3	NPM3_HUMAN	NCBI
**2963**	TRIP4	TRIP4_HUMAN	NCBI
**2964**	BCKDK	BCKD_HUMAN	NCBI
**2965**	PRUNE2	PRUN2_HUMAN	NCBI
**2966**	CPA4	CBPA4_HUMAN	NCBI
**2967**	RFXANK	RFXK_HUMAN	NCBI
**2968**	GREM2	GREM2_HUMAN	NCBI
**2969**	RNF135	RN135_HUMAN	NCBI
**2970**	SLC15A4	S15A4_HUMAN	NCBI
**2971**	ECI2	ECI2_HUMAN	NCBI
**2972**	AGR3	AGR3_HUMAN	NCBI
**2973**	FAM107A	F107A_HUMAN	NCBI
**2974**	MICALL1	MILK1_HUMAN	NCBI
**2975**	GPATCH1	GPTC1_HUMAN	NCBI
**2976**	CMTM3	CKLF3_HUMAN	NCBI
**2977**	UTP4	UTP4_HUMAN	NCBI
**2978**	IMP3	IMP3_HUMAN	NCBI
**2979**	NCAPG2	CNDG2_HUMAN	NCBI
**2980**	APMAP	APMAP_HUMAN	NCBI
**2981**	LARP6	LARP6_HUMAN	NCBI
**2982**	DMTF1	DMTF1_HUMAN	NCBI
**2983**	NME3	NDK3_HUMAN	NCBI
**2984**	CRB3	CRUM3_HUMAN	NCBI
**2985**	BEX2	BEX2_HUMAN	NCBI
**2986**	UPF3A	REN3A_HUMAN	NCBI
**2987**	FILIP1L	FIL1L_HUMAN	NCBI
**2988**	PFDN4	PFD4_HUMAN	NCBI
**2989**	KLK12	KLK12_HUMAN	NCBI
**2990**	CYBRD1	CYBR1_HUMAN	NCBI
**2991**	LGR6	LGR6_HUMAN	NCBI

No.	Symbol	Uniprot Name	Database
**2992**	MOAP1	MOAP1_HUMAN	NCBI
**2993**	MAF1	MAF1_HUMAN	NCBI
**2994**	CLDN8	CLD8_HUMAN	NCBI
**2995**	HOXB8	HXB8_HUMAN	NCBI
**2996**	SCGN	SEGN_HUMAN	NCBI
**2997**	GMFG	GMFG_HUMAN	NCBI
**2998**	SPON1	SPON1_HUMAN	NCBI
**2999**	ATAD1	ATAD1_HUMAN	NCBI
**3000**	MLXIP	MLXIP_HUMAN	NCBI
**3001**	PDE9A	PDE9A_HUMAN	NCBI
**3002**	ANGPTL1	ANGL1_HUMAN	NCBI
**3003**	MRGBP	MRGBP_HUMAN	NCBI
**3004**	INPP4A	INP4A_HUMAN	NCBI
**3005**	RBM47	RBM47_HUMAN	NCBI
**3006**	RNF128	RN128_HUMAN	NCBI
**3007**	SEC11A	SC11A_HUMAN	NCBI
**3008**	EGFL6	EGFL6_HUMAN	NCBI
**3009**	MANBA	MANBA_HUMAN	NCBI
**3010**	DSN1	DSN1_HUMAN	NCBI
**3011**	ADAMTS8	ATS8_HUMAN	NCBI
**3012**	LSM12	LSM12_HUMAN	NCBI
**3013**	KNTC1	KNTC1_HUMAN	NCBI
**3014**	UPK1A	UPK1A_HUMAN	NCBI
**3015**	ADCY2	ADCY2_HUMAN	NCBI
**3016**	PCBD2	PHS2_HUMAN	NCBI
**3017**	DLX6	DLX6_HUMAN	NCBI
**3018**	GMDS	GMDS_HUMAN	NCBI
**3019**	NOP16	NOP16_HUMAN	NCBI
**3020**	ACAA2	THIM_HUMAN	NCBI
**3021**	GLIS2	GLIS2_HUMAN	NCBI
**3022**	KYNU	KYNU_HUMAN	NCBI
**3023**	ZKSCAN3	ZKSC3_HUMAN	NCBI
**3024**	TUBGCP4	GCP4_HUMAN	NCBI
**3025**	NKD1	NKD1_HUMAN	NCBI
**3026**	FEZF1	FEZF1_HUMAN	NCBI
**3027**	TCF19	TCF19_HUMAN	NCBI
**3028**	GLYR1	GLYR1_HUMAN	NCBI
**3029**	ENO3	ENOB_HUMAN	NCBI
**3030**	PIGU	PIGU_HUMAN	NCBI
**3031**	UBE2Q1	UB2Q1_HUMAN	NCBI
**3032**	SIGLEC6	SIGL6_HUMAN	NCBI

No.	Symbol	Uniprot Name	Database
**3033**	CADPS	CAPS1_HUMAN	NCBI
**3034**	MUCL1	MUCL1_HUMAN	NCBI
**3035**	ZC3H13	ZC3HD_HUMAN	NCBI
**3036**	ZNF292	ZN292_HUMAN	NCBI
**3037**	EFNA2	EFNA2_HUMAN	NCBI
**3038**	PHF20	PHF20_HUMAN	NCBI
**3039**	UBE4A	UBE4A_HUMAN	NCBI
**3040**	GALNT4	GALT4_HUMAN	NCBI
**3041**	KDM4D	KDM4D_HUMAN	NCBI
**3042**	FUT9	FUT9_HUMAN	NCBI
**3043**	SSPN	SSPN_HUMAN	NCBI
**3044**	PCIF1	CAPAM_HUMAN	NCBI
**3045**	TNFAIP8L3	TP8L3_HUMAN	NCBI
**3046**	FRMD6	FRMD6_HUMAN	NCBI
**3047**	MIIP	MIIP_HUMAN	NCBI
**3048**	SLC35A3	S35A3_HUMAN	NCBI
**3049**	EI24	EI24_HUMAN	NCBI
**3050**	MAGEA6	MAGA6_HUMAN	NCBI
**3051**	PLEK2	PLEK2_HUMAN	NCBI
**3052**	IP6K2	IP6K2_HUMAN	NCBI
**3053**	NTSR2	NTR2_HUMAN	NCBI
**3054**	TAF1C	TAF1C_HUMAN	NCBI
**3055**	CSRP2	CSRP2_HUMAN	NCBI
**3056**	ATMIN	ATMIN_HUMAN	NCBI
**3057**	RASGRF2	RGRF2_HUMAN	NCBI
**3058**	HYAL3	HYAL3_HUMAN	NCBI
**3059**	ZIC5	ZIC5_HUMAN	NCBI
**3060**	NETO2	NETO2_HUMAN	NCBI
**3061**	SIX4	SIX4_HUMAN	NCBI
**3062**	RRP15	RRP15_HUMAN	NCBI
**3063**	SLC8B1	NCLX_HUMAN	NCBI
**3064**	PTPN4	PTN4_HUMAN	NCBI
**3065**	ACOXL	ACOXL_HUMAN	NCBI
**3066**	GCNT3	GCNT3_HUMAN	NCBI
**3067**	PUS7	PUS7_HUMAN	NCBI
**3068**	SPNS2	SPNS2_HUMAN	NCBI
**3069**	ZNF687	ZN687_HUMAN	NCBI
**3070**	FBXW4	FBXW4_HUMAN	NCBI
**3071**	ATOH8	ATOH8_HUMAN	NCBI
**3072**	PPP1R3C	PPR3C_HUMAN	NCBI
**3073**	TBPL1	TBPL1_HUMAN	NCBI

No.	Symbol	Uniprot Name	Database
**3074**	ABCA8	ABCA8_HUMAN	NCBI
**3075**	MYSM1	MYSM1_HUMAN	NCBI
**3076**	IL36B	IL36B_HUMAN	NCBI
**3077**	HOXD8	HXD8_HUMAN	NCBI
**3078**	DHX32	DHX32_HUMAN	NCBI
**3079**	SOX21	SOX21_HUMAN	NCBI
**3080**	SLC35C1	FUCT1_HUMAN	NCBI
**3081**	KIAA1549	K1549_HUMAN	NCBI
**3082**	NEK8	NEK8_HUMAN	NCBI
**3083**	FBXL2	FBXL2_HUMAN	NCBI
**3084**	ZNF146	OZF_HUMAN	NCBI
**3085**	SYT13	SYT13_HUMAN	NCBI
**3086**	PEX26	PEX26_HUMAN	NCBI
**3087**	BCL2L14	B2L14_HUMAN	NCBI
**3088**	ADAMDEC1	ADEC1_HUMAN	NCBI
**3089**	CA10	CAH10_HUMAN	NCBI
**3090**	ADPGK	ADPGK_HUMAN	NCBI
**3091**	CCDC66	CCD66_HUMAN	NCBI
**3092**	ETV7	ETV7_HUMAN	NCBI
**3093**	POTEE	POTEE_HUMAN	NCBI
**3094**	BET1L	BET1L_HUMAN	NCBI
**3095**	TM9SF2	TM9S2_HUMAN	NCBI
**3096**	SYPL1	SYPL1_HUMAN	NCBI
**3097**	SLC25A28	MFRN2_HUMAN	NCBI
**3098**	MEMO1	MEMO1_HUMAN	NCBI
**3099**	TRIM66	TRI66_HUMAN	NCBI
**3100**	OGDHL	OGDHL_HUMAN	NCBI
**3101**	CLK3	CLK3_HUMAN	NCBI
**3102**	AMIGO2	AMGO2_HUMAN	NCBI
**3103**	ADGRF5	AGRF5_HUMAN	NCBI
**3104**	FOXO6	FOXO6_HUMAN	NCBI
**3105**	GDF1	GDF1_HUMAN	NCBI
**3106**	CENPI	CENPI_HUMAN	NCBI
**3107**	SLC17A9	S17A9_HUMAN	NCBI
**3108**	SGPP1	SGPP1_HUMAN	NCBI
**3109**	SCAI	SCAI_HUMAN	NCBI
**3110**	TOM1L1	TM1L1_HUMAN	NCBI
**3111**	IQANK1	IQAK1_HUMAN	NCBI
**3112**	IQANK1	A0A1B0GUK7_HUMAN	NCBI
**3113**	B4GALT5	B4GT5_HUMAN	NCBI
**3114**	HNRNPLL	HNRLL_HUMAN	NCBI

No.	Symbol	Uniprot Name	Database
**3115**	NEUROG1	NGN1_HUMAN	NCBI
**3116**	AK6	KAD6_HUMAN	NCBI
**3117**	DNPEP	DNPEP_HUMAN	NCBI
**3118**	MDFIC	MDFIC_HUMAN	NCBI
**3119**	ATG9B	ATG9B_HUMAN	NCBI
**3120**	KRT80	K2C80_HUMAN	NCBI
**3121**	CHID1	CHID1_HUMAN	NCBI
**3122**	BATF3	BATF3_HUMAN	NCBI
**3123**	RHBDD2	RHBD2_HUMAN	NCBI
**3124**	SFMBT1	SMBT1_HUMAN	NCBI
**3125**	PDRG1	PDRG1_HUMAN	NCBI
**3126**	NOC4L	NOC4L_HUMAN	NCBI
**3127**	TEX11	TEX11_HUMAN	NCBI
**3128**	TRIM36	TRI36_HUMAN	NCBI
**3129**	RRAGB	RRAGB_HUMAN	NCBI
**3130**	GSDMC	GSDMC_HUMAN	NCBI
**3131**	CLDN12	CLD12_HUMAN	NCBI
**3132**	PRLHR	PRLHR_HUMAN	NCBI
**3133**	USP49	UBP49_HUMAN	NCBI
**3134**	LMOD1	LMOD1_HUMAN	NCBI
**3135**	N6AMT1	N6MT1_HUMAN	NCBI
**3136**	TIPIN	TIPIN_HUMAN	NCBI
**3137**	KRT23	K1C23_HUMAN	NCBI
**3138**	ABHD6	ABHD6_HUMAN	NCBI
**3139**	CLEC2B	CLC2B_HUMAN	NCBI
**3140**	PHRF1	PHRF1_HUMAN	NCBI
**3141**	STK17A	ST17A_HUMAN	NCBI
**3142**	ZNF746	ZN746_HUMAN	NCBI
**3143**	NUDCD1	NUDC1_HUMAN	NCBI
**3144**	CPA6	CBPA6_HUMAN	NCBI
**3145**	DDHD1	DDHD1_HUMAN	NCBI
**3146**	IL20RA	I20RA_HUMAN	NCBI
**3147**	CMTM4	CKLF4_HUMAN	NCBI
**3148**	PLXDC2	PXDC2_HUMAN	NCBI
**3149**	ATP11A	AT11A_HUMAN	NCBI
**3150**	SCRN1	SCRN1_HUMAN	NCBI
**3151**	CMTR1	CMTR1_HUMAN	NCBI
**3152**	LAGE3	LAGE3_HUMAN	NCBI
**3153**	TMUB1	TMUB1_HUMAN	NCBI
**3154**	HEYL	HEYL_HUMAN	NCBI
**3155**	PARP11	PAR11_HUMAN	NCBI

No.	Symbol	Uniprot Name	Database
**3156**	ITM2C	ITM2C_HUMAN	NCBI
**3157**	NANOGP8	NANP8_HUMAN	NCBI
**3158**	RTKN2	RTKN2_HUMAN	NCBI
**3159**	PHF20L1	P20L1_HUMAN	NCBI
**3160**	PHF14	PHF14_HUMAN	NCBI
**3161**	GID8	GID8_HUMAN	NCBI
**3162**	TRIM6	TRIM6_HUMAN	NCBI
**3163**	SLCO4A1	SO4A1_HUMAN	NCBI
**3164**	USP38	UBP38_HUMAN	NCBI
**3165**	FUT5	FUT5_HUMAN	NCBI
**3166**	YY2	TYY2_HUMAN	NCBI
**3167**	ART1	NAR1_HUMAN	NCBI
**3168**	TULP3	TULP3_HUMAN	NCBI
**3169**	HNF4G	HNF4G_HUMAN	NCBI
**3170**	CST5	CYTD_HUMAN	NCBI
**3171**	CACUL1	CACL1_HUMAN	NCBI
**3172**	PASD1	PASD1_HUMAN	NCBI
**3173**	PGPEP1	PGPI_HUMAN	NCBI
**3174**	PNMA8A	PNM8A_HUMAN	NCBI
**3175**	ADGRL4	AGRL4_HUMAN	NCBI
**3176**	SLC9A8	SL9A8_HUMAN	NCBI
**3177**	RUBCNL	PACER_HUMAN	NCBI
**3178**	B4GALT3	B4GT3_HUMAN	NCBI
**3179**	ZRANB1	ZRAN1_HUMAN	NCBI
**3180**	SH2D4A	SH24A_HUMAN	NCBI
**3181**	SLCO3A1	SO3A1_HUMAN	NCBI
**3182**	SEZ6L2	SE6L2_HUMAN	NCBI
**3183**	CPEB2	CPEB2_HUMAN	NCBI
**3184**	FAM98B	FA98B_HUMAN	NCBI
**3185**	PSG9	PSG9_HUMAN	NCBI
**3186**	MUC21	MUC21_HUMAN	NCBI
**3187**	TRIM58	TRI58_HUMAN	NCBI
**3188**	IRX5	IRX5_HUMAN	NCBI
**3189**	MRPL43	RM43_HUMAN	NCBI
**3190**	AGAP3	AGAP3_HUMAN	NCBI
**3191**	B3GNT8	B3GN8_HUMAN	NCBI
**3192**	SARDH	SARDH_HUMAN	NCBI
**3193**	STK31	STK31_HUMAN	NCBI
**3194**	TSPYL5	TSYL5_HUMAN	NCBI
**3195**	BRSK1	BRSK1_HUMAN	NCBI
**3196**	QSOX2	QSOX2_HUMAN	NCBI

No.	Symbol	Uniprot Name	Database
**3197**	SRGAP1	SRGP1_HUMAN	NCBI
**3198**	ST6GALNAC2	SIA7B_HUMAN	NCBI
**3199**	TSPAN6	TSN6_HUMAN	NCBI
**3200**	CHSY1	CHSS1_HUMAN	NCBI
**3201**	MDFI	MDFI_HUMAN	NCBI
**3202**	ODAM	ODAM_HUMAN	NCBI
**3203**	TCHH	TRHY_HUMAN	NCBI
**3204**	CDH10	CAD10_HUMAN	NCBI
**3205**	HIBCH	HIBCH_HUMAN	NCBI
**3206**	TMEM158	TM158_HUMAN	NCBI
**3207**	ACAP2	ACAP2_HUMAN	NCBI
**3208**	CBFA2T2	MTG8R_HUMAN	NCBI
**3209**	HERC3	HERC3_HUMAN	NCBI
**3210**	PARD3B	PAR3L_HUMAN	NCBI
**3211**	SUMF2	SUMF2_HUMAN	NCBI
**3212**	SPP2	SPP24_HUMAN	NCBI
**3213**	GTF3A	TF3A_HUMAN	NCBI
**3214**	TMBIM1	LFG3_HUMAN	NCBI
**3215**	ANKLE1	ANKL1_HUMAN	NCBI
**3216**	METTL17	MET17_HUMAN	NCBI
**3217**	PLXDC1	PLDX1_HUMAN	NCBI
**3218**	NEK4	NEK4_HUMAN	NCBI
**3219**	TPST1	TPST1_HUMAN	NCBI
**3220**	PRIMA1	PRIMA_HUMAN	NCBI
**3221**	WDR76	WDR76_HUMAN	NCBI
**3222**	DHRS9	DHRS9_HUMAN	NCBI
**3223**	CDK20	CDK20_HUMAN	NCBI
**3224**	CXXC4	CXXC4_HUMAN	NCBI
**3225**	TMPRSS13	TMPSD_HUMAN	NCBI
**3226**	POU3F3	PO3F3_HUMAN	NCBI
**3227**	URB1	NPA1P_HUMAN	NCBI
**3228**	ADAMTS16	ATS16_HUMAN	NCBI
**3229**	BICRA	BICRA_HUMAN	NCBI
**3230**	SPATA18	MIEAP_HUMAN	NCBI
**3231**	TRIM39	TRI39_HUMAN	NCBI
**3232**	ADGRA2	AGRA2_HUMAN	NCBI
**3233**	TMEM200A	T200A_HUMAN	NCBI
**3234**	RCAN2	RCAN2_HUMAN	NCBI
**3235**	MIS18A	MS18A_HUMAN	NCBI
**3236**	ASB9	ASB9_HUMAN	NCBI
**3237**	NAA40	NAA40_HUMAN	NCBI

No.	Symbol	Uniprot Name	Database
**3238**	SEMA3D	SEM3D_HUMAN	NCBI
**3239**	TNN	TENN_HUMAN	NCBI
**3240**	ANXA13	ANX13_HUMAN	NCBI
**3241**	CABLES2	CABL2_HUMAN	NCBI
**3242**	TMEM45A	TM45A_HUMAN	NCBI
**3243**	GNG4	GBG4_HUMAN	NCBI
**3244**	CDKL3	CDKL3_HUMAN	NCBI
**3245**	SLC51B	OSTB_HUMAN	NCBI
**3246**	CLCA4	CLCA4_HUMAN	NCBI
**3247**	NEK11	NEK11_HUMAN	NCBI
**3248**	ARHGAP6	RHG06_HUMAN	NCBI
**3249**	APOL3	APOL3_HUMAN	NCBI
**3250**	UNC5D	UNC5D_HUMAN	NCBI
**3251**	ASAP3	ASAP3_HUMAN	NCBI
**3252**	DAAM2	DAAM2_HUMAN	NCBI
**3253**	OLFM2	NOE2_HUMAN	NCBI
**3254**	GALR3	GALR3_HUMAN	NCBI
**3255**	FNDC1	FNDC1_HUMAN	NCBI
**3256**	CATSPER3	CTSR3_HUMAN	NCBI
**3257**	ANKZF1	ANKZ1_HUMAN	NCBI
**3258**	CCDC137	CC137_HUMAN	NCBI
**3259**	MORC4	MORC4_HUMAN	NCBI
**3260**	SNX16	SNX16_HUMAN	NCBI
**3261**	FLRT3	FLRT3_HUMAN	NCBI
**3262**	MAP9	MAP9_HUMAN	NCBI
**3263**	WDTC1	WDTC1_HUMAN	NCBI
**3264**	CILP2	CILP2_HUMAN	NCBI
**3265**	APCDD1	APCD1_HUMAN	NCBI
**3266**	NOTUM	NOTUM_HUMAN	NCBI
**3267**	CELF4	CELF4_HUMAN	NCBI
**3268**	TRIM55	TRI55_HUMAN	NCBI
**3269**	ARHGAP15	RHG15_HUMAN	NCBI
**3270**	GDPD5	GDPD5_HUMAN	NCBI
**3271**	STK17B	ST17B_HUMAN	NCBI
**3272**	CENPK	CENPK_HUMAN	NCBI
**3273**	B3GALT4	B3GT4_HUMAN	NCBI
**3274**	SMAP1	SMAP1_HUMAN	NCBI
**3275**	RAI2	RAI2_HUMAN	NCBI
**3276**	LONRF1	LONF1_HUMAN	NCBI
**3277**	ADHFE1	HOT_HUMAN	NCBI
**3278**	BARX2	BARX2_HUMAN	NCBI

No.	Symbol	Uniprot Name	Database
**3279**	DESI2	DESI2_HUMAN	NCBI
**3280**	SENP5	SENP5_HUMAN	NCBI
**3281**	SLC16A9	MOT9_HUMAN	NCBI
**3282**	DPPA2	DPPA2_HUMAN	NCBI
**3283**	UBE2Q2	UB2Q2_HUMAN	NCBI
**3284**	THSD1	THSD1_HUMAN	NCBI
**3285**	GPR137	G137A_HUMAN	NCBI
**3286**	ISX	ISX_HUMAN	NCBI
**3287**	STK16	STK16_HUMAN	NCBI
**3288**	ATG4D	ATG4D_HUMAN	NCBI
**3289**	MICALL2	MILK2_HUMAN	NCBI
**3290**	ANKRD6	ANKR6_HUMAN	NCBI
**3291**	ASB3	ASB3_HUMAN	NCBI
**3292**	AFAP1L1	AF1L1_HUMAN	NCBI
**3293**	ZYG11B	ZY11B_HUMAN	NCBI
**3294**	TFAP2E	AP2E_HUMAN	NCBI
**3295**	LGALS12	LEG12_HUMAN	NCBI
**3296**	TMEM240	TM240_HUMAN	NCBI
**3297**	TMEM176A	T176A_HUMAN	NCBI
**3298**	ADAMTSL3	ATL3_HUMAN	NCBI
**3299**	COMMD10	COMDA_HUMAN	NCBI
**3300**	DIRAS1	DIRA1_HUMAN	NCBI
**3301**	AK3	KAD3_HUMAN	NCBI
**3302**	TUSC1	TUSC1_HUMAN	NCBI
**3303**	MPP3	MPP3_HUMAN	NCBI
**3304**	TM4SF4	T4S4_HUMAN	NCBI
**3305**	MUC12	MUC12_HUMAN	NCBI
**3306**	CLDN23	CLD23_HUMAN	NCBI
**3307**	RAB3C	RAB3C_HUMAN	NCBI
**3308**	ZG16	ZG16_HUMAN	NCBI
**3309**	CTPS2	PYRG2_HUMAN	NCBI
**3310**	FOXS1	FOXS1_HUMAN	NCBI
**3311**	RPL22L1	RL22L_HUMAN	NCBI
**3312**	DENND4C	DEN4C_HUMAN	NCBI
**3313**	CA7	CAH7_HUMAN	NCBI
**3314**	CERS4	CERS4_HUMAN	NCBI
**3315**	DTX2	DTX2_HUMAN	NCBI
**3316**	PCDHGC3	PCDGK_HUMAN	NCBI
**3317**	HECTD2	HECD2_HUMAN	NCBI
**3318**	ZNF492	ZN492_HUMAN	NCBI
**3319**	ZNF768	ZN768_HUMAN	NCBI

No.	Symbol	Uniprot Name	Database
**3320**	FBXO8	FBX8_HUMAN	NCBI
**3321**	SPZ1	SPZ1_HUMAN	NCBI
**3322**	RNF186	RN186_HUMAN	NCBI
**3323**	CHRDL2	CRDL2_HUMAN	NCBI
**3324**	HOXA6	HXA6_HUMAN	NCBI
**3325**	ZNF263	ZN263_HUMAN	NCBI
**3326**	UBXN11	UBX11_HUMAN	NCBI
**3327**	CMTM2	CKLF2_HUMAN	NCBI
**3328**	ARHGAP9	RHG09_HUMAN	NCBI
**3329**	ZNF32	ZNF32_HUMAN	NCBI
**3330**	FLYWCH1	FWCH1_HUMAN	NCBI
**3331**	DIRAS2	DIRA2_HUMAN	NCBI
**3332**	BTG4	BTG4_HUMAN	NCBI
**3333**	SPRYD7	SPRY7_HUMAN	NCBI
**3334**	SPIDR	SPIDR_HUMAN	NCBI
**3335**	LEMD1	LEMD1_HUMAN	NCBI
**3336**	LPCAT2	PCAT2_HUMAN	NCBI
**3337**	SPTY2D1	SPT2_HUMAN	NCBI
**3338**	ELMO3	ELMO3_HUMAN	NCBI
**3339**	C12orf75	OCC1_HUMAN	NCBI
**3340**	SHISA3	SHSA3_HUMAN	NCBI
**3341**	THSD4	THSD4_HUMAN	NCBI
**3342**	TTYH2	TTYH2_HUMAN	NCBI
**3343**	ASB6	ASB6_HUMAN	NCBI
**3344**	KRT72	K2C72_HUMAN	NCBI
**3345**	OLFM1	NOE1_HUMAN	NCBI
**3346**	ZNF267	ZN267_HUMAN	NCBI
**3347**	C2orf68	CB068_HUMAN	NCBI
**3348**	CHPF2	CHPF2_HUMAN	NCBI
**3349**	TMEM123	PORIM_HUMAN	NCBI
**3350**	TMEM25	TMM25_HUMAN	NCBI
**3351**	CITED4	CITE4_HUMAN	NCBI
**3352**	USP51	UBP51_HUMAN	NCBI
**3353**	ABHD11	ABHDB_HUMAN	NCBI
**3354**	UBXN2A	UBX2A_HUMAN	NCBI
**3355**	LARGE2	LARG2_HUMAN	NCBI
**3356**	UTP23	UTP23_HUMAN	NCBI
**3357**	NLE1	NLE1_HUMAN	NCBI
**3358**	TMEM238L	T238L_HUMAN	NCBI
**3359**	SLC12A9	S12A9_HUMAN	NCBI
**3360**	TMEM176B	T176B_HUMAN	NCBI

No.	Symbol	Uniprot Name	Database
**3361**	CDIP1	CDIP1_HUMAN	NCBI
**3362**	COL21A1	COLA1_HUMAN	NCBI
**3363**	SAMD4B	SMAG2_HUMAN	NCBI
**3364**	SAT2	SAT2_HUMAN	NCBI
**3365**	HECA	HDC_HUMAN	NCBI
**3366**	UBTD1	UBTD1_HUMAN	NCBI
**3367**	METTL15	MET15_HUMAN	NCBI
**3368**	LRFN4	LRFN4_HUMAN	NCBI
**3369**	CBLN4	CBLN4_HUMAN	NCBI
**3370**	FBXL20	FXL20_HUMAN	NCBI
**3371**	MTFR2	MTFR2_HUMAN	NCBI
**3372**	B3GNT6	B3GN6_HUMAN	NCBI
**3373**	P2RY10	P2Y10_HUMAN	NCBI
**3374**	EIF5AL1	IF5AL_HUMAN	NCBI
**3375**	MRPL35	RM35_HUMAN	NCBI
**3376**	CPNE7	CPNE7_HUMAN	NCBI
**3377**	ZCCHC4	ZCHC4_HUMAN	NCBI
**3378**	CNTN3	CNTN3_HUMAN	NCBI
**3379**	CDH22	CAD22_HUMAN	NCBI
**3380**	GPR176	GP176_HUMAN	NCBI
**3381**	DKKL1	DKKL1_HUMAN	NCBI
**3382**	GPR31	GPR31_HUMAN	NCBI
**3383**	CSMD3	CSMD3_HUMAN	NCBI
**3384**	TCFL5	TCFL5_HUMAN	NCBI
**3385**	NIT1	NIT1_HUMAN	NCBI
**3386**	ABTB1	ABTB1_HUMAN	NCBI
**3387**	RAPGEF5	RPGF5_HUMAN	NCBI
**3388**	PHACTR3	PHAR3_HUMAN	NCBI
**3389**	ZNF677	ZN677_HUMAN	NCBI
**3390**	WSB2	WSB2_HUMAN	NCBI
**3391**	PKD1L2	PK1L2_HUMAN	NCBI
**3392**	RNF183	RN183_HUMAN	NCBI
**3393**	PCDH18	PCD18_HUMAN	NCBI
**3394**	ZFP82	ZFP82_HUMAN	NCBI
**3395**	MID1IP1	M1IP1_HUMAN	NCBI
**3396**	RNF166	RN166_HUMAN	NCBI
**3397**	METTL9	METL9_HUMAN	NCBI
**3398**	PVRIG	PVRIG_HUMAN	NCBI
**3399**	ENKUR	ENKUR_HUMAN	NCBI
**3400**	LPCAT4	LPCT4_HUMAN	NCBI
**3401**	TSC22D2	T22D2_HUMAN	NCBI

No.	Symbol	Uniprot Name	Database
**3402**	ASB4	ASB4_HUMAN	NCBI
**3403**	CDH9	CADH9_HUMAN	NCBI
**3404**	CHAC2	CHAC2_HUMAN	NCBI
**3405**	ZFP90	ZFP90_HUMAN	NCBI
**3406**	NME6	NDK6_HUMAN	NCBI
**3407**	DSCC1	DCC1_HUMAN	NCBI
**3408**	PARM1	PARM1_HUMAN	NCBI
**3409**	CCDC12	CCD12_HUMAN	NCBI
**3410**	CHSY3	CHSS3_HUMAN	NCBI
**3411**	CSMD2	CSMD2_HUMAN	NCBI
**3412**	CAPRIN2	CAPR2_HUMAN	NCBI
**3413**	TMEM160	TM160_HUMAN	NCBI
**3414**	ZNF367	ZN367_HUMAN	NCBI
**3415**	GRID2IP	GRD2I_HUMAN	NCBI
**3416**	BCL2L15	B2L15_HUMAN	NCBI
**3417**	FBXO39	FBX39_HUMAN	NCBI
**3418**	FOXD2	FOXD2_HUMAN	NCBI
**3419**	CDK15	CDK15_HUMAN	NCBI
**3420**	ZNF169	ZN169_HUMAN	NCBI
**3421**	ZNF3	ZNF3_HUMAN	NCBI
**3422**	ZNF683	ZN683_HUMAN	NCBI
**3423**	GXYLT1	GXLT1_HUMAN	NCBI
**3424**	PPP1R3F	PPR3F_HUMAN	NCBI
**3425**	ARHGAP30	RHG30_HUMAN	NCBI
**3426**	GALNT5	GALT5_HUMAN	NCBI
**3427**	SUSD4	SUSD4_HUMAN	NCBI
**3428**	SLAMF9	SLAF9_HUMAN	NCBI
**3429**	PRKRIP1	PKRI1_HUMAN	NCBI
**3430**	SLC25A21	ODC_HUMAN	NCBI
**3431**	ATXN7L3B	A7L3B_HUMAN	NCBI
**3432**	PARP6	PARP6_HUMAN	NCBI
**3433**	MTMR7	MTMR7_HUMAN	NCBI
**3434**	FBXW10	FBW10_HUMAN	NCBI
**3435**	HCFC1R1	HPIP_HUMAN	NCBI
**3436**	AGPAT4	PLCD_HUMAN	NCBI
**3437**	SUSD6	SUSD6_HUMAN	NCBI
**3438**	BEST4	BEST4_HUMAN	NCBI
**3439**	AGBL4	CBPC6_HUMAN	NCBI
**3440**	FAM83E	FA83E_HUMAN	NCBI
**3441**	TCAF2	TCAF2_HUMAN	NCBI
**3442**	TSSK6	TSSK6_HUMAN	NCBI

No.	Symbol	Uniprot Name	Database
**3443**	ANKRD22	ANR22_HUMAN	NCBI
**3444**	TGIF2LX	TF2LX_HUMAN	NCBI
**3445**	ZNF334	ZN334_HUMAN	NCBI
**3446**	MS4A12	M4A12_HUMAN	NCBI
**3447**	GPR63	GPR63_HUMAN	NCBI
**3448**	FAM171B	F171B_HUMAN	NCBI
**3449**	C3orf85	CC085_HUMAN	NCBI
**3450**	C3orf85	A0A1B0GV21_HUMAN	NCBI
**3451**	C3orf85	A0A1B0GVL1_HUMAN	NCBI
**3452**	C3orf85	A0A1B0GVS5_HUMAN	NCBI
**3453**	LACTB2	LACB2_HUMAN	NCBI
**3454**	LY6G6D	LY66D_HUMAN	NCBI
**3455**	RTP4	RTP4_HUMAN	NCBI
**3456**	ZDHHC1	ZDHC1_HUMAN	NCBI
**3457**	FBXL8	FBXL8_HUMAN	NCBI
**3458**	METRN	METRN_HUMAN	NCBI
**3459**	COX19	COX19_HUMAN	NCBI
**3460**	TUBA4B	TBA4B_HUMAN	NCBI
**3461**	SP5	SP5_HUMAN	NCBI
**3462**	SPACA9	SACA9_HUMAN	NCBI
**3463**	CCDC43	CCD43_HUMAN	NCBI
**3464**	SPINK4	ISK4_HUMAN	NCBI
**3465**	ZNF37A	ZN37A_HUMAN	NCBI
**3466**	CELF3	CELF3_HUMAN	NCBI
**3467**	HMX3	HMX3_HUMAN	NCBI
**3468**	MEGF6	MEGF6_HUMAN	NCBI
**3469**	SHF	SHF_HUMAN	NCBI
**3470**	TRIM67	TRI67_HUMAN	NCBI
**3471**	WDR54	WDR54_HUMAN	NCBI
**3472**	CCNI2	CCNI2_HUMAN	NCBI
**3473**	TMIGD1	TMIG1_HUMAN	NCBI
**3474**	GALNT16	GLT16_HUMAN	NCBI
**3475**	DACT3	DACT3_HUMAN	NCBI
**3476**	SLC36A4	S36A4_HUMAN	NCBI
**3477**	NXPE4	NXPE4_HUMAN	NCBI
**3478**	ZNF70	ZNF70_HUMAN	NCBI
**3479**	LYRM2	LYRM2_HUMAN	NCBI
**3480**	KBTBD11	KBTBB_HUMAN	NCBI
**3481**	CWH43	PG2IP_HUMAN	NCBI
**3482**	PCDHGA7	PCDG7_HUMAN	NCBI
**3483**	CCDC154	CC154_HUMAN	NCBI

No.	Symbol	Uniprot Name	Database
**3484**	PLEKHO2	PKHO2_HUMAN	NCBI
**3485**	NRIP2	NRIP2_HUMAN	NCBI
**3486**	VSTM2A	VTM2A_HUMAN	NCBI
**3487**	MFSD14B	MF14B_HUMAN	NCBI
**3488**	IZUMO2	IZUM2_HUMAN	NCBI
**3489**	RNF148	RN148_HUMAN	NCBI
**3490**	LYPD8	LYPD8_HUMAN	NCBI
**3491**	HAGHL	HAGHL_HUMAN	NCBI
**3492**	MFAP3L	MFA3L_HUMAN	NCBI
**3493**	PACRGL	PACRL_HUMAN	NCBI
**3494**	BARHL2	BARH2_HUMAN	NCBI
**3495**	NBPF4	NBPF4_HUMAN	NCBI
**3496**	ZNF839	ZN839_HUMAN	NCBI
**3497**	C8orf48	CH048_HUMAN	NCBI
**3498**	CCDC107	CC107_HUMAN	NCBI
**3499**	CT47A11	CT47A_HUMAN	NCBI
**3500**	RERGL	RERGL_HUMAN	NCBI
**3501**	ZNF880	ZN880_HUMAN	NCBI
**3502**	AMER3	AMER3_HUMAN	NCBI
**3503**	ZNF700	ZN700_HUMAN	NCBI
**3504**	RAD51C	RAD51C_HUMAN	GeneCards
**3505**	CTNNA1	CTNNA1_HUMAN	GeneCards
**3506**	FH	FH_HUMAN	GeneCards
**3507**	SDHC	SDHC_HUMAN	GeneCards
**3508**	CDC73	CDC73_HUMAN	GeneCards
**3509**	SUFU	SUFU_HUMAN	GeneCards
**3510**	TMEM127	TMEM127_HUMAN	GeneCards
**3511**	SDHAF2	SDHAF2_HUMAN	GeneCards
**3512**	AIP	AIP_HUMAN	GeneCards
**3513**	FANCA	FANCA_HUMAN	GeneCards
**3514**	ABRAXAS1	ABRAXAS1_HUMAN	GeneCards
**3515**	RECQL	RECQL_HUMAN	GeneCards
**3516**	CDK2	CDK2_HUMAN	GeneCards
**3517**	CYP17A1	CYP17A1_HUMAN	GeneCards
**3518**	FANCL	FANCL_HUMAN	GeneCards
**3519**	MT-CO1	MT-CO1_HUMAN	GeneCards
**3520**	ACVR1B	ACVR1B_HUMAN	GeneCards
**3521**	ERCC3	ERCC3_HUMAN	GeneCards
**3522**	MSR1	MSR1_HUMAN	GeneCards
**3523**	FBXO11	FBXO11_HUMAN	GeneCards
**3524**	DMD	DMD_HUMAN	GeneCards

No.	Symbol	Uniprot Name	Database
**3525**	MT-CYB	MT-CYB_HUMAN	GeneCards
**3526**	MIR7-3HG	MIR7-3HG_HUMAN	GeneCards
**3527**	WWOX	WWOX_HUMAN	GeneCards
**3528**	EIF3E	EIF3E_HUMAN	GeneCards
**3529**	POLK	POLK_HUMAN	GeneCards
**3530**	TSG101	TSG101_HUMAN	GeneCards
**3531**	NSD1	NSD1_HUMAN	GeneCards
**3532**	CLPTM1L	CLPTM1L_HUMAN	GeneCards
**3533**	LMNA	LMNA_HUMAN	GeneCards
**3534**	MXI1	MXI1_HUMAN	GeneCards
**3535**	CKS1B	CKS1B_HUMAN	GeneCards
**3536**	TPP1	TPP1_HUMAN	GeneCards
**3537**	TSHR	TSHR_HUMAN	GeneCards
**3538**	LDLR	LDLR_HUMAN	GeneCards
**3539**	SRD5A2	SRD5A2_HUMAN	GeneCards
**3540**	EXT1	EXT1_HUMAN	GeneCards
**3541**	DELEC1	DELEC1_HUMAN	GeneCards
**3542**	TRRAP	TRRAP_HUMAN	GeneCards
**3543**	GNE	GNE_HUMAN	GeneCards
**3544**	ZFHX3	ZFHX3_HUMAN	GeneCards
**3545**	CPT1A	CPT1A_HUMAN	GeneCards
**3546**	PTPRD	PTPRD_HUMAN	GeneCards
**3547**	RB1CC1	RB1CC1_HUMAN	GeneCards
**3548**	FGF10	FGF10_HUMAN	GeneCards
**3549**	CYP2D6	CYP2D6_HUMAN	GeneCards
**3550**	SF3B2	SF3B2_HUMAN	GeneCards
**3551**	KRT8	KRT8_HUMAN	GeneCards
**3552**	BLTP2	BLTP2_HUMAN	GeneCards
**3553**	ZNF276	ZNF276_HUMAN	GeneCards
**3554**	PIK3C3	PIK3C3_HUMAN	GeneCards
**3555**	EHBP1	EHBP1_HUMAN	GeneCards
**3556**	STAG2	STAG2_HUMAN	GeneCards
**3557**	RPS27A	RPS27A_HUMAN	GeneCards
**3558**	HERC2	HERC2_HUMAN	GeneCards
**3559**	TBX3	TBX3_HUMAN	GeneCards
**3560**	THADA	THADA_HUMAN	GeneCards
**3561**	HLA-DRB1	HLA-DRB1_HUMAN	GeneCards
**3562**	CYP2C19	CYP2C19_HUMAN	GeneCards
**3563**	IL2RB	IL2RB_HUMAN	GeneCards
**3564**	LIG1	LIG1_HUMAN	GeneCards
**3565**	BTRC	BTRC_HUMAN	GeneCards

No.	Symbol	Uniprot Name	Database
**3566**	CCNA1	CCNA1_HUMAN	GeneCards
**3567**	KRT5	KRT5_HUMAN	GeneCards
**3568**	TG	TG_HUMAN	GeneCards
**3569**	LIPE	LIPE_HUMAN	GeneCards
**3570**	H3-3A	H3-3A_HUMAN	GeneCards
**3571**	EBAG9	EBAG9_HUMAN	GeneCards
**3572**	AREL1	AREL1_HUMAN	GeneCards
**3573**	PNLIPRP1	PNLIPRP1_HUMAN	GeneCards
**3574**	TUBB	TUBB_HUMAN	GeneCards
**3575**	SMC3	SMC3_HUMAN	GeneCards
**3576**	ING4	ING4_HUMAN	GeneCards
**3577**	EPOR	EPOR_HUMAN	GeneCards
**3578**	HSPA1A	HSPA1A_HUMAN	GeneCards
**3579**	FGF1	FGF1_HUMAN	GeneCards
**3580**	CPT1B	CPT1B_HUMAN	GeneCards
**3581**	TRIP12	TRIP12_HUMAN	GeneCards
**3582**	KIR3DL1	KIR3DL1_HUMAN	GeneCards
**3583**	ADA	ADA_HUMAN	GeneCards
**3584**	INTS3	INTS3_HUMAN	GeneCards
**3585**	CP	CP_HUMAN	GeneCards
**3586**	CD59	CD59_HUMAN	GeneCards
**3587**	PPIG	PPIG_HUMAN	GeneCards
**3588**	DDX41	DDX41_HUMAN	GeneCards
**3589**	HPRT1	HPRT1_HUMAN	GeneCards
**3590**	ALPP	ALPP_HUMAN	GeneCards
**3591**	DHX29	DHX29_HUMAN	GeneCards
**3592**	PRPF8	PRPF8_HUMAN	GeneCards
**3593**	ARAP3	ARAP3_HUMAN	GeneCards
**3594**	HLA-DQA1	HLA-DQA1_HUMAN	GeneCards
**3595**	GNGT1	GNGT1_HUMAN	GeneCards
**3596**	RAB43	RAB43_HUMAN	GeneCards
**3597**	SBNO1	SBNO1_HUMAN	GeneCards
**3598**	RANBP2	RANBP2_HUMAN	GeneCards
**3599**	CXADR	CXADR_HUMAN	GeneCards
**3600**	SHCBP1L	SHCBP1L_HUMAN	GeneCards
**3601**	CDKN2AIP	CDKN2AIP_HUMAN	GeneCards
**3602**	RPL10L	RPL10L_HUMAN	GeneCards
**3603**	RARS1	RARS1_HUMAN	GeneCards
**3604**	CASP6	CASP6_HUMAN	GeneCards
**3605**	LINC01554	LINC01554_HUMAN	GeneCards
**3606**	RACK1	RACK1_HUMAN	GeneCards

No.	Symbol	Uniprot Name	Database
**3607**	PDHA1	PDHA1_HUMAN	GeneCards
**3608**	S100A6	S100A6_HUMAN	GeneCards
**3609**	HSPH1	HSPH1_HUMAN	GeneCards
**3610**	HUWE1	HUWE1_HUMAN	GeneCards
**3611**	VCL	VCL_HUMAN	GeneCards
**3612**	F2	F2_HUMAN	GeneCards
**3613**	SLC29A1	SLC29A1_HUMAN	GeneCards
**3614**	RALBP1	RALBP1_HUMAN	GeneCards
**3615**	KRT1	KRT1_HUMAN	GeneCards
**3616**	AKAP1	AKAP1_HUMAN	GeneCards
**3617**	PTPN21	PTPN21_HUMAN	GeneCards
**3618**	TSHZ3	TSHZ3_HUMAN	GeneCards
**3619**	ITGAM	ITGAM_HUMAN	GeneCards
**3620**	RHOC	RHOC_HUMAN	GeneCards
**3621**	ATIC	ATIC_HUMAN	GeneCards
**3622**	SKP1	SKP1_HUMAN	GeneCards
**3623**	PRKAB1	PRKAB1_HUMAN	GeneCards
**3624**	PRDX5	PRDX5_HUMAN	GeneCards
**3625**	TCIM	TCIM_HUMAN	GeneCards
**3626**	AIMP2	AIMP2_HUMAN	GeneCards
**3627**	SLC12A3	SLC12A3_HUMAN	GeneCards
**3628**	PTP4A1	PTP4A1_HUMAN	GeneCards
**3629**	ADAT1	ADAT1_HUMAN	GeneCards
**3630**	RASA2	RASA2_HUMAN	GeneCards
**3631**	MMUT	MMUT_HUMAN	GeneCards
**3632**	PPP6R2	PPP6R2_HUMAN	GeneCards
**3633**	CALB2	CALB2_HUMAN	GeneCards
**3634**	SLC5A5	SLC5A5_HUMAN	GeneCards
**3635**	DNAH8	DNAH8_HUMAN	GeneCards
**3636**	FOXP2	FOXP2_HUMAN	GeneCards
**3637**	WNK2	WNK2_HUMAN	GeneCards
**3638**	PLCB4	PLCB4_HUMAN	GeneCards
**3639**	NSUN2	NSUN2_HUMAN	GeneCards
**3640**	NAPB	NAPB_HUMAN	GeneCards
**3641**	RPS19	RPS19_HUMAN	GeneCards
**3642**	POLH	POLH_HUMAN	GeneCards
**3643**	RPL5	RPL5_HUMAN	GeneCards
**3644**	IL1R1	IL1R1_HUMAN	GeneCards
**3645**	CCNE2	CCNE2_HUMAN	GeneCards
**3646**	CPS1	CPS1_HUMAN	GeneCards
**3647**	AKR1C1	AKR1C1_HUMAN	GeneCards

No.	Symbol	Uniprot Name	Database
**3648**	NOP10	NOP10_HUMAN	GeneCards
**3649**	TF	TF_HUMAN	GeneCards
**3650**	MAPT	MAPT_HUMAN	GeneCards
**3651**	MDH2	MDH2_HUMAN	GeneCards
**3652**	MYBPC3	MYBPC3_HUMAN	GeneCards
**3653**	AGK	AGK_HUMAN	GeneCards
**3654**	NAA10	NAA10_HUMAN	GeneCards
**3655**	KRT14	KRT14_HUMAN	GeneCards
**3656**	ACVR1	ACVR1_HUMAN	GeneCards
**3657**	PPM1L	PPM1L_HUMAN	GeneCards
**3658**	PSMB8	PSMB8_HUMAN	GeneCards
**3659**	GNB1	GNB1_HUMAN	GeneCards
**3660**	LMNB1	LMNB1_HUMAN	GeneCards
**3661**	MCM4	MCM4_HUMAN	GeneCards
**3662**	RPL11	RPL11_HUMAN	GeneCards
**3663**	UBE2T	UBE2T_HUMAN	GeneCards
**3664**	IKBKG	IKBKG_HUMAN	GeneCards
**3665**	MRTFA	MRTFA_HUMAN	GeneCards
**3666**	SLMAP	SLMAP_HUMAN	GeneCards
**3667**	CCNB2	CCNB2_HUMAN	GeneCards
**3668**	STARD13	STARD13_HUMAN	GeneCards
**3669**	SPDEF	SPDEF_HUMAN	GeneCards
**3670**	BRMS1L	BRMS1L_HUMAN	GeneCards
**3671**	LRRFIP2	LRRFIP2_HUMAN	GeneCards
**3672**	ABL2	ABL2_HUMAN	GeneCards
**3673**	C1QBP	C1QBP_HUMAN	GeneCards
**3674**	CBFA2T3	CBFA2T3_HUMAN	GeneCards
**3675**	COL18A1	COL18A1_HUMAN	GeneCards
**3676**	CCNL2	CCNL2_HUMAN	GeneCards
**3677**	CEP55	CEP55_HUMAN	GeneCards
**3678**	CD69	CD69_HUMAN	GeneCards
**3679**	CLCA2	CLCA2_HUMAN	GeneCards
**3680**	ENTPD1	ENTPD1_HUMAN	GeneCards
**3681**	LHCGR	LHCGR_HUMAN	GeneCards
**3682**	GAB1	GAB1_HUMAN	GeneCards
**3683**	NOX1	NOX1_HUMAN	GeneCards
**3684**	CCNH	CCNH_HUMAN	GeneCards
**3685**	CCNB3	CCNB3_HUMAN	GeneCards
**3686**	SLC16A3	SLC16A3_HUMAN	GeneCards
**3687**	GPR161	GPR161_HUMAN	GeneCards
**3688**	TALDO1	TALDO1_HUMAN	GeneCards

No.	Symbol	Uniprot Name	Database
**3689**	WNT11	WNT11_HUMAN	GeneCards
**3690**	CIC	CIC_HUMAN	GeneCards
**3691**	TPI1	TPI1_HUMAN	GeneCards
**3692**	SI	SI_HUMAN	GeneCards
**3693**	RPL26	RPL26_HUMAN	GeneCards
**3694**	BCAS2	BCAS2_HUMAN	GeneCards
**3695**	DNAJB1	DNAJB1_HUMAN	GeneCards
**3696**	RASSF5	RASSF5_HUMAN	GeneCards
**3697**	METAP2	METAP2_HUMAN	GeneCards
**3698**	SS18	SS18_HUMAN	GeneCards
**3699**	CBR4	CBR4_HUMAN	GeneCards
**3700**	WNT9A	WNT9A_HUMAN	GeneCards
**3701**	MAPRE2	MAPRE2_HUMAN	GeneCards
**3702**	NHP2	NHP2_HUMAN	GeneCards
**3703**	RPL6	RPL6_HUMAN	GeneCards
**3704**	PGD	PGD_HUMAN	GeneCards
**3705**	EEF2	EEF2_HUMAN	GeneCards
**3706**	HNRNPC	HNRNPC_HUMAN	GeneCards
**3707**	LIMS1	LIMS1_HUMAN	GeneCards
**3708**	PHF6	PHF6_HUMAN	GeneCards
**3709**	PIAS1	PIAS1_HUMAN	GeneCards
**3710**	RNASE1	RNASE1_HUMAN	GeneCards
**3711**	PPAT	PPAT_HUMAN	GeneCards
**3712**	TUBG1	TUBG1_HUMAN	GeneCards
**3713**	BMPR1B	BMPR1B_HUMAN	GeneCards
**3714**	NMT1	NMT1_HUMAN	GeneCards
**3715**	CYP2B6	CYP2B6_HUMAN	GeneCards
**3716**	RBL2	RBL2_HUMAN	GeneCards
**3717**	CDA	CDA_HUMAN	GeneCards
**3718**	RPS10	RPS10_HUMAN	GeneCards
**3719**	TNK2	TNK2_HUMAN	GeneCards
**3720**	CDH11	CDH11_HUMAN	GeneCards
**3721**	RPL15	RPL15_HUMAN	GeneCards
**3722**	CD81	CD81_HUMAN	GeneCards
**3723**	ERGIC3	ERGIC3_HUMAN	GeneCards
**3724**	RPL29	RPL29_HUMAN	GeneCards
**3725**	SRSF1	SRSF1_HUMAN	GeneCards
**3726**	LSP1	LSP1_HUMAN	GeneCards
**3727**	RPL10	RPL10_HUMAN	GeneCards
**3728**	PFKP	PFKP_HUMAN	GeneCards
**3729**	PA2G4	PA2G4_HUMAN	GeneCards

No.	Symbol	Uniprot Name	Database
**3730**	SPON2	SPON2_HUMAN	GeneCards
**3731**	CDC37	CDC37_HUMAN	GeneCards
**3732**	HLA-DPB1	HLA-DPB1_HUMAN	GeneCards
**3733**	CAV2	CAV2_HUMAN	GeneCards
**3734**	RPS29	RPS29_HUMAN	GeneCards
**3735**	PICALM	PICALM_HUMAN	GeneCards
**3736**	ALOX15B	ALOX15B_HUMAN	GeneCards
**3737**	RPL22	RPL22_HUMAN	GeneCards
**3738**	SRF	SRF_HUMAN	GeneCards
**3739**	STEAP2	STEAP2_HUMAN	GeneCards
**3740**	CYP2C8	CYP2C8_HUMAN	GeneCards
**3741**	TBCE	TBCE_HUMAN	GeneCards
**3742**	GMNN	GMNN_HUMAN	GeneCards
**3743**	TRAF4	TRAF4_HUMAN	GeneCards
**3744**	AKT1S1	AKT1S1_HUMAN	GeneCards
**3745**	PIK3R5	PIK3R5_HUMAN	GeneCards
**3746**	DLL3	DLL3_HUMAN	GeneCards
**3747**	FRS2	FRS2_HUMAN	GeneCards
**3748**	PAX3	PAX3_HUMAN	GeneCards
**3749**	RPA1	RPA1_HUMAN	GeneCards
**3750**	DNAJC21	DNAJC21_HUMAN	GeneCards
**3751**	RPL35A	RPL35A_HUMAN	GeneCards
**3752**	SUMF1	SUMF1_HUMAN	GeneCards
**3753**	NCAPG	NCAPG_HUMAN	GeneCards
**3754**	MCPH1	MCPH1_HUMAN	GeneCards
**3755**	FZD1	FZD1_HUMAN	GeneCards
**3756**	GADD45G	GADD45G_HUMAN	GeneCards
**3757**	MYO7A	MYO7A_HUMAN	GeneCards
**3758**	TNFRSF17	TNFRSF17_HUMAN	GeneCards
**3759**	USF2	USF2_HUMAN	GeneCards
**3760**	PLEK	PLEK_HUMAN	GeneCards
**3761**	RIOX1	RIOX1_HUMAN	GeneCards
**3762**	FDXR	FDXR_HUMAN	GeneCards
**3763**	GNL3	GNL3_HUMAN	GeneCards
**3764**	UBE2I	UBE2I_HUMAN	GeneCards
**3765**	CDT1	CDT1_HUMAN	GeneCards
**3766**	RFC4	RFC4_HUMAN	GeneCards
**3767**	HDAC8	HDAC8_HUMAN	GeneCards
**3768**	RPS26	RPS26_HUMAN	GeneCards
**3769**	RPL10A	RPL10A_HUMAN	GeneCards
**3770**	ARHGAP45	ARHGAP45_HUMAN	GeneCards

No.	Symbol	Uniprot Name	Database
**3771**	ERN1	ERN1_HUMAN	GeneCards
**3772**	TAF1	TAF1_HUMAN	GeneCards
**3773**	ANAPC1	ANAPC1_HUMAN	GeneCards
**3774**	TRIM37	TRIM37_HUMAN	GeneCards
**3775**	CA12	CA12_HUMAN	GeneCards
**3776**	RAB25	RAB25_HUMAN	GeneCards
**3777**	PSMC4	PSMC4_HUMAN	GeneCards
**3778**	FDPS	FDPS_HUMAN	GeneCards
**3779**	GLO1	GLO1_HUMAN	GeneCards
**3780**	CYP11A1	CYP11A1_HUMAN	GeneCards
**3781**	SEMA6A	SEMA6A_HUMAN	GeneCards
**3782**	CHIC2	CHIC2_HUMAN	GeneCards
**3783**	CS	CS_HUMAN	GeneCards
**3784**	RPL19	RPL19_HUMAN	GeneCards
**3785**	MEAF6	MEAF6_HUMAN	GeneCards
**3786**	TP53BP2	TP53BP2_HUMAN	GeneCards
**3787**	NOP2	NOP2_HUMAN	GeneCards
**3788**	NOD1	NOD1_HUMAN	GeneCards
**3789**	MRPS11	MRPS11_HUMAN	GeneCards
**3790**	MX1	MX1_HUMAN	GeneCards
**3791**	VWA2	VWA2_HUMAN	GeneCards
**3792**	ASAH1	ASAH1_HUMAN	GeneCards
**3793**	PSMA1	PSMA1_HUMAN	GeneCards
**3794**	IGKC	IGKC_HUMAN	GeneCards
**3795**	CLTC	CLTC_HUMAN	GeneCards
**3796**	CCT4	CCT4_HUMAN	GeneCards
**3797**	PAG1	PAG1_HUMAN	GeneCards
**3798**	RPS14	RPS14_HUMAN	GeneCards
**3799**	PDS5B	PDS5B_HUMAN	GeneCards
**3800**	AKR1A1	AKR1A1_HUMAN	GeneCards
**3801**	ELOC	ELOC_HUMAN	GeneCards
**3802**	RPS9	RPS9_HUMAN	GeneCards
**3803**	RNF7	RNF7_HUMAN	GeneCards
**3804**	TH	TH_HUMAN	GeneCards
**3805**	THBD	THBD_HUMAN	GeneCards
**3806**	PPP1CB	PPP1CB_HUMAN	GeneCards
**3807**	RPL9	RPL9_HUMAN	GeneCards
**3808**	ZNHIT6	ZNHIT6_HUMAN	GeneCards
**3809**	SGMS1	SGMS1_HUMAN	GeneCards
**3810**	PNP	PNP_HUMAN	GeneCards
**3811**	MACROD1	MACROD1_HUMAN	GeneCards

No.	Symbol	Uniprot Name	Database
**3812**	TPO	TPO_HUMAN	GeneCards
**3813**	RPS15	RPS15_HUMAN	GeneCards
**3814**	CAPN2	CAPN2_HUMAN	GeneCards
**3815**	NEMF	NEMF_HUMAN	GeneCards
**3816**	SNCA	SNCA_HUMAN	GeneCards
**3817**	TMSB10	TMSB10_HUMAN	GeneCards
**3818**	PDLIM5	PDLIM5_HUMAN	GeneCards
**3819**	PGRMC1	PGRMC1_HUMAN	GeneCards
**3820**	PRMT7	PRMT7_HUMAN	GeneCards
**3821**	RPL35	RPL35_HUMAN	GeneCards
**3822**	RANBP1	RANBP1_HUMAN	GeneCards
**3823**	RPS17	RPS17_HUMAN	GeneCards
**3824**	RPS12	RPS12_HUMAN	GeneCards
**3825**	LPCAT1	LPCAT1_HUMAN	GeneCards
**3826**	NUDC	NUDC_HUMAN	GeneCards
**3827**	SAFB2	SAFB2_HUMAN	GeneCards
**3828**	KDM2B	KDM2B_HUMAN	GeneCards
**3829**	PCDH11X	PCDH11X_HUMAN	GeneCards
**3830**	PIK3C2A	PIK3C2A_HUMAN	GeneCards
**3831**	PSEN1	PSEN1_HUMAN	GeneCards
**3832**	GOLM2	GOLM2_HUMAN	GeneCards
**3833**	TACC2	TACC2_HUMAN	GeneCards
**3834**	RPL27A	RPL27A_HUMAN	GeneCards
**3835**	STRBP	STRBP_HUMAN	GeneCards
**3836**	SIAH1	SIAH1_HUMAN	GeneCards
**3837**	PRPSAP1	PRPSAP1_HUMAN	GeneCards
**3838**	MAP3K2	MAP3K2_HUMAN	GeneCards
**3839**	ABCB7	ABCB7_HUMAN	GeneCards
**3840**	ARHGDIB	ARHGDIB_HUMAN	GeneCards
**3841**	DNM1L	DNM1L_HUMAN	GeneCards
**3842**	MACROH2A1	MACROH2A1_HUMAN	GeneCards
**3843**	ANKRD17	ANKRD17_HUMAN	GeneCards
**3844**	SART1	SART1_HUMAN	GeneCards
**3845**	UBE2D1	UBE2D1_HUMAN	GeneCards
**3846**	HHIP	HHIP_HUMAN	GeneCards
**3847**	HBB	HBB_HUMAN	GeneCards
**3848**	AFF1	AFF1_HUMAN	GeneCards
**3849**	RING1	RING1_HUMAN	GeneCards
**3850**	NACC1	NACC1_HUMAN	GeneCards
**3851**	PRPF19	PRPF19_HUMAN	GeneCards
**3852**	ACP5	ACP5_HUMAN	GeneCards

No.	Symbol	Uniprot Name	Database
**3853**	ARID4A	ARID4A_HUMAN	GeneCards
**3854**	CDC42BPB	CDC42BPB_HUMAN	GeneCards
**3855**	PLSCR4	PLSCR4_HUMAN	GeneCards
**3856**	IL2RG	IL2RG_HUMAN	GeneCards
**3857**	DGCR8	DGCR8_HUMAN	GeneCards
**3858**	CALD1	CALD1_HUMAN	GeneCards
**3859**	EGLN2	EGLN2_HUMAN	GeneCards
**3860**	RAD9A	RAD9A_HUMAN	GeneCards
**3861**	RNF146	RNF146_HUMAN	GeneCards
**3862**	PRG2	PRG2_HUMAN	GeneCards
**3863**	SLC17A5	SLC17A5_HUMAN	GeneCards
**3864**	RPS11	RPS11_HUMAN	GeneCards
**3865**	CBLL1	CBLL1_HUMAN	GeneCards
**3866**	FAAH	FAAH_HUMAN	GeneCards
**3867**	POLR2L	POLR2L_HUMAN	GeneCards
**3868**	CETN3	CETN3_HUMAN	GeneCards
**3869**	RPL17	RPL17_HUMAN	GeneCards
**3870**	RPL34	RPL34_HUMAN	GeneCards
**3871**	SLC4A7	SLC4A7_HUMAN	GeneCards
**3872**	CFL2	CFL2_HUMAN	GeneCards
**3873**	PAPPA	PAPPA_HUMAN	GeneCards
**3874**	ZDHHC2	ZDHHC2_HUMAN	GeneCards
**3875**	RPL13A	RPL13A_HUMAN	GeneCards
**3876**	WDR77	WDR77_HUMAN	GeneCards
**3877**	TBC1D7	TBC1D7_HUMAN	GeneCards
**3878**	CEPT1	CEPT1_HUMAN	GeneCards
**3879**	SIPA1L1	SIPA1L1_HUMAN	GeneCards
**3880**	BNIP3L	BNIP3L_HUMAN	GeneCards
**3881**	MUS81	MUS81_HUMAN	GeneCards
**3882**	POLI	POLI_HUMAN	GeneCards
**3883**	POLR1H	POLR1H_HUMAN	GeneCards
**3884**	OCIAD1	OCIAD1_HUMAN	GeneCards
**3885**	FIP1L1	FIP1L1_HUMAN	GeneCards
**3886**	KAT6B	KAT6B_HUMAN	GeneCards
**3887**	AIFM2	AIFM2_HUMAN	GeneCards
**3888**	USH2A	USH2A_HUMAN	GeneCards
**3889**	RSF1	RSF1_HUMAN	GeneCards
**3890**	TUT4	TUT4_HUMAN	GeneCards
**3891**	SLC7A6	SLC7A6_HUMAN	GeneCards
**3892**	PISD	PISD_HUMAN	GeneCards
**3893**	CAPN9	CAPN9_HUMAN	GeneCards

No.	Symbol	Uniprot Name	Database
**3894**	GTF2B	GTF2B_HUMAN	GeneCards
**3895**	RCN1	RCN1_HUMAN	GeneCards
**3896**	ASS1	ASS1_HUMAN	GeneCards
**3897**	NUP93	NUP93_HUMAN	GeneCards
**3898**	RFC3	RFC3_HUMAN	GeneCards
**3899**	CHD5	CHD5_HUMAN	GeneCards
**3900**	UPF3B	UPF3B_HUMAN	GeneCards
**3901**	UBE2V1	UBE2V1_HUMAN	GeneCards
**3902**	RPS5	RPS5_HUMAN	GeneCards
**3903**	MAP2	MAP2_HUMAN	GeneCards
**3904**	PSMC5	PSMC5_HUMAN	GeneCards
**3905**	LMO7	LMO7_HUMAN	GeneCards
**3906**	CENPE	CENPE_HUMAN	GeneCards
**3907**	AQP1	AQP1_HUMAN	GeneCards
**3908**	SMAD5-AS1	SMAD5-AS1_HUMAN	GeneCards
**3909**	DUS2	DUS2_HUMAN	GeneCards
**3910**	PPP6C	PPP6C_HUMAN	GeneCards
**3911**	BLZF1	BLZF1_HUMAN	GeneCards
**3912**	DDX47	DDX47_HUMAN	GeneCards
**3913**	RBMX	RBMX_HUMAN	GeneCards
**3914**	RPA2	RPA2_HUMAN	GeneCards
**3915**	DOCK1	DOCK1_HUMAN	GeneCards
**3916**	RPL36	RPL36_HUMAN	GeneCards
**3917**	PYROXD1	PYROXD1_HUMAN	GeneCards
**3918**	BCDIN3D	BCDIN3D_HUMAN	GeneCards
**3919**	PCNX2	PCNX2_HUMAN	GeneCards
**3920**	DCTN4	DCTN4_HUMAN	GeneCards
**3921**	FLNC	FLNC_HUMAN	GeneCards
**3922**	SLC6A3	SLC6A3_HUMAN	GeneCards
**3923**	CLSPN	CLSPN_HUMAN	GeneCards
**3924**	C5	C5_HUMAN	GeneCards
**3925**	HUS1	HUS1_HUMAN	GeneCards
**3926**	EIF3M	EIF3M_HUMAN	GeneCards
**3927**	FAM83D	FAM83D_HUMAN	GeneCards
**3928**	RPL8	RPL8_HUMAN	GeneCards
**3929**	COPS2	COPS2_HUMAN	GeneCards
**3930**	IRX2-DT	IRX2-DT_HUMAN	GeneCards
**3931**	DUSP19	DUSP19_HUMAN	GeneCards
**3932**	URI1	URI1_HUMAN	GeneCards
**3933**	GGCT	GGCT_HUMAN	GeneCards
**3934**	RPL4	RPL4_HUMAN	GeneCards

No.	Symbol	Uniprot Name	Database
**3935**	KPTN	KPTN_HUMAN	GeneCards
**3936**	FAH	FAH_HUMAN	GeneCards
**3937**	BABAM1	BABAM1_HUMAN	GeneCards
**3938**	ESS2	ESS2_HUMAN	GeneCards
**3939**	LDHC	LDHC_HUMAN	GeneCards
**3940**	ELOB	ELOB_HUMAN	GeneCards
**3941**	SRP19	SRP19_HUMAN	GeneCards
**3942**	PDLIM7	PDLIM7_HUMAN	GeneCards
**3943**	SRCAP	SRCAP_HUMAN	GeneCards
**3944**	RUVBL2	RUVBL2_HUMAN	GeneCards
**3945**	KIF23	KIF23_HUMAN	GeneCards
**3946**	CLPB	CLPB_HUMAN	GeneCards
**3947**	MAPKAPK3	MAPKAPK3_HUMAN	GeneCards
**3948**	RNASET2	RNASET2_HUMAN	GeneCards
**3949**	GBA1	GBA1_HUMAN	GeneCards
**3950**	APH1A	APH1A_HUMAN	GeneCards
**3951**	PLEC	PLEC_HUMAN	GeneCards
**3952**	CCDC25	CCDC25_HUMAN	GeneCards
**3953**	USF1	USF1_HUMAN	GeneCards
**3954**	TATDN1	TATDN1_HUMAN	GeneCards
**3955**	ARFGAP1	ARFGAP1_HUMAN	GeneCards
**3956**	ALPL	ALPL_HUMAN	GeneCards
**3957**	SIAH2	SIAH2_HUMAN	GeneCards
**3958**	TAT	TAT_HUMAN	GeneCards
**3959**	RPL7	RPL7_HUMAN	GeneCards
**3960**	CBX2	CBX2_HUMAN	GeneCards
**3961**	NEU1	NEU1_HUMAN	GeneCards
**3962**	CRBN	CRBN_HUMAN	GeneCards
**3963**	SAGE1	SAGE1_HUMAN	GeneCards
**3964**	SPTLC1	SPTLC1_HUMAN	GeneCards
**3965**	WWP1	WWP1_HUMAN	GeneCards
**3966**	KRT13	KRT13_HUMAN	GeneCards
**3967**	GLP1R	GLP1R_HUMAN	GeneCards
**3968**	UGDH	UGDH_HUMAN	GeneCards
**3969**	LPIN1	LPIN1_HUMAN	GeneCards
**3970**	BHMT	BHMT_HUMAN	GeneCards
**3971**	RHD	RHD_HUMAN	GeneCards
**3972**	GALNS	GALNS_HUMAN	GeneCards
**3973**	ATXN2	ATXN2_HUMAN	GeneCards
**3974**	PSMD14	PSMD14_HUMAN	GeneCards
**3975**	TP53INP1	TP53INP1_HUMAN	GeneCards

No.	Symbol	Uniprot Name	Database
**3976**	RPL13	RPL13_HUMAN	GeneCards
**3977**	MFN1	MFN1_HUMAN	GeneCards
**3978**	PLA2G12B	PLA2G12B_HUMAN	GeneCards
**3979**	LUC7L2	LUC7L2_HUMAN	GeneCards
**3980**	NECTIN3	NECTIN3_HUMAN	GeneCards
**3981**	PDE5A	PDE5A_HUMAN	GeneCards
**3982**	CAPZB	CAPZB_HUMAN	GeneCards
**3983**	RPS2	RPS2_HUMAN	GeneCards
**3984**	SERBP1	SERBP1_HUMAN	GeneCards
**3985**	PIGS	PIGS_HUMAN	GeneCards
**3986**	CLDN11	CLDN11_HUMAN	GeneCards
**3987**	CCKAR	CCKAR_HUMAN	GeneCards
**3988**	PLK3	PLK3_HUMAN	GeneCards
**3989**	ZNF652	ZNF652_HUMAN	GeneCards
**3990**	PSMD5	PSMD5_HUMAN	GeneCards
**3991**	RPS8	RPS8_HUMAN	GeneCards
**3992**	CCR8	CCR8_HUMAN	GeneCards
**3993**	BDH1	BDH1_HUMAN	GeneCards
**3994**	JUNB	JUNB_HUMAN	GeneCards
**3995**	PYY	PYY_HUMAN	GeneCards
**3996**	RPL7A	RPL7A_HUMAN	GeneCards
**3997**	REN	REN_HUMAN	GeneCards
**3998**	TARDBP	TARDBP_HUMAN	GeneCards
**3999**	SNW1	SNW1_HUMAN	GeneCards
**4000**	UNC5B	UNC5B_HUMAN	GeneCards
**4001**	ADSL	ADSL_HUMAN	GeneCards
**4002**	PUS1	PUS1_HUMAN	GeneCards
**4003**	DHX36	DHX36_HUMAN	GeneCards
**4004**	DSE	DSE_HUMAN	GeneCards
**4005**	CRH	CRH_HUMAN	GeneCards
**4006**	SERPINE2	SERPINE2_HUMAN	GeneCards
**4007**	SDF4	SDF4_HUMAN	GeneCards
**4008**	ACVR2B	ACVR2B_HUMAN	GeneCards
**4009**	NOS1	NOS1_HUMAN	GeneCards
**4010**	GCG	GCG_HUMAN	GeneCards
**4011**	BRI3BP	BRI3BP_HUMAN	GeneCards
**4012**	PPP1R12A	PPP1R12A_HUMAN	GeneCards
**4013**	LMO4	LMO4_HUMAN	GeneCards
**4014**	RIF1	RIF1_HUMAN	GeneCards
**4015**	SLC6A15	SLC6A15_HUMAN	GeneCards
**4016**	SEPTIN11	SEPTIN11_HUMAN	GeneCards

No.	Symbol	Uniprot Name	Database
**4017**	CAMK2D	CAMK2D_HUMAN	GeneCards
**4018**	USP5	USP5_HUMAN	GeneCards
**4019**	GID4	GID4_HUMAN	GeneCards
**4020**	MT-ND4L	MT-ND4L_HUMAN	GeneCards
**4021**	DLAT	DLAT_HUMAN	GeneCards
**4022**	ALDH3A1	ALDH3A1_HUMAN	GeneCards
**4023**	RPS3A	RPS3A_HUMAN	GeneCards
**4024**	PKMYT1	PKMYT1_HUMAN	GeneCards
**4025**	SMN1	SMN1_HUMAN	GeneCards
**4026**	RPIA	RPIA_HUMAN	GeneCards
**4027**	NUF2	NUF2_HUMAN	GeneCards
**4028**	DDX10	DDX10_HUMAN	GeneCards
**4029**	UBA52	UBA52_HUMAN	GeneCards
**4030**	VPS13A	VPS13A_HUMAN	GeneCards
**4031**	RPL21	RPL21_HUMAN	GeneCards
**4032**	RPL23	RPL23_HUMAN	GeneCards
**4033**	TRIM5	TRIM5_HUMAN	GeneCards
**4034**	RPS23	RPS23_HUMAN	GeneCards
**4035**	RNF31	RNF31_HUMAN	GeneCards
**4036**	ABCF2	ABCF2_HUMAN	GeneCards
**4037**	GRID2	GRID2_HUMAN	GeneCards
**4038**	DDX39A	DDX39A_HUMAN	GeneCards
**4039**	MC4R	MC4R_HUMAN	GeneCards
**4040**	COL9A3	COL9A3_HUMAN	GeneCards
**4041**	CDIN1	CDIN1_HUMAN	GeneCards
**4042**	UAP1	UAP1_HUMAN	GeneCards
**4043**	UNC93A	UNC93A_HUMAN	GeneCards
**4044**	MCM8	MCM8_HUMAN	GeneCards
**4045**	MGA	MGA_HUMAN	GeneCards
**4046**	NAA15	NAA15_HUMAN	GeneCards
**4047**	IQCB1	IQCB1_HUMAN	GeneCards
**4048**	SLC33A1	SLC33A1_HUMAN	GeneCards
**4049**	MARS1	MARS1_HUMAN	GeneCards
**4050**	CLCN1	CLCN1_HUMAN	GeneCards
**4051**	ALYREF	ALYREF_HUMAN	GeneCards
**4052**	RPS25	RPS25_HUMAN	GeneCards
**4053**	UBB	UBB_HUMAN	GeneCards
**4054**	HSPA14	HSPA14_HUMAN	GeneCards
**4055**	TRIM8	TRIM8_HUMAN	GeneCards
**4056**	INTS13	INTS13_HUMAN	GeneCards
**4057**	OSBPL8	OSBPL8_HUMAN	GeneCards

No.	Symbol	Uniprot Name	Database
**4058**	PPIB	PPIB_HUMAN	GeneCards
**4059**	ARF1	ARF1_HUMAN	GeneCards
**4060**	RPL18A	RPL18A_HUMAN	GeneCards
**4061**	HSPA1L	HSPA1L_HUMAN	GeneCards
**4062**	LAP3	LAP3_HUMAN	GeneCards
**4063**	WDR13	WDR13_HUMAN	GeneCards
**4064**	PURA	PURA_HUMAN	GeneCards
**4065**	PACSIN3	PACSIN3_HUMAN	GeneCards
**4066**	AGTPBP1	AGTPBP1_HUMAN	GeneCards
**4067**	NUBPL	NUBPL_HUMAN	GeneCards
**4068**	ENOSF1	ENOSF1_HUMAN	GeneCards
**4069**	DPM1	DPM1_HUMAN	GeneCards
**4070**	DYNC1H1	DYNC1H1_HUMAN	GeneCards
**4071**	RPL24	RPL24_HUMAN	GeneCards
**4072**	PRICKLE1	PRICKLE1_HUMAN	GeneCards
**4073**	C11orf58	C11orf58_HUMAN	GeneCards
**4074**	MRPL28	MRPL28_HUMAN	GeneCards
**4075**	DYNC2H1	DYNC2H1_HUMAN	GeneCards
**4076**	RPL14	RPL14_HUMAN	GeneCards
**4077**	PRPF4	PRPF4_HUMAN	GeneCards
**4078**	MRPL1	MRPL1_HUMAN	GeneCards
**4079**	WRNIP1	WRNIP1_HUMAN	GeneCards
**4080**	GSTO1	GSTO1_HUMAN	GeneCards
**4081**	ULK2	ULK2_HUMAN	GeneCards
**4082**	ARSA	ARSA_HUMAN	GeneCards
**4083**	PIGB	PIGB_HUMAN	GeneCards
**4084**	PHF8	PHF8_HUMAN	GeneCards
**4085**	RUFY3	RUFY3_HUMAN	GeneCards
**4086**	PIN4	PIN4_HUMAN	GeneCards
**4087**	KRT2	KRT2_HUMAN	GeneCards
**4088**	POMGNT1	POMGNT1_HUMAN	GeneCards
**4089**	TWNK	TWNK_HUMAN	GeneCards
**4090**	EXD2	EXD2_HUMAN	GeneCards
**4091**	YWHAH	YWHAH_HUMAN	GeneCards
**4092**	ANAPC4	ANAPC4_HUMAN	GeneCards
**4093**	SLC26A4	SLC26A4_HUMAN	GeneCards
**4094**	KAT8	KAT8_HUMAN	GeneCards
**4095**	PPM1B	PPM1B_HUMAN	GeneCards
**4096**	LGR4	LGR4_HUMAN	GeneCards
**4097**	TRA2B	TRA2B_HUMAN	GeneCards
**4098**	CDC14A	CDC14A_HUMAN	GeneCards

No.	Symbol	Uniprot Name	Database
**4099**	ATG2B	ATG2B_HUMAN	GeneCards
**4100**	CYB5R3	CYB5R3_HUMAN	GeneCards
**4101**	RSPH1	RSPH1_HUMAN	GeneCards
**4102**	CALCRL	CALCRL_HUMAN	GeneCards
**4103**	HELQ	HELQ_HUMAN	GeneCards
**4104**	PPA2	PPA2_HUMAN	GeneCards
**4105**	MYOT	MYOT_HUMAN	GeneCards
**4106**	ERH	ERH_HUMAN	GeneCards
**4107**	CALU	CALU_HUMAN	GeneCards
**4108**	TBX2	TBX2_HUMAN	GeneCards
**4109**	ITIH4	ITIH4_HUMAN	GeneCards
**4110**	COQ8A	COQ8A_HUMAN	GeneCards
**4111**	ARPC2	ARPC2_HUMAN	GeneCards
**4112**	PSMD4	PSMD4_HUMAN	GeneCards
**4113**	SMPD4	SMPD4_HUMAN	GeneCards
**4114**	CMPK1	CMPK1_HUMAN	GeneCards
**4115**	RPS18	RPS18_HUMAN	GeneCards
**4116**	L3MBTL1	L3MBTL1_HUMAN	GeneCards
**4117**	TOP3A	TOP3A_HUMAN	GeneCards
**4118**	SUV39H2	SUV39H2_HUMAN	GeneCards
**4119**	DCTN1	DCTN1_HUMAN	GeneCards
**4120**	COP1	COP1_HUMAN	GeneCards
**4121**	SOX6	SOX6_HUMAN	GeneCards
**4122**	NXF1	NXF1_HUMAN	GeneCards
**4123**	STK19	STK19_HUMAN	GeneCards
**4124**	EIF4G2	EIF4G2_HUMAN	GeneCards
**4125**	H2BC5	H2BC5_HUMAN	GeneCards
**4126**	SEC31A	SEC31A_HUMAN	GeneCards
**4127**	UACA	UACA_HUMAN	GeneCards
**4128**	PITPNC1	PITPNC1_HUMAN	GeneCards
**4129**	PRP4K	PRP4K_HUMAN	GeneCards
**4130**	GINS2	GINS2_HUMAN	GeneCards
**4131**	BORCS6	BORCS6_HUMAN	GeneCards
**4132**	CTDP1	CTDP1_HUMAN	GeneCards
**4133**	DTNBP1	DTNBP1_HUMAN	GeneCards
**4134**	MRPS34	MRPS34_HUMAN	GeneCards
**4135**	PPP1R12C	PPP1R12C_HUMAN	GeneCards
**4136**	RPS21	RPS21_HUMAN	GeneCards
**4137**	MAT2B	MAT2B_HUMAN	GeneCards
**4138**	RPS4X	RPS4X_HUMAN	GeneCards
**4139**	LRRC3B	LRRC3B_HUMAN	GeneCards

No.	Symbol	Uniprot Name	Database
**4140**	H2AZ2	H2AZ2_HUMAN	GeneCards
**4141**	SENP2	SENP2_HUMAN	GeneCards
**4142**	LAS1L	LAS1L_HUMAN	GeneCards
**4143**	KRT16	KRT16_HUMAN	GeneCards
**4144**	ORC1	ORC1_HUMAN	GeneCards
**4145**	CCT7	CCT7_HUMAN	GeneCards
**4146**	SLCO2B1	SLCO2B1_HUMAN	GeneCards
**4147**	PSMC3IP	PSMC3IP_HUMAN	GeneCards
**4148**	SNRNP70	SNRNP70_HUMAN	GeneCards
**4149**	CHST11	CHST11_HUMAN	GeneCards
**4150**	MOV10	MOV10_HUMAN	GeneCards
**4151**	LARS1	LARS1_HUMAN	GeneCards
**4152**	GPR143	GPR143_HUMAN	GeneCards
**4153**	PAN2	PAN2_HUMAN	GeneCards
**4154**	DOCK2	DOCK2_HUMAN	GeneCards
**4155**	TWSG1	TWSG1_HUMAN	GeneCards
**4156**	MMS19	MMS19_HUMAN	GeneCards
**4157**	NUCB2	NUCB2_HUMAN	GeneCards
**4158**	MADD	MADD_HUMAN	GeneCards
**4159**	MYBBP1A	MYBBP1A_HUMAN	GeneCards
**4160**	LITAF	LITAF_HUMAN	GeneCards
**4161**	LGI1	LGI1_HUMAN	GeneCards
**4162**	ASH2L	ASH2L_HUMAN	GeneCards
**4163**	SUPT5H	SUPT5H_HUMAN	GeneCards
**4164**	RPS16	RPS16_HUMAN	GeneCards
**4165**	DIDO1	DIDO1_HUMAN	GeneCards
**4166**	DNAJC7	DNAJC7_HUMAN	GeneCards
**4167**	ZFP91	ZFP91_HUMAN	GeneCards
**4168**	MT-RNR1	MT-RNR1_HUMAN	GeneCards
**4169**	NABP1	NABP1_HUMAN	GeneCards
**4170**	GABARAP	GABARAP_HUMAN	GeneCards
**4171**	SCYL1	SCYL1_HUMAN	GeneCards
**4172**	RHBDF1	RHBDF1_HUMAN	GeneCards
**4173**	FILIP1	FILIP1_HUMAN	GeneCards
**4174**	EMD	EMD_HUMAN	GeneCards
**4175**	PQBP1	PQBP1_HUMAN	GeneCards
**4176**	LONP1	LONP1_HUMAN	GeneCards
**4177**	RPLP2	RPLP2_HUMAN	GeneCards
**4178**	LINC01587	LINC01587_HUMAN	GeneCards
**4179**	RAB11FIP2	RAB11FIP2_HUMAN	GeneCards
**4180**	RCOR1	RCOR1_HUMAN	GeneCards

No.	Symbol	Uniprot Name	Database
**4181**	RXFP2	RXFP2_HUMAN	GeneCards
**4182**	PPARGC1B	PPARGC1B_HUMAN	GeneCards
**4183**	SMARCAD1	SMARCAD1_HUMAN	GeneCards
**4184**	RPS13	RPS13_HUMAN	GeneCards
**4185**	RNPEP	RNPEP_HUMAN	GeneCards
**4186**	FASTKD3	FASTKD3_HUMAN	GeneCards
**4187**	RPL37A	RPL37A_HUMAN	GeneCards
**4188**	FKBP8	FKBP8_HUMAN	GeneCards
**4189**	GTF2F1	GTF2F1_HUMAN	GeneCards
**4190**	KRT10-AS1	KRT10-AS1_HUMAN	GeneCards
**4191**	BOLA2	BOLA2_HUMAN	GeneCards
**4192**	NELFA	NELFA_HUMAN	GeneCards
**4193**	NBPF1	NBPF1_HUMAN	GeneCards
**4194**	DRD4	DRD4_HUMAN	GeneCards
**4195**	DLG3	DLG3_HUMAN	GeneCards
**4196**	NR2E3	NR2E3_HUMAN	GeneCards
**4197**	WNT8A	WNT8A_HUMAN	GeneCards
**4198**	CNKSR2	CNKSR2_HUMAN	GeneCards
**4199**	SNX14	SNX14_HUMAN	GeneCards
**4200**	SPAST	SPAST_HUMAN	GeneCards
**4201**	SRRT	SRRT_HUMAN	GeneCards
**4202**	ULBP2	ULBP2_HUMAN	GeneCards
**4203**	GRIK2	GRIK2_HUMAN	GeneCards
**4204**	RPL12	RPL12_HUMAN	GeneCards
**4205**	RNF4	RNF4_HUMAN	GeneCards
**4206**	TECR	TECR_HUMAN	GeneCards
**4207**	FAIM2	FAIM2_HUMAN	GeneCards
**4208**	CD2AP	CD2AP_HUMAN	GeneCards
**4209**	GNG5	GNG5_HUMAN	GeneCards
**4210**	PANK4	PANK4_HUMAN	GeneCards
**4211**	SLC35F2	SLC35F2_HUMAN	GeneCards
**4212**	HDAC10	HDAC10_HUMAN	GeneCards
**4213**	GABARAPL2	GABARAPL2_HUMAN	GeneCards
**4214**	MAGI3	MAGI3_HUMAN	GeneCards
**4215**	RRAGC	RRAGC_HUMAN	GeneCards
**4216**	H2AC13	H2AC13_HUMAN	GeneCards
**4217**	INF2	INF2_HUMAN	GeneCards
**4218**	EMG1	EMG1_HUMAN	GeneCards
**4219**	NIP7	NIP7_HUMAN	GeneCards
**4220**	RPL38	RPL38_HUMAN	GeneCards
**4221**	DYSF	DYSF_HUMAN	GeneCards

No.	Symbol	Uniprot Name	Database
**4222**	AMD1	AMD1_HUMAN	GeneCards
**4223**	STK38	STK38_HUMAN	GeneCards
**4224**	NEIL3	NEIL3_HUMAN	GeneCards
**4225**	EIF3D	EIF3D_HUMAN	GeneCards
**4226**	WWC1	WWC1_HUMAN	GeneCards
**4227**	BDP1	BDP1_HUMAN	GeneCards
**4228**	GNG13	GNG13_HUMAN	GeneCards
**4229**	VPS33B	VPS33B_HUMAN	GeneCards
**4230**	AS3MT	AS3MT_HUMAN	GeneCards
**4231**	CAMK2B	CAMK2B_HUMAN	GeneCards
**4232**	SIDT2	SIDT2_HUMAN	GeneCards
**4233**	ARHGEF5	ARHGEF5_HUMAN	GeneCards
**4234**	RPL30	RPL30_HUMAN	GeneCards
**4235**	HEXIM1	HEXIM1_HUMAN	GeneCards
**4236**	HSDL2	HSDL2_HUMAN	GeneCards
**4237**	RAPGEF1	RAPGEF1_HUMAN	GeneCards
**4238**	AVP	AVP_HUMAN	GeneCards
**4239**	EIF3L	EIF3L_HUMAN	GeneCards
**4240**	TMED1	TMED1_HUMAN	GeneCards
**4241**	POLR2K	POLR2K_HUMAN	GeneCards
**4242**	CTR9	CTR9_HUMAN	GeneCards
**4243**	NKRF	NKRF_HUMAN	GeneCards
**4244**	SLC6A14	SLC6A14_HUMAN	GeneCards
**4245**	PNMT	PNMT_HUMAN	GeneCards
**4246**	TNNI3K	TNNI3K_HUMAN	GeneCards
**4247**	PON2	PON2_HUMAN	GeneCards
**4248**	CINP	CINP_HUMAN	GeneCards
**4249**	TCF25	TCF25_HUMAN	GeneCards
**4250**	GALK1	GALK1_HUMAN	GeneCards
**4251**	SCAF1	SCAF1_HUMAN	GeneCards
**4252**	WASF2	WASF2_HUMAN	GeneCards
**4253**	SCFD1	SCFD1_HUMAN	GeneCards
**4254**	FAM210B	FAM210B_HUMAN	GeneCards
**4255**	RNF126	RNF126_HUMAN	GeneCards
**4256**	UTP20	UTP20_HUMAN	GeneCards
**4257**	RAMP3	RAMP3_HUMAN	GeneCards
**4258**	UBA5	UBA5_HUMAN	GeneCards
**4259**	C14orf93	C14orf93_HUMAN	GeneCards
**4260**	OSBPL11	OSBPL11_HUMAN	GeneCards
**4261**	ROMO1	ROMO1_HUMAN	GeneCards
**4262**	ZNHIT1	ZNHIT1_HUMAN	GeneCards

No.	Symbol	Uniprot Name	Database
**4263**	DNAJC10	DNAJC10_HUMAN	GeneCards
**4264**	CPSF1	CPSF1_HUMAN	GeneCards
**4265**	EEF1B2	EEF1B2_HUMAN	GeneCards
**4266**	PSMC6	PSMC6_HUMAN	GeneCards
**4267**	CDKL5	CDKL5_HUMAN	GeneCards
**4268**	SMOX	SMOX_HUMAN	GeneCards
**4269**	NUDT21	NUDT21_HUMAN	GeneCards
**4270**	HOOK3	HOOK3_HUMAN	GeneCards
**4271**	VBP1	VBP1_HUMAN	GeneCards
**4272**	KRT76	KRT76_HUMAN	GeneCards
**4273**	GAR1	GAR1_HUMAN	GeneCards
**4274**	RNF26	RNF26_HUMAN	GeneCards
**4275**	NTMT1	NTMT1_HUMAN	GeneCards
**4276**	MT-ATP6	MT-ATP6_HUMAN	GeneCards
**4277**	YEATS4	YEATS4_HUMAN	GeneCards
**4278**	FAU	FAU_HUMAN	GeneCards
**4279**	ING2	ING2_HUMAN	GeneCards
**4280**	KRT73	KRT73_HUMAN	GeneCards
**4281**	DHX30	DHX30_HUMAN	GeneCards
**4282**	NSDHL	NSDHL_HUMAN	GeneCards
**4283**	DYNC1I2	DYNC1I2_HUMAN	GeneCards
**4284**	SRRM1	SRRM1_HUMAN	GeneCards
**4285**	PABPN1	PABPN1_HUMAN	GeneCards
**4286**	CXCL6	CXCL6_HUMAN	GeneCards
**4287**	NDUFA10	NDUFA10_HUMAN	GeneCards
**4288**	UBE2D3	UBE2D3_HUMAN	GeneCards
**4289**	NUP133	NUP133_HUMAN	GeneCards
**4290**	SLC35F5	SLC35F5_HUMAN	GeneCards
**4291**	MCOLN1	MCOLN1_HUMAN	GeneCards
**4292**	EIF5	EIF5_HUMAN	GeneCards
**4293**	MAZ	MAZ_HUMAN	GeneCards
**4294**	SLC52A3	SLC52A3_HUMAN	GeneCards
**4295**	BYSL	BYSL_HUMAN	GeneCards
**4296**	DYNLL1	DYNLL1_HUMAN	GeneCards
**4297**	RIOK2	RIOK2_HUMAN	GeneCards
**4298**	TECTA	TECTA_HUMAN	GeneCards
**4299**	RPLP1	RPLP1_HUMAN	GeneCards
**4300**	IPO7	IPO7_HUMAN	GeneCards
**4301**	APCS	APCS_HUMAN	GeneCards
**4302**	DCLRE1A	DCLRE1A_HUMAN	GeneCards
**4303**	IVNS1ABP	IVNS1ABP_HUMAN	GeneCards

No.	Symbol	Uniprot Name	Database
**4304**	IMMT	IMMT_HUMAN	GeneCards
**4305**	STAMBP	STAMBP_HUMAN	GeneCards
**4306**	MPHOSPH8	MPHOSPH8_HUMAN	GeneCards
**4307**	WAC	WAC_HUMAN	GeneCards
**4308**	FEM1C	FEM1C_HUMAN	GeneCards
**4309**	GALT	GALT_HUMAN	GeneCards
**4310**	CLASRP	CLASRP_HUMAN	GeneCards
**4311**	SAP18	SAP18_HUMAN	GeneCards
**4312**	PPP1R9B	PPP1R9B_HUMAN	GeneCards
**4313**	RTCB	RTCB_HUMAN	GeneCards
**4314**	GPS1	GPS1_HUMAN	GeneCards
**4315**	HRNR	HRNR_HUMAN	GeneCards
**4316**	RYK	RYK_HUMAN	GeneCards
**4317**	DARS1	DARS1_HUMAN	GeneCards
**4318**	SPATA13	SPATA13_HUMAN	GeneCards
**4319**	TRPA1	TRPA1_HUMAN	GeneCards
**4320**	ANKS1A	ANKS1A_HUMAN	GeneCards
**4321**	DDX59	DDX59_HUMAN	GeneCards
**4322**	NSF	NSF_HUMAN	GeneCards
**4323**	UCK2	UCK2_HUMAN	GeneCards
**4324**	DERL1	DERL1_HUMAN	GeneCards
**4325**	VPS35	VPS35_HUMAN	GeneCards
**4326**	AGPS	AGPS_HUMAN	GeneCards
**4327**	UBAP1	UBAP1_HUMAN	GeneCards
**4328**	ANKRD12	ANKRD12_HUMAN	GeneCards
**4329**	BAIAP2	BAIAP2_HUMAN	GeneCards
**4330**	CYP21A2	CYP21A2_HUMAN	GeneCards
**4331**	SRRM2	SRRM2_HUMAN	GeneCards
**4332**	MT-ND5	MT-ND5_HUMAN	GeneCards
**4333**	FARP2	FARP2_HUMAN	GeneCards
**4334**	TRAPPC4	TRAPPC4_HUMAN	GeneCards
**4335**	GATAD2A	GATAD2A_HUMAN	GeneCards
**4336**	TTF2	TTF2_HUMAN	GeneCards
**4337**	ARPC3	ARPC3_HUMAN	GeneCards
**4338**	GNG11	GNG11_HUMAN	GeneCards
**4339**	CUTC	CUTC_HUMAN	GeneCards
**4340**	MT-CO3	MT-CO3_HUMAN	GeneCards
**4341**	OPA1	OPA1_HUMAN	GeneCards
**4342**	B3GAT1	B3GAT1_HUMAN	GeneCards
**4343**	MRPL11	MRPL11_HUMAN	GeneCards
**4344**	RBM15B	RBM15B_HUMAN	GeneCards

No.	Symbol	Uniprot Name	Database
**4345**	DCAF8	DCAF8_HUMAN	GeneCards
**4346**	VPS52	VPS52_HUMAN	GeneCards
**4347**	BZW1	BZW1_HUMAN	GeneCards
**4348**	GARS1	GARS1_HUMAN	GeneCards
**4349**	KRT6B	KRT6B_HUMAN	GeneCards
**4350**	ERI2	ERI2_HUMAN	GeneCards
**4351**	COPS3	COPS3_HUMAN	GeneCards
**4352**	DDA1	DDA1_HUMAN	GeneCards
**4353**	ZC3HAV1	ZC3HAV1_HUMAN	GeneCards
**4354**	TRMT2A	TRMT2A_HUMAN	GeneCards
**4355**	VGLL4	VGLL4_HUMAN	GeneCards
**4356**	WDR36	WDR36_HUMAN	GeneCards
**4357**	NDOR1	NDOR1_HUMAN	GeneCards
**4358**	MOK	MOK_HUMAN	GeneCards
**4359**	SUPT6H	SUPT6H_HUMAN	GeneCards
**4360**	FTSJ3	FTSJ3_HUMAN	GeneCards
**4361**	CDKL2	CDKL2_HUMAN	GeneCards
**4362**	TCAF1	TCAF1_HUMAN	GeneCards
**4363**	SYMPK	SYMPK_HUMAN	GeneCards
**4364**	LUC7L	LUC7L_HUMAN	GeneCards
**4365**	EDA	EDA_HUMAN	GeneCards
**4366**	UBE2D2	UBE2D2_HUMAN	GeneCards
**4367**	DNAJB4	DNAJB4_HUMAN	GeneCards
**4368**	OLFML3	OLFML3_HUMAN	GeneCards
**4369**	FKTN	FKTN_HUMAN	GeneCards
**4370**	GRHL1	GRHL1_HUMAN	GeneCards
**4371**	ITPA	ITPA_HUMAN	GeneCards
**4372**	CEP41	CEP41_HUMAN	GeneCards
**4373**	ATXN7	ATXN7_HUMAN	GeneCards
**4374**	ACCS	ACCS_HUMAN	GeneCards
**4375**	FBXO30	FBXO30_HUMAN	GeneCards
**4376**	AKAP8L	AKAP8L_HUMAN	GeneCards
**4377**	UBE4B	UBE4B_HUMAN	GeneCards
**4378**	LRRC59	LRRC59_HUMAN	GeneCards
**4379**	CCM2	CCM2_HUMAN	GeneCards
**4380**	SLC28A3	SLC28A3_HUMAN	GeneCards
**4381**	TMEM164	TMEM164_HUMAN	GeneCards
**4382**	ZFP64	ZFP64_HUMAN	GeneCards
**4383**	CORO1A	CORO1A_HUMAN	GeneCards
**4384**	LUC7L3	LUC7L3_HUMAN	GeneCards
**4385**	RPL36A	RPL36A_HUMAN	GeneCards

No.	Symbol	Uniprot Name	Database
**4386**	ARHGAP8	ARHGAP8_HUMAN	GeneCards
**4387**	TMEM230	TMEM230_HUMAN	GeneCards
**4388**	KRT75	KRT75_HUMAN	GeneCards
**4389**	NHS	NHS_HUMAN	GeneCards
**4390**	ABCB10	ABCB10_HUMAN	GeneCards
**4391**	DNMBP	DNMBP_HUMAN	GeneCards
**4392**	NUP85	NUP85_HUMAN	GeneCards
**4393**	CLINT1	CLINT1_HUMAN	GeneCards
**4394**	DNAJA3	DNAJA3_HUMAN	GeneCards
**4395**	DIMT1	DIMT1_HUMAN	GeneCards
**4396**	MID1	MID1_HUMAN	GeneCards
**4397**	MED14	MED14_HUMAN	GeneCards
**4398**	GOLIM4	GOLIM4_HUMAN	GeneCards
**4399**	QPRT	QPRT_HUMAN	GeneCards
**4400**	EIF4ENIF1	EIF4ENIF1_HUMAN	GeneCards
**4401**	PAF1	PAF1_HUMAN	GeneCards
**4402**	RIOK3	RIOK3_HUMAN	GeneCards
**4403**	NLGN3	NLGN3_HUMAN	GeneCards
**4404**	EXTL3	EXTL3_HUMAN	GeneCards
**4405**	DNAJC6	DNAJC6_HUMAN	GeneCards
**4406**	CLSTN3	CLSTN3_HUMAN	GeneCards
**4407**	WDR48	WDR48_HUMAN	GeneCards
**4408**	MRPS28	MRPS28_HUMAN	GeneCards
**4409**	INCENP	INCENP_HUMAN	GeneCards
**4410**	SERF2	SERF2_HUMAN	GeneCards
**4411**	EP400	EP400_HUMAN	GeneCards
**4412**	SPDL1	SPDL1_HUMAN	GeneCards
**4413**	AGPAT1	AGPAT1_HUMAN	GeneCards
**4414**	GTF3C4	GTF3C4_HUMAN	GeneCards
**4415**	PPP4C	PPP4C_HUMAN	GeneCards
**4416**	PPP4R1	PPP4R1_HUMAN	GeneCards
**4417**	POLA2	POLA2_HUMAN	GeneCards
**4418**	ITPRID1	ITPRID1_HUMAN	GeneCards
**4419**	TUBGCP6	TUBGCP6_HUMAN	GeneCards
**4420**	ZNF346	ZNF346_HUMAN	GeneCards
**4421**	ZYX	ZYX_HUMAN	GeneCards
**4422**	MRTO4	MRTO4_HUMAN	GeneCards
**4423**	ERLIN2	ERLIN2_HUMAN	GeneCards
**4424**	SSR1	SSR1_HUMAN	GeneCards
**4425**	DNAJA2	DNAJA2_HUMAN	GeneCards
**4426**	NT5DC2	NT5DC2_HUMAN	GeneCards

No.	Symbol	Uniprot Name	Database
**4427**	MTCH2	MTCH2_HUMAN	GeneCards
**4428**	WDR26	WDR26_HUMAN	GeneCards
**4429**	MRPS14	MRPS14_HUMAN	GeneCards
**4430**	SUPT16H	SUPT16H_HUMAN	GeneCards
**4431**	FAM210A	FAM210A_HUMAN	GeneCards
**4432**	HBD	HBD_HUMAN	GeneCards
**4433**	GDAP1L1	GDAP1L1_HUMAN	GeneCards
**4434**	PIF1	PIF1_HUMAN	GeneCards
**4435**	NDUFS2	NDUFS2_HUMAN	GeneCards
**4436**	GPD1	GPD1_HUMAN	GeneCards
**4437**	MED4	MED4_HUMAN	GeneCards
**4438**	PYGB	PYGB_HUMAN	GeneCards
**4439**	BMS1	BMS1_HUMAN	GeneCards
**4440**	PAQR3	PAQR3_HUMAN	GeneCards
**4441**	PARD6B	PARD6B_HUMAN	GeneCards
**4442**	CNP	CNP_HUMAN	GeneCards
**4443**	MIPEP	MIPEP_HUMAN	GeneCards
**4444**	TMEM161A	TMEM161A_HUMAN	GeneCards
**4445**	MRPS35	MRPS35_HUMAN	GeneCards
**4446**	ATP5PD	ATP5PD_HUMAN	GeneCards
**4447**	FDFT1	FDFT1_HUMAN	GeneCards
**4448**	DLG4	DLG4_HUMAN	GeneCards
**4449**	SVIL	SVIL_HUMAN	GeneCards
**4450**	GPS2	GPS2_HUMAN	GeneCards
**4451**	RPL32	RPL32_HUMAN	GeneCards
**4452**	ATXN2L	ATXN2L_HUMAN	GeneCards
**4453**	ZNF277	ZNF277_HUMAN	GeneCards
**4454**	PTCD3	PTCD3_HUMAN	GeneCards
**4455**	TAX1BP3	TAX1BP3_HUMAN	GeneCards
**4456**	MED25	MED25_HUMAN	GeneCards
**4457**	SLC23A2	SLC23A2_HUMAN	GeneCards
**4458**	ATP2C1	ATP2C1_HUMAN	GeneCards
**4459**	HNRNPH3	HNRNPH3_HUMAN	GeneCards
**4460**	ZC3H14	ZC3H14_HUMAN	GeneCards
**4461**	RBM28	RBM28_HUMAN	GeneCards
**4462**	RUFY1	RUFY1_HUMAN	GeneCards
**4463**	CDK13	CDK13_HUMAN	GeneCards
**4464**	TRMT10A	TRMT10A_HUMAN	GeneCards
**4465**	LMAN1	LMAN1_HUMAN	GeneCards
**4466**	HNRNPA0	HNRNPA0_HUMAN	GeneCards
**4467**	ODF2	ODF2_HUMAN	GeneCards

No.	Symbol	Uniprot Name	Database
**4468**	SEPTIN7	SEPTIN7_HUMAN	GeneCards
**4469**	GNB1L	GNB1L_HUMAN	GeneCards
**4470**	OTOF	OTOF_HUMAN	GeneCards
**4471**	HP1BP3	HP1BP3_HUMAN	GeneCards
**4472**	MRPL15	MRPL15_HUMAN	GeneCards
**4473**	UBE2F	UBE2F_HUMAN	GeneCards
**4474**	TIMM44	TIMM44_HUMAN	GeneCards
**4475**	RHOU	RHOU_HUMAN	GeneCards
**4476**	ADGRE2	ADGRE2_HUMAN	GeneCards
**4477**	EIF3C	EIF3C_HUMAN	GeneCards
**4478**	ZNF185	ZNF185_HUMAN	GeneCards
**4479**	ESPL1	ESPL1_HUMAN	GeneCards
**4480**	EIF4H	EIF4H_HUMAN	GeneCards
**4481**	NDUFA8	NDUFA8_HUMAN	GeneCards
**4482**	TBRG4	TBRG4_HUMAN	GeneCards
**4483**	ANP32B	ANP32B_HUMAN	GeneCards
**4484**	L3MBTL3	L3MBTL3_HUMAN	GeneCards
**4485**	CHST8	CHST8_HUMAN	GeneCards
**4486**	SLC66A2	SLC66A2_HUMAN	GeneCards
**4487**	PFDN2	PFDN2_HUMAN	GeneCards
**4488**	RPL39	RPL39_HUMAN	GeneCards
**4489**	RUSF1	RUSF1_HUMAN	GeneCards
**4490**	JADE3	JADE3_HUMAN	GeneCards
**4491**	MAGOH	MAGOH_HUMAN	GeneCards
**4492**	TEKT4	TEKT4_HUMAN	GeneCards
**4493**	USP37	USP37_HUMAN	GeneCards
**4494**	HIVEP1	HIVEP1_HUMAN	GeneCards
**4495**	HIRA	HIRA_HUMAN	GeneCards
**4496**	SNRPD1	SNRPD1_HUMAN	GeneCards
**4497**	C1orf35	C1orf35_HUMAN	GeneCards
**4498**	TUBGCP3	TUBGCP3_HUMAN	GeneCards
**4499**	MEPCE	MEPCE_HUMAN	GeneCards
**4500**	NOL6	NOL6_HUMAN	GeneCards
**4501**	GRHPR	GRHPR_HUMAN	GeneCards
**4502**	NANS	NANS_HUMAN	GeneCards
**4503**	TXNDC9	TXNDC9_HUMAN	GeneCards
**4504**	FGG	FGG_HUMAN	GeneCards
**4505**	DNAJB6	DNAJB6_HUMAN	GeneCards
**4506**	DOP1A	DOP1A_HUMAN	GeneCards
**4507**	GTF2E2	GTF2E2_HUMAN	GeneCards
**4508**	NDE1	NDE1_HUMAN	GeneCards

No.	Symbol	Uniprot Name	Database
**4509**	C1D	C1D_HUMAN	GeneCards
**4510**	BAG5	BAG5_HUMAN	GeneCards
**4511**	MAIP1	MAIP1_HUMAN	GeneCards
**4512**	ARHGAP32	ARHGAP32_HUMAN	GeneCards
**4513**	NDUFS3	NDUFS3_HUMAN	GeneCards
**4514**	SCAMP3	SCAMP3_HUMAN	GeneCards
**4515**	THOC3	THOC3_HUMAN	GeneCards
**4516**	RAB18	RAB18_HUMAN	GeneCards
**4517**	COX4I2	COX4I2_HUMAN	GeneCards
**4518**	PCNX1	PCNX1_HUMAN	GeneCards
**4519**	PSMC2	PSMC2_HUMAN	GeneCards
**4520**	RPL37	RPL37_HUMAN	GeneCards
**4521**	STX12	STX12_HUMAN	GeneCards
**4522**	MANF	MANF_HUMAN	GeneCards
**4523**	RAI14	RAI14_HUMAN	GeneCards
**4524**	TRAPPC12	TRAPPC12_HUMAN	GeneCards
**4525**	SAP30BP	SAP30BP_HUMAN	GeneCards
**4526**	MRPL44	MRPL44_HUMAN	GeneCards
**4527**	BAZ1B	BAZ1B_HUMAN	GeneCards
**4528**	ZNF22	ZNF22_HUMAN	GeneCards
**4529**	EIF3G	EIF3G_HUMAN	GeneCards
**4530**	DDX23	DDX23_HUMAN	GeneCards
**4531**	EIF3K	EIF3K_HUMAN	GeneCards
**4532**	PSD	PSD_HUMAN	GeneCards
**4533**	DUSP23	DUSP23_HUMAN	GeneCards
**4534**	TDP2	TDP2_HUMAN	GeneCards
**4535**	P2RY14	P2RY14_HUMAN	GeneCards
**4536**	TNPO3	TNPO3_HUMAN	GeneCards
**4537**	RFX6	RFX6_HUMAN	GeneCards
**4538**	FUT7	FUT7_HUMAN	GeneCards
**4539**	ANKRD36B	ANKRD36B_HUMAN	GeneCards
**4540**	DAD1	DAD1_HUMAN	GeneCards
**4541**	INTS11	INTS11_HUMAN	GeneCards
**4542**	TRA2A	TRA2A_HUMAN	GeneCards
**4543**	MRM3	MRM3_HUMAN	GeneCards
**4544**	GTF3C5	GTF3C5_HUMAN	GeneCards
**4545**	MRPS27	MRPS27_HUMAN	GeneCards
**4546**	ZNF326	ZNF326_HUMAN	GeneCards
**4547**	ZGPAT	ZGPAT_HUMAN	GeneCards
**4548**	PSMD13	PSMD13_HUMAN	GeneCards
**4549**	MFSD11	MFSD11_HUMAN	GeneCards

No.	Symbol	Uniprot Name	Database
**4550**	CHMP2A	CHMP2A_HUMAN	GeneCards
**4551**	MSANTD3	MSANTD3_HUMAN	GeneCards
**4552**	LEO1	LEO1_HUMAN	GeneCards
**4553**	MRPS5	MRPS5_HUMAN	GeneCards
**4554**	ELOF1	ELOF1_HUMAN	GeneCards
**4555**	NABP2	NABP2_HUMAN	GeneCards
**4556**	ZMIZ2	ZMIZ2_HUMAN	GeneCards
**4557**	ARHGEF10	ARHGEF10_HUMAN	GeneCards
**4558**	EPB41L4A	EPB41L4A_HUMAN	GeneCards
**4559**	ZC3H18	ZC3H18_HUMAN	GeneCards
**4560**	CDH12	CDH12_HUMAN	GeneCards
**4561**	NUDT5	NUDT5_HUMAN	GeneCards
**4562**	EDC4	EDC4_HUMAN	GeneCards
**4563**	CLTCL1	CLTCL1_HUMAN	GeneCards
**4564**	STEEP1	STEEP1_HUMAN	GeneCards
**4565**	ZFAND1	ZFAND1_HUMAN	GeneCards
**4566**	CHMP1A	CHMP1A_HUMAN	GeneCards
**4567**	RNPS1	RNPS1_HUMAN	GeneCards
**4568**	ORC6	ORC6_HUMAN	GeneCards
**4569**	ABCB8	ABCB8_HUMAN	GeneCards
**4570**	KRR1	KRR1_HUMAN	GeneCards
**4571**	KCNK9	KCNK9_HUMAN	GeneCards
**4572**	PPIP5K2	PPIP5K2_HUMAN	GeneCards
**4573**	ASAP2	ASAP2_HUMAN	GeneCards
**4574**	KHDC4	KHDC4_HUMAN	GeneCards
**4575**	SPAG6	SPAG6_HUMAN	GeneCards
**4576**	ANKS1B	ANKS1B_HUMAN	GeneCards
**4577**	E2F8	E2F8_HUMAN	GeneCards
**4578**	SLC23A1	SLC23A1_HUMAN	GeneCards
**4579**	DNAJB11	DNAJB11_HUMAN	GeneCards
**4580**	SSNA1	SSNA1_HUMAN	GeneCards
**4581**	POLR2C	POLR2C_HUMAN	GeneCards
**4582**	JPT1	JPT1_HUMAN	GeneCards
**4583**	NARF	NARF_HUMAN	GeneCards
**4584**	PAIP1	PAIP1_HUMAN	GeneCards
**4585**	TRMT10C	TRMT10C_HUMAN	GeneCards
**4586**	HM13	HM13_HUMAN	GeneCards
**4587**	ITSN1	ITSN1_HUMAN	GeneCards
**4588**	DHX16	DHX16_HUMAN	GeneCards
**4589**	ARL1	ARL1_HUMAN	GeneCards
**4590**	WLS	WLS_HUMAN	GeneCards

No.	Symbol	Uniprot Name	Database
**4591**	EMILIN1	EMILIN1_HUMAN	GeneCards
**4592**	PI4KB	PI4KB_HUMAN	GeneCards
**4593**	NELFE	NELFE_HUMAN	GeneCards
**4594**	ERGIC2	ERGIC2_HUMAN	GeneCards
**4595**	RPH3A	RPH3A_HUMAN	GeneCards
**4596**	VPS11	VPS11_HUMAN	GeneCards
**4597**	TDRD3	TDRD3_HUMAN	GeneCards
**4598**	PRPF38A	PRPF38A_HUMAN	GeneCards
**4599**	CLPTM1	CLPTM1_HUMAN	GeneCards
**4600**	NACA	NACA_HUMAN	GeneCards
**4601**	CLK4	CLK4_HUMAN	GeneCards
**4602**	GIT2	GIT2_HUMAN	GeneCards
**4603**	MRPS23	MRPS23_HUMAN	GeneCards
**4604**	RRS1	RRS1_HUMAN	GeneCards
**4605**	WBP4	WBP4_HUMAN	GeneCards
**4606**	PPP6R3	PPP6R3_HUMAN	GeneCards
**4607**	INPP5E	INPP5E_HUMAN	GeneCards
**4608**	MED13	MED13_HUMAN	GeneCards
**4609**	BAZ1A	BAZ1A_HUMAN	GeneCards
**4610**	CCDC80	CCDC80_HUMAN	GeneCards
**4611**	VAT1	VAT1_HUMAN	GeneCards
**4612**	UQCRFS1	UQCRFS1_HUMAN	GeneCards
**4613**	MRPL13	MRPL13_HUMAN	GeneCards
**4614**	ORAI3	ORAI3_HUMAN	GeneCards
**4615**	COG3	COG3_HUMAN	GeneCards
**4616**	CHMP5	CHMP5_HUMAN	GeneCards
**4617**	RPRD2	RPRD2_HUMAN	GeneCards
**4618**	PSMG1	PSMG1_HUMAN	GeneCards
**4619**	YBX2	YBX2_HUMAN	GeneCards
**4620**	ZBTB12	ZBTB12_HUMAN	GeneCards
**4621**	MCMBP	MCMBP_HUMAN	GeneCards
**4622**	HINT2	HINT2_HUMAN	GeneCards
**4623**	SH3BP1	SH3BP1_HUMAN	GeneCards
**4624**	SASS6	SASS6_HUMAN	GeneCards
**4625**	MRPS2	MRPS2_HUMAN	GeneCards
**4626**	TFB1M	TFB1M_HUMAN	GeneCards
**4627**	CRIPT	CRIPT_HUMAN	GeneCards
**4628**	MMAB	MMAB_HUMAN	GeneCards
**4629**	SKIC8	SKIC8_HUMAN	GeneCards
**4630**	LAMTOR3	LAMTOR3_HUMAN	GeneCards
**4631**	SPG21	SPG21_HUMAN	GeneCards

No.	Symbol	Uniprot Name	Database
**4632**	HBS1L	HBS1L_HUMAN	GeneCards
**4633**	DCAF7	DCAF7_HUMAN	GeneCards
**4634**	ACTR5	ACTR5_HUMAN	GeneCards
**4635**	FRY	FRY_HUMAN	GeneCards
**4636**	SUB1	SUB1_HUMAN	GeneCards
**4637**	DNM3	DNM3_HUMAN	GeneCards
**4638**	MIGA2	MIGA2_HUMAN	GeneCards
**4639**	ACSS2	ACSS2_HUMAN	GeneCards
**4640**	DCP1A	DCP1A_HUMAN	GeneCards
**4641**	GMPPA	GMPPA_HUMAN	GeneCards
**4642**	LZTFL1	LZTFL1_HUMAN	GeneCards
**4643**	TRUB2	TRUB2_HUMAN	GeneCards
**4644**	POLR3K	POLR3K_HUMAN	GeneCards
**4645**	RSPRY1	RSPRY1_HUMAN	GeneCards
**4646**	N4BP2	N4BP2_HUMAN	GeneCards
**4647**	BCO1	BCO1_HUMAN	GeneCards
**4648**	CHCHD3	CHCHD3_HUMAN	GeneCards
**4649**	THOC2	THOC2_HUMAN	GeneCards
**4650**	SNRNP40	SNRNP40_HUMAN	GeneCards
**4651**	KIR2DS4	KIR2DS4_HUMAN	GeneCards
**4652**	NKAP	NKAP_HUMAN	GeneCards
**4653**	MCF2	MCF2_HUMAN	GeneCards
**4654**	ALDH3A2	ALDH3A2_HUMAN	GeneCards
**4655**	PRPF40A	PRPF40A_HUMAN	GeneCards
**4656**	BTBD2	BTBD2_HUMAN	GeneCards
**4657**	SARNP	SARNP_HUMAN	GeneCards
**4658**	PDAP1	PDAP1_HUMAN	GeneCards
**4659**	OXSM	OXSM_HUMAN	GeneCards
**4660**	BCAS4	BCAS4_HUMAN	GeneCards
**4661**	ARHGEF16	ARHGEF16_HUMAN	GeneCards
**4662**	POP4	POP4_HUMAN	GeneCards
**4663**	GMPR2	GMPR2_HUMAN	GeneCards
**4664**	TIMM13	TIMM13_HUMAN	GeneCards
**4665**	STK38L	STK38L_HUMAN	GeneCards
**4666**	B3GALT1	B3GALT1_HUMAN	GeneCards
**4667**	KBTBD8	KBTBD8_HUMAN	GeneCards
**4668**	HLX	HLX_HUMAN	GeneCards
**4669**	CYP26B1	CYP26B1_HUMAN	GeneCards
**4670**	FLAD1	FLAD1_HUMAN	GeneCards
**4671**	NOA1	NOA1_HUMAN	GeneCards
**4672**	ZDHHC4	ZDHHC4_HUMAN	GeneCards

No.	Symbol	Uniprot Name	Database
**4673**	PEX1	PEX1_HUMAN	GeneCards
**4674**	YPEL5	YPEL5_HUMAN	GeneCards
**4675**	NDUFA9	NDUFA9_HUMAN	GeneCards
**4676**	RPF2	RPF2_HUMAN	GeneCards
**4677**	TXNDC15	TXNDC15_HUMAN	GeneCards
**4678**	PANX1	PANX1_HUMAN	GeneCards
**4679**	TXNL4A	TXNL4A_HUMAN	GeneCards
**4680**	DTWD1	DTWD1_HUMAN	GeneCards
**4681**	AIRIM	AIRIM_HUMAN	GeneCards
**4682**	FAM193A	FAM193A_HUMAN	GeneCards
**4683**	WFS1	WFS1_HUMAN	GeneCards
**4684**	MAGI1	MAGI1_HUMAN	GeneCards
**4685**	BUD13	BUD13_HUMAN	GeneCards
**4686**	SLC39A12	SLC39A12_HUMAN	GeneCards
**4687**	ABHD10	ABHD10_HUMAN	GeneCards
**4688**	BORCS5	BORCS5_HUMAN	GeneCards
**4689**	LOXL3	LOXL3_HUMAN	GeneCards
**4690**	TXLNA	TXLNA_HUMAN	GeneCards
**4691**	TOMM22	TOMM22_HUMAN	GeneCards
**4692**	AGPAT5	AGPAT5_HUMAN	GeneCards
**4693**	ITSN2	ITSN2_HUMAN	GeneCards
**4694**	NDUFB9	NDUFB9_HUMAN	GeneCards
**4695**	ZNHIT2	ZNHIT2_HUMAN	GeneCards
**4696**	MCCC1	MCCC1_HUMAN	GeneCards
**4697**	TBCA	TBCA_HUMAN	GeneCards
**4698**	GPKOW	GPKOW_HUMAN	GeneCards
**4699**	SIPA1L3	SIPA1L3_HUMAN	GeneCards
**4700**	GLTP	GLTP_HUMAN	GeneCards
**4701**	MICU1	MICU1_HUMAN	GeneCards
**4702**	VAPB	VAPB_HUMAN	GeneCards
**4703**	DCTN2	DCTN2_HUMAN	GeneCards
**4704**	SEPTIN10	SEPTIN10_HUMAN	GeneCards
**4705**	RNF220	RNF220_HUMAN	GeneCards
**4706**	MRPS31	MRPS31_HUMAN	GeneCards
**4707**	PSMG2	PSMG2_HUMAN	GeneCards
**4708**	CDC42SE2	CDC42SE2_HUMAN	GeneCards
**4709**	ERGIC1	ERGIC1_HUMAN	GeneCards
**4710**	ARMC6	ARMC6_HUMAN	GeneCards
**4711**	DCUN1D5	DCUN1D5_HUMAN	GeneCards
**4712**	H4C5	H4C5_HUMAN	GeneCards
**4713**	PHACTR4	PHACTR4_HUMAN	GeneCards

No.	Symbol	Uniprot Name	Database
**4714**	DNAJB7	DNAJB7_HUMAN	GeneCards
**4715**	ABCG1	ABCG1_HUMAN	GeneCards
**4716**	LRCH3	LRCH3_HUMAN	GeneCards
**4717**	MRPS18A	MRPS18A_HUMAN	GeneCards
**4718**	MED8	MED8_HUMAN	GeneCards
**4719**	GCH1	GCH1_HUMAN	GeneCards
**4720**	SLC12A4	SLC12A4_HUMAN	GeneCards
**4721**	VTA1	VTA1_HUMAN	GeneCards
**4722**	MED30	MED30_HUMAN	GeneCards
**4723**	LBX1	LBX1_HUMAN	GeneCards
**4724**	MICU2	MICU2_HUMAN	GeneCards
**4725**	DNAJB2	DNAJB2_HUMAN	GeneCards
**4726**	PPP4R3A	PPP4R3A_HUMAN	GeneCards
**4727**	PPM1A	PPM1A_HUMAN	GeneCards
**4728**	TMEM39A	TMEM39A_HUMAN	GeneCards
**4729**	MBIP	MBIP_HUMAN	GeneCards
**4730**	QTRT1	QTRT1_HUMAN	GeneCards
**4731**	SCAF8	SCAF8_HUMAN	GeneCards
**4732**	DNAJC24	DNAJC24_HUMAN	GeneCards
**4733**	CHMP3	CHMP3_HUMAN	GeneCards
**4734**	SUN2	SUN2_HUMAN	GeneCards
**4735**	STYX	STYX_HUMAN	GeneCards
**4736**	EIF3J	EIF3J_HUMAN	GeneCards
**4737**	CXXC1	CXXC1_HUMAN	GeneCards
**4738**	IMP4	IMP4_HUMAN	GeneCards
**4739**	PMPCB	PMPCB_HUMAN	GeneCards
**4740**	OPTN	OPTN_HUMAN	GeneCards
**4741**	PRRG1	PRRG1_HUMAN	GeneCards
**4742**	NTM	NTM_HUMAN	GeneCards
**4743**	ETF1	ETF1_HUMAN	GeneCards
**4744**	USH1C	USH1C_HUMAN	GeneCards
**4745**	LMAN2	LMAN2_HUMAN	GeneCards
**4746**	DAW1	DAW1_HUMAN	GeneCards
**4747**	MOSPD1	MOSPD1_HUMAN	GeneCards
**4748**	RAET1G	RAET1G_HUMAN	GeneCards
**4749**	MTX1	MTX1_HUMAN	GeneCards
**4750**	TMA7	TMA7_HUMAN	GeneCards
**4751**	STK26	STK26_HUMAN	GeneCards
**4752**	UTP11	UTP11_HUMAN	GeneCards
**4753**	TBCK	TBCK_HUMAN	GeneCards
**4754**	AOC1	AOC1_HUMAN	GeneCards

No.	Symbol	Uniprot Name	Database
**4755**	SALL1	SALL1_HUMAN	GeneCards
**4756**	MPHOSPH6	MPHOSPH6_HUMAN	GeneCards
**4757**	IFRD1	IFRD1_HUMAN	GeneCards
**4758**	ANKEF1	ANKEF1_HUMAN	GeneCards
**4759**	RABGGTA	RABGGTA_HUMAN	GeneCards
**4760**	CDK5RAP1	CDK5RAP1_HUMAN	GeneCards
**4761**	GPBP1L1	GPBP1L1_HUMAN	GeneCards
**4762**	PDCL3	PDCL3_HUMAN	GeneCards
**4763**	FEM1B	FEM1B_HUMAN	GeneCards
**4764**	RBM12B	RBM12B_HUMAN	GeneCards
**4765**	CHRND	CHRND_HUMAN	GeneCards
**4766**	GDE1	GDE1_HUMAN	GeneCards
**4767**	TRIR	TRIR_HUMAN	GeneCards
**4768**	C8B	C8B_HUMAN	GeneCards
**4769**	CEP76	CEP76_HUMAN	GeneCards
**4770**	UQCRH	UQCRH_HUMAN	GeneCards
**4771**	UBAC1	UBAC1_HUMAN	GeneCards
**4772**	GSTA3	GSTA3_HUMAN	GeneCards
**4773**	CRIP2	CRIP2_HUMAN	GeneCards
**4774**	HOMER2	HOMER2_HUMAN	GeneCards
**4775**	MPPED2	MPPED2_HUMAN	GeneCards
**4776**	TUBD1	TUBD1_HUMAN	GeneCards
**4777**	CISD1	CISD1_HUMAN	GeneCards
**4778**	CARMIL1	CARMIL1_HUMAN	GeneCards
**4779**	ANKRD13A	ANKRD13A_HUMAN	GeneCards
**4780**	CERS5	CERS5_HUMAN	GeneCards
**4781**	GTF2H2	GTF2H2_HUMAN	GeneCards
**4782**	CTDNEP1	CTDNEP1_HUMAN	GeneCards
**4783**	VRK3	VRK3_HUMAN	GeneCards
**4784**	MPST	MPST_HUMAN	GeneCards
**4785**	MRPS12	MRPS12_HUMAN	GeneCards
**4786**	C1QTNF4	C1QTNF4_HUMAN	GeneCards
**4787**	GAREM1	GAREM1_HUMAN	GeneCards
**4788**	MED22	MED22_HUMAN	GeneCards
**4789**	H3C14	H3C14_HUMAN	GeneCards
**4790**	MRPL42	MRPL42_HUMAN	GeneCards
**4791**	SUCLG1	SUCLG1_HUMAN	GeneCards
**4792**	IER3IP1	IER3IP1_HUMAN	GeneCards
**4793**	RAB34	RAB34_HUMAN	GeneCards
**4794**	DNAJB12	DNAJB12_HUMAN	GeneCards
**4795**	HUNK	HUNK_HUMAN	GeneCards

No.	Symbol	Uniprot Name	Database
**4796**	MGST3	MGST3_HUMAN	GeneCards
**4797**	CCDC174	CCDC174_HUMAN	GeneCards
**4798**	MRPL32	MRPL32_HUMAN	GeneCards
**4799**	TSNAX	TSNAX_HUMAN	GeneCards
**4800**	TMEM209	TMEM209_HUMAN	GeneCards
**4801**	MRPS18C	MRPS18C_HUMAN	GeneCards
**4802**	CLNS1A	CLNS1A_HUMAN	GeneCards
**4803**	THOC7	THOC7_HUMAN	GeneCards
**4804**	HSPBP1	HSPBP1_HUMAN	GeneCards
**4805**	YAF2	YAF2_HUMAN	GeneCards
**4806**	RNF38	RNF38_HUMAN	GeneCards
**4807**	GPM6B	GPM6B_HUMAN	GeneCards
**4808**	VAMP3	VAMP3_HUMAN	GeneCards
**4809**	FIBP	FIBP_HUMAN	GeneCards
**4810**	SETD6	SETD6_HUMAN	GeneCards
**4811**	DSPP	DSPP_HUMAN	GeneCards
**4812**	SH3GLB2	SH3GLB2_HUMAN	GeneCards
**4813**	DNAJB8	DNAJB8_HUMAN	GeneCards
**4814**	COL6A2	COL6A2_HUMAN	GeneCards
**4815**	MKLN1	MKLN1_HUMAN	GeneCards
**4816**	CAMKV	CAMKV_HUMAN	GeneCards
**4817**	DCTPP1	DCTPP1_HUMAN	GeneCards
**4818**	KCTD9	KCTD9_HUMAN	GeneCards
**4819**	MKRN2	MKRN2_HUMAN	GeneCards
**4820**	TIMMDC1	TIMMDC1_HUMAN	GeneCards
**4821**	PTPN23	PTPN23_HUMAN	GeneCards
**4822**	ITIH3	ITIH3_HUMAN	GeneCards
**4823**	LNX2	LNX2_HUMAN	GeneCards
**4824**	ANKRD27	ANKRD27_HUMAN	GeneCards
**4825**	B3GLCT	B3GLCT_HUMAN	GeneCards
**4826**	SLC25A26	SLC25A26_HUMAN	GeneCards
**4827**	KAZN	KAZN_HUMAN	GeneCards
**4828**	SC5D	SC5D_HUMAN	GeneCards
**4829**	HECTD3	HECTD3_HUMAN	GeneCards
**4830**	IMPG1	IMPG1_HUMAN	GeneCards
**4831**	TOM1L2	TOM1L2_HUMAN	GeneCards
**4832**	CBX6	CBX6_HUMAN	GeneCards
**4833**	RIC8A	RIC8A_HUMAN	GeneCards
**4834**	CDC14B	CDC14B_HUMAN	GeneCards
**4835**	MRPL20	MRPL20_HUMAN	GeneCards
**4836**	REXO4	REXO4_HUMAN	GeneCards

No.	Symbol	Uniprot Name	Database
**4837**	MRPL17	MRPL17_HUMAN	GeneCards
**4838**	MED26	MED26_HUMAN	GeneCards
**4839**	LRRC4C	LRRC4C_HUMAN	GeneCards
**4840**	MT-ND6	MT-ND6_HUMAN	GeneCards
**4841**	SPAG16	SPAG16_HUMAN	GeneCards
**4842**	MRPS24	MRPS24_HUMAN	GeneCards
**4843**	CYRIB	CYRIB_HUMAN	GeneCards
**4844**	TLK2	TLK2_HUMAN	GeneCards
**4845**	FBXO46	FBXO46_HUMAN	GeneCards
**4846**	KIFAP3	KIFAP3_HUMAN	GeneCards
**4847**	RASD1	RASD1_HUMAN	GeneCards
**4848**	AGXT	AGXT_HUMAN	GeneCards
**4849**	SKIDA1	SKIDA1_HUMAN	GeneCards
**4850**	FAM117B	FAM117B_HUMAN	GeneCards
**4851**	SNX27	SNX27_HUMAN	GeneCards
**4852**	MRPL24	MRPL24_HUMAN	GeneCards
**4853**	TSSK1B	TSSK1B_HUMAN	GeneCards
**4854**	WDR46	WDR46_HUMAN	GeneCards
**4855**	RNF5	RNF5_HUMAN	GeneCards
**4856**	DENND2B	DENND2B_HUMAN	GeneCards
**4857**	TMEM126A	TMEM126A_HUMAN	GeneCards
**4858**	VAMP1	VAMP1_HUMAN	GeneCards
**4859**	ZCRB1	ZCRB1_HUMAN	GeneCards
**4860**	EFHC1	EFHC1_HUMAN	GeneCards
**4861**	DDX28	DDX28_HUMAN	GeneCards
**4862**	CPVL	CPVL_HUMAN	GeneCards
**4863**	ARHGEF17	ARHGEF17_HUMAN	GeneCards
**4864**	RHOT2	RHOT2_HUMAN	GeneCards
**4865**	MRPL49	MRPL49_HUMAN	GeneCards
**4866**	PARP10	PARP10_HUMAN	GeneCards
**4867**	ENOPH1	ENOPH1_HUMAN	GeneCards
**4868**	NDRG3	NDRG3_HUMAN	GeneCards
**4869**	WIPF2	WIPF2_HUMAN	GeneCards
**4870**	HMOX2	HMOX2_HUMAN	GeneCards
**4871**	DDX31	DDX31_HUMAN	GeneCards
**4872**	UGP2	UGP2_HUMAN	GeneCards
**4873**	SEC61A2	SEC61A2_HUMAN	GeneCards
**4874**	GTPBP3	GTPBP3_HUMAN	GeneCards
**4875**	CYB5B	CYB5B_HUMAN	GeneCards
**4876**	CDC42EP2	CDC42EP2_HUMAN	GeneCards
**4877**	ZSWIM7	ZSWIM7_HUMAN	GeneCards

No.	Symbol	Uniprot Name	Database
**4878**	OTUD7A	OTUD7A_HUMAN	GeneCards
**4879**	MSL3	MSL3_HUMAN	GeneCards
**4880**	DCXR	DCXR_HUMAN	GeneCards
**4881**	CWC25	CWC25_HUMAN	GeneCards
**4882**	ZNF410	ZNF410_HUMAN	GeneCards
**4883**	SH3BGRL2	SH3BGRL2_HUMAN	GeneCards
**4884**	ELF2	ELF2_HUMAN	GeneCards
**4885**	TVP23B	TVP23B_HUMAN	GeneCards
**4886**	ABHD16A	ABHD16A_HUMAN	GeneCards
**4887**	PRPSAP2	PRPSAP2_HUMAN	GeneCards
**4888**	PPIL1	PPIL1_HUMAN	GeneCards
**4889**	RPE65	RPE65_HUMAN	GeneCards
**4890**	RNLS	RNLS_HUMAN	GeneCards
**4891**	TBC1D22A	TBC1D22A_HUMAN	GeneCards
**4892**	PHC2	PHC2_HUMAN	GeneCards
**4893**	DYNLRB2	DYNLRB2_HUMAN	GeneCards
**4894**	XPNPEP3	XPNPEP3_HUMAN	GeneCards
**4895**	RNPC3	RNPC3_HUMAN	GeneCards
**4896**	SCUBE1	SCUBE1_HUMAN	GeneCards
**4897**	UTP25	UTP25_HUMAN	GeneCards
**4898**	NIT2	NIT2_HUMAN	GeneCards
**4899**	EMC4	EMC4_HUMAN	GeneCards
**4900**	VPS29	VPS29_HUMAN	GeneCards
**4901**	GGT7	GGT7_HUMAN	GeneCards
**4902**	POMP	POMP_HUMAN	GeneCards
**4903**	DRAP1	DRAP1_HUMAN	GeneCards
**4904**	FNDC11	FNDC11_HUMAN	GeneCards
**4905**	STAU2	STAU2_HUMAN	GeneCards
**4906**	MRPL22	MRPL22_HUMAN	GeneCards
**4907**	MRPS10	MRPS10_HUMAN	GeneCards
**4908**	TBC1D22B	TBC1D22B_HUMAN	GeneCards
**4909**	GFM1	GFM1_HUMAN	GeneCards
**4910**	KCTD6	KCTD6_HUMAN	GeneCards
**4911**	PPP6R1	PPP6R1_HUMAN	GeneCards
**4912**	ELOVL2	ELOVL2_HUMAN	GeneCards
**4913**	SIKE1	SIKE1_HUMAN	GeneCards
**4914**	ZNG1A	ZNG1A_HUMAN	GeneCards
**4915**	SDF2L1	SDF2L1_HUMAN	GeneCards
**4916**	SEC24C	SEC24C_HUMAN	GeneCards
**4917**	TMEM9	TMEM9_HUMAN	GeneCards
**4918**	FBXO42	FBXO42_HUMAN	GeneCards

No.	Symbol	Uniprot Name	Database
**4919**	POLE4	POLE4_HUMAN	GeneCards
**4920**	AGMAT	AGMAT_HUMAN	GeneCards
**4921**	PDIA2	PDIA2_HUMAN	GeneCards
**4922**	RCCD1	RCCD1_HUMAN	GeneCards
**4923**	NUP155	NUP155_HUMAN	GeneCards
**4924**	CRIM1	CRIM1_HUMAN	GeneCards
**4925**	KRT79	KRT79_HUMAN	GeneCards
**4926**	WDR70	WDR70_HUMAN	GeneCards
**4927**	SLC1A2	SLC1A2_HUMAN	GeneCards
**4928**	TMEM258	TMEM258_HUMAN	GeneCards
**4929**	FBXL3	FBXL3_HUMAN	GeneCards
**4930**	RPUSD4	RPUSD4_HUMAN	GeneCards
**4931**	MED31	MED31_HUMAN	GeneCards
**4932**	GTF3C1	GTF3C1_HUMAN	GeneCards
**4933**	IWS1	IWS1_HUMAN	GeneCards
**4934**	H3C10	H3C10_HUMAN	GeneCards
**4935**	VPS37A	VPS37A_HUMAN	GeneCards
**4936**	CEP135	CEP135_HUMAN	GeneCards
**4937**	ASNSD1	ASNSD1_HUMAN	GeneCards
**4938**	C8orf44	C8orf44_HUMAN	GeneCards
**4939**	UTP15	UTP15_HUMAN	GeneCards
**4940**	FCGBP	FCGBP_HUMAN	GeneCards
**4941**	CTDSPL2	CTDSPL2_HUMAN	GeneCards
**4942**	NUP37	NUP37_HUMAN	GeneCards
**4943**	USE1	USE1_HUMAN	GeneCards
**4944**	RPUSD3	RPUSD3_HUMAN	GeneCards
**4945**	CEP57L1	CEP57L1_HUMAN	GeneCards
**4946**	MRPL16	MRPL16_HUMAN	GeneCards
**4947**	FSD1	FSD1_HUMAN	GeneCards
**4948**	RORB	RORB_HUMAN	GeneCards
**4949**	TRAK2	TRAK2_HUMAN	GeneCards
**4950**	CLIC5	CLIC5_HUMAN	GeneCards
**4951**	PHC1	PHC1_HUMAN	GeneCards
**4952**	GIMAP1	GIMAP1_HUMAN	GeneCards
**4953**	ZBTB21	ZBTB21_HUMAN	GeneCards
**4954**	XPO7	XPO7_HUMAN	GeneCards
**4955**	MARCHF7	MARCHF7_HUMAN	GeneCards
**4956**	ZNG1E	ZNG1E_HUMAN	GeneCards
**4957**	NBPF6	NBPF6_HUMAN	GeneCards
**4958**	APOBEC2	APOBEC2_HUMAN	GeneCards
**4959**	SRPRB	SRPRB_HUMAN	GeneCards

No.	Symbol	Uniprot Name	Database
**4960**	ENY2	ENY2_HUMAN	GeneCards
**4961**	KICS2	KICS2_HUMAN	GeneCards
**4962**	KCNIP4	KCNIP4_HUMAN	GeneCards
**4963**	SLC25A40	SLC25A40_HUMAN	GeneCards
**4964**	MRPL57	MRPL57_HUMAN	GeneCards
**4965**	MRPS33	MRPS33_HUMAN	GeneCards
**4966**	RPP14	RPP14_HUMAN	GeneCards
**4967**	MON1A	MON1A_HUMAN	GeneCards
**4968**	CLIP2	CLIP2_HUMAN	GeneCards
**4969**	MRPL48	MRPL48_HUMAN	GeneCards
**4970**	GOLGA7	GOLGA7_HUMAN	GeneCards
**4971**	ZNF507	ZNF507_HUMAN	GeneCards
**4972**	TRIOBP	TRIOBP_HUMAN	GeneCards
**4973**	KRT77	KRT77_HUMAN	GeneCards
**4974**	CLN6	CLN6_HUMAN	GeneCards
**4975**	KATNAL1	KATNAL1_HUMAN	GeneCards
**4976**	GCC1	GCC1_HUMAN	GeneCards
**4977**	EMILIN2	EMILIN2_HUMAN	GeneCards
**4978**	TTC33	TTC33_HUMAN	GeneCards
**4979**	TANC2	TANC2_HUMAN	GeneCards
**4980**	RNF121	RNF121_HUMAN	GeneCards
**4981**	ZCCHC10	ZCCHC10_HUMAN	GeneCards
**4982**	ADISSP	ADISSP_HUMAN	GeneCards
**4983**	EIF1	EIF1_HUMAN	GeneCards
**4984**	ABCB9	ABCB9_HUMAN	GeneCards
**4985**	DISP1	DISP1_HUMAN	GeneCards
**4986**	ZDHHC6	ZDHHC6_HUMAN	GeneCards
**4987**	MINK1	MINK1_HUMAN	GeneCards
**4988**	RNF152	RNF152_HUMAN	GeneCards
**4989**	UBTD2	UBTD2_HUMAN	GeneCards
**4990**	LRCH1	LRCH1_HUMAN	GeneCards
**4991**	SNF8	SNF8_HUMAN	GeneCards
**4992**	INTS5	INTS5_HUMAN	GeneCards
**4993**	SNX18	SNX18_HUMAN	GeneCards
**4994**	TMEM9B	TMEM9B_HUMAN	GeneCards
**4995**	ABHD14B	ABHD14B_HUMAN	GeneCards
**4996**	NTAQ1	NTAQ1_HUMAN	GeneCards
**4997**	ADGRA3	ADGRA3_HUMAN	GeneCards
**4998**	ACTR8	ACTR8_HUMAN	GeneCards
**4999**	CAV3	CAV3_HUMAN	GeneCards
**5000**	HOOK2	HOOK2_HUMAN	GeneCards

No.	Symbol	Uniprot Name	Database
**5001**	ANKRD10	ANKRD10_HUMAN	GeneCards
**5002**	KANSL2	KANSL2_HUMAN	GeneCards
**5003**	BRWD1	BRWD1_HUMAN	GeneCards
**5004**	KRT33B	KRT33B_HUMAN	GeneCards
**5005**	ELP6	ELP6_HUMAN	GeneCards
**5006**	CASD1	CASD1_HUMAN	GeneCards
**5007**	TMEM259	TMEM259_HUMAN	GeneCards
**5008**	RPGR	RPGR_HUMAN	GeneCards
**5009**	ANO3	ANO3_HUMAN	GeneCards
**5010**	TXLNG	TXLNG_HUMAN	GeneCards
**5011**	ZNF518B	ZNF518B_HUMAN	GeneCards
**5012**	CASKIN2	CASKIN2_HUMAN	GeneCards
**5013**	GABRA1	GABRA1_HUMAN	GeneCards
**5014**	RDH14	RDH14_HUMAN	GeneCards
**5015**	SMOC1	SMOC1_HUMAN	GeneCards
**5016**	MTHFSD	MTHFSD_HUMAN	GeneCards
**5017**	SPSB1	SPSB1_HUMAN	GeneCards
**5018**	B3GNT2	B3GNT2_HUMAN	GeneCards
**5019**	PTCHD3	PTCHD3_HUMAN	GeneCards
**5020**	ZBTB44	ZBTB44_HUMAN	GeneCards
**5021**	SUGCT	SUGCT_HUMAN	GeneCards
**5022**	PPP3CB	PPP3CB_HUMAN	GeneCards
**5023**	RFX7	RFX7_HUMAN	GeneCards
**5024**	ZWINT	ZWINT_HUMAN	GeneCards
**5025**	PARD6G	PARD6G_HUMAN	GeneCards
**5026**	TRMO	TRMO_HUMAN	GeneCards
**5027**	TP53RK	TP53RK_HUMAN	GeneCards
**5028**	MTUS2	MTUS2_HUMAN	GeneCards
**5029**	RABAC1	RABAC1_HUMAN	GeneCards
**5030**	OARD1	OARD1_HUMAN	GeneCards
**5031**	SLC38A6	SLC38A6_HUMAN	GeneCards
**5032**	SLC25A17	SLC25A17_HUMAN	GeneCards
**5033**	LMAN2L	LMAN2L_HUMAN	GeneCards
**5034**	HACL1	HACL1_HUMAN	GeneCards
**5035**	TECTB	TECTB_HUMAN	GeneCards
**5036**	RHOV	RHOV_HUMAN	GeneCards
**5037**	ARL15	ARL15_HUMAN	GeneCards
**5038**	HS3ST3B1	HS3ST3B1_HUMAN	GeneCards
**5039**	KLHDC2	KLHDC2_HUMAN	GeneCards
**5040**	CLEC3A	CLEC3A_HUMAN	GeneCards
**5041**	ISOC1	ISOC1_HUMAN	GeneCards

No.	Symbol	Uniprot Name	Database
**5042**	CHCHD2	CHCHD2_HUMAN	GeneCards
**5043**	PANK1	PANK1_HUMAN	GeneCards
**5044**	OSR2	OSR2_HUMAN	GeneCards
**5045**	NPEPL1	NPEPL1_HUMAN	GeneCards
**5046**	TBC1D10B	TBC1D10B_HUMAN	GeneCards
**5047**	METRNL	METRNL_HUMAN	GeneCards
**5048**	TIMM10	TIMM10_HUMAN	GeneCards
**5049**	TST	TST_HUMAN	GeneCards
**5050**	RPAIN	RPAIN_HUMAN	GeneCards
**5051**	WDR45	WDR45_HUMAN	GeneCards
**5052**	CNIH1	CNIH1_HUMAN	GeneCards
**5053**	ILKAP	ILKAP_HUMAN	GeneCards
**5054**	OSBPL7	OSBPL7_HUMAN	GeneCards
**5055**	SLC41A2	SLC41A2_HUMAN	GeneCards
**5056**	IER5L	IER5L_HUMAN	GeneCards
**5057**	KRT3	KRT3_HUMAN	GeneCards
**5058**	ZSCAN9	ZSCAN9_HUMAN	GeneCards
**5059**	TFCP2L1	TFCP2L1_HUMAN	GeneCards
**5060**	COQ7	COQ7_HUMAN	GeneCards
**5061**	ACSF2	ACSF2_HUMAN	GeneCards
**5062**	MAP3K19	MAP3K19_HUMAN	GeneCards
**5063**	LY86	LY86_HUMAN	GeneCards
**5064**	MNS1	MNS1_HUMAN	GeneCards
**5065**	INTS9	INTS9_HUMAN	GeneCards
**5066**	GCM1	GCM1_HUMAN	GeneCards
**5067**	ELOVL1	ELOVL1_HUMAN	GeneCards
**5068**	ZBTB14	ZBTB14_HUMAN	GeneCards
**5069**	TRAPPC3	TRAPPC3_HUMAN	GeneCards
**5070**	CCDC28B	CCDC28B_HUMAN	GeneCards
**5071**	SLC13A5	SLC13A5_HUMAN	GeneCards
**5072**	PHETA1	PHETA1_HUMAN	GeneCards
**5073**	ACAP3	ACAP3_HUMAN	GeneCards
**5074**	SLC52A1	SLC52A1_HUMAN	GeneCards
**5075**	AJAP1	AJAP1_HUMAN	GeneCards
**5076**	SCMH1	SCMH1_HUMAN	GeneCards
**5077**	C5orf15	C5orf15_HUMAN	GeneCards
**5078**	AASS	AASS_HUMAN	GeneCards
**5079**	STYXL1	STYXL1_HUMAN	GeneCards
**5080**	KIR3DL3	KIR3DL3_HUMAN	GeneCards
**5081**	KIAA1191	KIAA1191_HUMAN	GeneCards
**5082**	MINDY4	MINDY4_HUMAN	GeneCards

No.	Symbol	Uniprot Name	Database
**5083**	EFCAB2	EFCAB2_HUMAN	GeneCards
**5084**	PLBD2	PLBD2_HUMAN	GeneCards
**5085**	RASD2	RASD2_HUMAN	GeneCards
**5086**	EDEM3	EDEM3_HUMAN	GeneCards
**5087**	FAM3A	FAM3A_HUMAN	GeneCards
**5088**	IFT57	IFT57_HUMAN	GeneCards
**5089**	FERRY3	FERRY3_HUMAN	GeneCards
**5090**	IQCH	IQCH_HUMAN	GeneCards
**5091**	PPP2R3C	PPP2R3C_HUMAN	GeneCards
**5092**	YIF1A	YIF1A_HUMAN	GeneCards
**5093**	YPEL3	YPEL3_HUMAN	GeneCards
**5094**	LRFN5	LRFN5_HUMAN	GeneCards
**5095**	RPP21	RPP21_HUMAN	GeneCards
**5096**	CEP162	CEP162_HUMAN	GeneCards
**5097**	KIF9	KIF9_HUMAN	GeneCards
**5098**	VSTM2L	VSTM2L_HUMAN	GeneCards
**5099**	RAET1E	RAET1E_HUMAN	GeneCards
**5100**	LETM2	LETM2_HUMAN	GeneCards
**5101**	RILPL1	RILPL1_HUMAN	GeneCards
**5102**	RUSC1	RUSC1_HUMAN	GeneCards
**5103**	MYL11	MYL11_HUMAN	GeneCards
**5104**	COLEC12	COLEC12_HUMAN	GeneCards
**5105**	BTBD10	BTBD10_HUMAN	GeneCards
**5106**	TBC1D16	TBC1D16_HUMAN	GeneCards
**5107**	RAB20	RAB20_HUMAN	GeneCards
**5108**	TEX9	TEX9_HUMAN	GeneCards
**5109**	POLR2J3	POLR2J3_HUMAN	GeneCards
**5110**	TRMT12	TRMT12_HUMAN	GeneCards
**5111**	NIF3L1	NIF3L1_HUMAN	GeneCards
**5112**	SLC17A1	SLC17A1_HUMAN	GeneCards
**5113**	WDR91	WDR91_HUMAN	GeneCards
**5114**	MXD3	MXD3_HUMAN	GeneCards
**5115**	MSGN1	MSGN1_HUMAN	GeneCards
**5116**	TSPAN18	TSPAN18_HUMAN	GeneCards
**5117**	DNAJC4	DNAJC4_HUMAN	GeneCards
**5118**	OSER1	OSER1_HUMAN	GeneCards
**5119**	TLE6	TLE6_HUMAN	GeneCards
**5120**	RALYL	RALYL_HUMAN	GeneCards
**5121**	SDR16C5	SDR16C5_HUMAN	GeneCards
**5122**	KIR3DX1	KIR3DX1_HUMAN	GeneCards
**5123**	TM2D2	TM2D2_HUMAN	GeneCards

No.	Symbol	Uniprot Name	Database
**5124**	POLR2J2	POLR2J2_HUMAN	GeneCards
**5125**	ASGR1	ASGR1_HUMAN	GeneCards
**5126**	DEXI	DEXI_HUMAN	GeneCards
**5127**	KLRC3	KLRC3_HUMAN	GeneCards
**5128**	NT5C3B	NT5C3B_HUMAN	GeneCards
**5129**	RAB40C	RAB40C_HUMAN	GeneCards
**5130**	CIAO2A	CIAO2A_HUMAN	GeneCards
**5131**	PDIK1L	PDIK1L_HUMAN	GeneCards
**5132**	STPG2	STPG2_HUMAN	GeneCards
**5133**	ACTA2	ACTA_HUMAN	CTD
**5134**	BAK1	BAK_HUMAN	CTD
**5135**	LPL	LIPL_HUMAN	CTD
**5136**	ID3	ID3_HUMAN	CTD
**5137**	HSPA2	HSP72_HUMAN	CTD
**5138**	SLC22A1	S22A1_HUMAN	CTD
**5139**	SERTAD1	SRTD1_HUMAN	CTD
**5140**	TPM3	TPM3_HUMAN	CTD
**5141**	CBR1	CBR1_HUMAN	CTD
**5142**	CYP26A1	CP26A_HUMAN	CTD
**5143**	FZD2	FZD2_HUMAN	CTD
**5144**	SH3KBP1	SH3K1_HUMAN	CTD
**5145**	RHOJ	RHOJ_HUMAN	CTD
**5146**	ACTG2	ACTH_HUMAN	CTD
**5147**	GSTM5	GSTM5_HUMAN	CTD
**5148**	CDH5	CADH5_HUMAN	CTD
**5149**	CKM	KCRM_HUMAN	CTD
**5150**	RFC1	RFC1_HUMAN	CTD
**5151**	ATP7A	ATP7A_HUMAN	CTD
**5152**	A2M	A2MG_HUMAN	CTD
**5153**	GPNMB	GPNMB_HUMAN	CTD
**5154**	MAF	MAF_HUMAN	CTD
**5155**	ALOX12B	LX12B_HUMAN	CTD
**5156**	GSTM3	GSTM3_HUMAN	CTD
**5157**	ACHE	ACES_HUMAN	CTD
**5158**	ABCD3	ABCD3_HUMAN	CTD
**5159**	CACNA2D1	CA2D1_HUMAN	CTD
**5160**	DONSON	DONS_HUMAN	CTD
**5161**	CBX5	CBX5_HUMAN	CTD
**5162**	LAMA1	LAMA1_HUMAN	CTD
**5163**	ABCB6	ABCB6_HUMAN	CTD
**5164**	ABCE1	ABCE1_HUMAN	CTD

No.	Symbol	Uniprot Name	Database
**5165**	ABCA3	ABCA3_HUMAN	CTD
**5166**	ABCB11	ABCBB_HUMAN	CTD
**5167**	ABCA5	ABCA5_HUMAN	CTD
**5168**	COL7A1	CO7A1_HUMAN	CTD
**5169**	PRKD1	KPCD1_HUMAN	CTD
**5170**	ABCA12	ABCAC_HUMAN	CTD
**5171**	SFRP4	SFRP4_HUMAN	CTD
**5172**	PPP1R14A	PP14A_HUMAN	CTD
**5173**	RASL11A	RSLBA_HUMAN	CTD
**5174**	CABLES1	CABL1_HUMAN	CTD
**5175**	FBN2	FBN2_HUMAN	CTD
**5176**	ABCC6	MRP6_HUMAN	CTD
**5177**	CABYR	CABYR_HUMAN	CTD
**5178**	TCERG1L	TCRGL_HUMAN	CTD
**5179**	SLC22A15	S22AF_HUMAN	CTD
**5180**	BOC	BOC_HUMAN	CTD
**5181**	PGM1	PGM1_HUMAN	CTD
**5182**	CHD1	CHD1_HUMAN	CTD
**5183**	PCP4	PCP4_HUMAN	CTD
**5184**	DEFA5	DEF5_HUMAN	CTD
**5185**	PAX8	PAX8_HUMAN	CTD
**5186**	GRIN2A	NMDE1_HUMAN	CTD
**5187**	TRIM28	TIF1B_HUMAN	CTD
**5188**	DPAGT1	GPT_HUMAN	CTD
**5189**	SFXN1	SFXN1_HUMAN	CTD
**5190**	NME2	NDKB_HUMAN	CTD
**5191**	HOXD1	HXD1_HUMAN	CTD
**5192**	CD109	CD109_HUMAN	CTD
**5193**	NTNG1	NTNG1_HUMAN	CTD
**5194**	GNB4	GBB4_HUMAN	CTD
**5195**	FHL3	FHL3_HUMAN	CTD
**5196**	JPH3	JPH3_HUMAN	CTD
**5197**	IPP	IPP_HUMAN	CTD
**5198**	PAIP2	PAIP2_HUMAN	CTD
**5199**	RNF182	RN182_HUMAN	CTD
**5200**	ABCA13	ABCAD_HUMAN	CTD
**5201**	SMAD9	SMAD9_HUMAN	CTD
**5202**	FOXL2	FOXL2_HUMAN	CTD
**5203**	ICAM5	ICAM5_HUMAN	CTD
**5204**	BOLL	BOLL_HUMAN	CTD
**5205**	CALCOCO2	CACO2_HUMAN	CTD

No.	Symbol	Uniprot Name	Database
**5206**	PMM2	PMM2_HUMAN	CTD
**5207**	HAPLN1	HPLN1_HUMAN	CTD
**5208**	NT5C	NT5C_HUMAN	CTD
**5209**	PPOX	PPOX_HUMAN	CTD
**5210**	ABCD2	ABCD2_HUMAN	CTD
**5211**	ABCA6	ABCA6_HUMAN	CTD
**5212**	ABCA9	ABCA9_HUMAN	CTD
**5213**	ABCC8	ABCC8_HUMAN	CTD
**5214**	ABCD4	ABCD4_HUMAN	CTD
**5215**	STARD8	STAR8_HUMAN	CTD
**5216**	GRID1	GRID1_HUMAN	CTD
**5217**	TDRG1	TDRG1_HUMAN	CTD
**5218**	ZNF432	ZN432_HUMAN	CTD
**5219**	ABCA4	ABCA4_HUMAN	CTD
**5220**	SH3TC1	S3TC1_HUMAN	CTD
**5221**	HOXD9	HXD9_HUMAN	CTD
**5222**	GUCY1A2	GCYA2_HUMAN	CTD
**5223**	TRMT11	TRM11_HUMAN	CTD
**5224**	PPM1E	PPM1E_HUMAN	CTD
**5225**	ZNF569	ZN569_HUMAN	CTD
**5226**	CNPPD1	CNPD1_HUMAN	CTD
**5227**	CDH7	CADH7_HUMAN	CTD
**5228**	ABCA10	ABCAA_HUMAN	CTD
**5229**	CPAMD8	CPMD8_HUMAN	CTD
**5230**	NEURL1	NEUL1_HUMAN	CTD
**5231**	PRR5-ARHGAP8	B1AHC3_HUMAN	CTD
**5232**	PRR5-ARHGAP8	B1AHC4_HUMAN	CTD
**5233**	PRR5-ARHGAP8	H0Y9T8_HUMAN	CTD
**5234**	OXT	NEU1_HUMAN	CTD
**5235**	RNF34	RNF34_HUMAN	CTD
**5236**	IFNA1	IFNA1_HUMAN	CTD
**5237**	FMO3	FMO3_HUMAN	CTD

Colorectal cancer-related genes were retrieved from NCBI, GeneCards, CTD, and TTD databases. Gene symbols were standardized using UniProtKB. Duplicate entries and genes lacking validated protein annotations were removed prior to intersection analysis.

Table S2. Degree centrality analysis of the compound–target interaction network of *Paris polyphylla* in colorectal cancer.

Name	Degree	Genes target
Diosmetin	107	ABCC1, CYP1B1, XDH, CA2, CA7, CA12, CA4, AKR1B1, CDK5R1, CDK5, CCNB3, CDK1, CCNB1, CCNB2, ARG1, PLG, PTPRS, ABCB1, APP, NOX4, MAOA, FLT3, ALOX5, ADORA1, GLO1, GSK3B, MMP9, MMP2, ABCG2, SYK, PARP1, TTR, MMP12, CD38, AKR1B10, TNKS2, TNKS, TOP1, PIM1, ADORA2A, ACHE, CDK6, CYP19A1, PLA2G2A, TERT, ESR1, ESR2, CSNK2A1, HSD17B1, CBR1, OPRD1, IGF1R, EGFR, ALOX15, ALOX12, HSD17B2, CA1, CA9, KIT, CDK2, F2, CDK1, PTGS2, CFTR, AVPR2, AURKB, DRD4, MPO, PIK3R1, DAPK1, PYGL, SRC, PTK2, KDR, MMP13, MMP3, CA3, PLK1, CA6, PKN1, CA14, MET, NEK2, CXCR1, CAMK2B, ALK, AKT1, NEK6, PLA2G1B, CA5A, BACE1, AXL, NUAK1, AKR1C2, AKR1C1, AKR1C3, AKR1C4, CA13, AKR1A1, MCL1, GPR35, ST6GAL1, AMY1A, GRK6, CYP1A1, CYP2C8, PKM
Spirostanol	64	HSP90AA1, NR1H3, PSEN2, PSENEN, NCSTN, APH1A, PSEN1, APH1B, HSD11B1, PDE10A, CYP2C9, CYP3A4, CYP2C19, CES2, ADORA1, ADORA2A, CNR1, CNR2, IL6ST, HSD3B2, ALK, PCSK7, F2R, SMO, PIK3CD, PIK3CB, PIK3CG, PIK3CA, COL4A3BP, MDM2, CYP24A1, AKR1C3, NR3C1, PYGL, LSS, GRM2, NR1H4, PTGES, PTGS2, FASN, MTNR1A, MTNR1B, INSR, AR, HSD11B2, PRKCA, MAPK14, GRM1, NOS2, MAPK8, KDR, REN, VEGFA, FGF1, FGF2, HPSE, HCRTR2, HCRTR1, MTOR, TBXAS1, OPRL1, OXTR, PTGS1, HPGDS
20-Hydroxyecdysone	60	CYP19A1, FNTA, FNTB, SHBG, SIGMAR1, SERPINA6, TNF, CYP17A1, HSD11B1, NOS2, NR3C2, PRKCH, CDC25A, NPC1L1, BACE1, SRD5A2, BCHE, RORA, ITGAL, AR, NR3C1, ADORA3, ESR1, ESR2, SLC6A3, CES2, PTGES, HSD11B2, POLB, PGR, AKR1B10, IDO1, PTPN11, ALOX5, MAPK3, FDFT1, PTPN1, PTPN2, CES1, SLC6A4, MAPK8, MAPK9, AURKA, DRD1, DRD2, DRD3, SRD5A1, NR1I2, HSD17B3, G6PD, FABP1, SLC22A6, CYP51A1, PGK1, SF3B3, EGFR, IL6, GLUL, SYK, MTOR
Pennogenin	54	IL2, STAT3, SHH, NPC1L1, HSD11B1, RORC, EPHB4, PSEN2, PSENEN, NCSTN, APH1A, PSEN1, APH1B, CYP2C9, CYP3A4, CYP2C19, CDK2, CCNA1, CCNA2, CFD, PTAFR, HSP90AA1, SMO, MTNR1A, MTNR1B, KCNH2, PDE2A, PDE4B, ALK, NR1H3, PTPN1, PDGFRB, KIT, ADORA1, ADORA2A, PYGL, MAPK14, MDM2, MTOR, PIK3CA, PDE10A, MAP3K14, OPRL1, OPRD1, SYK, CCNT1, ACHE, PIK3CB, CCR1, BCL2L1, PIK3CD, PIK3CG, MAP2K1, DGAT1
Diosgenin	43	NR1H3, IL2, SHH, PTPN1, NPC1L1, ALK, MDM2, NR1H2, PDE10A, DGAT1, PCSK7, KCNA3, MTNR1A, MTNR1B, OPRL1, NR1H4, OPRM1, TACR1, CYP2C9, CYP3A4, FASN, PTGS1, MAPK8, MAPK14, CYP24A1, SMO, ADORA1, ADORA2A, INSR, PTK6, KIT, PIK3CB, PIK3CD, PIK3CG, RASGRP3, PIK3CA, AVPR1A, PDGFRB, CFD, CYP51A1, COL4A3BP, PYGL, PGK1
Dextrin	34	CDK1, VEGFA, FGF1, HPSE, FGF2, LGALS4, LGALS8, PSEN2, PSENEN, NCSTN, APH1A, PSEN1, APH1B, LGALS3, HSP90AA1, HTR2B, ADRA2A, ADRA2C, ADRA2B, DRD1, DRD2, ADRA1D, HTR2A, HTR2C, DRD3, CYP2D6, HTR6, ADRA1A, HTR1B, RORC, STAT3, SLC6A2, AMY2A, TRPV1

Name	Degree	Genes target
Pregnane-3,20-diol	31	SHBG, AR, ESR1, ESR2, NR1H4, GPBAR1, HSD11B1, SHH, TRPM8, CA4, CDC25A, CDC25B, NR1I3, CA2, CA1, UGT2B7, POLA1, G6PD, SERPINA6, NPC1L1, CYP19A1, GABBR1, NR3C1, SIGMAR1, NR1H3, CES2, AKR1B10, POLB, TBXAS1, STS, PGR
Flavone	30	CYP19A1, CA2, ADORA1, ADORA2A, CA1, PARP1, CA12, CA9, TNKS2, TNKS, ABCG2, PRKDC, MAOB, PDE5A, TBXAS1, CYP1A1, TACR2, CYP1A2, CYP1B1, PLA2G4A, FYN, PLA2G2A, AR, MAPK3, CALM1, ADORA3, ADORA2B, CASP3, ABCC1, TENC1
Kaempferol 3-gentiobioside-7-rhamnoside	11	NMUR2, ADRA2A, ADRA2C, ACHE, RPS6KA3, CA2, CA7, CA12, CA4, NQO2, NOX4
Prosapogenin A	2	STAT3, IL2
Diosgenin tetraglycoside	1	STAT3
Polyphyllin E (RG)	1	IL2

Degree values were calculated using the Network Analyzer tool in Cytoscape. The “Genes target” column lists colorectal cancer-associated genes interacting with each compound.

Table S3. Protein–protein interaction (PPI) network data, including degree, betweenness centrality, and closeness centrality.

Name	Degree	BetweennessCentrality	ClosenessCentrality
STAT3	46	0.0968	0.4968
EGFR	46	0.0752	0.5016
SRC	43	0.1315	0.4937
HSP90AA1	42	0.1301	0.4787
IL6	40	0.1073	0.4801
AKT1	37	0.0384	0.4787
CASP3	30	0.0428	0.4486
TNF	30	0.0525	0.4499
ESR1	30	0.1440	0.4861
PIK3CA	26	0.0064	0.4187
PTGS2	25	0.0981	0.4448
MAPK3	24	0.0284	0.4551
PIK3R1	24	0.0054	0.4164
PTPN11	24	0.0037	0.3975
CYP3A4	22	0.0510	0.3560
MDM2	21	0.0299	0.4220
FGF2	20	0.0067	0.4005
MTOR	20	0.0062	0.4278
PIK3CD	20	0.0014	0.3729
PIK3CB	20	0.0014	0.3729
IGF1R	20	0.0049	0.4132
BCL2L1	19	0.0051	0.4089
PGR	19	0.0172	0.4232
MET	19	0.0042	0.4089
MAPK14	18	0.0061	0.4232
PTK2	18	0.0035	0.4110
MMP9	18	0.0072	0.4243
MAPK8	18	0.0032	0.4266
CCNB1	17	0.0137	0.3703
KDR	17	0.0024	0.3801
PARP1	17	0.0385	0.4026
CDK2	17	0.0184	0.3915
CDK1	17	0.0130	0.3783
CYP2C9	16	0.0080	0.3576
CYP1A1	16	0.0457	0.3905
KIT	16	0.0089	0.4067
GSK3B	16	0.0102	0.4057
PDGFRB	16	0.0016	0.3848

Name	Degree	BetweennessCentrality	ClosenessCentrality
CYP2C19	15	0.0117	0.3626
PIK3CG	15	0.0016	0.3738
UGT2B7	14	0.0082	0.3146
AR	14	0.0245	0.4187
CCNA2	14	0.0050	0.3560
CYP2C8	13	0.0037	0.3536
APP	13	0.0255	0.3975
CYP1A2	13	0.0075	0.3319
PTGS1	12	0.0071	0.3703
PLK1	12	0.0033	0.3413
PRKCA	12	0.0296	0.3886
MAP2K1	12	0.0023	0.4015
CYP2D6	11	0.0400	0.3896
MMP2	11	0.0009	0.4153
NR3C1	11	0.0136	0.4057
MCL1	11	0.0100	0.3783
ALOX15	11	0.0037	0.3473
CCNB2	11	0.0003	0.3013
IL2	11	0.0048	0.3877
PLA2G4A	10	0.0053	0.3609
AURKA	10	0.0060	0.3520
CCNA1	10	0.0007	0.3140
HPGDS	10	0.0031	0.3504
INSR	10	0.0001	0.3552
ESR2	10	0.0026	0.3925
PTPN1	10	0.0003	0.3668
SYK	10	0.0007	0.3703
MAOB	9	0.0563	0.3406
CYP17A1	9	0.0082	0.3466
ALOX12	9	0.0003	0.3312
ALOX5	9	0.0003	0.3312
AURKB	9	0.0019	0.3384
MAPK9	9	0.0005	0.3774
CBR1	8	0.0390	0.3043
ALK	8	0.0003	0.3694
IDO1	8	0.0313	0.3801
MMP3	8	0.0014	0.3747
FGF1	8	0.0002	0.3609
IL6ST	8	0.0004	0.3576
ABCB1	7	0.0125	0.3820
NR1I2	7	0.0022	0.3191

Name	Degree	BetweennessCentrality	ClosenessCentrality
PSEN1	7	0.0036	0.3376
PRKDC	7	0.0050	0.3839
NOS2	7	0.0017	0.3935
PLA2G2A	7	0.0008	0.3435
CDK5	7	0.0029	0.3198
NR3C2	7	0.0039	0.3576
NEK2	7	0.0000	0.2935
HSD11B1	7	0.0052	0.3369
CYP1B1	7	0.0013	0.3191
AKR1C1	6	0.0005	0.2996
TRPV1	6	0.0764	0.3473
CDK6	6	0.0024	0.3489
ACHE	5	0.0088	0.3244
BACE1	5	0.0008	0.3043
AXL	5	0.0000	0.3660
CYP24A1	5	0.9000	1.0000
SLC6A3	5	0.0193	0.3198
SHH	5	0.0036	0.3473
CCNB3	5	0.0000	0.2935
MPO	5	0.0103	0.3576
HSD17B1	5	0.0001	0.2990
HSD11B2	5	0.0010	0.3140
LGALS3	5	0.0029	0.3593
ABCG2	4	0.0151	0.2985
XDH	4	0.0143	0.3326
SRD5A2	4	0.0022	0.3271
TERT	4	0.0000	0.3481
APH1A	4	0.0010	0.3172
ARG1	4	0.0002	0.3504
PTGES	4	0.0083	0.3264
CES1	4	0.0044	0.3054
KCNH2	4	0.0311	0.3765
REN	4	0.0024	0.3443
RORC	4	0.0127	0.3512
OPRM1	4	0.0050	0.2799
CCR1	3	0.0004	0.3497
CD38	3	0.0002	0.3369
CES2	3	0.0000	0.2712
DRD4	3	0.0016	0.2726
SLC6A4	3	0.0127	0.2557
PTPN2	3	0.0000	0.3584

Name	Degree	BetweennessCentrality	ClosenessCentrality
FASN	3	0.0253	0.3305
F2	3	0.0008	0.3369
F2R	3	0.0005	0.3333
MMP12	3	0.0000	0.3376
CFTR	2	0.0143	0.3443
ADORA2A	2	0.0127	0.2071
CNR1	2	0.0253	0.2599
AKR1B1	2	0.0000	0.2340
AKR1B10	2	0.0000	0.2340
CA12	2	0.0000	0.6250
CA9	2	0.0000	0.6250
CAMK2B	2	0.0013	0.2764
DAPK1	2	0.0000	0.3271
CXCR1	2	0.0000	0.3257
CSNK2A1	2	0.0000	0.3278
SHBG	2	0.0000	0.3285
NR1H2	2	0.0000	0.2492
NR1H3	2	0.0000	0.2492
PGK1	2	0.0008	0.3257
MMP13	2	0.0000	0.3348
SIGMAR1	2	0.0023	0.2844
LGALS4	2	0.0001	0.3066
TNKS	2	0.0000	0.3002
TNKS2	2	0.0000	0.3002
PKM	2	0.0001	0.2985
TACR1	2	0.0000	0.2634
TOP1	2	0.0000	0.2940
SMO	2	0.0002	0.2829
ABCC1	1	0.0000	0.2302
ADORA2B	1	0.0000	0.1718
AKR1A1	1	0.0000	0.2336
CA2	1	0.0000	0.5556
CA4	1	0.0000	0.5556
CA7	1	0.0000	0.5556
NR1H4	1	0.0000	0.3278
GLO1	1	0.0000	0.2545
MAP3K14	1	0.0000	0.3244
HTR2B	1	0.0000	0.2039
KCNA3	1	0.0000	0.2740
MTNR1A	1	0.0000	1.0000
MTNR1B	1	0.0000	1.0000

Name	Degree	BetweennessCentrality	ClosenessCentrality
NOX4	1	0.0000	0.2500
POLB	1	0.0000	0.2875
PIM1	1	0.0000	0.3326
RORA	1	0.0000	0.2604
TRPM8	1	0.0000	0.2582

Table S4. Data of genes target and KEGG pathway network construction.

KEGG pathway	Degree	Genes target
Pathways in cancer	49	CAMK2B, HSP90AA1, AR, MTOR, VEGFA, ESR2, CCNA1, PIK3R1, SHH, TERT, STAT3, PDGFRB, MAPK3, KIT, BCL2L1, CCNA2, MDM2, F2, MMP2, EGFR, GSK3B, CASP3, PIK3CB, PIK3CA, SMO, PIM1, PIK3CD, PTGS2, PTK2, IL6ST, MMP9, MET, PRKCA, CDK6, IGF1R, FGF1, ESR1, IL6, FGF2, DAPK1, AKT1, MAP2K1, NOS2, CDK2, MAPK8, MAPK9, F2R, IL2, ALK
EGFR tyrosine kinase inhibitor resistance	22	PIK3CD, KDR, PDGFRB, AKT1, MAPK3, MAP2K1, SRC, AXL, BCL2L1, MTOR, MET, VEGFA, PRKCA, IGF1R, EGFR, IL6, GSK3B, PIK3R1, PIK3CB, FGF2, PIK3CA, STAT3
Proteoglycans in cancer	30	PIK3CD, CAMK2B, PTPN11, SRC, PTK2, MTOR, MMP9, MET, VEGFA, PRKCA, IGF1R, HPSE, ESR1, PIK3R1, SHH, FGF2, TNF, STAT3, MAPK14, KDR, AKT1, MAPK3, MAP2K1, MDM2, MMP2, EGFR, CASP3, PIK3CB, SMO, PIK3CA
Endocrine resistance	21	MAPK14, PIK3CD, CYP2D6, AKT1, MAPK3, MAP2K1, SRC, PTK2, MTOR, MMP9, MDM2, ESR2, IGF1R, MMP2, MAPK8, EGFR, ESR1, PIK3R1, MAPK9, PIK3CB, PIK3CA
Progesterone-mediated oocyte maturation	22	MAPK14, PIK3CD, HSP90AA1, CCNB3, PLK1, AKT1, MAPK3, MAP2K1, CCNB1, CCNB2, CCNA2, CDK2, CDK1, IGF1R, MAPK8, CCNA1, PIK3R1, MAPK9, PIK3CB, PGR, AURKA, PIK3CA
